# Conserved white-rot enzymatic mechanism for wood decay in the Basidiomycota genus *Pycnoporus*

**DOI:** 10.1093/dnares/dsaa011

**Published:** 2020-06-12

**Authors:** Shingo Miyauchi, Hayat Hage, Elodie Drula, Laurence Lesage-Meessen, Jean-Guy Berrin, David Navarro, Anne Favel, Delphine Chaduli, Sacha Grisel, Mireille Haon, François Piumi, Anthony Levasseur, Anne Lomascolo, Steven Ahrendt, Kerrie Barry, Kurt M LaButti, Didier Chevret, Chris Daum, Jérôme Mariette, Christophe Klopp, Daniel Cullen, Ronald P de Vries, Allen C Gathman, Matthieu Hainaut, Bernard Henrissat, Kristiina S Hildén, Ursula Kües, Walt Lilly, Anna Lipzen, Miia R Mäkelä, Angel T Martinez, Mélanie Morel-Rouhier, Emmanuelle Morin, Jasmyn Pangilinan, Arthur F J Ram, Han A B Wösten, Francisco J Ruiz-Dueñas, Robert Riley, Eric Record, Igor V Grigoriev, Marie-Noëlle Rosso

**Affiliations:** 1 INRAE, UMR1163, Biodiversity and Biotechnology of Fungi, Aix Marseille University, 13009 Marseille, France; 2 INRAE, UMR1136, Interactions Arbres/Microorganismes, Université de Lorraine, Nancy, France; 3 INRAE, CIRM-CF, UMR1163, Aix Marseille University, Marseille, France; 4 US Department of Energy, Joint Genome Institute, Walnut Creek, CA, USA; 5 INRAE, UMR1319, Micalis, Plateforme d’Analyse Protéomique de Paris Sud-Ouest, Jouy-en-Josas, France; 6 INRAE, Genotoul Bioinfo, UR875, Mathématiques et Informatique Appliquées de Toulouse, Castanet-Tolosan, France; 7 USDA Forest Products Laboratory, Madison, WI, USA; 8 Fungal Physiology, Westerdijk Fungal Biodiversity Institute and Fungal Molecular Physiology, Utrecht University, Utrecht, The Netherlands; 9 Department of Microbiology, University of Helsinki, Helsinki, Finland; 10 Department of Biology, Southeast Missouri State University, Cape Girardeau, MI, USA; 11 CNRS, UMR7257, AFMB, Aix Marseille University, Marseille, France; 12 INRAE, USC1408, AFMB, Marseille, France; 13 Department of Molecular Wood Biotechnology and Technical Mycology, Büsgen-Institute, Georg-August-University Göttingen, Göttingen, Germany; 14 Center for Molecular Biosciences (GZMB), Georg-August-University Göttingen, Göttingen, Germany; 15 Centro de Investigaciones Biológicas, CSIC, Madrid, Spain; 16 Molecular Microbiology and Biotechnology, Institute of Biology Leiden, Leiden University, Leiden, The Netherlands; 17 Microbiology, Utrecht University, Utrecht, The Netherlands; 18 Department of Plant and Microbial Biology, University of California Berkeley, Berkeley, CA, USA

**Keywords:** wood decay, lignocellulose, CAZyme, lytic polysaccharide monooxygenase, Class II Peroxidase

## Abstract

White-rot (WR) fungi are pivotal decomposers of dead organic matter in forest ecosystems and typically use a large array of hydrolytic and oxidative enzymes to deconstruct lignocellulose. However, the extent of lignin and cellulose degradation may vary between species and wood type. Here, we combined comparative genomics, transcriptomics and secretome proteomics to identify conserved enzymatic signatures at the onset of wood-decaying activity within the Basidiomycota genus *Pycnoporus*. We observed a strong conservation in the genome structures and the repertoires of protein-coding genes across the four *Pycnoporus* species described to date, despite the species having distinct geographic distributions. We further analysed the early response of *P. cinnabarinus*, *P. coccineus* and *P. sanguineus* to diverse (ligno)-cellulosic substrates. We identified a conserved set of enzymes mobilized by the three species for breaking down cellulose, hemicellulose and pectin. The co-occurrence in the exo-proteomes of H_2_O_2_-producing enzymes with H_2_O_2_-consuming enzymes was a common feature of the three species, although each enzymatic partner displayed independent transcriptional regulation. Finally, cellobiose dehydrogenase-coding genes were systematically co-regulated with at least one AA9 lytic polysaccharide monooxygenase gene, indicative of enzymatic synergy *in vivo*. This study highlights a conserved core white-rot fungal enzymatic mechanism behind the wood-decaying process.

## 1. Introduction

Saprotrophic fungi of Northern Hemisphere and tropical forests impact the carbon cycling through mineralization and alteration of C storage in wood and litter dead organic matter.^1^^,^^2^ White-rot (WR) fungi are wood decayers with the capacity to mineralize lignin with ultimate formation of CO_2_ and H_2_O.^3^. WR fungi deploy a wide arsenal of hydrolytic and oxidative enzymes to degrade wood and their genomes typically contain genes coding for glycoside hydrolases, carbohydrate esterases and polysaccharide lyases that collectively cleave cellulose, hemicellulose and pectin backbones and lateral chains, and oxidative enzymes that target the highly recalcitrant lignin, crystalline cellulose or cellulose-bound xylan.^4^^,^^5^ Beyond these shared features, several studies have highlighted significant polymorphism between WR fungi regarding their ability to selectively degrade lignin over cellulose^6–8^ and in the gene portfolios involved in lignocellulose breakdown.^9–11^ Scarce studies at the intra-genus level have shown that functional diversity between species may arise from diversity in gene content.^12^^,^^13^

Among WR fungi, the genus *Pycnoporus* (Basidiomycota, Agaricomycetes) has been studied for the efficiency of lignin degradation, the capacity to secrete laccases and biotechnological applications related to aromatic compound functionalization, biopolymer synthesis and biomass pre-treatment in the pulp and paper industry.^14^ Four *Pycnoporus* species have been differentiated,^15^^,^^16^ which form a monophyletic group within the Trametes clade.^17^ The four species are found in different geo-climatic areas with limited geographical overlap; *P. cinnabarinus* is widely distributed in the Northern hemisphere*, P. coccineus* is found in countries bordering the Indian and Pacific Oceans, *P. sanguineus* is found in the tropics and subtropics of both hemispheres and *P. puniceus* is found in paleotropical areas.^15^^,^^16^ The four species are found on stumps and either standing or fallen trunks of deciduous trees.

Our aim was to investigate the *Pycnoporus* intra-genus genomic and functional diversity focusing on lignocellulose breakdown. We examined whether divergence in distinct geographic areas had led to genomic diversity, and if there was a signature of conserved enzymatic mechanics in terms of transcriptome and exo-proteome responses to lignocellulosic substrates. We sequenced the genomes of *P. coccineus*, *P. sanguineus* and *P*. *puniceus* monokaryotic strains. We overviewed the genomic features among the three species, in comparison to the previously sequenced genome of *P. cinnabarinus*^9^ and to other evolutionarily related wood-decay fungi. Then, we captured the transcriptomic and exoproteomic responses of three *Pycnoporus* species to a panel of cellulosic and lignocellulosic substrates representative of Gramineae and hardwoods. The focus was the early responses to the substrates in order to minimize inter-species differences influenced by varied growth abilities on the substrates. Our omics integrative approach enabled to identify a common set of lignocellulose-degrading enzymes mobilized by the fungi at the initial stage of lignocellulose degradation, leading to discoveries of genus-wide conserved expression patterns indicative of conserved enzymatic synergies.

## 2. Materials and methods

### 2.1. Genome sequencing and assembly

The monokaryotic strains *P. coccineus* BRFM 310, *P. sanguineus* BRFM 1264 and *P. puniceus* BRFM 1868 were generated after fruiting of the parental strains BRFM 66 (IMB WOO6-2), BRFM 902 and BRFM 1856, respectively, as described previously^14^ (Supplementary Information). All strains were maintained at the International Centre of Microbial Resources (CIRM; https://www6.inra.fr/cirm/). The *P. coccineus* BRFM 310 genome was sequenced using the Illumina platform (99.4×) and assembled with AllPathsLG version R46652^18^ (GenBank accession number: NCSW00000000). The *P. sanguineus* BRFM 1264 genome was sequenced using 454 (16.8×) and Solexa (87×) technologies and assembled with CABOG^19^ (GenBank accession number: VOCM00000000). The *P. puniceus* BRFM 1868 genome was sequenced using PacBio technology (97×) and assembled with FALCON, improved with finisherSC,^20^ polished with Quiver (GenBank accession number: VICQ00000000). The three genomes were annotated using the JGI annotation pipeline,^21^ which takes multiple inputs (scaffolds, ESTs and known genes) and runs several analytical tools for gene prediction and annotation, and deposits the results in the JGI Genome Portal MycoCosm (http://genome.jgi.doe.gov/fungi). The previously sequenced and annotated genome of *P. cinnabarinus* BRFM 137^9^ was also deposited in MycoCosm.

### 2.2. Construction of phylogenetic tree

We constructed a phylogeny based on orthologous genes among the selected fungi using FastOrtho with the parameters set to 50% identity, 50% coverage and inflation 3.0.^22^ The protein sequences used for the process were downloaded from the JGI fungal portal MycoCosm. We identified the clusters of orthologous genes with single copy genes, aligned the sequences of each cluster with MAFFT 7.221,^23^ eliminated ambiguous regions (containing gaps and poorly aligned regions) and concatenated the alignments with Gblocks 0.91b.^24^ We constructed a phylogenetic tree with RAxML 7.7.2^25^ using the standard algorithm, the PROTGAMMAWAG model of sequence evolution and 500 bootstrap replicates.

### 2.3. Comparative genomic analysis

Genome completeness with single copy orthologues was calculated using BUSCO v3.0.2 with default parameters.^26^ The coverage of transposable elements (TEs) in genomes was calculated using a custom pipeline transposon identification nominative genome overview.^27^ The counts for plant cell wall (PCW)-degrading enzymes, predicted secreted auxiliary activity (AA) enzymes and fungal cell wall (FCW)-degrading enzymes were combined and visualized with custom R scripts, proteomic information navigated genomic outlook^28^ incorporating R packages ggplot2, ggtree and egg.^29–31^

### 2.4. Expert functional annotations

Genes from the *A-* and *B*-mating type loci were identified and manually curated as described in Kues et al.^32^ CAZymes and AA were annotated as in Lombard et al.^33^ Gene models from AA2 Class II peroxidases, AA3_2 glucose-methanol-choline (GMC)-oxidoreductases and AA5 copper radical oxidases were further inspected by sequence-by-sequence exhaustive analysis and phylogenetic analysis. Peptidases were annotated using Blastx searches of gene models against InterPro and MEROPS databases^34^^,^^35^ followed by manual curation. Glutathione transferases (GST)-coding genes were annotated with a combination of automated blastp using functionally characterized GST sequences from *P. chrysosporium*,^36–38^ phylogenetic analysis and active site comparison. Genes coding for hydrophobins, laccases, the secretory pathway and carbon catabolism were also manually inspected. Proteins were predicted secreted if they fulfilled three conditions: (i) presence of a secretion signal peptide, (ii) absence of endoplasmic reticulum retention motif and (iii) absence of transmembrane helix outside the signal peptide, as previously described.^39^ Predicted secreted proteins shorter than 300 amino acids were annotated as small secreted proteins (SSPs). Detailed analyses of expert annotations are provided in the Supporting Information file.

### 2.5. Cultures

Media for cultures on agar plates contained diammonium tartrate (1.84 g/l), yeast nitrogen base (0.17 g/l), agar (15 g/l) and were supplemented with either maltose (20 g/l), Avicel (AVI) PH 101 (Fluka) (15 g/l), ground and sifted wheat straw (WS) fragments <2 mm (15 g/l), *Pinus halepensis* pine wood fragments <2 mm (15 g/l) or *Populus tremuloides* Wiley-milled aspen (Asp) fragments (180 µm < fragment size < 2 mm; 15 g/l). The plates were inoculated with one fungal disk (4 mm diameter) of 7-day-old mycelia and incubated at 30 °C. Liquid cultures were maintained at 30 °C in a rotary shaker at 120 rpm in 250-ml Erlenmeyer flasks containing 100 ml of culture medium (Supplementary Information) supplemented with either maltose (20 g/l), AVI (15 g/l), WS fragments (15 g/l), *P. halepensis* wood fragments (15 g/l) or *P. tremuloides* fragments (15 g/l). Each culture was done in triplicate. Inoculums of the liquid cultivations were prepared as described in Herpoël et al.^40^

### 2.6. Integration of transcriptome and exo-proteome profiles

LC-MS/MS analysis of the secreted proteins was performed as described in Navarro et al.^41^ Briefly, 10 µg of diafiltered proteins were loaded on SDS-PAGE gels and allowed to migrate on a 0.5 cm length. Each lane was cut into two slices for in-gel digestion according to a standard trypsinolysis protocol. On-line analysis of the peptides was performed with a Q-exactive mass spectrometer (Thermo Fisher Scientific), using a nanoelectrospray ion source. Protein identification was performed by querying MS/MS data against the genome of *P. cinnabarinus* BRFM 137, *P. coccineus* BRFM 310 or *P. sanguineus* BRFM 1264, together with an in-house contaminant database, using the *X*! Tandem Cyclone software. All peptides that matched with an *E* value lower than 0.05 were parsed with *X*! Tandem pipeline software. Proteins identified with at least two unique peptides and a log (*E* value) lower than −2.6 were validated.

For total RNA extractions, mycelia were ground in liquid nitrogen using a Freezer/Mill Cryogenic Grinder (SPEX Sample Prep, UK). Total RNA was extracted from 100 mg ground tissue in 1 ml TRIZOL (Ambion). Nucleic acids were precipitated with isopropanol, resuspended in water and treated with RNase-Free DNase I (QIAGEN). Total RNA was precipitated with LiCl and resuspended in DEPC-treated water. RNA quantity and quality were determined using the Experion RNA StdSens kit (QIAGEN). The transcriptome response of *P. sanguineus* to pine could not be analysed because of poor quality of the extracted RNAs. Double stranded cDNAs were synthesized from PolyA RNA and fragmented (200–300 bp) before construction of the sequencing libraries (Kapa Library Amplification Kit; Kapa Biosystems). Sequencing was done on the Illumina HighSeq-2500 JGI platform generating paired end reads of 150 bp each. Paired end 150 bp Illumina reads were trimmed for quality and aligned to the corresponding genome using TopHat 2 with only unique mapping allowed.^42^ Gene models for which the mean raw read counts were inferior to 5 were considered as not transcribed and their read counts were changed to 0.

The counts of mapped Illumina reads from biological triplicates of each growth condition (GEO accession number GSE82486) were normalized with the DESeq2 package and log2 transformed.^43^ The normalized read counts of genes coding for CAZymes, peptidases, hydrophobins and SSPs from *P. cinnabarinus* BRFM 137, *P. coccineus* BRFM 310 and *P. sanguineus* BRFM 1264 were retrieved and combined by conducting; (i) removal of batch effects with Combat function in SVA package^44^ and (ii) quantile normalization with the preprocessCore package.^45^ We used self-organizing map (SOM) to group genes into nodes according to similar transcript levels obtained from the different substrate conditions. Self-organising maps were constructed with the R package kohonen.^46^ The genes showing similar transcription levels were sorted and grouped into nodes of SOMs. It was empirically found that about 35 genes in a single node of the SOM gave the best resolution of the gene clusters. In terms of the standard formula ‘*X* × sqrt (*N*)’ to calculate the number of map units, where *N* was the number of the rows/genes of the data, *X* was 1.5. The number of iterations (epochs) was 100 times more than the map units to minimize the mean distance between the weights of the neighbouring nodes. The default initialization, learning rate and radius were used. Hexagonal SOM models were constructed. The mean reads (>12 log2) of the nodes (grouped genes) with the replicates combined were calculated for each substrate.

We integrated SOM with the protein secretome information analysed by peptide-LC-MS/MS, using SOM Harboring Informative Nodes with Gene Ontology.^10^^,^^47^^,^^48^

### 2.7. Transcription regulation of co-orthologous genes

One-to-one orthologous genes from *P. cinnabarinus* BRFM 137, *P. coccineus* BRFM 310 and *P. sanguineus* BRFM 1264 were retrieved using OrthoFinder v. 2.3.8.^49^ Heatmaps were created on the log2-fold change of transcript read counts in each growth condition when compared with growth on maltose after DESeq2 normalization using the ‘Heatmap’ function from the package ‘ComplexHeatmap’ v1.10.1 in R. Pairwise comparisons of gene expression based on Pearson correlation coefficients among all replicates was performed on CAZyme, peptidase, hydrophobin and SSP co-orthologs after read count normalization by DESeq2, batch effect removal and quantile normalization ‘cor’ function in R and visualized as heatmap with R package, ggplot2.

## 3. Results and discussion

### 3.1. Four *Pycnoporus* species share similar genomic features and CAZomes

To assess the genomic diversity in the genus *Pycnoporus*, the genome of *P. coccineus* BRFM 310 (herein named Pycco), *P. sanguineus* BRFM 1264 (Pycsa) and *P. puniceus* BRFM 1868 (Pycpun) were sequenced and compared with that of *P. cinnabarinus* BRFM 137 (herein named Pycci).^9^ The genome size of *P. coccineus*, *P. sanguineus* and *P. puniceus*, ranging from 30 to 36 Mb were in line with that of *P. cinnabarinus* (33.67 Mb) and WR relatives (Table 1; Supplementary Fig. S1 and Table S1). We observed low amounts of repeat sequences (1.8–12.3%) and the absence of major rearrangements in the genomes (Supplementary Figs S2 and S3). The genes coding for mating type, Class II peroxidases, CAZymes, peptidases, GSTs, hydrophobins, proteins from the secretory pathway, the glycosylation pathway, the carbon catabolism pathway and SSPs were inspected by expert annotation (Supplementary Tables S2–S15 and Figs S4–S13). We observed a high proportion of conserved protein-coding genes across the genomes (82.3% of the *P. cinnabarinus* protein-coding genes) and a low proportion of species-specific genes (4–5%; Supplementary Fig. S14). Inspection of mating type genes showed high sequence identity between the alleles of the four *Pycnoporus* species (Supplementary Table S4). In particular, *P. coccineus* BRFM 310 and *P. sanguineus* BRFM 1264 alleles were much more similar to each other than to that of the two other *Pycnoporus* species, in support of the notion that these two species are closely related.^15^^,^^50^

**Table 1:** Features of *P. coccineus* BRFM 310, *P. puniceus* BRFM 1868 and *P. sanguineus* BRFM 1264 genome assemblies and annotations

	*P. cinnabarinus*	*P. coccineus*	*P. puniceus*	*P. sanguineus*
Genome size (Mbp)	33.67	32.76	30.26	36.04
Number of contigs	2,036	469	105	2,046
Number of scaffolds	784	222	105	657
Scaffold N50	54	20	12	35
Scaffold L50 (Mbp)	0.17	0.47	0.79	0.32
Transposable element coverage (%)	8.15	1.8	12.33	4.91
Transposable element coverage (Mbp)	2.74	0.59	3.73	1.77
Number of predicted proteins	10,442	12,690	10,050	14,165
BUSCO complete protein sequences	1,268	1,321	1,309	1,293
BUSCO fragmented protein sequences	32	7	14	21
BUSCO missing protein sequences	35	7	12	21

The reliability of gene structural annotations was assessed using universal single-copy orthologs (BUSCO). The genome of *P. cinnabarinus* BRFM 137^9^ is indicated for comparison.

**Figure 1:**
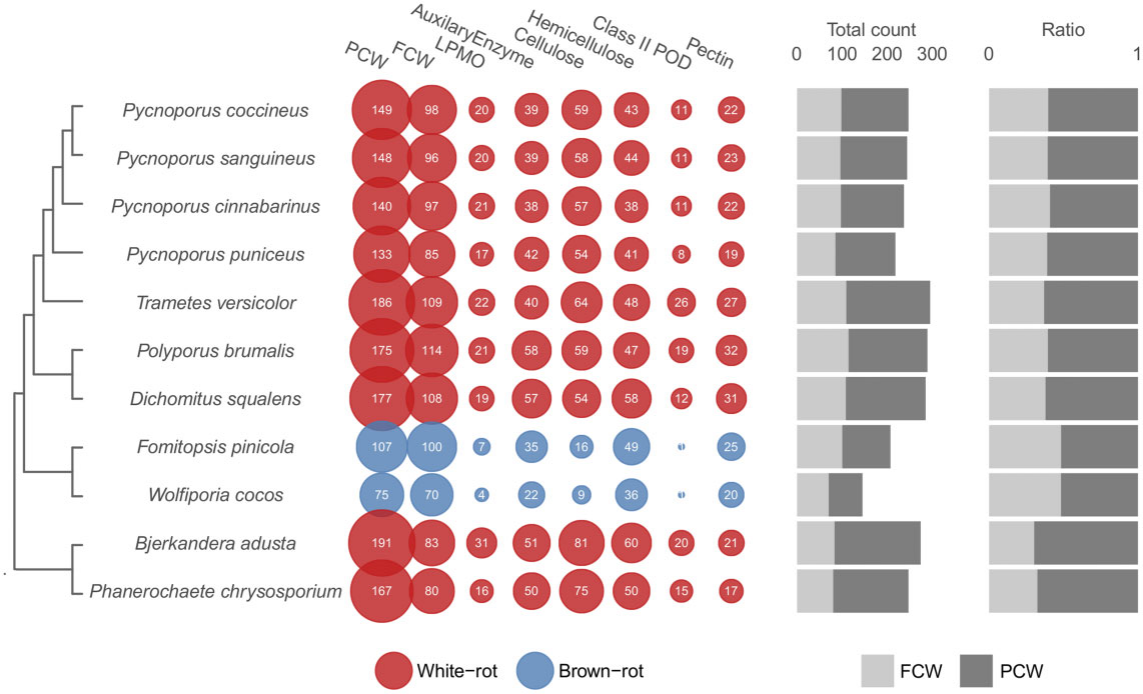
Gene counts for CAZyme domains of PCW and FCW-degrading enzymes. The bar plots show the total count of genes including PCW and FCW-degrading enzymes (left); and the ratio of PCW to FCW-degrading enzymes (right). The counts for AA enzymes that could contribute to PCW degradation include AA1_1 laccases and predicted secreted AA3, AA4 and AA5. The counts for PCW-active LPMOs include AA9, AA13, AA14 and AA16. Enzymes active on FCWs, cellulose, hemicellulose or pectin were classified according to Supplementary Figs S15–S18.

The CAZyme gene repertoires (CAZome) in the three newly sequenced genomes were similar to that of *P. cinnabarinus* (mean 436 CAZymes classified into 108 CAZy families). The gene counts for FCW-degrading enzymes were similar to that of other Polyporales fungi (Supplementary Fig. S15). As a common feature of WR fungi,^4^^,^^51^*Pycnoporus* genomes contained a large number of genes coding for PCW-active CAZymes when compared with brown-rot fungi, which use Fenton chemistry in combination with a limited number of CAZymes for PCW breakdown (Fig. 1, Supplementary Figs S16–S18). The genomes were rich in lytic polysaccharide monooxygenases (LPMOs) active on crystalline cellulose and β-(1,4)-linked hemicellulose polysaccharides (CAZy family AA9; 13–17 genes). The Class II PODs (in total 9–11 genes) were identified as manganese peroxidases (MnP), versatile peroxidases (VP) and lignin peroxidases (LiP; Supplementary Fig. S19 and Table S5). We observed a high number of predicted secreted oxidoreductases that could act as AA enzymes for the oxidative degradation of PCWs. Among them, GMC-oxidoreductases from CAZy subfamily AA3_2 (20–22 genes) have pivotal roles in PCW degradation. Secreted AA3_2 are involved in the generation of H_2_O_2_, a co-substrate for class II peroxidases and LPMOs. AA3_2 also contribute to the oxidation of saccharides and to the redox cycling of aromatic alcohols and quinones. In addition, we identified three AA5_1 glyoxal oxidase genes in each of the genomes, which encode copper radical oxidases involved in extracellular H_2_O_2_ production (Supplementary Figs S9–S13 and Table S6).

### 3.2. *Pycnoporus* species show diverse responses to lignocellulosic substrates

**Figure 2:**
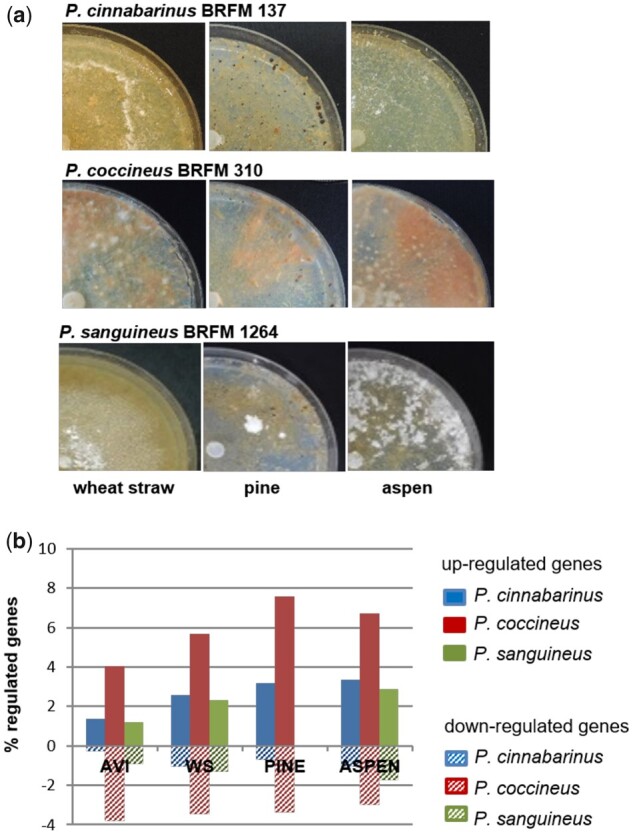
Phenotype polymorphism across three *Pycnoporus* species. (**a**) Agar plates after 6 weeks cultivation on ground WS, pine or Asp. *P. cinnabarinus* BRFM 137 did not develop mycelium on pine and Asp. The white dots formed by *P. sanguineus* on pine and Asp are arthrospores indicating that the fungus stopped growing to form dormant structures. (**b**) Percentage of regulated genes after 3-day growth in liquid cultures on AVI, WS, pine or Asp when compared with maltose (|fold change| ≥ 4) in the three *Pycnoporus* species. No RNASeq data was available for *P. sanguineus* grown on pine.

**Figure 3:**
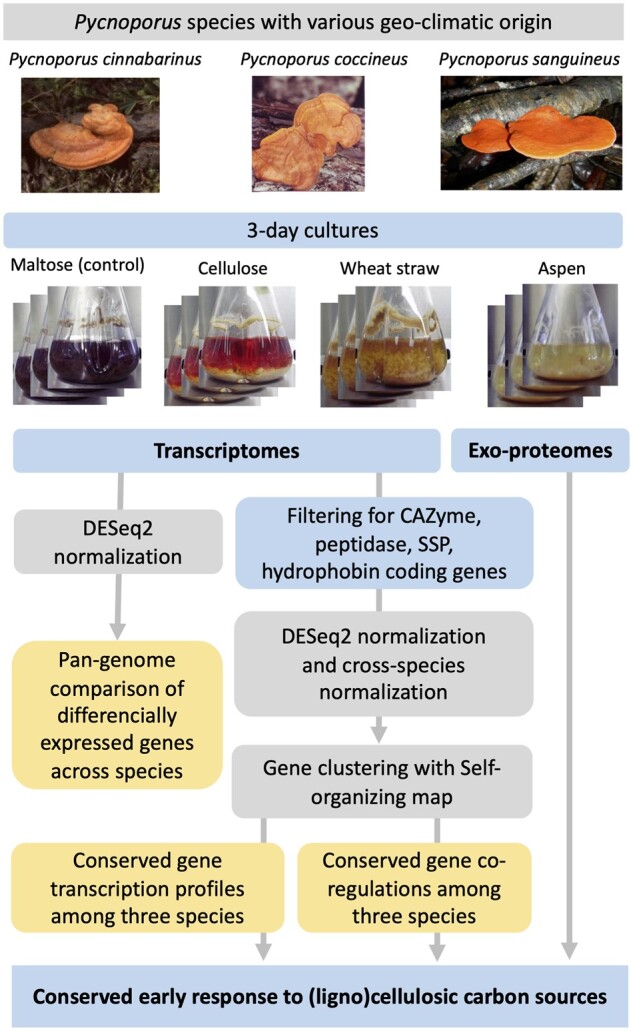
Cross-species comparison of the early response of three *Pycnoporus* species to lignocellulosic carbon sources.

To assess the functional diversity within the genus *Pycnoporus*, we compared the ability of the phylogenetically most closely related species; *P. cinnabarinus*, *P. coccineus* and *P. sanguineus* to grow on a variety of plant-derived carbon sources on agar plate assays. In these conditions, we observed differences in fungal growth on complex lignocellulosic substrates (Fig. 2a and Supplementary Fig. S20). We next analysed the early response of the three species to cellulose, WS and woody substrates in agitated liquid culture media and compared their transcriptomes and the secreted proteins collected from the media. Maltose was used as a control, as it was shown not to induce carbon catabolic repression in ascomycetes.^52^ AVI was used as a cellulose-enriched substrate, and WS, pine and Asp were used as representatives of Gramineae, softwood and hardwood, respectively. At day 3, most cultures had initiated growth and consumed all maltose initially present in the medium. This time-point was therefore selected to analyse the early response of each species to the substrates (Fig. 3; Supplementary Figs S21 and S22).

**Figure 4:**
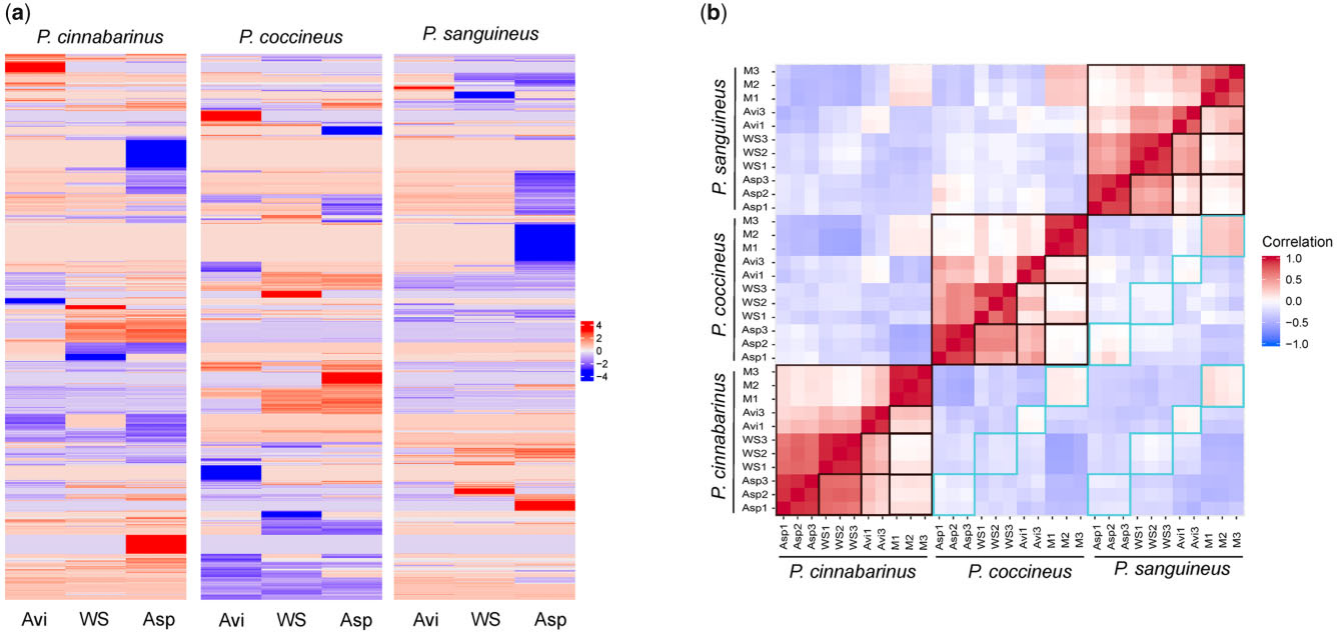
Global transcriptome similarity between co‐orthologous genes from *P. cinnabarinus*, *P. coccineus* and *P. sanguineus*. (**a**) Heatmap of changes in transcript read counts (log2 fold change) after 3-day growth on each carbon source when compared with maltose for 6,524 groups of 1-to-1 co‐ortholog genes. (**b**) Pearson correlation coefficient for the normalized transcript read counts in each growth condition for the 405 1-to-1 co‐ortholog CAZyme, peptidase, SSP and hydrophobin genes identified in the genomes. The comparisons of the response of each species to various substrates are highlighted in black boxes. Cross-species comparisons on a same substrate are highlighted in blue boxes. M: maltose; AVI: Avicel; WS: wheat straw; Asp: aspen.

The global transcriptome responses varied among the species. Especially, *P. coccineus* showed the highest proportion of regulated genes with up to 7.6% of the genes up-regulated on pine (fold change in transcript abundance ≥4; Fig. 2b). Similarity between the transcriptomes of the three species was assessed by analysing the differential expression of one‐to‐one orthologous genes (co‐orthologs) after 3-day growth on cellulose, WS or Asp when compared with maltose. To identify co‐orthologs, in silico-deduced proteomes of the three species were clustered into 13,836 orthogroups using OrthoFinder, of which 6,524 represented co‐orthologs. Similar transcript regulations were frequent between co-orthologs of two species. Surprisingly, however, we observed poor conservation of transcript regulation of co-orthologs across the three species, including for genes with high transcription induction or repression on particular carbon sources (Fig. 4a). We examined the transcript read counts of CAZyme-coding genes. Transcriptome profiles were more similar within the species cultured under the different conditions than between the species cultured on the same substrates (Supplementary Fig. S23). Also, we observed in each species that approximately half of the regulated CAZymes (41–51%) were up-regulated in response to cellulose, WS and Asp, highlighting the presence of core regulations to (ligno)cellulosic substrates with diverse compositions (Supplementary Fig. S24).

### 3.3. Conserved gene regulations in response to lignocellulosic substrates

In search for conserved enzymatic mechanisms involved in the initiation of lignocellulose breakdown, we analysed the gene sets sharing similar transcription profiles at the onset of the response to cellulose, WS or Asp. We focused on genes coding for proteins typically found in fungal exo-proteomes, i.e. CAZymes, peptidases, SSPs and hydrophobins.^53^ A total of 2,227 manually curated genes from the genomes of *P. cinnabarinus*, *P. coccineus* and *P. sanguineus* were analysed. In order to combine all RNA-seq data in a single cross-species analysis, transcript read counts were normalized using the DESeq2 package,^43^ the normalized read counts were log2 transformed and subjected to removal of batch effects and to quantile normalization. We checked the impact of each normalization step on the distribution of the data (Supplementary Figs S25–S28). Inspecting the one-to-one co-orthologs from this set of genes (405 orthology groups), we observed low conservation of the normalized transcript read counts across the species, except for growth on maltose and cellulose, and for *P. coccineus* and *P. sanguineus* grown on Asp, indicating that complex lignocellulosic substrates induced more diverse responses across the species (Fig. 4b).

**Figure 5:**
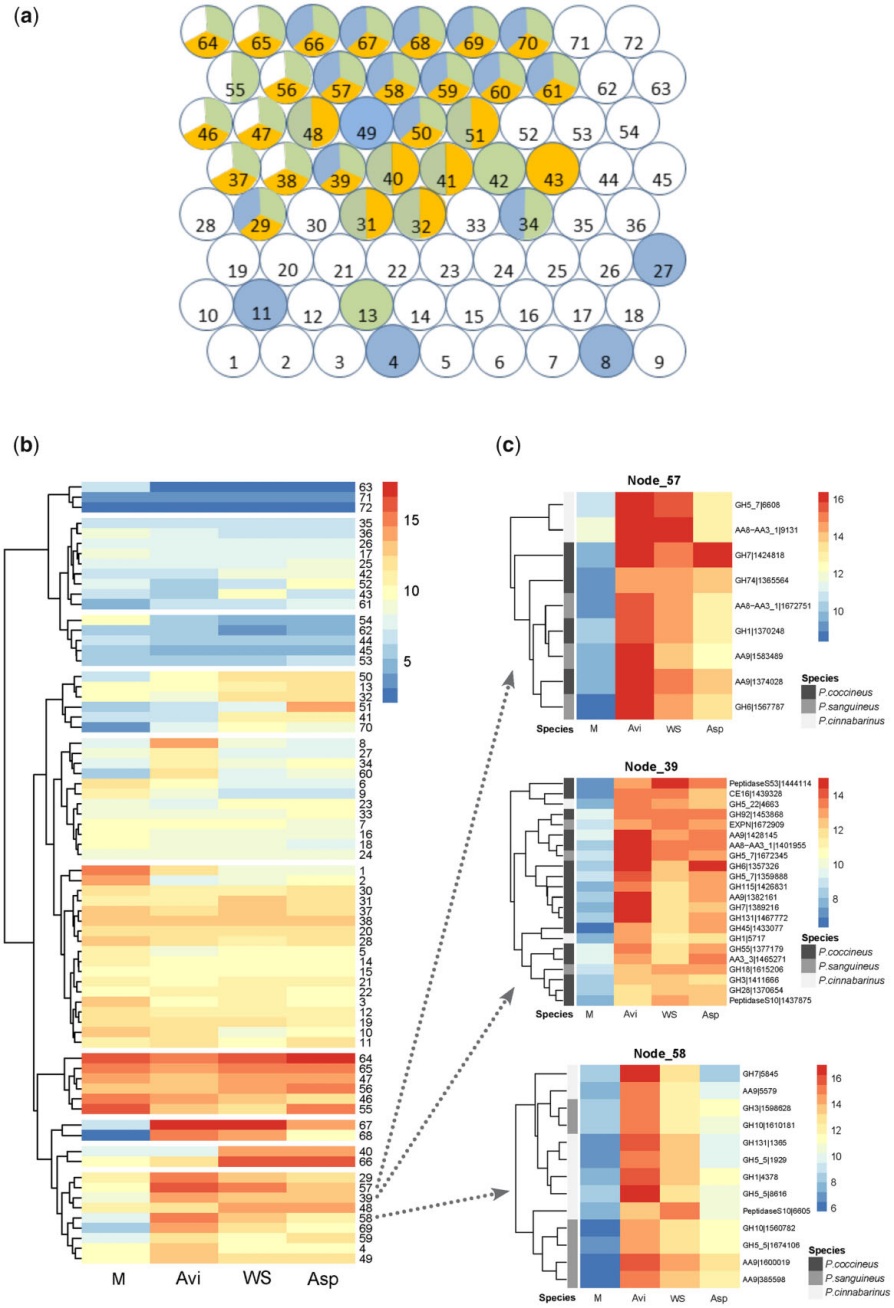
Clustering of genes coding for predicted CAZymes, peptidases, SSPs and hydrophobins in three *Pycnoporus* species according to their transcription profile on maltose (M), AVI, WS and Asp. (**a**) SOM clustering resulted in 72 nodes with average 31 genes per node. Nodes containing genes highly transcribed or up-regulated on cellulose (blue), Asp (green) or WS (orange) when compared with maltose are highlighted.(**b**) Hierarchical clustering of the nodes according to the averaged normalized transcript read counts on each carbon source. (**c**) Gene content and transcript profiles of nodes 57, 39 and 58. AA8-AA3-1: cellobiose dehydrogenase.

We grouped genes with similar transcript profiles into nodes (clusters of co-regulated genes) using the SOM unsupervised learning method. SOM is a data-driven clustering method constructing a topographic organization of nodes in which neighbouring nodes share similar transcriptome patterns, and thereby alleviates the requirement for arbitrary clustering thresholds. SOM allows the co-localization on the SOM map of genes with similar transcript levels. We used SOM on the normalized read counts from the three species in order to identify both the co-regulated genes within each species and the genes with similar transcript patterns across the species. We produced 72 nodes containing on average 31 genes per node (Fig. 5a and b). Exo-proteome data (Supplementary Tables S16–S18) were combined to the SOM map to integrate gene transcription and protein secretion information.

**Figure 6:**
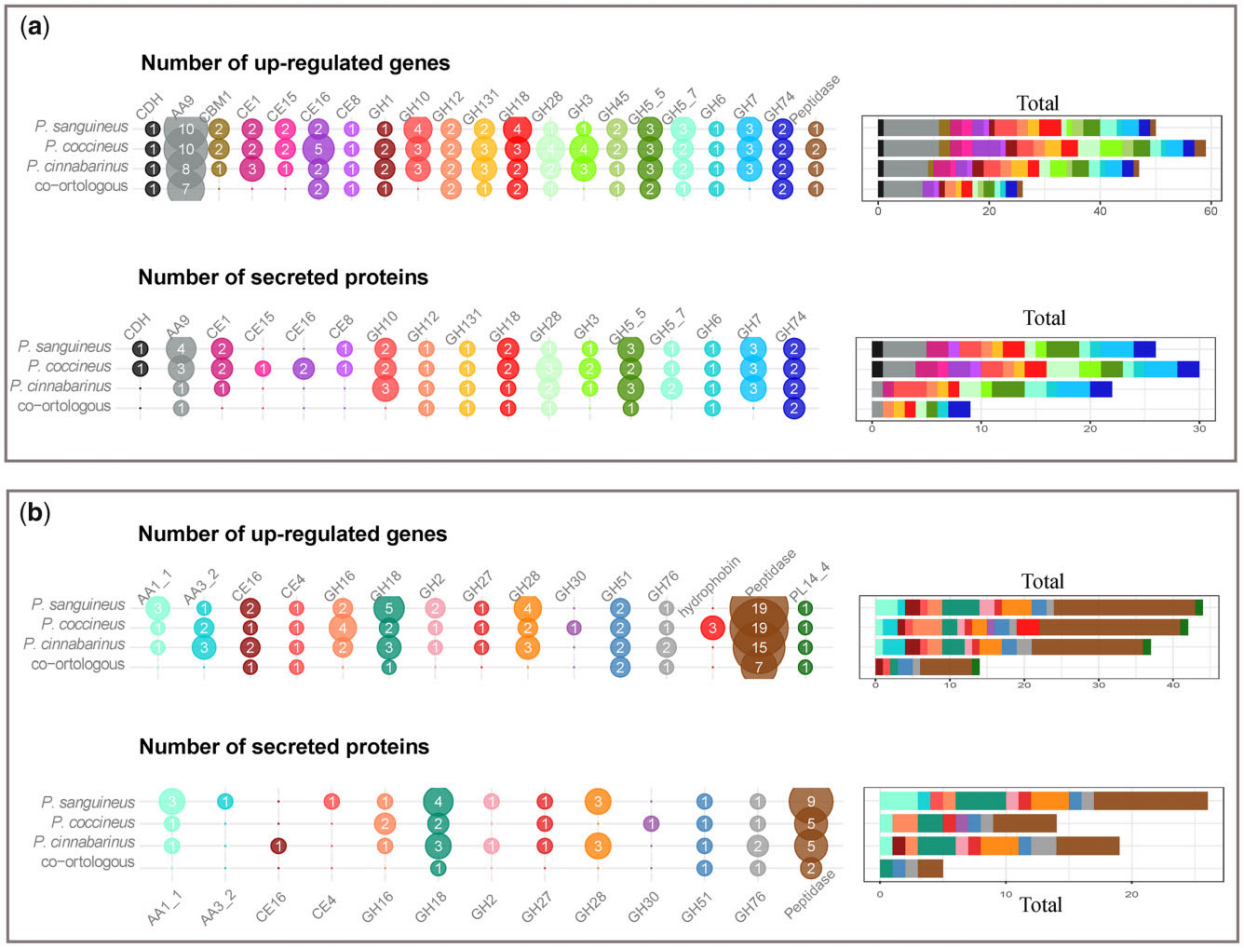
Shared expression regulation of CAZyme genes across the three *Pycnoporus* species. (**a**) Numbers of genes with shared differential expression on cellulose and numbers of proteins secreted during growth on cellulose. (**b**) Numbers of genes with shared specific differential expression on lignocellulosic substrates, not on cellulose. The numbers of orthologous groups of genes with conserved transcription regulation and secretion across the three species are indicated.

We first examined the transcriptional response to cellulose. We identified 19 nodes containing 250 genes highly transcribed (mean normalized log2 read count ≥12) or up-regulated (mean fold change ≥4 compared with maltose; Supplementary Table S19). Inspecting the gene content for these nodes (e.g. Fig. 5c), we looked for shared gene differential expression across the three species. In the three species, we found up-regulation for CAZyme-coding genes involved in β-(1,4)-glucan linkage breakdown including endoglucanases from family 5 subfamily 5 (GH5_5), GH45 and GH131, cellobiohydrolases (GH6, GH7), cellobiose dehydrogenases (CDH) and AA9 LPMOs (Fig. 6a). Also, there was a wide panel of enzymes active on hemicelluloses from families GH10, GH74, GH5_7, GH12, GH115, CE1, CE15, CE16, enzymes active on pectin CE8, GH28 and enzymes with promiscuous activities on glycosidic bounds GH1 and GH3. Among the 20 CAZyme families identified, 14 were represented by co-orthologous genes from the 3 species, showing conservation of their regulation in response to cellulose across the genus. Globally, 50% of the enzymes encoded by the up-regulated genes were detected in the culture medium (Fig. 6a), strengthening a role for these enzymes as key players in lignocellulose breakdown by *Pycnoporus* fungi.

To identify the genes specifically regulated for the complex lignocellulosic substrates, we selected genes up-regulated on WS or Asp, not on cellulose, when compared with maltose. There were 13 nodes including 225 genes that met at least one of the following criteria; (i) average normalized log2 read counts >12 on WS or Asp and <12 on maltose and cellulose, or (ii) mean fold change ≥4 on WS or Asp, and ≤4 on cellulose when compared with maltose. From these nodes, we identified the gene families with homologs among the three species. These included genes coding for enzymes that target hemicelluloses (CE16 and CE4 acetylesterases, GH2 β-mannosidases or β-glucuronidases, GH16 xyloglucan hydrolase, GH27 α-galactosidases, GH30 β-glucosidase/β-xylosidase, GH51 α-l-arabinofuranosidases and GH31 α-xylosidases/α-glucosidases), enzymes that target pectin (GH28 polygalacturonases, PL14_4 β-1,4-glucuronan lyases), AA1_1 laccases and AA3_2 GMC-oxidoreductases (Fig. 6b, Supplementary Table S20). Genes coding for glycoside hydrolases active on the FCW (GH18 chitinases, GH76 α-mannanases) and peptidases were also specifically up-regulated in the three species.

**Figure 7:**
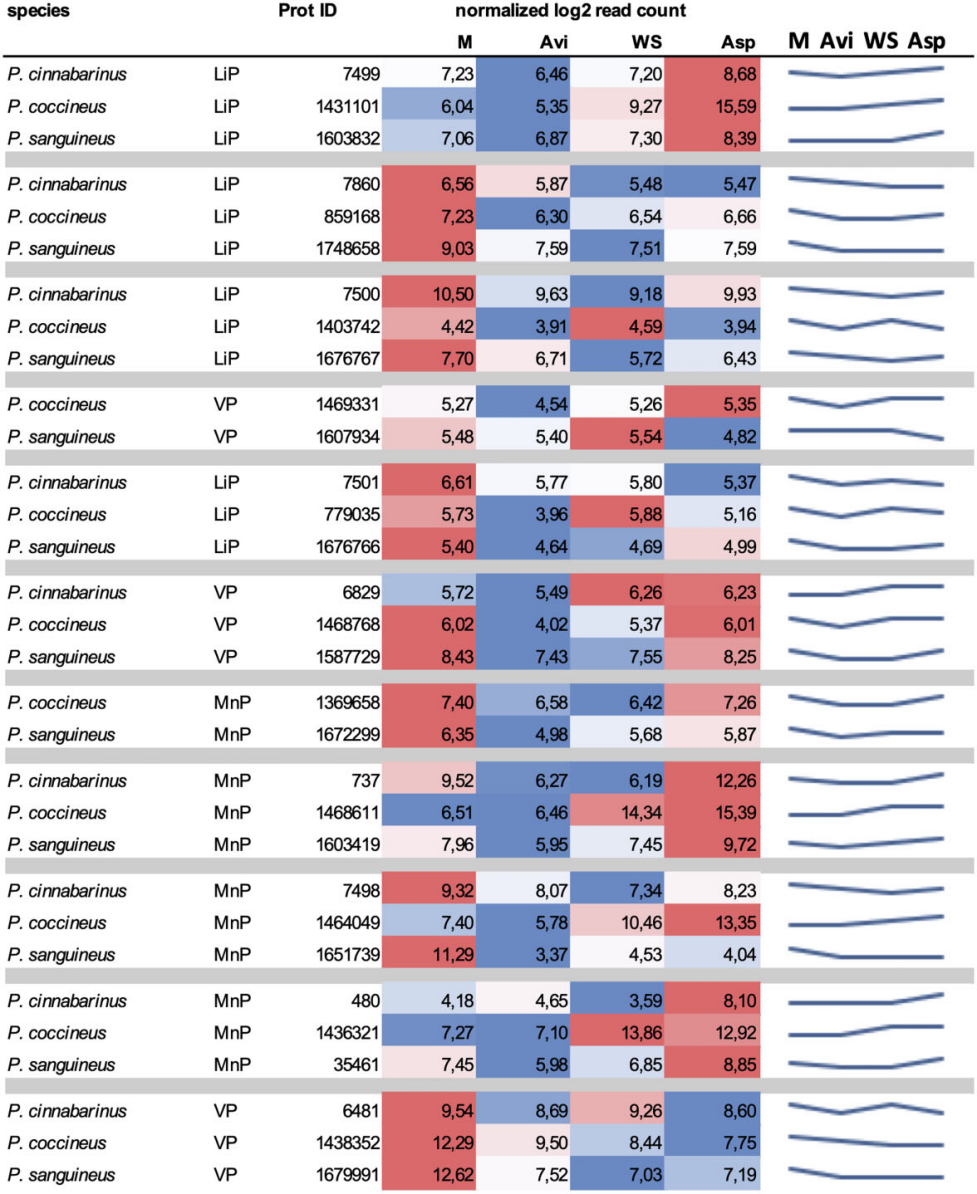
Regulation of Class II peroxidase genes in response to the substrates. Orthologous genes are grouped for comparison of their transcription profiles.

The genomes of the three species encoded the three types of class II peroxidases involved in lignin breakdown; MnP, LiP and VP (Supplementary Table S5). Orthologous MnP and LiP genes (defined by protein sequence phylogeny; Supplementary Fig. S6) did not show conserved regulation across the species in response to lignocellulosic substrates (Fig. 7). The highest induction factors were found in *P. coccineus* and a MnP (protein ID #1468611)- and a LiP (#1431101)-coding gene reached 800- and 1,500-fold induction on Asp, respectively. For the three species, we observed no up-regulation of the VP-coding genes.

### 3.4. Co-regulated genes indicative of enzymatic synergies

**Figure 8:**
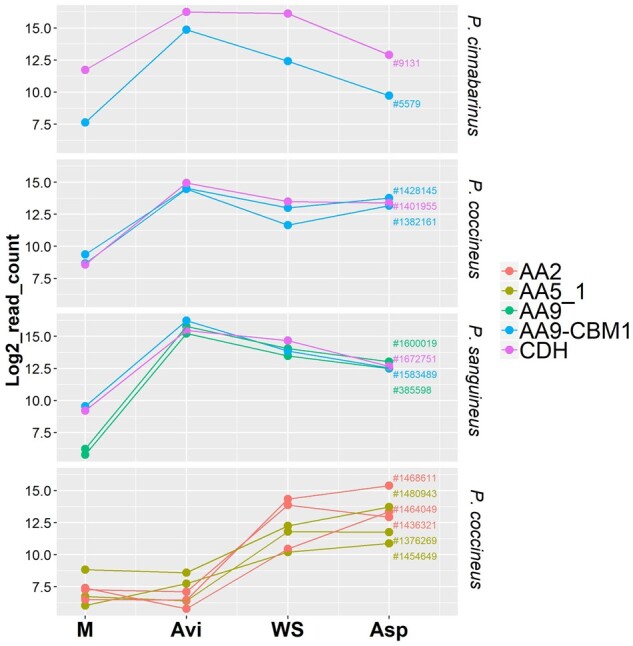
Conserved co-regulations of CDH and AA9 LPMO genes in *P. cinnabarinus*, *P. coccineus* and *P. sanguineus* and co-regulations of MnP- and GLOX-genes in *P. coccineus*. Transcript levels are expressed as log2-transformed read counts after 3-day-growth on maltose (M), AVI, WS or Asp. CBM: carbohydrate-binding module.

We investigated potential enzymatic synergies conserved in the three *Pycnoporus* species through co-regulated gene transcription with co-secreted corresponding proteins. In each species, one single CDH gene was present in the genome, which shared a similar transcription pattern with AA9 LPMO-coding genes (nodes 39, 57 and 58; Figs 5c and 8). These AA9 LPMO and CDH genes were up-regulated on cellulose and the corresponding proteins were identified in the exo-proteomes. AA9 LPMOs oxidatively cleave glycosidic chains at the cellulose surface thereby creating new substrate-binding sites for hydrolytic cellulases.^54^ CDHs are composed of a flavin adenine dinucleotide-binding dehydrogenase domain (AA3_1) connected via a flexible linker to a haem *b*-binding cytochrome domain^55^ (Cyt*b*; AA8). *In vitro*, CDHs behave as electron donors for AA9 LPMOs and boost LPMO activity (reviewed in Berrin *et al.*^56^). Our results suggest CDH as a biologically relevant electron donor for AA9 LPMOs.

As indicated above, genes coding for predicted class II peroxidases (AA2s) were specifically up-regulated on WS or Asp, but not on cellulose (Fig. 5b). Node 40, 41 and 32 contained three *mnp* genes from *P. coccineus* co-regulated with the three predicted glyoxal oxidase genes identified in this genome, suggesting that glyoxal oxidases might provide these MnPs with the H_2_O_2_ required for their activity (Fig. 6). In contrast in *P. cinnabarinus* and *P. sanguineus*, we did not observe any co-regulations at the transcript level between class II peroxidases and glyoxal oxidases.

### 3.5. Differentially regulated genes indicative of detoxification

In natural environments, lignocellulose degradation may lead to the release of toxic degradation products and extractives (e.g. phenols, terpenoids and tannins).^57^^,^^58^ We investigated if the regulation of genes involved in detoxification was conserved across the three *Pycnoporus* species in our growth conditions. For this purpose, we examined genes coding for putative GST in the cross-species SOM analysis.

We identified a GST gene from class GTT2 in *P. sanguineus* that was clustered in node 31 filtered for high transcript level on WS and Asp, and one GTT2 gene from *P. cinnabarinus* that was clustered in node 22 filtered for up-regulation on WS (Supplementary Table S20). One isoform of this class from *Phanerochaete chrysosporium* has been functionally characterized and has been shown to reduce high-molecular weight peroxides.^59^^,^^60^ In contrast, a total of four GST genes of *P. coccineus* were regulated on the tested substrates; two GST class A (FuA) genes were up-regulated on all the tested substrates (Supplementary Table S19) whereas one S-glutathionyl hydroquinone reductase and one GST Omega were specifically up-regulated in response to WS or Asp (Supplementary Table S20). For the case of *P. chrysosporium*, these classes of GSTs have potential roles in the detoxification of wood-derived molecules acting as ligandins for wood extractives or as catalysts for deglutathionylation of various substrates, including hydroquinone conjugates and terpenes.^36^^,^^59^^,^^61^ These findings suggest that different GST-related detoxification systems may exist in *P. coccineus*.

Cytochrome P450 (CYP450) monooxygenases are commonly involved in the first step of eliminating toxic molecules including molecules released from lignocellulose. CYP450 can initiate the modification of these molecules via hydroxylation, epoxidation or monooxygenation.^62^^,^^63^ The genomes of *P. cinnabarinus*, *P. coccineus* and *P. sanguineus* contain 107, 132 and 113 predicted CYP450, respectively. Inspection of the transcript profiles for these genes showed that *P. coccineus* had the highest number of up-regulated CYP450 genes in response to the tested substrates (17 genes) compared with *P. cinnabarinus* and *P. sanguineus* (14 and 6 up-regulated genes, respectively). Some CYP450 genes were commonly up-regulated in at least two species, which were from families CYP63, CYP5035, CYP5139, CYP5144 and CYP5150 According to the Fungal cytochrome P450 database^64^ (Supplementary Table S13). Of these, CYP63, CYP5139 and CYP5144 were shown to be active on multiple xenobiotic compounds such as polycyclic aromatic hydrocarbons, alkylphenols and alkanes.^63^^,^^65^^,^^66^ CYP5035s oxidize plant chemicals (i.e. resin and flavonoids), and the number of genes for CYP5035 and CYP5150 expanded in basidiomycetes that grow on wood or litter.^63^ Our results suggest that these CYP450 families could be differently utilized by the species for detoxification of molecules released from the lignocellulosic substrates.

## 4. Discussion

### 4.1. Phenotype diversity outreaches diversity of the predicted proteome across the genus *Pycnoporus*

The four *Pycnoporus* species studied here are found in different geo-climatic areas^15^^,^^16^ covering the Northern hemisphere (*P. cinnabarinus*), countries bordering the Indian and Pacific Oceans (*P. coccineus*), the tropics and subtropics of both hemispheres (*P. sanguineus*) and paleotropical areas (*P. puniceus*), with overlap limited to Japan and Eastern China. Accordingly, the parental strains of the monokaryons studied here were collected in Europe, China, South America and Indonesia, respectively. Here, we show that the genomes sequenced from geographically distant strains did not show any major rearrangements in structure and protein-coding gene composition. Our findings suggest that geographical isolation of the species the genus *Pycnoporus* has not yet lead to any noticeable genome level diversity. In addition, the high sequence identity between mating type genes of the four species, particularly between *P. coccineus* and *P. sanguineus* suggests that the speciation events are recent in this genus. In the genus *Laetiporus*, another group of wood-decay fungi from the order Polyporales that originated about 20 mya,^67^ several regional speciation events have been estimated between 9.88 and 2.89 mya. Some of these speciation events have been related to vicariance that emerged due to tectonic activity and the disconnection of the continental plates or from wind and ocean current dispersion of basidiospores.^67^ It is currently estimated that the genus *Pycnoporus* originated about 40–50 mya (Nagy LG, personal communication). Further molecular studies are needed to understand the dispersal routes and the role of regional speciation in the evolutionary history of *Pycnoporus*.

Despite conserved CAZomes, the *P. cinnabarinus*, *P. coccineus* and *P. sanguineus* strains analysed here showed different abilities to grow on (ligno)cellulosic substrates. This finding demonstrates that gene repertoires obtained from genome sequencing are not sufficient to predict the ability of a strain to grow on recalcitrant raw biomass. The comparison of the early responses of the fungi to the substrates showed that gene transcription profiles better correlated with the strains rather than with the substrates. A similar trend was observed in the WR fungus *Pleurotus ostreatus*, between monokaryotic strains issued from a same parental dikaryotic strain. In *P. ostreatus*, these differences in gene regulation have been partly attributed to the presence of transposable elements near the differentially regulated genes.^68^ A closer analysis of transposable element localization or methylation status of each monokaryon haplotype would help understanding the transcription regulation we observed.

### 4.2. Conserved sets of enzymes produced at the onset of wood-decay activity

As a conserved response to cellulose across three *Pycnoporus* species, we identified here a set of CAZyme genes with shared transcriptional regulations. For a sub-set of these genes, the corresponding enzymes were commonly found in the secretomes of the three species at the analysed time point, further strengthening a role for these enzymes as key players in lignocellulose breakdown by these fungi. These included genes coding for enzymes active on β-1,4-glucan linkages (GH5_5 and GH131 endo-β-1,4-glucanases, GH6 and GH7 cellobiohydrolases and AA9 LPMOs) and genes targeting the hemicellulose backbone (GH10 endoxylanases, GH12 and GH74 xyloglucan hydrolases, GH5_7 β-mannosidases) or branched groups (CE1 feruloyl esterases) and enzymes active on pectin (GH28 polygalacturonases). In the presence of lignocellulosic substrates, additional genes were commonly induced and the corresponding CAZymes secreted, that target hemicelluloses (GH16 xyloglucan hydrolase, GH27 β-galactosidases, GH30 β-glucosidases/β-xylosidases, GH51 α-l-arabinofuranosidases), peptidases and laccases (AA1_1).

### 4.3. AA9 LPMOs and their enzymatic partners

AA9 LPMOs are known as key players in oxidative cellulose depolymerization by wood-decay fungi.^56^ Expansions in AA9 gene numbers is a common feature of the genomes of WR fungi,^69^ but whether members of the AA9 gene family play different roles during wood decay has not been elucidated. We identified 17, 16 and 11 genes coding for AA9 LPMOs in *P. cinnabarinus*, *P. coccineus* and *P. sanguineus*, from which 11 (64%), 13 (81%) and 10 (90%), respectively, were up-regulated during the early response to cellulose-containing substrates. In each strain, several AA9 LPMO genes shared similar transcription profiles, and several AA9 LPMOs were concomitantly secreted, indicating that several AA9 LPMOs co-secreted by the fungi might act simultaneously on the substrate.

The identification of enzymatic partners for LPMOs is a very active field of research.^70–73^ Looking at conserved co-regulations across the three strains, we identified CDHs as invariably co-regulated with at least one AA9 LPMO gene. CDHs have been shown to reduce O_2_ and generate H_2_O_2_ which in turn can fuel, via the Fenton reaction, the production of hydroxyl radicals that disrupt lignocellulose polymers.^74^ More recently, CDHs have been shown to activate AA9 LPMOs *in vitro* through electron transfer^75^ and it has been proposed that CDHs could also activate AA9 LPMOs through the generation of H_2_O_2_, a co-substrate for the enzyme.^76^ Our findings support the hypothesis of synergy between AA9 LPMOs and CDHs in vivo as a conserved mechanism for cellulose degradation in the genus *Pycnoporus* spp.

### 4.4. Co-secretion of extracellular H_2_O_2_-generating and -consuming enzymes

Extracellular H_2_O_2_ is a central factor in oxidative lignocellulose breakdown. It was proposed that WR fungi could avoid oxidative damages due to H_2_O_2_ accumulation in the vicinity of the hyphae by the co-secretion of H_2_O_2_-generating and H_2_O_2_-consuming enzymatic partners.^72^ Some secreted GMC-oxidoreductases such as glucose dehydrogenases and aryl-alcohol quinone oxidoreductases (AA3_2), which generate H_2_O_2_, are able to prime AA9 LPMO activity *in vitro*.^70^^,^^73^ Similarly, glyoxal oxidases can generate H_2_O_2_ and prime MnP and LiP activity *in vitro*.^77^ A biological relevance for these synergetic activities was previously suggested from the co-occurrence in fungal secretomes of class II peroxidases with GMC-oxidoreductases^10^^,^^51^^,^^78–82^ or glyoxal oxidases.^10^^,^^51^^,^^79^^,^^81^^,^^83–85^ Here, we confirmed the co-occurrence in the secretomes of GMC-oxidoreductases and glyoxal oxidases with class II peroxidases and AA9 LPMOs as a common feature of three *Pycnoporus* species. However, we detected no evidence for co-regulation of the corresponding genes at the transcript level, except between three MnPs and two glyoxal oxidase encoding genes in *P. coccineus*. We hypothesize that independent tight regulations of gene expression for H_2_O_2_-generating and H_2_O_2_-consuming enzymes at the transcription level could allow a more flexible system to rapidly adapt extracellular reactive oxygen species concentrations and avoid oxidative damage to the hyphae.

## 5. Conclusion

Although WR fungi are well-studied for the diversity of enzymatic systems for lignocellulose breakdown, their intra-genus diversity has not been extensively explored so far. Here, we investigated the functional diversity within the genus *Pycnoporus*, a group of fungi from the Trametes clade renowned for the ecological significance and a great potential for novel biocatalysts. We introduced a new method for cross-species comparisons of transcriptomes, using the genome sequences of the four species described in the genus and expert gene functional annotations. The transcriptomic aspect of observations was validated with experimentally identified secreted proteins, supporting our hypotheses. We identified a conserved set of genes responsive to lignocellulosic substrates that outlines key enzymatic mechanics for wood decomposition activity in these fungi. We observed that the mechanisms involved in oxidative lignin breakdown were more diverse than those in polysaccharide breakdown and the fungal enzymes detoxifying lignocellulose-released products were different among the species.

## Supplementary Material

dsaa011_Supplementary_Data
