# Supplementary material for: Conserved white-rot enzymatic mechanism for wood decay in the Basidiomycota genus Pycnoporus
Source: DNA Res. 2020 Jun 12;27(2):dsaa011. doi: 10.1093/dnares/dsaa011 (PMC7406137; doi:10.1093/dnares/dsaa011)
Supplement: dsaa011_Supplementary_Data [file dsaa011_supplementary_data.pdf]

Supporting Information

Article title: **Conserved white rot enzymatic mechanism for wood decay in the *Basidiomycota* genus *Pycnoporus*.**

Authors: Shingo Miyauchi, Hayat Hage, Elodie Drula, Laurence Lesage-Meessen, Jean-Guy Berrin, David Navarro, Anne Favel, Delphine Chaduli, Sacha Grisel, Mireille Haon, François Piumi, Anthony Levasseur, Anne Lomascolo, Steven Ahrendt, Kerrie Barry, Kurt M. LaButti, Didier Chevret, Chris Daum, Jérôme Mariette, Christophe Klopp, Daniel Cullen, Ronald P. de Vries, Allen C. Gathman, Matthieu Hainaut, Bernard Henrissat, Kristiina S. Hildén, Ursula Kües, Walt Lilly, Anna Lipzen, Miia R. Mäkelä, Angel T. Martinez, Mélanie Morel-Rouhier, Emmanuelle Morin, Jasmyn Pangilinan, Arthur F.J. Ram, Han A.B. Wösten, Francisco J. Ruiz-Dueñas, Robert Riley, Eric Record, Igor V. Grigoriev, Marie-Noëlle Rosso

1. List of supplementary tables 1
2. List of supplementary figures 3
3. Production of monokaryons 5
4. Fungal growth comparisons on different carbon sources 5
5. The mating type loci of *Pycnoporus* species 5
6. Peroxidase gene models in *Pycnoporus* genomes 8
7. Copper Radical Oxidases 11
8. Laccases 11
9. (GMC)-oxidoreductases 11
10. Small Secreted Proteins 11
11. Secretory pathway 12
12. Glutathione-S Transferases 12
13. P450s 13
14. Peptidases 13
15. References 14

1. List of supplementary tables

Table S1. Genome features for *Cylindrobasidium torrendii* (Cylto1), *Fistulina hepatica* (Fishe1), *Dichomitus squalens* (Dicsq1), *Fomitopsis pinicola* (Fompi3), *Polyporus brumalis* (Polbr1), *Pycnoporus cinnabarinus* (Pycci1), *Pycnoporus coccineus* (Pycco1), *Pycnoporus*

puniceus (Pycpun1), *Pycnoporus sanguineus* (Pycsa1), *Trametes versicolor* (Trave1) and *Wolfiporia cocos* (Wolco1).

Table S2. *A* and *B* mating type loci and related genes as manually annotated in the JGI databases.

Table S3. Numbers of predicted *B* mating type genes and similar non-mating-type genes found in the genomes of the four *Pycnoporus* strains.

Table S4. Identities and similarities between orthologous proteins linked to mating type or mating-type-like genes in *Pycnoporus* species.

Table S5. JGI protIDs for the 60 protein models of class I peroxidase (CCP), class II peroxidase (short MnP, LiP, and typical and atypical VP) and HTP peroxidase genes in the genomes of three *Pycnoporus* species.

Table S6. JGI protIDs for the copper radical oxidase gene models in the genomes of three *Pycnoporus* species.

Table S7. Genes coding for laccases, multicopper oxidases and ferroxidases in the genomes of *P. cinnabarinus* BRFM 137, *P. coccineus* BRFM 310 and *P. sanguineus* BRFM 1264.

Table S8. AA3 gene transcription regulation and protein detection in the secretomes of *Pycnoporus coccineus* BRFM310.

Table S9. *P. cinnabarinus* BRFM 137 genome annotation related to protein secretion pathways.

Table S10. *P. cinnabarinus* BRFM 137 genome annotation related to protein glycosylation pathways.

Table S11. Propagation of functional annotations for predicted proteins involved in the secretory pathway, from *P. cinnabarinus* BRFM 137 to *P. coccineus* BRFM 310 and *P. sanguineus* BRFM 1264.

Table S12. Propagation of functional annotations from P. cinnabarinus BRFM 137 to P. coccineus BRFM 310 and P. sanguineus BRFM 1264 predicted proteins involved in the glycosylation pathway.

Table S13. Number of predicted Cyt450 genes up-regulated after 3 day growth on Avicel, wheat straw, pine or aspen.

Table S14. Gene count for predicted peptidases in the genomes of P. cinnabarinus BRFM 137, P. coccineus BRFM 310 and P. sanguineus BRFM 1264.

Table S15. Numbers of peptidases identified in at least one secretome obtained from *P. cinnabarinus* BRFM 137, *P. coccineus* BRFM 310 and *P. sanguineus* BRFM 1264 grown on maltose, Avicel, wheat straw, aspen or pine.

Table S16. List of proteins detected in the secretomes of *P. cinnabarinus* BRFM 137 grown on Aspen, Pine, wheat straw, Avicel or maltose.

Table S17. List of proteins detected in the secretomes of *P. coccineus* BRFM 310 grown on Aspen, Pine, wheat straw, Avicel or maltose.

Table S18. List of proteins detected in the secretomes of *P. sanguineus* BRFM 1264 grown on Aspen , Pine, wheat straw, Avicel or maltose.

Table S19. List of the groups of genes (nodes) from *P. cinnabarinus* BRFM 137, *P. coccineus* BRFM 310 and *P. sanguineus* BRFM 1264 up-regulated in response to Avicel, and their transcription profile on wheat straw and aspen.

Table S20. List of the groups of genes (nodes) from *P. cinnabarinus* BRFM 137, *P. coccineus* BRFM 310 and *P. sanguineus* BRFM 1264 up-regulated in response to wheat straw or aspen, not Avicel.

2. List of supplementary figures

Fig. S1. Genomic features of 11 fungi.

Fig. S2. Transposable elements present in 11 polyporales genomes.

Fig S3. Dot plot representation of the pair-wise alignments of Pycci, Pycco, Pycsa and Pycpun scaffolds.

Fig. S4. Homology models for the molecular structures of class I and II heme peroxidases from the *P. coccineus* BRFM 310 genome.

Fig. S5. Homology models for the molecular structures of heme-thiolate peroxidases (HTPs) from the *P. coccineus* BRFM 310 genome..

Fig. S6. Dendrogram of sixty deduced heme peroxidase sequences (and one lip pseudogene) from three *Pycnoporus* species.

Fig. S7. Dendrogram showing evolutionary relationships among 310 basidiomycete Class I and Class II heme peroxidases.

Fig. S8. Dendrogram focused on HTP peroxidases (a total of 179) showing evolutionary relationships

Fig. S9. Relationships between predicted CRO proteins of 15 Basidiomycete species.

Fig. S10. Phylogenetic analysis of AA3 protein sequences from *P. coccineus* BRFM 310 and 46 fungal AA3s with characterized activities.

Fig. S11. Phylogenetic analysis of AA3 protein sequences from *P. cinnabarinus* BRFM 137 and 46 fungal AA3s with characterized activities.

Fig. S12. Phylogenetic analysis of AA3 protein sequences from *P. sanguineus* BRFM 1264 and 46 fungal AA3s with characterized activities.

Fig. S13. Phylogenetic analysis of AA3 protein sequences from *P. puniceus* BRFM 1868 and 46 fungal AA3s with characterized activities.

Fig. S14. Conservation of predicted protein coding genes between the four *Pycnoporus* genomes and analysis of the strain-specific protein coding genes.

Fig. S15. Gene counts for CAZyme domains active on fungal cell walls in Polyporales genomes.

Fig. S16. Gene counts for CAZyme domains of CAZymes active on cellulose and β-1,4-glucans in Polyporales genomes.

Fig. S17. Gene counts for CAZyme domains of CAZymes active on hemicellulose in Polyporales genomes.

Fig. S18. Gene counts for CAZyme domains of CAZymes active on pectin in Polyporales genomes.

Fig. S19. Gene counts for CAZyme domains of Auxiliary Activity enzymes.

Fig. S20. Growth of *P. cinnabarinus* BRFM 137, *P. coccineus* BRFM 310 and *P. sanguineus* BRFM 1264 on 17 different carbon sources.

Fig. S21. Growth of *P. cinnabarinus* BRFM 137, *P. coccineus* BRFM 310 and *P. sanguineus* BRFM 1264 on six different carbon sources in liquid cultures as determined by qPCR.

Fig. S22. Maltose concentration in the culture medium during growth of *Pycnoporus coccineus* BRFM310 on maltose, Avicel, xylans and galactomannans, wheat straw, pine, or aspen.

Fig. S23. Comparison of the transcript levels for CAZyme coding genes between the three strains grown on four different substrates by Principal Component Analysis.

Fig S24. Numbers of CAZyme coding genes differentially regulated on cellulose (Avicel), wheat straw, aspen or pine, in *Pycnoporus cinnabarinus* BRFM 137 (a), *Pycnoporus coccineus* BRFM 310 (b), and *Pycnoporus sanguineus* BRFM 1264 (c).

Fig. S25. Distribution of log2 transformed transcript count per gene after normalization with DESeq2 (a), and after additional quantile normalization (b), batch effect removal (c), or both quantile normalization and batch effect removal (d).

Fig. S26. The first and second principal components from principal component analysis (PCA) using the DESeq2-normalized log2-transformed transcript count per gene (a), and after additional quantile normalization (b), batch effect removal (c), or both quantile normalization and batch effect removal (d).

Fig. S27. The first and second principal components from Single Value Decomposition (SVD) with the DESeq2-normalized log2-transformed transcript count per (a), and after additional quantile normalization (b), batch effect removal (c), or both quantile normalization and the batch effect removal (d).

Fig. S28. Distribution of the first principal component from Single Value Decomposition (SVD) with the DESeq2-normalized log2-transformed transcript count per gene (a), and after additional quantile normalization (b), the removal of batch effect (c), or both quantile normalization and the batch effect removal (d).

3. Production of monokaryons

P. coccineus BRFM 310 was obtained from BRFM 66 (IMB W006-2, ITS accession number AF363754) collected on rotten wood of broad-leaves tree in Guangxi, Mt Daming, China. *P. sanguineus* BRFM 1264 was obtained from BRFM 902 (GUY 110, ITS accession number FJ234193) collected on burnt wood in Macouria, French Guyana. Strains BRFM 66 and BRFM 902 were, respectively, classified among the *P. coccineus*-like group and the sub-clade of *P. sanguineus sensu stricto* in a phylogenetic analysis of the *Pycnoporus* genus (Lesage-Meessen *et al.*, 2011). The strain BRFM 902 was subsequently designated as reference strain for *P. sanguineus* (L) Murrill, Surinam (Lamark, 1783). *P. puniceus* BRFM 1868 was obtained from BRFM 1856 collected on a log in Lombok island, Indonesia.

4. Fungal growth comparisons on different carbon sources

Fungal growth comparisons on agar plates were done on 17 different carbon sources. For this purpose, one fungal disk was deposited in the center of Petri dishes containing minimal medium and 10 g/l simple carbohydrates or 30 g/l complex carbohydrates. The minimal medium contained the following (per liter): 6.0 g of $NaNO_3$, 1.5 g of KH_2PO_4, 0.5 g of KCl, 0.5 g of $MgSO_4$, 20 g Agar, 200 µl of trace elements (10 g/l EDTA, 4.4 g/l $ZnSO_4 \cdot 7H_2O$, 1.01 g/l $MnCl_2 \cdot 4H_2O$, 0.32 g/l $CoCl_2 \cdot 6H_2O$, 0.315 g/l $CuSO_4 \cdot 5H_2O$, 0.22 g/l $(NH_4)_6Mo_7O_{24} \cdot 4H_2O$, 1.47 g/l $CaCl_2 \cdot 2H_2O$, and 1.0 g/l $FeSO_4 \cdot 7H_2O$). Guar Gum, Arabic gum and beechwood xylan, Oat spelt xylan, Apple pectin, Citrus pectin, Lignin, Inulin, Starch were from Sigma (St. Louis, Mo.). Wheat bran, Sugar Beet Pulp, Citrus Pulp, Soybean hulls, Rice bran, Cotton Seed Pulp and Alfalfa Meal were from CBS-KNAW, Utrecht University, The Netherlands. Inoculated plates were kept at 30°C for 7 days (Fig. S5). Fungal growth in liquid cultures was determined by quantitative PCR (qPCR) as described in Zhou et al., 2015. Briefly, genomic DNA was extracted from ground mycelia using the NucleoSpin® Plant II kit (Macherey-Nagel, Germany). qPCR was performed using qPCR Master Mix for SYBR Plus (Bio-Rad, France) on a C1000 Touch Thermal Cycler CFX96 real-time system (Bio-Rad). The oligonucleotide primers were designed to amplify a 150-bp fragment in the 5.8S conserved sequence: 5.8S-F (5'-TTTCAGCAACGGATCTCTTGGC-3'), 5.8S-R (5'-CAAACAGGCATGCTCCTCGG-3'). Standard curves were established for each strain using serial dilutions of genomic DNA ranging from 1 ng/µl down to 1.10 − 4 ng/µl. The PCR cycle was as follows: 30 sec at 95°C, and then 5 sec at 95°C, 5 sec at 65°C for 39 cycles, followed by a melt curve step (65 to 95°C, with 0.5°C steps). The mycelium dry weight was deduced from genomic DNA quantification using conversion factors pre-established for each strain.

5. The mating type loci of *Pycnoporus* species

Pycnoporus species are heterothallic tetrapolar Agaricomycetes (Nobles & Frew, 1962; Lomascolo *et al.*, 2011) which means that their life cycle is controlled by two distinct mating type loci called *A* and *B*. The *A* locus of Agaricomycetes encodes two distinct homeodomain transcription factors classified as HD1 and HD2 by the presence of a TALE-type and a classical homeodomain, respectively (Kües, 2015). The *A* loci of most Agaricomycetes are flanked on one side by a conserved gene *mip* for a mitochondrial metalloendopeptidase (James *et al.*, 2004, 2013) and on the other side by a conserved gene of unknown function which is referred to as *β-fg* because of its flanking position relative to the mating type *Aβ* sublocus of *Coprinopsis cinerea*

(Kües *et al.*, 1992; James *et al.*, 2013). We therefore identified in the genomes of all *Pycnoporus* strains their locations by taking advantage of sequence conservations. Accordingly, *P. cinnabarinus* BRFM 137, *P. coccineus* BRFM 310 and *P. sanguineus* BRFM 1264 possess an *A* mating type locus of about 4.2-4.7 kb in length, with a single divergently transcribed HD1-HD2 gene pair. As typical for the Polyporales (James *et al.*, 2013; Levasseur *et al.*, 2014; Kues *et al.*, 2015), the orientation of the HD1-HD2 gene pair is transversed relative to genes *mip1* and *β-fg* as compared to the Agaricales and other clades of Agaricomycetes (Kües *et al.*, 2011). Further interesting is *P. puniceus* BRMF 1868. It has a complete HD1-HD2 gene pair linked with a functional gene *mip1* (on scaffold 7) while a DNA segment with these three genes (at least 14837 bp; 99 % identity to the scaffold 7 sequence) had been duplicated (found as scaffold 78) and diverged so that the copies of all three genes became likely non-functional. Further, gene *β-fg* has been replaced from its original position and is found in the genome in single copy inserted into the >750 kb-long scaffold 16.

Predicted protein sequences were collected for the *mip1* genes, *β-fg* genes and *HD1* and *HD2* genes for all four *Pycnoporus* strains (Table S2). As reported before for *P. cinnabarinus* BRFM 137 (Levasseur *et al.*, 2014), *Pycnoporus* HD1 proteins by sequence degeneration lack a recognizable HD1-protein typical TALE homeodomain but they have retained a mating N domain and a mating C domain defined by similarities to N-terminal and C-terminal sequence regions of HD1 mating type proteins encoded by *Coprinopsis cinerea b1* alleles (Tymon *et al.*, 1992). HD2 proteins in contrast have a classical 60 aa-long homeodomain with a WFQNR DNA-binding motif in the third helix.

The *B* mating type loci of Agaricomycetes contain several pheromone and pheromone receptor genes grouped together in a larger DNA sequence region (Brown & Casselton, 2001; Kües, 2015). In addition, there can be genes for non-mating type pheromone receptors and genes for pheromone-like short peptides (Kües *et al.*, 2011; Kües, 2015). Previously, up to four genes for G protein-coupled 7-transmembrane pheromone receptors (Ste3 proteins) and seven genes for potential pheromone and pheromone-like precursors with typical N-terminal MDA/DF and C-terminal CAAX (cysteine-aliphatic-aliphatic-any amino acid) motifs and internal charged dipeptides for peptide secretion, precursor and further pheromone processing, respectively (Kües, 2015) were identified from transcriptome analysis in *P. cinnabarinus* BRFM 137 (Levasseur *et al.*, 2014). Two of the receptors (Ste3N and Ste3C, now Ste3.1 and Ste3.2 respectively) resembled in sequence typical *B* mating type Ste3-type pheromone receptors and the two others (Ste3.3 and Ste3.4) non-mating-type Ste3-like proteins. Of the putative pheromones, three of the pheromone precursors were very different in sequence as expected when linked to *B* mating type function (Ph proteins) while the four others (Phl precursors) were more similar to each other in sequence and length and resembled precursors of presumed non-mating-type pheromone-like peptides (Levasseur *et al.*, 2014). We now found in the *P. cinnabarinus* BRFM 137 genome four complete *Ste3* genes, one more gene for a putative *B* mating type pheromone (lacking the typical N-terminal MDDF motif likely by a G nucleotide deletion in the original ATG start codon with the consequence of a move of a prior ATG codon as a new start into the reading frame of the gene) and two further genes for non-mating-type pheromone-like peptides. In total, there are the four typical genes for *B* mating type pheromone precursors (*Ph1* to *Ph4*) and five genes (*Phl1* to *Phl5*) for precursors of pheromone-like peptides (Table S2-S3).

Altogether, the *B* mating type and similar genes of *P. cinnabarinus* BRFM 137 were found on four distinct scaffolds (Table S2). Suspected *B* mating type genes reside all on scaffold 184836 at its upper end (196.2 kb total length). The two genes for the non-mating type Ste3-like proteins (*Ste3.3* and *Ste3.4*) are found at the upper end of scaffold 184788 (71.7 kb total length). Gene *Phl5* is present on scaffold 184857 (35.7 kb) next to a gene *Dam1* for an essential component of kinetochores (Buttrick & Millar, 2011). The orthologs of all these genes reside in one continuous DNA region of about 60 kb in length on scaffold 14 in *P. coccineus* BRFM 310 and on scaffold 718000065086 in *P. sanguineus* BRFM 1264, respectively (Table S2). This suggests that all these genes together represent the broader *B* mating type locus in *Pycnoporus* species with the actual *B* mating type genes (from 311212 to 325411 on scaffold 14 in *P. coccineus* BRFM 310; from 1774998 to 192157 on scaffold 718000065086 in *P. sanguineus* BRFM 1264) and any linked genes for non-mating-type pheromone-like peptides (upstream) and Ste3-like proteins (downstream). Because the respective groups of genes on the three scaffolds 184857, 184836 and 184788 in *P. cinnabarinus* BRFM 137 cannot be simply aligned up into a continuous larger DNA region, substantial rearrangements gene orders with translocations appear to have happened in the species. The situation in *P. puniceus* BRFM 1868 is similar where groups of genes are found on scaffold 31, 57 and 6, respectively. All potential genes with *B* mating type function in *P. puniceus* BRFM 1868 reside on scaffold 57 (Table S2).

Regarding the continuous structure of their broader *B* mating type loci, *P. coccineus* BRFM 310 and *P. sanguineus* BRFM 1264 appear much more similar to each other than to the two other *Pycnoporus* species in support of the notion that these two species are closest related. Numbers of *B* mating type and similar non-mating-type genes (Table S3) and relative orders of *Ste3, Ph* and *Phl* genes differ between the four strains (Table S2), suggesting younger evolutionary activities in *B* mating type locus differentiation, possibly involved in *B* allele generation and species diversification.

In all four *Pycnoporus* species, there is an independent locus in the genome separated from the *B* locus for one pheromone-like peptide (the *Phl2* gene in *P. cinnabarinus* BRFM 137 on scaffold 184858). In all species, this gene is neighbored by the mating-related gene *Ste20* coding for a kinase acting in the mitogen-activated protein kinase (MAPK) pathway, the genes of which in some other basidiomycetes are found within mating type loci (Karos *et al.*, 2000; Coelho *et al.*, 2008; Kourist *et al.*, 2015). Lack of close linkage of the *Phl2-Ste20* DNA segment to the *B* mating type locus in the four *Pycnoporus* strains reinforces that the pheromone-like peptides of *Phl* genes have no mating type function. The primary and the predicted mature products of different *Phl* genes within a strain and between species are comparably well conserved (Table S2). This contrasts the relatively little conserved *Ph* genes for typical *B* mating type pheromones. However, some of the processed pheromones appear to be shared between species (Table S2). *Ph* genes are intermingled in the *B* loci in all four species with genes for those Ste3 receptors that group in phylogenetic analysis with typical *B* mating type pheromone receptors and separately from non-mating-type Ste3-like proteins (Levasseur *et al.*, 2014) while genes for non-mating-type Ste3-like proteins are not accompanied by any precursor genes for pheromones or pheromone-like peptides as is expected from observations in other species (Kües *et al.*, 2011; Kües, 2015; Kues *et al.*, 2015).

The same *Phl-Ste20* constellation than in the *Pycnoporus* strains is found on scaffold 3 (models 184724 and 144172 encoded) in the also sequenced *Trametes versicolor* strain FP-101664 SS1

(Floudas *et al.*, 2012) of the broader trametoid clade (Justo & Hibbett, 2011; Carlson *et al.*, 2014). Unlinked on scaffold 8, *T. versicolor* has also copies of the two more strongly conserved non-mating type *Ste3* genes (IDs 73179 and 170723) positioned downstream of its continuous *B* mating type locus (with 7 *Phl* genes and 5 pheromone precursor genes intermingled in between 3 *B* mating type pheromone receptor genes) while the gene for *Dam1* in *T. versicolor* strain FP-101664 SS1 (ID 174288) was moved to another location (scaffold 12). *Dam1*, *Ste3.3* and *Ste3.4* of *T. versicolor* were used for weighing in the trametoid clade in comparisons of encoded proteins between the different *Pycnoporus* species (Table S4). *P. coccineus* BRFM 310 and *P. sanguineus* BRFM 1264 showed for all proteins closer relationships as compared to the other species, similar as for the proteins from genes flanking the *A* mating type locus (Table S4). Looking at sequence similarities of Mip1 and β-fg proteins, also these data suggests that *P. coccineus* and *P. sanguineus* are more closely related to each other than to the other two *Pycnoporus* species and to *T. versicolor* (IDs 138945 and 138946) and it appears that the data reflect the same phylogenetic relations between the four *Pycnoporus* species as formerly reported from ITS sequences, β-tubulin and a laccase (Lesage-Meessen *et al.*, 2011). Importantly, the same tendencies of higher sequence conservation between *P. coccineus* and *P. sanguineus* as compared to the other species are again apparent in comparisons of proteins from *HD1* and *HD2* genes of the *A* mating type loci (*T. versicolor* IDs 187232 and 187235) and in comparisons of Ste3.1 and Ste3.2 proteins from the *B* mating type loci (*T. versicolor* IDs 73174 and 170716), (Table S4).

6. Peroxidase gene models in *Pycnoporus* genomes

A preliminary screening of the automatically-annotated genomes of *Pycnoporus cinnabarinus* BRFM 137, *P. coccineus* BRFM 310 and *P. sanguineus* BRFM 1264 was performed using the Search option (“peroxidase” as search term) at the JGI web-site. Then, a sequence-by-sequence exhaustive analysis was used to identify heme peroxidases (Table S15). The revision of the previously annotated genome sequence of *P. cinnabarinus* BRFM 137 evidenced the presence of thirteen sequences encoding heme peroxidases, which means two over those previously reported by Levasseur *et al.*, 2014. Manual annotation of all the gene models identified was based on the highest sequence identities for each protein sequence derived from the predicted gene, multiple alignments with other 458 basidiomycete heme peroxidase protein sequences, and examination of theoretical molecular structures obtained by homology modeling using crystal structures of related peroxidases as templates and programs implemented by the automated protein homology modeling server “SWISS-MODEL” (Bordoli *et al.*, 2008).

The heme peroxidases could be classified into three different groups as follows: **i)** Cytochrome *c* peroxidases (CCP) (1 model in each genome analyzed) belonging to Class I of prokaryotic-origin peroxidases; **ii)** Class II ligninolytic peroxidases, including members of the new subfamily of "short" manganese peroxidases (MnP) with both Mn-mediated and Mn-independent activity on low redox potential substrates (Fernández-Fueyo *et al.*, 2014b), lignin peroxidases (LiP) able to oxidize high redox potential non-phenolic aromatic compounds, and typical and atypical versatile peroxidases (VP and VP-atypical) sharing catalytic properties of MnP and LiP, and **iii)** Heme-thiolate peroxidases (HTP), some of them with peroxygenase and peroxidase activity (Hofrichter & Ullrich, 2014), differing from the above Class I and Class II peroxidases in the presence of a proximal cysteine (instead of a histidine) acting as the fifth heme iron ligand. No generic

peroxidases (GP) able to oxidize low redox potential aromatic compounds in direct contact with heme were identified from these genomes.

Class II ligninolytic peroxidases could be annotated as LiP, MnP and VP on the basis of the presence or absence of only a few amino acid residues at the substrate oxidation sites (Ruiz-Dueñas *et al.*, 2009) after homology modeling. In this respect: **i**) LiPs are characterized by harboring an exposed catalytic tryptophan homologous to Trp171 in *Phanerochaete chrysosporium* LiP-H8 and Trp164 of *Pleurotus eryngii* VPL; **ii**) MnPs are characterized by containing a Mn(II)-oxidation site near the internal propionate of heme formed by three acidic residues homologous to *P. chrysosporium* MnP1 Glu35, Glu39 and Asp179, and *P. eryngii* VPL Glu36, Glu40 and Asp175; and **iii**) VPs are characterized by presenting both the catalytic tryptophan and the Mn(II) oxidation site of LiPs and MnPs, respectively (atypical VPs contain an atypical Mn-oxidation site formed by one glutamate and two aspartate residues). Regarding MnPs, these peroxidases were annotated as members of the subfamily of short MnPs containing a short C-terminal tail like the exhaustively characterized short MnPs from *Pleurotus ostreatus* and *Ceriporiopsis subvermispora* (Fernández-Fueyo *et al.*, 2014b,a).

A representation of the homology models obtained for the enzymes identified in *P. coccineus* BRFM 310, including key amino acid residues putatively involved in catalysis, is presented in Fig. S14 and Fig. S15 as an example of the homology models obtained for all the peroxidases identified. LiP, MnP and VP models were obtained using the *Phanerochaete chrysosporium* LiPH8 (PDB entries 1B80 and 1B82), *Pleurotus ostreatus* MnP4 (PDB entry 4BM1) and VP1 (PDB entries 4BLK and 4BLN), and *Pleurotus eryngii* VPL2 (PDB entries 2BOQ, 4FCS and 3FMU) crystal structures as templates. Regarding HTPs, the members of this superfamily identified in the *Pycnopous* genomes were modeled with the crystal structures of chloroperoxidase (CPO) (PDB entry 2CIW) from the ascomycete *Leptoxyphium fumago* and unspecific peroxygenase (UPO) (PDB entry 2YP1) from the basidiomycete *Agrocybe aegerita*. Unlike Class II peroxidases, differences in the amino acid residues of the heme environment were observed among the HTP models analyzed. One of the HTPs identified in each genome seems to be a CPO-type peroxidase containing Glu and His residues at the heme distal side involved in enzyme activation by H_2O_2 (Sundaramoorthy *et al.*, 1995) (Fig. S16), whereas the other HTP models harbour Asn and His residues not present neither in CPO nor in UPO (in the latter Glu and Arg residues occupy homologous positions) (Piontek *et al.*, 2013). This fact suggests putative differences in their catalytic properties which should be confirmed by heterologous expression and kinetic characterization of these enzymes. Finally, genes encoding dye-decolorizing peroxidases (DyP) were not identified in any of the *Pycnoporus* genomes analyzed as previously described in the *P. cinnabarinus* genome, a fact that is not frequent among white-rot basidiomycetes where this peroxidase family is widespread with only a few exceptions (Floudas *et al.*, 2012; Ruiz-Dueñas *et al.*, 2013).

A dendrogram showing sequence relationships between the peroxidases identified in the above *Pycnoporus* strains, and structural-functional classification of the ligninolytic peroxidases according to the presence of different catalytic sites in their theoretical molecular structure is shown in Fig. S16. Those peroxidases corresponding to the same isoenzyme in the three *Pycnoporus* strains have the same name and appear clustered together. The only exception is LiP4, which had been previously called LiP5 in *P. cinnabarinus* due to an error naming a VP as if it were a LiP (=LiP4) (Levasseur *et al.*, 2014). As observed, in all cases the enzymes from *P. coccineus* and *P. sanguineus* are more related to each other than with those from *P. cinnabarinus*.

It is interesting to mention that among the *P. cinnabarinus* BRFM 137 peroxidase sequences deposited at JGI and included in this dendrogram, there are three (LiP3, ID# 7501; VP1, ID# 6829; and VP-atypical, ID# 6481) that needed manual curation to yield the sequences published by Levasseur et al.. On the other hand, model 1748658 from *P. sanguineus* BRFM 1264 was annotated as a putative non-functional LiP4. Its amino acid sequence converted into a structural homology model presents the general folding of a typical ligninolytic peroxidase. However it lacks key residues involved in enzyme activation by H_2O_2 (His and Arg residues at the distal heme side), in addition to amino acids in other key positions yielding an active enzyme. This model could be a natural pseudogene or the result of an error in the sequencing/assembling process.

The evolutionary relationships of Class I and Class II heme peroxidases from the *Pycnoporus* strains and other basidiomycetes are shown in Fig. S17. CCPs and hybrid ascorbate-cytocrome *c* peroxidases (APX-CCP) (the latter absent from the genomes of the *Pycnoporus strains*) form two clusters (A and B) clearly separated from class II peroxidases. Among these last, low redox potential GPs (formed by unclustered peroxidases and a few peroxidases grouped together into cluster C) have been described to be at the origin of the high redox potential ligninolytic peroxidases (Floudas *et al.*, 2012), and at least one GP representative of this family is observed in most of the genome sequences of both white and brown-rot basidiomycetes. However, although this is the generality, GP-encoding genes have not been conserved in the *Pycnoporus* species' genomes, as also observed in other sequenced species (*Dichomitus squalens*, *Trametes versicolor* and *Ganoderma* sp) belonging, like those from the genus *Pycnoporus*, to the core Polyporoid clade (Ruiz-Dueñas *et al.*, 2013). MnPs derived from ancestral GPs were the first ligninolytic peroxidases to appear by progressive incorporation of three acidic residues forming the Mn-oxidation site (Floudas *et al.*, 2012). In fact, there are atypical MnPs encoded in the *Auricularia delicata*, *Fomitiporia mediterranea* and *Stereum hirsutum* genomes that contain only two of the acidic residues representing intermediate evolutionary states between GPs and MnPs. Regarding the members of the MnP family, these appear grouped into three different clusters in the dendrogram. Long MnPs, including the classical enzymes from *P. chrysosporium* (Gold *et al.*, 2000), and extralong MnPs grouped into cluster D were initially classified as two different MnP subfamilies (Floudas *et al.*, 2012), although studies have demonstrated that both types of MnPs present similar catalytic properties and stability, and in consequence they are considered members of the same MnP family (Fernández-Fueyo *et al.*, 2014a). Unlike long and extralong MnPs, short MnPs are grouped into two different clusters (E and F). On one hand, short MnPs comprised in cluster E are related to a group of atypical VPs (and a few VPs) from species of the core Polyporoid clade, including three atypical forms corresponding to the same isoenzyme in three of the four *Pycnoporus* strains analyzed. On the other hand, most of short MnPs, including those identified in the *Pycnoporus* strains analyzed, are intermixed with two VP/LiP intermediates from *C. subvermispora* (Fernández-Fueyo *et al.*, 2012) and a few VPs from *Spongipellis* sp, *Bjerkandera adusta*, *Bjerkandera* sp. and *Pleurotus* species constituting cluster F. Finally, cluster G is characterized by containing LiPs (from *B. adusta*, *Phlebia brevispora*, *Phlebia radiata*, *P. chrysosporium*, *Phlebiopsis gigantea* and *T. versicolor*) and some VPs, including those from the three *Pycnoporus* strains.

The analysis of the position of the peroxidase genes in the different *Pycnoporus* genomes revealed that four of them (*lip*3, *lip*2, *lip*1 and *mnp*3) co-localize in the same syntenic block encompassing at least 11 genes. This peroxidase grouping was already described in *P. cinnabarinus* and in the closely-related *T. versicolor* (Levasseur *et al.*, 2014). In a similar way,

*htp*1, *htp*4, *htp*2 and *vp*2 cluster together on the same scaffold in the *P. coccineus and P. sanguineus* genome assemblies (*htp*1, *htp*2 and *vp*2 in *P. cinnabarinus* due to the absence of *htp4* in its genome sequence).

Finally, the analysis of the evolutionary relationships of 117 basidiomycete HTPs (Fig. S18) confirmed that the enzymes identified in the *Pycnoporus* species belong to two different groups (clusters B and C in the dendrogram), probably exhibiting different catalytic properties, as previously predicted from the analysis of the amino acid residues located at the heme environment.

7. Copper Radical Oxidases

We identified in each genome seven genes coding for Copper Radical Oxidases (CROs) instead of 9 in the well-studied white-rot fungus *Phanerochaete chrysosporium*. Among them, three code for glyoxal oxidases (Table S21). GLX catalyze the two-electron oxidation of simple aldehydes and hydroxycarbonyls with the reduction of molecular oxygen to hydrogen peroxide. GLX thereby generate extracellular hydroperoxide that supports peroxidase activity and lignin degradation (Kersten & Kirk, 1987). For each predicted CRO in *P. cinnabarinus* we identified the orthologous gene in *P. coccineus* and *P. sanguineus*, including for the two characterized PciGLOX1 and PciGLOX2 enzymes (Daou *et al.*, 2016).

8. Laccases

We identified five laccase, one multicopper oxidase and one ferroxidase coding genes in each genome (Table S22). The exon-intron structure of the *lac* genes was conserved with 11 introns in *lac1*, *lac*3, and *lac4* and 13 exons in *lac2* and *lac5*. The *lac3* and *lac1* genes were in tandem on scaffold 185007 from Pycci, scaffold 16 from Pycco and scaffold 7180000650860 from Pycsa, with intergenic regions ranging from 25,9 kb to 32,2 kb.

9. (GMC)-oxidoreductases

(GMC)-oxidoreductases were identified by using the CAZy annotation pipeline which is based on blastp search thresholds using characterized enzymes as queries and the conservation of HMM profiles (Lombard *et al.*, 2014). To enhance protein function prediction, the proteins classified as AA3s in the CAZy database were further compared with 46 sequences of characterized fungal AA3s (Sützl *et al.*, 2018). Phylogenetic trees were built using ClustalW for the alignment of the sequences and MEGA-CC (Kumar *et al.*, 2012) for maximum likelihood analysis with 500 bootstrap values (Fig. S12, Fig. S20-22).

10. Small Secreted Proteins

Predicted secreted proteins were identified that met the five conditions : 1) a peptide signal predicted by SignalP, 2) predicted as secreted by TargetP and wolfpsort, 3) no Lys-Asp-Glu-Leu (KDEL) motif in C-terminal (prosite accession'PS00014'), and 4) 0 or 1 transmembrane helix found by TMHMM (Pellegrin *et al.*, 2015). If there is one helix it should overlap the signal peptide. Small Secreted Proteins were predicted secreted proteins <300 aa long.

11. Secretory pathway

Genes coding for proteins involved in the secretory and glycosylation pathways were identified in Pycci using reciprocal best blast hits and e value<10^{-10} with *Aspergillus niger* proteins as queries (Pel *et al.*, 2007). Proteins involved in the secretion pathway included genes involved in protein entry into the endoplasmic reticulum (signal recognition, signal peptidase complex, translocation into the ER, protein folding into the ER), Protein misfolding (Unfolded Protein Response (UPR), ER associated Degradation (ERAD), proteasome), protein complex involved in protein transport (exocyst complex, SEC34/SEC35 complex, Trapp complex, COPI and COPII subunits), proteins involved in vesicle formation and docking (SNARE proteins, secretion related GTPases and interacting proteins, ER to Golgi and Intra-Golgi transport, Golgi to endosome transport, Vacuolar protein sorting, Cellular export and secretion; Table S23). Proteins involved in glycosylation pathways included proteins involved in the biosynthesis of nucleotide sugars for glycosylation events (UDP-Glucose, UDP-N-acetyl-glucosamine, GDP-mannose, UDP-galactose), transporters of sugar nucleotide donors (GDP-mannose, UDP-GlcNac, UDP-galactose, UDP-galactofuranose, Oligosaccharyltransferase subunits), synthesis of the dolicholphosphate linked ER-precursor Glc3Man9GlcNAc2, processing of the ER-precursor Glc3Man9GlcNAc2 after transfer to a polypeptide, Golgi mannosyltransferase, O-Glycosylation in ER, putative alpha-1,2-mannosidase with no homology to the MNS1/ER-alpha-1,2-mannosidase family, GPI anchor biosynthesis, GPI-anchor transamidase complex (Table S24). Functional annotations were propagated from *P. cinnabarinus* predicted proteins to *P. coccineus* and *P. sanguineus* predicted proteins by homology search using orthoMCL with default parameters and the four genomes *A. niger* CBS 513.88, Pycci, Pycco and Pycsa (Tables S25-26).

12. Glutathione-S Transferases

The GST-coding genes were predicted with a combination of automated gene callers, and blastP tool using sequences from *Phanerochaete chrysosporium* that have been phylogenetically classified and functionally characterized for many of them (Meux *et al.*, 2011, 2013; Mathieu *et al.*, 2013; Thuillier *et al.*, 2013, 2014; Roret *et al.*, 2015). The repartition of the sequences within the various classes was based both on phylogenetic relationship between *P. chrysosporium* and *Pycnoporus* sequences and active site comparison especially for GSTs from the Omega, GHR and Ure2p classes.

In fungi, seven GST classes have been defined (Morel *et al.*, 2009). Among them, GSTs involved in translation (EFB class) and GSTs associated to membranes (MAPEG class) are generally not considered as true GST. Isoforms from the other classes (GSTO, GHR, Ure2p, GSTFuA and GTT2) are putatively involved in detoxification processes. The Pycci, Pycco and Pycsa genomes carry respectively 23, 34 and 35 gene copies. For comparison, *Phanerochaete chrysosporium*, *Serpula lacrymans*, *Postia placenta* and *Trametes versicolor* exhibit respectively 25, 30, 45 and 41 GST gene copies in their genomes (Morel *et al.*, 2013). As other lignolytic fungi, GSTO, Ure2pA and GSTFuA are expanded in the three Pycnoporus species compared to GHR and Ure2pB classes. The expansion of GSTO class could be explained by a high number of serine containing GSTs. Contrary to the classical cysteine-containing GSTOs, which display a deglutathionylation activity (Meux *et al.*, 2011), serine-containing GSTOs exhibit the classical glutathione transferase activity, known to be involved in detoxification pathways (Deroy *et al.*, 2015). Since GSTOs are able to interact with wood extractives, which are wood-derived

molecules with potential antimicrobial activity, the serine containing GSTOs could protect *Pycnoporus* sp. from extractive toxicity.

However, these genes are not highly expressed in the culture conditions tested and are not induced by woody substrates. This is also the case for the GSTs from the other classes, except for one GSTFuA, that is induced in *P. coccineus* BRFM 310 during growth on pine. GSTFuA are fungal specific GSTs, that, additionally to their glutathionylation activity, have the properties to alternatively act as ligandins by binding wood extractive compounds at a L-site overlapping the glutathione binding pocket.

Noticeable was the difference between the global expression pattern of the GSTome for the three strains independently from the substrate. In *P. cinnabarinus* BRFM 137, the most strongly expressed GST gene codes for a predicted GTT2.1. GTT2.1s are specifically found in wood degraders. GTT2.2 expression is induced in *P. chrysopsporium* in presence of oak acetonic extracts and the recombinant protein is not active as a glutathione transferase but rather as a peroxidase (Thuillier *et al.*, 2014). This GTT2.1 could thus be involved in oxidative stress rescue in *P. cinnabarinus*. Ure2pB genes are highly transcribed both in *P. coccineus* BRFM 310 and *P. sanguineus* BRFM 1264 but not in *P. cinnabarinus* BRFM 137. The physiological role of Ure2pB is still unknown but the *P. chrysosporium* ortholog is constitutively expressed and displays a deglutathionylation activity (Thuillier *et al.*, 2013).

Altogether, these results show that the regulation of the GSTome differs in each strain despite very similar GST gene repertoires.

13. P450s

The RNASeq analysis showed several genes up-regulated or highly transcribed on (ligno)cellulose that had a cytochrome P450 predicted function according to KOGG classification. In this study, a gene was considered up-regulated if its log2 fold change was higher than 2 during growth on (ligno)cellulosic substrate as compared to maltose or if its log2 read count was higher than 12 on one of the tested substrates (cellulose, wheat straw, pine or aspen). The sequences of retrieved predicted cytochrome P450s were subjected to blastp similarity searches in the Fungal Cytochrome P450 Database (FCPD; last accessed 01 July 2018; (Park *et al.*, 2008)).

14. Peptidases

Blastx searches of gene models from all three species of *Pycnoporus* against InterPro and MEROPS databases (Mitchell *et al.*, 2015; Rawlings *et al.*, 2016) followed by hand curation found 340 putative peptidases encoded by the *P. coccineus* BRFM 310, 354 encoded by *P. sanguineus* BRFM 1264, and 321 encoded by *P. cinnabarinus* BRFM 137. These numbers were in good agreement with the automated annotation results at the Joint Genome Institute where the respective peptidase counts are 360, 378, and 340. As in most basidiomycetes, the large majority of peptidase genes encode members of three MEROPS-recognized mechanistic classes: aspartic proteases, metalloproteases, and serine proteases (Table S27). Aspartic peptidase genes average just fewer than twenty percent of the total peptidases in basidiomycete genomes (Lilly and Gathman, unpublished analysis). Metallopeptidases and serine peptidases average 31 and 32 percent respectively. *Pycnoporus* species have a larger proportion of genes encoding aspartic

peptidases, a situation found in *Phanerochaete chrysosporium* and *Postia placenta* among those species with published genomes.

Three families of peptidases, aspartic peptidase A1, serine peptidase family S10, and serine peptidase family S53 (including cross-listed S8/S53 members) are expanded in all three *Pycnoporus* species. Together these three families represent just under 30 % of all peptidase genes found in each genome. Members of these families (Type examples: A1 = pepsin, S10 = carboxypeptidase Y, S53=sedolysin) all are active only at acid pH. In addition, in most fungi the vast majority of these enzymes are secreted. A similar situation exists for the *Pycnoporus* species, where in *P. sanguineus* 89 % of the gene models for these families have signal peptides based on SignalP analysis; only slightly fewer (80%) have signal peptides in *P. coccineus*. In contrast, few of these proteins are predicted to have signal peptides in *P. cinnabarinus*, possibly due to lower quality of the gene models.

Proteomic data from the secretome of these species when grown on six different carbon sources found only members of the aforementioned expanded families. However, each species showed a different spectrum of secreted peptidases. The total number of peptidase gene products identified in the secretomes was highest for *P. coccineus* (31 peptidases identified), including 12 predicted S53 gene products. In *P. cinnabarinus* and *P. sanguineus*, across all media, one specific A1 peptidase contributed over half of the total A1 proteomic spectra, and the greatest number of spectra of any peptidase found. This peptidase also returned the highest number of A1 peptidase spectra on each different medium. In *P. coccineus* secretomes, one specific A1 and one peptidase assigned to the S8 and S53 family accounted for half of the spectra. A comparison of the sequences of the A1 peptidases genes from all three species shows that, in most cases, it is the products of the directly orthologous genes that are found in the medium. Similarly, the S10 peptidase genes found in the various media are, in most cases, products of orthologous genes (not shown). The paucity of secreted products of S53 genes in *P. sanguineus* and *P. cinnabarinus* makes a similar analysis difficult for them.

Overall expression data for peptidases in the three species in basal maltose medium revealed that members of all major mechanistic classes are expressed. However, expression was found for only a fraction of the total population of peptidase genes in each species (*P. coccineus* = 181 expressed/340 total; *P. sanguineus* = 97 expressed/354 total; *P. cinnabarinus* = 125 expressed/321 total). Serine peptidases represented the highest proportion of expressed peptidase genes in each species, followed by metalloprotease genes and aspartic protease genes. Transcription levels were studied on five different media, and expression levels compared to basal maltose medium. For those situations where the Log2FoldChange > |2|, most of the up-regulated genes were members of the expanded A1, S10, and S53 peptidase gene families.

15. References

Bolstad B. **2019**. preprocessCore: A collection of pre-processing functions (R package version 1.46.0, Ed.). *https://github.com/bmbolstad/preprocessCore.*

Bordoli L, Kiefer F, Arnold K, Benkert P, Battey J, Schwede T. **2008**. Protein structure homology modeling using SWISS-MODEL workspace. *Nat Protoc.* **4**: 1.

Brown AJ, Casselton LA. **2001**. Mating in mushrooms: increasing the chances but prolonging the affair. *Trends in genetics : TIG* **17**: 393–400.

Buttrick GJ, Millar JBA. **2011**. Ringing the changes: emerging roles for DASH at the kinetochore-microtubule Interface. *Chromosome research : an international journal on the molecular, supramolecular and evolutionary aspects of chromosome biology* **19**: 393–407.

Carlson A, Justo A, Hibbett DS. **2014**. Species delimitation in Trametes: a comparison of ITS, RPB1, RPB2 and TEF1 gene phylogenies. *Mycologia* **106**: 735–745.

Coelho MA, Rosa A, Rodrigues N, Fonseca Á, Gonçalves P. **2008**. Identification of Mating Type Genes in the Bipolar Basidiomycetous Yeast Rhodosporidium toruloides: First Insight into the MAT Locus Structure of the Sporidiobolales *Eukaryotic Cell* **7**: 1053 LP – 1061.

Couturier M, Navarro D, Chevret D, Henrissat B, Piumi F, Ruiz-Dueñas F, Martinez AT, Grigoriev I V, Riley R, Lipzen A, *et al.* 2015. Enhanced degradation of softwood versus hardwood by the white-rot fungus *Pycnoporus coccineus. Biotechnol Biofuels* **8:216**.

Daou M, Piumi F, Cullen D, Record E, Faulds C. **2016**. Heterologous production and characterization of two glyoxal oxidases from *Pycnoporus cinnabarinus*. *Appl Environ Microbiol.* **82**: 4867–4875.

Deroy A, Saiag F, Kebbi-Benkeder Z, Touahri N, Hecker A, Morel-Rouhier M, Colin F, Dumarcay S, Gérardin P, Gelhaye E. **2015**. The GSTome reflects the chemical environment of white-rot fungi. *PLoS One* **10**: e0137083.

Fernández-Fueyo E, Acebes S, Ruiz-Dueñas FJ, Martínez MJ, Romero A, Medrano FJ, Guallar V, Martínez AT. **2014a**. Structural implications of the C-terminal tail in the catalytic and stability properties of manganese peroxidases from ligninolytic fungi. *Acta Crystallogr D Biol Crystallogr.* **70**: 3253–3265.

Fernández-Fueyo E, Ruiz-Dueñas FJ, Martínez MJ, Romero A, Hammel KE, Medrano FJ, Martínez AT. **2014b**. Ligninolytic peroxidase genes in the oyster mushroom genome: Heterologous expression, molecular structure, catalytic and stability properties and lignin-degrading ability. *Biotechnol Biofuels* **7**: 2.

Fernández-Fueyo E, Ruiz-Dueñas FJ, Miki Y, Martínez MJ, Hammel KE, Martínez AT. **2012**. Lignin-degrading peroxidases from genome of selective ligninolytic fungus *Ceriporiopsis subvermispora*. *J Biol Chem.* **287**: 16903–16916.

Floudas D, Binder M, Riley R, Barry K, Blanchette RA, Henrissat B, Martínez AT, Otillar R, Spatafora JW, Yadav JS, *et al.* 2012. The Paleozoic origin of enzymatic lignin decomposition reconstructed from 31 fungal genomes. *Science* **336**: 1715–1719.

Gold MH, Youngs HL, Gelpke MD. **2000**. Manganese peroxidase. *Met Ions Biol Syst* **37**: 559–586.

Hofrichter M, Ullrich R. **2014**. Oxidations catalyzed by fungal peroxygenases. *Curr Opin Chem Biol.* **19**: 116–125.

James TY, Kues U, Rehner SA, Vilgalys R. **2004**. Evolution of the gene encoding mitochondrial intermediate peptidase and its cosegregation with the A mating-type locus of mushroom fungi. *Fungal genetics and biology : FG & B* **41**: 381–390.

James TY, Sun S, Li W, Heitman J, Kuo H-C, Lee Y-H, Asiegbu FO, Olson A. **2013**. Polyporales genomes reveal the genetic architecture underlying tetrapolar and bipolar mating systems. *Mycologia* **105**: 1374–1390.

Justo A, Hibbett DS. **2011**. Phylogenetic classification of Trametes (Basidiomycota, Polyporales) based on a five-marker dataset. *Taxon* **60**: 1567–1583.

Karos M, Chang YC, McClelland CM, Clarke DL, Fu J, Wickes BL, Kwon-Chung KJ. **2000**. Mapping of the *Cryptococcus neoformans* MATalpha locus: presence of mating type-specific mitogen-activated protein kinase cascade homologs. *Journal of bacteriology* **182**: 6222–6227.

Kersten PJ, Kirk TK. **1987**. Involvement of a new enzyme, glyoxal oxidase, in extracellular H2O2 production by *Phanerochaete chrysosporium*. *J Bacteriol.* **169**: 2195–2201.

Kim D, Pertea G, Trapnell C, Pimentel H, Kelley R, Salzberg SL. **2013**. TopHat2: accurate alignment of transcriptomes in the presence of insertions, deletions and gene fusions. *Genome Biol.* **14**: R36–R36.

Kourist R, Bracharz F, Lorenzen J, Kracht ON, Chovatia M, Daum C, Deshpande S, Lipzen A, Nolan M, Ohm RA, *et al.* 2015. Genomics and transcriptomics analyses of the oil-accumulating basidiomycete yeast T*richosporon oleaginosus*: insights into substrate utilization and alternative evolutionary trajectories of fungal mating systems. *mBio* **6**: e00918.

Kües U. **2015**. From two to many: Multiple mating types in Basidiomycetes. *Fungal Biology Reviews* **29**.

Kües U, James TY, Heitman J. **2011**. 6 Mating Type in Basidiomycetes: Unipolar, Bipolar, and Tetrapolar Patterns of Sexuality. In: Pöggeler S, Wöstemeyer J, eds. Evolution of Fungi and Fungal-Like Organisms. Berlin, Heidelberg: Springer Berlin Heidelberg, 97–160.

Kues U, Nelson DR, Liu C, Yu G-J, Zhang J, Li J, Wang X-C, Sun H. **2015**. Genome analysis of medicinal *Ganoderma* spp. with plant-pathogenic and saprotrophic life-styles. *Phytochemistry* **114**: 18–37.

Kües U, Richardson WVJ, Tymon A, Mutasa-Gottgens E, Göttgens B, Gaubatz S, Gregoriades A, Casselton L. **1992**. The combination of dissimilar alleles of the Aα and Aβ gene complexes, whose proteins contain homeo domain motifs, determines sexual development in the mushroom *Coprinus cinereus*. *Genes & development* **6**: 568–577.

Kumar S, Stecher G, Peterson D, Tamura K. **2012**. MEGA-CC: computing core of molecular evolutionary genetics analysis program for automated and iterative data analysis. *Bioinformatics* **28**: 2685–2686.

Lamark JB. **1783**. *Agaricus ruber*. Paris.

Leek JT, Johnson WE, Parker HS, Jaffe AE, Storey JD. **2012**. The sva package for removing batch effects and other unwanted variation in high-throughput experiments. *Bioinformatics* **28**: 882–883.

Lesage-Meessen L, Haon M, Uzan E, Levasseur A, Piumi F, Navarro D, Taussac S, Favel A, Lomascolo A. **2011**. Phylogeographic relationships in the polypore fungus *Pycnoporus* inferred from molecular data. *FEMS Microbiol Lett.* **325**: 37–48.

Levasseur A, Lomascolo A, Chabrol O, Ruiz-Duenas F, Boukhris-Uzan E, Piumi F, Kues U, Ram A, Murat C, Haon M, *et al.* 2014. The genome of the white-rot fungus *Pycnoporus cinnabarinus*: a basidiomycete model with a versatile arsenal for lignocellulosic biomass breakdown. *BMC Genomics* **15**: 486.

Lomascolo A, Uzan-Boukhris E, Gimbert I, Sigoillot J-C, Lesage-Meessen L. **2011**. Peculiarities of *Pycnoporus* species for applications in biotechnology. *Applied microbiology and biotechnology* **92**: 1129–49.

Lombard V, Golaconda Ramulu H, Drula E, Coutinho PM, Henrissat B. **2014**. The carbohydrate-active enzymes database (CAZy) in 2013. *Nucleic Acids Res.* **42**: D490-5.

Love MI, Huber W, Anders S. **2014**. Moderated estimation of fold change and dispersion for RNA-seq data with DESeq2. *Genome Biol.* **15**: 550.

Mathieu Y, Prosper P, Favier F, Harvengt L, Didierjean C, Jacquot J-P, Morel-Rouhier M, Gelhaye E. **2013**. Diversification of fungal specific Class A glutathione transferases in saprotrophic fungi. *PLOS ONE* **8**: e80298.

Meux E, Morel M, Lamant T, Gérardin P, Jacquot J-P, Dumarçay S, Gelhaye E. **2013**. New substrates and activity of Phanerochaete chrysosporium Omega glutathione transferases. *Biochimie* **95**: 336–346.

Meux E, Prosper P, Ngadin A, Didierjean C, Morel M, Dumarçay S, Lamant T, Jacquot J-P, Favier F, Gelhaye E. **2011**. Glutathione transferases of Phanerochaete chrysosporium: S-glutathionyl-p-hydroquinone reductase belongs to a new structural class. *J Biol Chem.* **286**: 9162–9173.

Mitchell A, Chang H-Y, Daugherty L, Fraser M, Hunter S, Lopez R, McAnulla C, McMenamin C, Nuka G, Pesseat S, *et al.* 2015. The InterPro protein families database: the classification resource after 15 years. *Nucleic Acids Res.* **43**: D213–D221.

Miyauchi S, Rancon A, Drula E, Hage H, Chaduli D, Favel A, Grisel S, Henrissat B, Herpoël-Gimbert I, Ruiz-Dueñas FJ, *et al*. 2018. Integrative visual omics of the white-rot fungus *Polyporus brumalis* exposes the biotechnological potential of its oxidative enzymes for delignifying raw plant biomass. *Biotechnol Biofuels*. **11**:201.

Morel M, Meux E, Mathieu Y, Thuillier A, Chibani K, Harvengt L, Jacquot J-P, Gelhaye E. **2013**. Xenomic networks variability and adaptation traits in wood decaying fungi. *Microb Biotechnol.* **6**: 248–263.

Morel M, Ngadin AA, Droux M, Jacquot JP, Gelhaye E. **2009**. The fungal glutathione S-transferase system. Evidence of new classes in the wood-degrading basidiomycete *Phanerochaete chrysosporium*. *Cell Mol Life Sci.* **66**: 3711–3725.

Navarro D, Rosso M-N, Haon M, Olivé C, Bonnin E, Lesage-Meessen L, Chevret D, Coutinho PM, Henrissat B, Berrin J-G. **2014**. Fast solubilization of recalcitrant cellulosic biomass by the basidiomycete fungus Laetisaria arvalis involves successive secretion of oxidative and hydrolytic enzymes. *Biotechnology for biofuels* **7**: 143.

Nobles MK, Frew BP. **1962**. Studies in wood-inhabiting Hymenomycetes V. The genus *Pycnoporus* Karst. . *Can J Bot.* **40**: 987–1016.

Park J, Lee S, Choi J, Ahn K, Park B, Park J, Kang S, Lee Y-H. **2008**. Fungal cytochrome P450 database. *BMC Genomics* **9**: 402.

Pel HJ, Winde JH, Archer DB, Dyer PS, Hofmann G, Schaap PJ, Turner G, Vries RP, Albang R, Albermann K, *et al.* 2007. Genome sequencing and analysis of the versatile cell factory *Aspergillus niger* CBS 513.88. *Nat Biotechnol* **25**.

Pellegrin C, Morin E, Martin FM, Veneault-Fourrey C. **2015**. Comparative analysis of secretomes from ectomycorrhizal fungi with an emphasis on small-secreted proteins. *Front. Microbiol.* **6**.

Piontek K, Strittmatter E, Ullrich R, Grobe G, Pecyna MJ, Kluge M, Scheibner K, Hofrichter M, Plattner DA. **2013**. Structural basis of substrate conversion in a new aromatic peroxygenase: Cytochrome P450 functionality with benefits. *J Biol Chem.* **288**: 34767–34776.

Rawlings ND, Barrett AJ, Finn R. **2016**. Twenty years of the MEROPS database of proteolytic enzymes, their substrates and inhibitors. *Nucleic Acids Res.* **44**: D343–D350.

Roret T, Thuillier A, Favier F, Gelhaye E, Didierjean C, Morel-Rouhier M. **2015**. Evolutionary divergence of Ure2pA glutathione transferases in wood degrading fungi. *Fungal Genetics and Biology* **83**: 103–112.

Ruiz-Dueñas FJ, Lundell T, Floudas D, Nagy LG, Barrasa JM, Hibbett DS, Martínez AT. **2013**. Lignin-degrading peroxidases in Polyporales: An evolutionary survey based on ten sequenced genomes. *Mycologia* **105**: 1428–1444.

Ruiz-Dueñas FJ, Morales M, García E, Miki Y, Martínez MJ, Martínez AT. **2009**. Substrate oxidation sites in versatile peroxidase and other basidiomycete peroxidases. . *J Exp Bot.* **60**: 441–452.

Sundaramoorthy M, Terner J, Poulos TL. **1995**. The crystal structure of chloroperoxidase: A heme peroxidase-cytochrome P450 functional hybrid. *Structure* **3**: 1367–1377.

Sützl L, Laurent CVFP, Abrera AT, Schütz G, Ludwig R, Haltrich D. **2018**. Multiplicity of enzymatic functions in the CAZy AA3 family. *Appl Microbiol Biotechnol.* **102**: 2477–2492.

Thuillier A, Chibani K, Belli G, Herrero E, Dumarçay S, Gérardin P, Kohler A, Deroy A, Dhalleine T, Bchini R, *et al.* 2014. Transcriptomic responses of *Phanerochaete chrysosporium* to oak acetonic extracts: focus on a new glutathione transferase. *Appl Environ Microbiol.* **80**: 6316–6327.

Thuillier A, Roret T, Favier F, Gelhaye E, Jacquot J-P, Didierjean C, Morel-Rouhier M. **2013**. Atypical features of a Ure2p glutathione transferase from *Phanerochaete chrysosporium*. *FEBS Lett.* **587**: 2125–2130.

Tymon AM, Kües U, Richardson W V, Casselton LA. **1992**. A fungal mating type protein that regulates sexual and asexual development contains a POU-reflated domain. *The EMBO journal* **11**: 1805–1813.

Wehrens R, Buydens LMC. **2007**. Self- and Super-Organizing Maps in R: The kohonen Package. *J. Stat. Softw.* **21**: 1–19.

Table S1. Genome features for *Cylindrobasidium torrendii* (Cylto1), *Fistulina hepatica* (Fishe1), *Dichomitus squalens* (Dicsq1), *Fomitopsis pinicola* (Fompi3), *Polyporus brumalis* (Polbr1), *Pycnoporus cinnabarinus* (Pycci1), *Pycnoporus coccineus* (Pycco1), *Pycnoporus puniceus* (Pycpun1), *Pycnoporus sanguineus* (Pycsa1), *Trametes versicolor* (Trave1) and *Wolfiporia cocos* (Wolco1).

JGI genome ID	Cylto1	Fishe1	Dicsq1	Fompi3	Polbr1	Pycci1	Pycco1	Pycpun1	Pycsa1	Trave1	Wolco1
Genome size (Mbp)	31.57	33.85	42.75	41.61	45.72	33.67	32.76	30.26	36.04	44.79	50.48
TE Coverage (%)	1.54	11.27	9.33	5.61	3.66	8.15	1.8	12.33	4.91	4.05	28.83
TE Coverage (Mbp)	0.49	3.81	3.99	2.33	1.67	2.74	0.59	3.73	1.77	1.81	14.55
Number of protein coding genes	13940	11244	12290	13885	18244	10442	12690	10050	14165	14296	12746
Number of Contigs	1222	914	2852	988	1040	2036	469	105	2046	1443	2228
Number of Scaffolds	1149	588	542	504	621	784	222	105	657	283	348
Scaffold N50	94	27	16	37	34	54	20	12	35	7	7
Scaffold L50 (Mbp)	0.09	0.35	0.64	0.37	0.36	0.17	0.47	0.79	0.32	2.88	2.54
Number of predicted secreted proteins	861	469	724	676	958	558	751	594	756	867	483
Number of predicted proteins	13940	11244	12290	13885	18244	10442	12690	10050	14165	14296	12746
BUSCO Complete protein sequences	1288	1270	1309	1315	1320	1268	1321	1309	1293	1317	1308
BUSCO Fragmented protein sequences	24	32	14	11	10	32	7	14	21	11	12
BUSCO Missing protein sequences	23	33	12	9	5	35	7	12	21	7	15

Table S2. *A* and *B* mating type loci and related genes as manually annotated in the JGI databases. Protein IDs and gene locations on scaffolds refer to the JGI databases. For *Pycnoporus cinnabarinus* BRFM 137, protIDs are indicated when the structural annotation on the JGI database is correct. Colored sequences in pheromone precursor sequences mark predicted pheromone sequences: yellow: unique sequence, light-blue: sequence occurs more than once in a species, green: sequence occurs also in another species.

Protein	Gene location	Sequence
***Pycnoporus cinnabarinus* BRFM 137**		
***A* mating type locus**		
Mip1<br>(8574)	scf185007:30946-28465	MLAKAAKHALSRPTSLLLRVPLRVFTRGQWDPRLVSRS<br>ATTSAIQPASVDDMNLIAFFDYPQPPQNVTNPTGIFGH<br>RSLTTPAAFIALADSTLRRAQLLTARILRARESRDELF<br>KVVKNLDRLSDMLCGVIDLAELLRNAHPDPAWVQSADE<br>VYEKMCEFMNVLNTNVGLYEVLVSVLSDQEIVKSLCPE<br>AYQTALIFWRDFEKSGIHLTPAQRERFVSLSTEILVLG<br>RQFLNETAAPRPPARIKFSELQSVKDLGMGARLRLQAQ<br>VTKRELLVYPGSLQAQMIMRSAPDEEARKKVYMAANSS<br>TSHQIETLERLLRARGELARLVGKESYAHMALADKMAK<br>SPENVQHFLDALMDQTRLYARRALRNLSMRKQAHLKTS<br>PFPIIQPWDRDYYCPPEPPAPPVLPPLTLGTVFMALSR<br>LFRALYGISLRLAEPAPGEAWHVDVRKLEVVDEEHGVL<br>GWIYADLFARSGKPAGAAHYTVRCSRRTDDDDEEGDLM<br>FADERDRTAVQLSRDFEVERQSRLPGQPLIFQLPVVVL<br>LCEFMRPTVASGPTVLDWHEVLTLFHEMGHAMHSMIGR<br>TEYHNVSGTRCATDFVELPSILMEHFLNSSSVLSLFQR<br>DLEGEFCHTARPHEDPCRSIDTHTQILLAMLDQIYHSS<br>AVLHAGFDSTAALETLSSDRGLIPYIPGTSWQTQFGHL<br>FGYGATYYSYLFDRAIASRVWSKLFSEAPLNRDMGERL<br>KQEVLRFGGGRDPWVMVGTLVDSADLTVGDTKAMAEVG<br>RWRIEDEISMYGRH*
a2-1<br>(HD2)	scf185007:26368-28230	MPSATVFAVHTDTFRRIERTALRLAQLTVSGSSKPHVA<br>PPTLSKPDKLSLRHPAPIAPQLLALGVDASSSTRISCA<br>FLLAASQLKDLCESAFQRRIKPEDHSHLLLDRTLPTSV<br>AISLSAIYERALHEWTSFLLQNVTPRVLKLQRRLTHSS<br>KQPKRATFKPPFNQSAVPLLERFFDQNAFPSRLEKYEL<br>AVQCHMEYRQIHVWFQNRRRRSRKEGKELKQRLSENSV<br>LQSLETSVVEALLPADVDEDAEQLGAEKGDPLLQTAGD<br>CLHSRAPPHAFPSPWPPLCPYDPFPISPGERRFTTPWP<br>RKSGQSISSGPLIDMDQLERAFSTLSLSGETLCKFSEI<br>GAGHPWQRRPPLCIGFSVLCSRAPHPALVNRCGTLSCS<br>PGGTAALLATANPRIQRPSAPVATEIALQLPERTYQSS<br>LVQFRRPPESTKSASTPPCTASGPRLRRGLPRRIPKNP<br>PLKCHASSSSRPLGQAQIEKTRCWSSTSPVRPSSPDGS<br>GSDTDSLPSTPLFNPTRSFTAELCGVDFGSDGLIPDLA<br>VDPALWFTTQNPSSSVVRIAGVDESLASHTRIATDT*
a1-1<br>(HD1)	scf185007:26143-24762	MSTLTKRLLSAEDDFLTALEQGTEALASFDRHWEALMN<br>EIDRAMEFDSLDYDTLILVHSTSLRISVLADASIDLFR<br>TQDAYTSELMSHLDSLLSDIALSPKPSQHQLPPSSPRK<br>FETSPPVKMPTSTPSPEALVTKTCKLPKRKWDEDSSSS<br>GTQKRQRTTANAQSVPRSHCLSTARVTSTKQPRFDPPE<br>PASTASRGEAGAFEITPLLLCRKRRASDAELSCRLGGP<br>KRVHLGPRVHAVSDSFTASLNIRDAQRPAVESVPSFPV<br>NCYPSTVVTDTSLNTQQQDGVDGGFTSATFPLQRSDCH<br>NQSSSHTTVDDLDEFLRYILEPNCITNLELDCSTSEDD

		ESAPSTPSTGSIAKRDYVYDFESPISTSSPISLSHIGT<br>PPLIVGSHVWWGRCWWLDTSMRLGGSSGY*
β-fg	scf185007:23338-24263	MDNFMNFAKQGMQAYERSHSDVSRTGGQEYNAPSQTSA<br>HDRPQFDDDEVVNTARREGSGDSSLFSSALSFVKQNKT<br>AHQEPIDEEGVQHAHRKAYEEGSAGSLSAGSLGSAAAL<br>QVLRKFTSGGASSGGGSQSQLISLAMAEASKLFDQSGG<br>ASSGNKQDAVNGAAMTIMKLLVQSKFSGGPVDGANSGG<br>LSALMGLASKFI*
***B* mating type locus**		
Dam1	scf184857:20718-19738<br>(14 internal aa missing by poor DNA sequences)	MPPNPPQTPLRRLSQGSLFRLSRSGAFPDAPHGLGFIE<br>PALAELIDEAEALQTNVEGLRNLSDALGTFNESFASWL<br>YVMNMNALTTDWPQAPTQASFR...EAMRAAEAAARRP<br>PTPPEPTRTIERSELPTGDGPTESTIAATSAAAQGAKP<br>GAVKKKVAAKPKMTAKEKRERALEVDRFVSALPLEFRG<br>NDPTLRRHMEMVIEKFLDRPDEGYNIVAFVKPPDLNQA<br>RVNKCLIALVNRKIVRKDNSTGSVLYHWNGLPN*
Phl5	scf184857:18939-19044	MDAFFTIAPAIPVAAEEPIESVPVDADGVGTTGTHGTC<br>TIA*
Phl3	scf184836:160112-160217	MDAFFTIATPVPVEEPANDVEDVLIDADAIGTPGTHGS<br>CVIA*
Phl4	scf184836:170205-170342	MDVFFVIAPPVPTEDPASSSDSTDILFADQDRIGTPGT<br>HGSCVIA*
Phl1	scf184836:173829-173934	MDAFFTIAPAVPETVEDQPLETILVETDRLGTAGTHGT<br>CIIA*
Ste3.1<br>(3805)	scf184836:178191-176393	MADPTYPLFPIFAFLGFILALVPFPWHLQAWNAGTCIF<br>MVWASLASLIQFVDSIFWHDNAINKAPIWCDISTKFIV<br>GAGVGIPAASACISRRLYTIASTSYVTVSARERRRAIY<br>IDVTIGVGIPVVVMLLHYVVQGHRFNILGDIGCVPTIF<br>NTPLAYPLVYMWPPLLGCVSFVYSALTLRAFWVRRMQF<br>RDVVCKSSSMTLSRYFRLLLLASIDMACNVPLTVLNMY<br>INNDGVTMSPWVSWANTHYNFSHVEQIPSILWRTNRAF<br>VVSAELGRWIFPCCALIFFALFGFAEEARKHYSALFWS<br>IAKKLGFSKPEPSAVKQKIFSFVRASNNTHTIDHDVLP<br>PYSLPKPVHYNNLDSFATSGGSLHDIDLEKAGISPTFT<br>NTSFSSDDSHLSPFGQEFALSPLSPDVRPSSEDTQAEH<br>RIVISDEHPPSRPPTPSLVVGNAAPSIPAFHRPFSPPS<br>ICPVSSLDEECRGSILITVHTEKTDTH*
Ph4	scf184836:180543-180394	MQPRRPDNLNDDFGLIISVPLEEGGSAPTEGVPVDEDS<br>PSRAGVYCIIA*
Ph3	scf184836:182175-182280	MDEFFTIPFPPTDEGIDFGETPFDCPVDAERYGAGQTS<br>YFCVVA*
Ph1	scf184836:184363-184468	MDDFSLITVDTLATIDSLPVDAPSAHSSSTLSSSRAHD<br>GFAHACTSLPVNEDHMNGSYGGYCIIA*
Ste3.2	scf184836:187293-185714	MDPSYPAFPILSFLGFVLVLVPLPWHLQAWNSGTCLFI<br>FWTAIASLNLFVNSVVWHGNALNTAPIWCDISSRITIA<br>GTVAIPAASLCINRRLYQIASIRTVSLTRADKRRAVIV<br>DICIGLGIPTLAIIMSYVVSGHRFDIYEDIGCQPAIYT<br>TPFTFPLLLCWPIVIGLVSGVYCAMSLYAFNKRRAQFV<br>EFMSAHSALTITRYFRLMAIAMTDLLCSVPISAYGIYL<br>NIVDGGINPWISWDDTHFQFWRVDQVPAAFWRASRDTV<br>ISFELARWLIPFCAFMFFAFFGFAAEARAYYARLLNKV<br>LNLLGVTRADKLPHSLFGSKAGQSLSSSSKSSHALPTF<br>CPRPPPPPPFTSLSFGSESNASYDLDKASSIESGAPPYE<br>VRLGELPDTSSTSCSEPSTPCGHHAA*
Ph2	scf184836:188931-189036	MDQFDIDLDFLSPASLSSASSSQYPCIAPVIPTNGDTP<br>EPSHDQPPRDYDHRSTGFSAVCIIA*

Ste3.3	scf184788:63889-62023<br>(2 introns at 3'end of gene included as compared to genes in the other species)	MHAELPIISFLCIPTLLIVAPAFASSRNLPVLSLAAWL<br>LCCNLIHGINTLVWAGNSAAHIPAWCDIMTRVLMAAQL<br>ALPGSALALVMRLRRCALGQETTGRRSSVSTDVILCLA<br>LPIVYIITHIIVQPHRFDIAADYGCVASIYTSTVSIIL<br>IWIPPLLFCIGTLVYTFLAIRARLGSGLFFFSHMQDAP<br>RMSALAFIRPLVISVSISLISMSTTIFSVTAHLVSVGG<br>LQAWATESWAQVHAETSQVFVIPATSRIILQSLAADWW<br>AVPAYTFVFVSMTGLALIHPEGSKTYGSVRRWLRRVIL<br>RRASEDALAHAKSFGGQTLCSSPSSPTSMYEMKAGWED<br>TWRLSAPAKVKLPPLTIPAGPSETTIAGSNPEQDDTFA<br>RSTMRYVESPTGREALGLPPMPPAIYHPAQRGGSVSPP<br>VTLPRSPSPPKSRIAPRDEDEAPSARPDSMILSAGAWP<br>RPPSTIPTTPTPLASSTISVGAYAYEEPHTAPFVDSVS<br>DVAPGPGLAVPKHIRKVRSREVLFSRSLSVSSRGRRDG<br>SSGGLSDGIYMTVVKETE*
Ste3.4<br>(2712)	scf184788:41546-43176	MSIPWLVQVDIVAAFSLLGFILITIPLYWHLEAWNVGC<br>VLYIFWIGTQSLFQFINSVVWRDNAINWAPVWCDITTH<br>FTIGSSIGVCCASLVINRRLYHIANISTVSISRSDKRR<br>NMVTDLAIGVGIPIVSMCLYWVYQGHRFDILEGVGCTE<br>EFPNSFLAYLLYFTWPIVIGLVSATYCILTLRAFFKRR<br>RDFSTLVASNNNLTFNRYFRLMGLAAIEVLFTIPLSTY<br>NIVENARTPIYPFRGFADLHFWFSRVDQRSALSWRSEP<br>ATVAAVHFKQWTVIGCALLFFLFFGMAEEARKHYRLAL<br>SSVAKRVGLSTFEKGTGFSSTGSGFKPTTSGFGRITIP<br>TFVQRSTRRGSMDSFSDRLSTNISISDAIDEKGAATYS<br>PVESSAGSSTFIASPVDTEKGVSPQESLPPVSLPPVSF<br>PPPPRAYDPESPTRREADVPASVRPHSIDMV*
***STE20* locus**		
Phl2	scf184858:142687-142792	MDAFFTIAAPIPAEEPTTEVLVDRDDTGTSGTHGTCVI<br>A*
Ste20	scf184858:138747-1441164<br>(frameshift at the 5'end requires introduction of an extra intron for full length protein production; part of aa sequence is missing by poor DNA sequences at 3'end)	MPQGSRLLKKASSSSSSSDLGRGHQPKQLSKSPPPSPG<br>SERKSRPEPWPYVPSSNPSAQYSLLEKLGTGSFGTVYK<br>AIHNDTKQIVAIKQIDLEDTDDDISEIQQEIANLAQCD<br>YRLWIVMEYLAGGSCLDLLKAGPFSEAHIAVICRELLL<br>GLDYLHSEGTIHRDIKAANVLLSASGKVKLADFGVAAQ<br>LTSTLRHTFVGTPFWMAPEVIRQAGYDAKADIWSLGIT<br>AIEMAKGEPPLAEYHPMRVLFLIPKAKPPVLEGPFPMA<br>FKDFVAQCLTKDPHSRPTTKELLQHRFIKNARKTTYLT<br>ELIERYQDYRNRSPGRGPQMYQATVRNSGAWDGTLRSE<br>WDFNTIRSTSAMGSLRSMAKDIMPPGMELDDQYYDEVP<br>EEDHSAYEGQGSIDTTAATTKGSDVPLRSPINGLGMNA<br>DAGHSTVVIRSYAPSGNQKDAPSLMSDNGSDETASPAI<br>ITPLQSVENVEAQVADAIEPPPAYSGSVRSNRRASYAM<br>RSNLASGTVLSEADIGTGVDTIRPVKKVDAVRSLRLSE<br>EYVGSIRSREGSVSSAPPSPQSSKGSHKRGTSEFAKAG<br>KSIVEDVMLPLLQKATRDDMDAREIESLSMISRGFEEL<br>GAVNPELAYNLVLDMLSGINENSAVRN...EGLKLKWP<br>SIL*
***Pycnoporus coccineus* BRFM 310**		
***A* mating type locus**		
Mip1<br>(1366177)	scf16:282559-280065	MLAKAAKHAFSRPTTVLPRIRSCLVAESRRHARVISRS<br>ATTSTIQPASVDDTNLIAFFDYPQAPQNVTTPTGIFGH<br>RALTTPAAFIALADSTLRRAQLLTERILRARESRDELF<br>KVVKNLDRLSDMLCGVIDLAELLRNAHPDPAWVQSADD<br>VYEKMCEFMNVLNTNVGLYEVLGVVLADHEIVKSLSPE<br>AYQTALIFWRDFEKSGIHLPPAQRERFVSLSTEILVLG

		RQFLNETAAPRPPARIKFSELQGVKDLGMGARLRLQAQ VTKRDLLVYPGSLQAQMIMRSAPAEEPRKKVYIASNSS TPEQIETLERLLRARGELARLVGKESYAHMTLTDKMAK SPENVQRFLDALMDHTRPYARRALRNLSMRKQAHLNTP PLPVIQPWDRDYYCPPEPPAPPVSLPPLTLGTVFMALS RLFKALYGISLHLSEPSPGEVWHSDVRKLEVVDEEQGV LGWIYADLFARSGKPGGAAHYTVRCSRRTDDDDEEGDF RHAEDSDRVLAELSTVFEAERRMRLPRQNVTFQLPVVV LLCEFLRPSIGRGPTVLEWHEVLTLFHEMGHAMHSMIG RTEYQNVSGTRCPTDFVELPSILMEHFLNSPAVLALFD RDGTHAIRQNGAHHEDPCRAIDTHTQILLAMLDQRYHS PDVLSSNFDSTRALAELSTTRGLIPYVPGTSWQTQFGH LFGYGATYYSYLFDRAIASRVWRVLFSDNPLDRQGGEH YKQTVLKHGGGKDPWVMVGSLLRSSVLSVGNADAMAEV GKWRIEDEVAYGGQG*
HD2 (1467109)	scf16:277793-279634	MPLTNQFGADRVALRRIFLAARRSVEKRAEYSPSVTCE SPALDIPALSLPVPESLVPRLLQRGVDYVSAETISGIL CRALLRLKNMFEANYKLRCQRLRSEAAFFHDTTFLANL LSAYSIPYHKATRHWTAYIVEDYIPRLLRARSEYKRRS LNPKGSVQARPAFNHSAIPTLEQFFSKNPFPSRLEKFE LASTCKMEYRQIHVWFQNRRSRLRKEGKELKKPERKGV LPEEVEHRVTEIFFPSEQGEEADDDDDDDDTSLGGSPS TLSPSRPSTLLNVPAPAHAYPAPYPPVCAEDPFPLDSR RPPFELPWLRTPAPQSAVPKPSVDLDSLVSLLSKMTLV DHHTEVASTPPFASGCKATTPCAIGFVTPCVRAPHPAL VRKSRGVPRSTWCSPMLLSGPHIVASNLFPAPEPQRSS CADAPPPSTLAAADGVPSVRRKRALPRRVPKHPPSSRF ALDMHNTERPGPQFTQDARRISSLTSSASSSSGSDVES ALPTPELSTSELPPFKEFLSFTSIPDDFSWLADSASLP WDPLGFDVDTKPRSVAVQPSTHVVF*
HD1 (1434663)	scf16:277593-276124	MALPPPTMSSLKQRLLSADRDFVSALGHGIDALVAFDR SWERLIDDVDAAFRTTGLDDDICALVHATATRIANLAD SSDSQLAYCKSFTAELVDQIQDIMSNLTLTDSSDPTQT SVRTSLETSTIPNAPNTRKRQRTPSSEYDEYDGAILSS KRCRITSPALEDKCQKFPAGLACSIISNSGRHVAASTT PSSSVASNRKRRFSESEPVEPVPPCKRRYIGPRLHAVS DSFIAPRLNETKTQATATSLLHVDPDLDKSPISWLVDE LAEQSSNLPSSDPINSSSNANSGGLELYDAVPNINGAS LSLPNLDNLDAFLETIFLPHDSIVVPTSLAGHSTPSVP SRALRGSPSSSCSSDSDSGSSPASSTPSTPRFSPATPS LDSDILKFDLGASQYFPLNDEWAPGDELQLSSFFDVVP PLPEVKWPSSSIDSSWLDVSRLVSPSLDEDLDSLL*
β-fg (1434662)	scf16:274641-275589	MDSFMNLAKQGMQAYERSQSDVSRTGGHEYNSPDHTRS TQDRPQFDDDEVVNTASREGSGDTSLFSSALSFIKQNK SAHEEPIDEEGVQHAHRKAYEEGSAGSLSAGSLGSAAA MQVLKKFTSSGSSGGGSQSQLISLAMAEASKLFDSAGG ASSGNKQDAVNGAAMTVMKLLVQSKFSGAVGGGNSGGL SGLMGLASKFM*
***B* mating type locus**		
1434136 (Dam1)	scf14:287472-288558	MPPNPPQTPLRRLSQGSLFRLSRSGAYPDAPHGLGFLE PALAELIDEAEALQTNVEGLRSLGDALGAFNESFASWL YVMNMNALTTDWPQAPTQASFMLAARRAEEDARAALEA MRAAEAAARRPPTPPEPTRTLERSDLATGDSVTESTFT AAPSASAAASGPAPGAKPGVVKKKVVGKPKMSAREKRE RALEVERLLSALPLEFRGNDPTLRRHMEMVVEKFLDRP EEGYNIVAFVKPPDLNQARVNKCLIALVNRKIVRKDNS TGSVLYHWNGLPQ*

1548828 (Phl)	scf14:289404-289280	MDAFFTIASAIPVSVEEPVESVPIDADAVGTNGTHGSC IVA*
1548827 (Phl)	scf14:301538-301666	MDAFFTIAAPVPVEEPAVGVEDVLVDNDAIGTNGTHGS CVIA*
1548826 (Phl)	scf14:306745-306611	MDAFFVIAAPVPAEDPASSPDGDILFADQDRLGTLGTH GTCVIA*
1548825 (Phl)	scf14:308529-308704	MLDAFFTIAPADPVEAPADVPMELDSTGTNGYYDFCTI G*
1548824 (Phl)	scf14:310044-310172	MDAFFTIAPAVPETVEDQPLETILVETDRLGTNGTHGS CIIA*
1434140 (Ste3)	scf14:313043-311212	MIFPLAPQPDPTYPLFPVFAFLGFLLALVPLPWHLQAW NAGTCMYMLWASLASLVEFVDSIVWNGSLKDAAPVWCD ISTKFLIGAGVGIPASSLCIARRLYKITSVSTVSVTRK EKLRSVYIDVAIAIGIPVIVMALHYIVQGHRYNIIENV GCTPDIWNTAPAYPLVFMWPVLLGCITFVYAALTLRTF YMHRVRFNQVLSSNTSLTVSRYLRLILLCCVEMALVMP LGAFSIYINTAGLHIARWVSWSNTHYNFSFVELFPTYV WQAKLASHVAIEMGRWIYPCSAILFFMLFGFAEEARRC YIGAFWKAAKAFGISPKPTSGKGRMKGLEGAFKMPDGS TGDILPPYTPPAHVRKKRADSLTSSLATTLDHFDVEKG LRPPSMHVLSHLVSPADSEFGSHSDMQLSPVETSAFSH GAAAGHSESDVASEAHISISDVHPASRPTTPPAQLRAV SPTIPEFHRPFSPPITWPRATLAPSRKASAGSISIMVH TESRTY*
1548831 (Ph)	scf14:314292-314139	MDAFSSITDFLESEAARTADAPASPSTESGVPVNFEYV NNNYSHSWCTIA*
1365351 (Ste3)	scf14:317702-316200	MAYPILPISAFIGAVLVLVPVPSHWRARNYATVSLVAW LFILDVIYGINSIVWRDNVEIKLLVWCDITTKLTIGAS VALPAAAMCICKHLELVASGRVVRVSHADKRRRMYFDL AMCYSLPAIIMALHYIVQGHRFDIVEAFGCQPATYYSI PGVFIVWFPPLLLAVISMVYAGLALYHFLRHRVTFATV LQNSNSSITPNRYLRLMALAVTEIIWQITLTVLPMYDN ISAGLRPWTTWADVHSDWLRVDRYLLVEFVPAYRQQLF LVFFAIPASSLLFFIFFGFGEQAVRDYRHTFDWVRAKV FRQKNNMQTKGMMMWYVSLRSAAPYVSLILLVVFQLIR KRFLTFALRHPALRLRRRKQPPGLQPSVSSRRIPNVLQ RRGQEGVGRPPLQRFVSARCRSGHARSGRPQSHRDRSI HLLPLRSRARYHGTPRRSSYRSPYRP*
1548835 (Ph)	scf14:318348-318404	MDEFTAFSTSVADIGLVEPRELTTGGECPVDSERYGAG QTSFYCVIA*
1548834 (Ph)	scf14:319912-320114	MDEFSFLTVTSDDLVDQRALDDTSTHTSMSFSSSAQRR DALFAAVASSTPVNEDRIEGYNGYCVIA*
1434144 (Ph)	scf14:321520-321319	MDDFENVDWFVEPEGLAPVASSRTYDEPMTYDVPIDAE HQPTTSHFWCIIA*
1388054 (Ste3)	scf14:322025-323885	MSGAPSDPTYPAFPVLAILGAVVALVPLPWHFQAWNAG TCLFMIWTSIACLNLAINAIVWSNNALNPAPVWCDISS RIIVATGVALPAASLCIQRRLYNIATIKTVSITRAEKR KGILIDLAIGVGIPLLQILFQYIVSGHRFDIYEGIGCY PYTYNTPLAYPFSLVWPLVIALVSACYCVLTFAAFLRR RAHFNAYLASNTSLTANRYFRLMGIASVEIVCTVPISA YGLYLNLTAAPVSPWISWADTHFNYSKVDQFPAALWRM DRTAVVSFELSRWLAPFCAFVFFGFFGFAQEARSSYRK AFLWVCHLLPFRTLFPERPPKGAVSIEKAAGWHTKQDM DTLPQYSVRPPRPPPLTSFTSSARTEDTLSMSDTPYYE KFPETPSTADSDLSHDGLPHHGPLTV*
1548833 (Ph)	scf14:325235-325411	MDEFDTYFFTLSQSSASTTSGPIAPVLPNAVDGLELSD ELPRDYDHRSNGFGAVCIIA*

1434147<br>(Ste3)	scf14:331415-333277	MRAELPTLSFLCVAALLLVAPVYASSRNLPVLSLAAWL<br>ICCNLIHGINAIVWAGNSIDHIPAWCDIVTRVLLAAQL<br>ALPGSALALVMKLRRCALGRETTGKRSPVAMDVILCLA<br>LPIIYIITHVIVQPHRFDIAADFGCVASLYTSSVTIIL<br>IWIPPLLLCITTFVYTSLAIRARLESGLFFFSHMQDAP<br>RMSMLAFIRSLVISVSITLISLSMTIFSMTAHLISVGG<br>LQAWATDSWAQVHAEMSQIFVVPTSSRLTLKSIEADWW<br>AIPAYTLVFVAMTGLAFVHPEGFTPYGRVQRWIHRTVL<br>RRSPEDSFSQAKSFGGQTLCSSPSSPTSMYEMKAGWDD<br>TWRPSAPAKVKLPPLVIPAAPSETTIAVAEAEQDDRFA<br>RSTMRYVESPTGREALGLPPIPPAIYEPGRRNDSVSPP<br>VTPPRAGSPPKPSMTLQDGETAQARPDSMILSGAWPRP<br>PSTIPASPRTPSPKTPLMVTPPSPLPPHSPSSTPRPPS<br>IMSVTTSIASSTISLGGYAYEEPYVAPFQDRVSDAPGP<br>GLAVPKHIRKIRSRDMLFPKSLSVSSRGRRNGSNGGVS<br>EGIYMTVVKETE*
1365397<br>(Ste3)	scf14:341986-343643	MSIPWLVQVDIVAAFSLLGFLLVSIPLYWHLEAWNVGC<br>VLYIFWIGTQSLFQFINVMVWRDNAINWAPVWCDITTH<br>FTIGSSIGVCCASLVINRRLYHIANISTVSISRADKRR<br>NIITDLAIGLGIPILAIALYWFYQGHRFDILEGVGCTE<br>EYPNSFLAYLLYITWPIPIGLVSATYCILTLRAFFKRR<br>RDFSTLVASNNNLTFNRYFRLMGLAAIEVLCTIPLATY<br>NIVQNLTTIPIYPFIGYADLHFWFSRVDQISAVSWRAY<br>PATVAAVAFKQWTVIGCAILFFLFFGLAEEARKHYRLA<br>ISSVAKRVGLTTFEKGTGFSSTGYPTTSGFGKITIPTF<br>VQRSTRRGSMDSFSDRLSTNISISDVVDEKAAYSPVDG<br>SGSSAGGSSTCIASPVDTEKGISPVESLPPVSLLPVSF<br>PPPPRAYAPQSPTRRSADVPASVRPDSIDMV*
***STE20* locus**		
1548837<br>(Phl)	scf12:328927-329047	MDAFFTIAPPVPVDEPTTEVFIDRDSVGTNGDHGSCII<br>A*
1548839<br>(Ste20)	scf12:324921-327352	MPQGSRLLKKASSSSSSSDLGRGYQPKQLSKSPPPSPG<br>SERKARPEPWPYVPSSNPAAQYSLLEKLGTGSFGTVYK<br>AIHNDTKQIVAIKQIDLEDTDDDISEIQQEIANLAQCD<br>SEYVTRYYGSFVVAYKLWIVMEYLAGGSCLDLLKAGPF<br>SEAHIAVICRELLLGLDYLHSEGTIHRDIKAANVLLSA<br>SGKVKLADFGVAAQLTSTLRHTFVGTPFWMAPEVIRQA<br>GYDAKADIWSLGITAIEMAKGEPPLAEYHPMRVLFLIP<br>KAKPPVLEGQFSMAFKDFVAQCLTKDPHSRPTTKELLQ<br>HRFIKNARKTSYLTELIERYQDYRNRSPGRGPQMYQAT<br>VRNSGAWDGTLRSEWDFNTIRSTSAMGSLRSMAKDIMP<br>PGMEPDDQYYDEVPEEDESAYEAQSSIDTAAATTKGSD<br>VPLQPQINGIGMNADAGHSTVVIRSYAPSGDQKDTPSL<br>MSDNGSDETTGPAVVTPQQSVEDVEAQVADATEPPPAY<br>SGSVRSNRRASYAMRNNVSSGTVLSEADIGTGVDTIRP<br>VKKVDAVRSLRLSEEYVGSLRSREGSVSSVPSSPQSSK<br>GSHKRGTSEFAKAGKAIVEDVMLPLLQKATRDDMDARE<br>IESLSMISRGFEELGAVNPELAYNLVLDMLSGINENSS<br>IRNHIQTSRSLFPHKRIIRRSEMTAKGLVVTEEEEISG<br>LPTPSTSSPAQPQAEPGSPVRKSPISELLYLRWLEGLK<br>LKWPSIL*
***Pycnoporus puniceus* BRFM 1868**		
***A* mating type locus**		
Mip1<br>(437979)	Scf7:289087-286604	MLAKAAKHVLSRPTTLRLHFRGSINADRRWGKALVSRS<br>ATTSAIQPASVDDRDLIAFFDYPQAPQKVTTPTGLFGH<br>RALTTPAAFNALADSTLRRAQLLTERVLRARESRDELF<br>KVVKNLDRLSDMLCGVIDLAELLRNAHPDPVWVQTADD

		VYEKLCEFMNVLNTNVGLYEVLGTVLRDREIVKSLSSE<br>AYQTALIFWRDFEKSGIHLPPAQRDRFVSLSTEILVLG<br>RQFLNEAAAPRPPARIRFSELQGVKDLGMGARLRLQAQ<br>VTKRDLLVYPGSLQAQMIMRSAPAEEPRRKLYMAANSS<br>TYEQIETLERLLRARGELARLVGRESYAHMVLADKMAK<br>SPENVRLFLDALMDRTRPYARRALRTLSMRKQADLKTP<br>PFPIIQPWDRDYYCPPEPPSPPVSLPPLTLGTVFMALS<br>RLFRSLFGVSLRISEPSLGEVWHRDVRKLEVIDEQRGL<br>LGSIYADLFARSGKPGGAAHYTVRCSRRTDDDDEEGDM<br>IYSDEHLRAAVQLSRDFEEERRVQNRCRGVTLQLPMVV<br>LLCEFLRPTTGRGPAVLGWHEVFTLFHEMGHAMHSMIG<br>QTEYQNVSGTRCATDFVELPSILMEHFLSSSAILSLFD<br>RDSTSSVRYSLIHHEDPCCSIDTHTQILLAMLDQIYHS<br>PSVLTPQFDSTETLATLHEARSLIPYVAGTSWQTQFGH<br>LFGYGATYYSYLFDRAIASRVWRRLFRHDPLNRDMGER<br>YMQEVLRHGGGKDAWKMVGALLDSLQLANGDAGAMAEV<br>GKWRIEDDIAVPGRH*
HD2<br>(300860)	scf7:284511-286367	MSTSTNMFRQAEVDTLRHTYGRAKALARMVARKPDSSS<br>NLQCAVALSDLSLPIPKPLTSRFMSLGVDERSAALIAS<br>TLARVITSYRNSCEADYRRRRAALQGGPGTPATLPALY<br>IAVYTQAVNDWSRYLLEDIIPRVIQAGKRRKAGETKSS<br>GVQQARRPFNQNAVPLLERFFASNAFPSRLEKYELALT<br>CSMDYRQIHVWFQNRRSRCRKESRTLEKREASNGPRQI<br>LEQTVIDTLLPRESWEEDHQNTVDGGTTCLGGCKQLNR<br>LLSPVAPPHAFPSPYPPVCSYDPFPAAEGRRSFHTPWM<br>RTNRTSNPPRPSTSISDLASWLAKLSISDNGHEATQQP<br>YTIADSLRVCETSQRVLGFVTPCAPAPHPALIQRDRGR<br>PRRHHSAAHRMPTKDHPNISLRPVQLPDITWAAMRSDL<br>QPDTTSCPTSSSALSRPPQQNAKRILPRRAPKHPPRSH<br>ILDKDPLSSGGPPAKRRPRHSFASTSSIGSACSSDTDS<br>PLATPPTPPIALPLTPETYFSQGIGAAKSPEMSQTASL<br>TPISLHLCEPLCGAVDLNSAFSSSHDFD*
HD1<br>(483661)	scf7:28457-282575	MSHFKERLRTIEDDFLSALSEGGDALVSFENRWDNLYK<br>EIDAAIESTSIDAETLAFAHATALRCATLADLSIETFT<br>SCEAIGETLMNELETIISELHVSADYSSDPSTSRSSVP<br>CSALSGTEPKRGRAAMDEVQFLQRSSKRRRLSVRPRAA<br>RGPTAPLALSTRPSLVHLTDMSATSTAARAETPGREGS<br>SKRKRCASDAELASRSSPSKRRYIGPRLQAVSDSFPVS<br>LSKRSVPSLAASCSAEFSSDLAPSSAAADVRTASRPSI<br>FETESKDQLLNLQDSLYRLPWTEDDILNNACPPREDPD<br>ALSTLEELNELLRFVQERVLPARVDSNPSQSLTPDPLM<br>LGYLPPSTRPLTCSTDEDADAATLSSSSSASSSSPSSP<br>SPLTPPDQYLELQLCDSPQGEVAGLSWIEGSLFSDIYS<br>SSCQPFESALRNPAMWESLAPSLDIARPSVEGIDHWPF<br>PAPPYLSFLLPAIGGLSTSCGDVVHAPAGEEHPCMGQS<br>LDAYLSSPWRCLDKSPSVVTIHESIPTS*
**Duplicated while likely non-functional *A* mating type locus**		
Mip1^{trun}<br>(582179)	scf78:11202-8725	Protein truncated by frameshift:<br>MLAKAAKHVLSRPTTLRLHFRGSINADSGGKALVSRSS<br>PLAIQPASVDDRDLIAFFDYPQAPQKVTTPTGLFGHRA<br>LTTPAAFNALADSTLRRAQLLTERVLRARESRDELFKV<br>VKNLDRLSDMLCGVIDLAELLRNAHPDPVWVQTADDVY<br>EKLCEFMNVLNTNVGLYEVLGTVLRDREIVKSLSSEAY<br>QTALIFWRDFEKSGIHLPPAQRDRFVSLSTEILVLGRQ<br>FLNEAAAPRPPARIRFSELQGVKDLGMGARLRLQAQVT<br>KRDLLVYPGSLQAQMIMRSAPAEEPRRKLYMAANSSTY<br>EQIETLERLLRARGELARLVGRESYAHMVLADKMAKSP<br>ENVRLFLDALMDRTRPYARRALRTLSMRKQADLKTPPF

		PIIQPWDRDYYCPPEPPSPQCPYLLSPWVLSSWRSRDS<br>SDHSLVCPCGSQNHP*
HD2^{def}<br>(311548)	scf78:6745-8258	MVARKPDSSSNLQCAVALSDLSLPIPKPLTSRFMSLGV<br>DERSAALIASTLARVITSYRNSCEADYRRRRAALQGGL<br>VRQRRCLRYTSRSIRKPSTIGLVTSSRTSFHGLFKLES<br>VGRPAKRSHLAFSRLGGLQSECCPLLERFFASNAFPSR<br>LEKYELALTCSMDYRQIHVWNRRSRCRKESRTLEKREA<br>SNGPRQILEQTVIDTLLRVSPGRKTIKIRRWRHYLLGG<br>CKQLNRLLSPVAPPHAFPSPYPRSVHMIPSPLPKAGGP<br>STHVDANEPHIKPARPSTSISDLASWLAKLSISDNGHE<br>ATQQPIHHCGLSQSMRNIPRVLGFVTPCAPAPHPALIQ<br>RDRGRPRRHHSAAHRMPTKDHPNISLRPVQLPDITWAA<br>MRSDLQPDTTSCPTSSSALSRPPQQNAKRILPRRAPKH<br>PPRSHILDKDPLSSGGPPRSDAPGIPSRLRHP*
HD1^{def}<br>(582180)	scf78:6460-5133	MVARPPTPQFPTLRLNSSLVTHVSLQGRLRTIEMTSCP<br>LSRKVAMLSSPSKTAGQPLQGDRRGYIESRSLRHSPSP<br>TQPRCVARRSLISPSRRSPAAKLSEKRLNELETIISEL<br>HVSADYSSDPSTSRSSVPCSALSGTEPKRGRAAMTKFN<br>FCNGPPSGGGVTVELSVRPRAARGPTAPWLFQRVLRWS<br>ISQTCATSTAARAETPGREGSSKRKRCASDAELASRSS<br>PSKRRYIGPRLQAVSDSFPVSLSKRSVPSLAASCSAEF<br>SSDLAPSVLLRTYAQRRDHLYSKPNRRINSSICRTRYT<br>VCHVRTEDDILNNACPPREDPDALSTLEELNELLRFVQ<br>ERVLPARVDSNPSQSLTPDPLMLGYLPPSTRPLTCSTD<br>EDADAATLSSSSSHPLLLHPLLSPPLRLTNT*
**Locus of translocated *β-fg***		
β-fg<br>(582165)	scf16:709545-710502	MDNFINLAKQGVEAYERSHSDVSRTGGQEYNSPHHSTS<br>AYDRPQLDDEEVVNTASREGSGDHSLFSSALNFVKQNK<br>SEHEQPIDEEGVQHAHRKAYEEDSASSLSAGSLGSAAA<br>LQVLKRFTSSGGGGSQSQLLSLAMAEASNLFDKSGGAS<br>SGNKQDAVNGAAMTVMKLLVQSKFSGAIGGGDSGGLSG<br>LMSMASKFM*
***B* mating type locus**		
581213<br>(Dam1)	scf31:25573-26627	MPPNPPQTPLRRLSQGSLFRLSRSGAYPDAPHGLGFLE<br>PALAELIDEAEALQTNVEGLRGLGDALSTFNESFASWL<br>YVMNMNALTTDWPQAPTQASFVLAARRAEEDARAALEA<br>MRAAEAAARRPPTPPEQTRTVERSELPTADGPTESTIA<br>TATSAPGAKTGAVVKKKVAAKPKMSAKEKRERGLEMER<br>LGNDPALRRHMEMVIEKFLDKPEEGFNIVAFVKPPDLN<br>QARVNKCLIALVNRKIVRKDNSTGSLLYHWNGLPQ*
582166<br>(Phl)	scf31:27766-27461	MDAFFTIAPAVPVSVEQPVEDVPMDSDDVGTNGSHSTC<br>TIA*
582173<br>(Phl)	scf31:42044-41907	MDAFFVIASAVPAEDPASSSEDDILFADQDRLSTNGDH<br>AAVCIIS*
582176<br>(Phl)	scf31:44004-44121	MDAFFTIAPAVPVEDPVEVPKELDNPGTNGDHGNCTIA<br>*
582170<br>(Phl)	scf31:45855-45983	MDAFFTIAPAVPEPAEEQPVETILVEADRVGTNGNFSG<br>CVIA*
582172<br>(Phl)	scf57:33350-33478	MDAFFTISPAVPEPAEEQPVETILVEADRVGTNGSHEG<br>CVIA*
503832<br>(Ste3)	scf57:36017-34203	MLSPLVSQPDPTYPLFPIFAFFGFILALVPLPWHFQAW<br>NAGTCIYMLWASLASLVEFVDSIVWNGSLKDVAPVWCD<br>ISTKFLIGAGVGIPASSLCIARRLYKITSVSTVSITRK<br>EKLRSVYIDIAIAIGVPILVMVLHYVVQGHRYNIIENV<br>GCTPDIWNTVPAYPLVFMWPVLLGCITFVYSALTLRTF<br>YIHRVRFNQVLSSNTSLTVSRYLRLILLCCVEMALVTP

		LGAFSIYINTAGLHIARWVSWANTHYDFSFVELFPTAV<br>WQAKLASHVAIEMGRWIYPCSALLFFMLFGFAEEARRC<br>YVGAFWRAATVFGISPKPAAGNDKLNGFKRGFQAPTSP<br>SGDTLPPYTPPAHTRKKRTDSLTSSLAPTLDHIDVEKG<br>LRPPSTHVLSCLSNPADSEFGCHSDIQLSPLAPSSVFP<br>PDEAHPASDSESEAHISISDVHPASRPPTRESHPQPVA<br>PSVPAIHRPFSPSIAWPLSTLTVPRKASAGSISIMIHT<br>ESRTY*
582154<br>(Ph)	scf57:37157-37005	MDAFSSIPEFLESDAARTADAPASPSTESGVPVNFEYI<br>NNNYSHSWCTIA*
582153<br>(Ph)	scf57:38271-38399	MDEFDFTTITLAPERPLDDTVYGHIPVNEDSPSRAGVY<br>CVIA*
450535<br>(Ste3)	scf57:42283-40534	MVDPTYPLFPIFAFVGVVLAIIPLPWHLEAWNSATCYY<br>MMWASLSCLNEFVNCVVWAHDAVDRAPIWCEFSTRVTI<br>ATSVGIPAAAMCINQRLYSISRVQAVMITRAEKRRAVL<br>LDTFVCVLFPIVIVALSYVVQGHRYNILEELGCFPALY<br>NTLLTYFLVNWWPLVLGLIASVYCVLSLLEFNRRRAQF<br>NEFLSSNKSSLTLGRYFRLMALSTTSLLLMIPVSSYGI<br>YLNVTAQPLGPWVSWSDTHFDFERIEQIAAVAWRSSRT<br>SVVVHELNRWLSPACAFIFFLYFGVASEARRNYYKAFW<br>YVLGKFGVMPATRNEKSGLQSLRNMPTPAKFLPSSESL<br>PPYSYPKPPRMEQGPLNPGSYSSSIRSFHDEKDLSTYR<br>MLESTTTLAAGSTTPIYDRFSRRDPADFPQDAVPPLPF<br>SHSLNVSPLSPTDITVRSPTDSVFSYNSEQSAVSLSYT<br>DIESAYGARVPSFVSLRVEHSSEPPTPLNLHHVTA*
582150<br>(Ph)	scf57:43140-43339	MDEFSIFISSSEDVFRGVSAADSTSGSSPPHSSSQGRD<br>VLVARVAYSPPVNEDRIEGYNGYCVIA*
582147<br>(Ph)	scf6:451689-451844	MDNFDIIDLFIEPEGLAPMLSSSHDELHLFGDVPLDAE<br>HEPTTSHFWCTIA*
582157<br>(Ste3)	scf6:450991-449538	MPGAPSDPTYPAFPVLAILGAVMVLVPLPWHFEAWNAG<br>TCLFMIWTSIACFNLAINAMVWNNNVINSAPVWCDISS<br>RIIVAVGVAIPAASLCIQRRLYNIATIKSVSVSLAEKR<br>RSVWIDLAIGVGIPLLQILFQYIVSGHRFDIYERVGCS<br>PYTYNTPLAYPFSFLWPLVIALISACYCVLTFAAFLRR<br>RAQFNAYLASNTSMTANRYFRLMGIASVEIVCTVPVSA<br>YGLYLNLSSAPMNPWISWSDTHFDYSKVDQFPAALWRM<br>DRNAVISFELSRWLAPFCAFVFFGFFGFAQEARSNYRK<br>ALLWVCNLYAFRRLFPTRLPKGAVSIEKAVGWHSKQDE<br>DVLPVYSVCTPRPLPRSFFMSSTKSEDGLSMSDTPSDE<br>KFPETPSTA*
582146<br>(Ph)	scf6:447855-448206	MNSFSSSHHRSSSPRTAVRIYQWMRITSTTSVWSARLH<br>DEHLVLLCIRAYSHSGPLRCGLQSHLLKASTHHTCIFL<br>VDAISSGFVLSLVNSCVVT*
582144<br>(Ph)	scf6:447851-448034	MDEFLLVIAPPVLEPEDSGADIPVDEDHKYNFSLVCTI<br>A*
582145<br>(Ste3)	scf6:444588-446076	MRAELPYVAFLAAVLVLVPLPWHWRARNVATLSMIAWL<br>FVINVIYGVDAMIWHHKVQITAVVWCDITTKIIIGAGM<br>ALPAACMCISIHLAQVASVSRVRNTKADKRRRQIIELL<br>LCFGIPCIWMALHYTVQGHRFDIIEDYGCRPNTYISIP<br>AIFLIWVPPLIFATVTLVYAGIALMHFLRHRITFARHL<br>ENASSGLTTSRYLRLMAMAFVEIIITAVSSSLTLWFTT<br>LGLRPWTNWADVHWNFSRIDVYVTAFQPPLVNNYYYAI<br>WYIIPVSSVIFFAFFAFGQDAVKEYTACLVWVRDRVFK<br>CGFRKTVRKSKQCDGPFVSLPSSSLVDSSATIHSLPSY<br>QSAVGEDDVQVLSEKGFDNKERYVARPIDRTDIMPIPI<br>HHISAFTADNDSSYSSSLPSTPSHTDTSHGHSYLEAAP<br>CRPIPHDLV*

482390<br>(Ste3)	scf6:435140-433247	MRAELPTVSFFCVAALVLVAPLFASSRNFPVLSLAAWL<br>ICCNIIHGVNTIIWAGNDAIHVPVWCDIVTRILLAAQI<br>AIPGSALALVLRLRRCALGQDTSRGRSPTTPDVMLCLI<br>LPILYMILYIIQQPHRFDIATDFGCVVSIYTSSLSIIF<br>IWIPPLLLCIAIFVFGFLSIRARLGNGLFFFSHMQDAP<br>RLSALAFIRPLVISVSTCLISFAVTIFSMTAHLISVGG<br>LQAWTVETWAQVHAEMSQIFVIPANSTLVLRSIQAEWW<br>TVPAYTFVFVLMTGLAVVSGAHAEGSRTYEALPRWLRR<br>TFLRRTPDDPFAQAKGFGVQTLSSSPSSPTSMYEMKSG<br>WNDSWRPAAPAQVKLPPLTIPSASSESTIAVNEHDDPF<br>ARSTMQYIESPTGREALGLPPLPPALYLPTARNGSVSP<br>PVAPVAPARSPSPPQPRASSPTEDTSQARPDSMVLSGS<br>WPRPPSTIPTSPRTPSPKNPVTVTPPSPAPSSARAPSP<br>TPRPPSIISFTSSVASPTVSMSGYVRDEPHDVPFQDSA<br>ASNALGPGLAVPKHIRKVRSRDVLLPRSLSVSSRAKRN<br>GSDGGLSGGIYMTVVRETE*
287178<br>(Ste3)	scf6:424488-422872	MSVPWLVQVDIVGAMSLLSFILICIPLYWHLEAWNVGC<br>VVYIFWIGTQCLIQGINTLMWRNNAINWAPVWCDITTR<br>FTIGSSIGVCCASLVINRRLYHIASISTVSVTRADKRR<br>NLITDLGVGLGIPVLAVALYWFYQGHRFDILEGIGCIE<br>EYPNTWLAYLLYITWPIPIGLVSATYCTLTLRAFLKRR<br>RDFNTLVASNNNLTFNRYFRLMGLAAIEVIFTIPLTVY<br>NMVMDLTTLPIYKFVGLADLHFWFSRVDQKSAVSWRSD<br>PGTVERMRLRQWLVIGCALLFFLFFGLAEEARKHYRLA<br>LSSVAKRVGITSFEKKSGFSSTGSNFKPNTSGFGRITI<br>PTFVQRSTRRGSMDSFSDRLSTNISISDVIEEKATLSP<br>VGSSGSSTFIASPVDTEKGASPQELLPPVSFPPPPRAY<br>DPESPTRRDADVPASVRPNSIEMV*
***STE20* locus**		
582175<br>(Phl)	scf1:1495340-1495321	MDAFFVIAPPVPVDEPTTEVLIDRDDTGSNGDHTSCTI<br>A*
532784<br>(Ste20)	scf1:1499310-1496870	MPQGSRLLKKASSSSNSSDIGHGFQPKQLSKSPPSSPG<br>GERKARPEPWPYVPSSNPSAQYSLLEKLGTGSFGTVYK<br>AIHNETKQIVAIKQIDLEDSDDDISEIQQEIANLAQCD<br>SEYVTRYYGSFVVAYKLWIVMEYLAGGSCLDLLKAGPF<br>SEAHIAVICRELLLGLDYLHSEGTIHRDIKAANVLLSA<br>SGKVKLADFGVAAQLTSTLRHTFVGTPFWMAPEVIRQA<br>GYDAKADIWSLGITAIEMAKGEPPLAEYHPMRVLFLIP<br>KAKPPVLEGPFSMAFKDFVSQCLTKDPHARPTTKELLQ<br>HRFIKNARKTSYLTELIERYQDYRNRSPGRGPQMYQAT<br>VRNSGAWDGTLRSEWDFNTIRSTSAMGSLRSMAKDIMP<br>PGMELDDQYYDEVPEEDESALEEHGSIDTTAATTKGSD<br>VPLQPPIGLGGLGMNADAGHSTVVIRSYPPAGEEKDTP<br>SLLTDNGSDETAHSPTVVTPLQSVEDVEAQVADADATE<br>PPPAYSGSVRSSRRASYAMRSNVATGTVLSEADIGTGV<br>DTIRPVKKVDAVRSLRLSEEYVGSLRSRESSVSSVPSS<br>PQSARGSHKRTASEAAKAGKAIVEDVMLPLLQKATRDD<br>MDAREIESLSMISRGFEELGAVNPELAYNLVLDMLSGI<br>NENSAVRNHIQTSRGLFPHKRIIRRSEMTAKGLVVTEE<br>EEISGLPSTSTSSPAQAQAEPGSPVRKSPISELLYLRW<br>LEGLKLKWPSIL*
***Pycnoporus sanguineus* BRFM 1264**		
***A* mating type locus**		
Mip1<br>(1646990)	Sc718000065073:252548-250057	MLAKAAKHAFSRPTTLLPRIRRCLFAESQRHPRVISRS<br>ATTSAIQPASVDDTNLIAFFDYPQAPQNVTSPTGIFGH<br>RALTTPSAFIALADSTLRRAQLLTERILKARESRDELF<br>KVVKNLDRLSDMLCGVIDLAELLRNAHPDPMWVQSADD

		VYEKMCEFMNVLNTNVGLYEVLGVVLADQEIVKSLSPE<br>AYQTALIFWRDFEKSGIHLPPAQRERFVSLSTEILVLG<br>RQFLNETAAPRPPARIKFSELQGVKDLGMGARLRLQAQ<br>VTKRDLLIYPGSLQAQMIMRSAPAEEPRKKVYIASNSS<br>TPEQIETLERLLRARGELARLVGKESYAHMTLADKMAK<br>SPENVQHFLDALMDHTRAYARRALRNLSMRKQAHLNTP<br>PFPIIQPWDRDYYCPPEPPAPPVSLPPLTLGTVFMALS<br>RLFQALYGISLRLSEPSPGEVWHSDVRKLEVVDEDQGV<br>LGWIYADLFARAGKPGGAAHYTVRCSRRTDDDDEEGDF<br>RHAKDSDRALAELSTVFEAERRMRLPRQNVTFQLPVVV<br>LLCEFLRPSVGRGPTVLEWHDVLTLFHEMGHAMHSMIG<br>RTEYQNVSGTRCPTDFVELPSILMEHFLNSPAVLALFE<br>RDGTHTIRQNGNHHEDPCRAIDTHTQILLAMLDQKYHS<br>PDVLSSNFDSTRALAELSASRGLIPYVSGTSWQTQFGH<br>LFGYGATYYSYIFDRAIASRVWRVLFSDSPLSRQGGEH<br>YKQTVLKHGGGKDPWGMVGSLLRSSVLSVGNADAMAEV<br>GKWRIEDEVAFGGQGG*
HD2<br>(175610)	scf718000065073:247475-249323	MGGSQAPVDATIFVLAVIMQLKNERIALKPILAAARRL<br>QKLTERTPLSTCKLSAAPAHTLSLPLPESLVPKLLQLG<br>VDPRPAERISQAVTRALLRLKDTLEANFRTRCQRNDSE<br>AALLHDPTFCSTVLSAYVSIYHKAKLNWIAYIVDDCIP<br>RLLRVQSAHWPQPSGSKPPTPARPMFNHSAVPILEDSF<br>SINPFPSRLEKLELASRCRMEYRQIHVWFQNRRSRLRK<br>EGIELKRPERKSALPEEVENIVTEVFFPSDQEEDGDDE<br>ALTMLTALSSASQKSLLLNIPAPAHAFPAPYPPLCADD<br>PFPMDSRGPPFALPWLRTPTVKKSRSSAATDLDTLASM<br>LSKLTLIDHRVELISSTRPSSGCRSASHCAIGFVTLCS<br>RAPHPALVRWPSRGPRSLSTRRQIYGDESLSPKDAVAG<br>AQNGPRSARRRRALPRRIPNHPPTSQPALDAQDVKRLS<br>NHLVRESSRTSSLASCSSSSSGSDVDSPLPTPELSISD<br>LPSLKGHLSSTSAVDDLSWLANSACLPWAQLDFTVDVE<br>PQNTAFLSPTGALSEAWLSGASPLLPPL*
HD1<br>(1775000)	scf718000065073:247322-245859	MSSLKQRLLSAEDEFISALADGDDALVAFADKWERLLE<br>EADVAFHDHCPDSDMGALVYTTSIRIATLAESSSELYA<br>RHESFAAQLTDQLEALMSDLAIFDSSPLSQIPTTLPPC<br>TTSIKSKRRRTSSPERELTYEPTKRRRVIADPPSCGED<br>RKSSPSGDSYTSPATFYPPSPSLPRDTVPSRKRRCSDS<br>EPTSDAPPHKRRYVGPRLHAVSDSFVASRFVGASGQGI<br>TRPLSSNNPNPDQRPIIQPLHMSSGQQSLGSSSPDCAE<br>IERYEPPRLDVCNVLPDLDPATLPLPGLDSLDAFLETI<br>FQTHDSIIPHPPLPRCDTPYSPSHFLQKGPASGAVHHS<br>SDSSTSASDSDTGSSPASSTPSSPRSFPATPLLDADIL<br>KFDLGSSQCFPTDDEWLLRTEFQLDSFYDAPPTLPETK<br>WPFSSVGSSWIDVSRLIRPSLDEEFESLL*
β-fg<br>(1590238)	scf718000065073:244391-245337	MDSFMNLAKQGMQAYERSQSDVSRTGGHEYNSPNHTTS<br>AQDRPQFDDDEVVNTASREGSGDTSLFSSALSFIKQNK<br>SAHEDPIDEEGVQHAHRKAYEEGSAGSLSAGSLGSAAA<br>MQVLKQFTSSGSSGTGSQSQLISLAMAEASKLFDSAGG<br>ASSGNKQDAVNGAAMTVMKLLVQSKFSGAIGGGNSGGL<br>GGLMGLASKFM*
***B* mating type locus**		
1344969<br>(Dam1)	scf7180000650862:151588-152768	MPPNPPQTPLRRLSQGSLFRLSRSGAYPDAPHGLGFLE<br>PALAELVDEAEALQTNVEGLRSLGDALGTFNESFASWL<br>YVMNMNALTTDWPQAPTQASFMLAARRAEEDARAALEA<br>IRAAEAAARRPPTPPEPTRTIERSDVATADGATESTFT<br>AAAPSASASASAPSGPGPGGAKPGVVVKKKVAGKPKMSAK<br>EKRERALEVERLVSALPLEFRGNDPTLRRHMEMVIEKF

		LDRPDEGYNIVAFVKPPDLNQARVNKCLIALVNRKIVRKDNSTGSVLYHWNGLPQ*
1774987 (Phl)	scf7180000650862:166107-166229	MDAFFTIAAPVPVEEPTVEDVLVDSDAVGTNGSHGSCVIA*
1774989 (Phl)	scf7180000650862:171000-170866	MDAFFVIATPVPAEDPASSSDSDILFADQDRLGTIGDHGSCVIA*
1774997 (Phl)	scf7180000650862:172759-172932	MDAFFTIAPAVPVEAPADVPMELDSTGTNGDHGSCTIA*
1774990 (Phl)	scf7180000650862:174396-174524	MDAFFTIAPAVPETVEDQPLETILVESDRLGTNGSHGSCVIA*
1774998 (Ph)	scf7180000650862:178211-178074	MDSFVCLTVFSDLASDPGNALTSDFVSREREVNHGSSSYSWCIVT*
1615275 (Ste3)	scf7180000650862:180536-178912	MRYPEFPVGAFLAAVLVLIPLPAHWRSRNIATVSIIIWLFVLDVVYGVNTIVWDSNIRKHLFVWCDITTKLAIGASVALPAATMCVCRNLELVASGRIARLTRDDKRRKIAFDLAMCFGLPALVMALHYIVQGHRFDILEFVGCLPATYYSIPAVFIIWFPPLLLSVLTSVYAALALRHFFRQRLTFAMQLKNSNSALSTGRYLRLVAMSILQIVWQTTLTALTMYDNISPGLRPWTNWADVHSDFGRVDTIPMFVYPVSYQRQFFLFLWVMPVSSYIFFIFFGFGEEAVKDYKNAFKWIRCKFFRQTLPESKTPLEDSFVRSPRSPLAPVKFTGLDSREDTLPAYSPASTDRTFAAFDKERVVIGEKITPDQLDTLSSVDDHSEVYSRPRHNYASSSFSSITTSPHIHISDAYPPPSPPAPVASLPVPTYHRPFSPPTVCPVSPTEPHALNAVHVTVQTRTEVEDMV*
1775001 (Ph)	scf7180000650862:181650-181456	MTAVSAFRVILQQSPSDRHSPVAPAGDLSNVPRIFIRCAPSNRPPSLPPKRVELLCSRCYCVVL*
159815 (Ph)	scf7180000650862:182012-181845	MDDFTDITSFSTILDSMAATSQESSDATPFLGPDGIPVDMEYVHGSSSYSWCVVA*
1774984 (Ph)	scf7180000650862:185053-185190	MDDFTVIIDPTNVPFPVTQPTSPSDYPTEQEQYGAGQTTFYCIIA*
1774991 (Ph)	scf7180000650862:186484-186681	MDEFSLPTVTSEDLVAQRALDDTSAHTSMLSSSAHRRDALFAAVASSTPVNEDRIEGYNGYCVIA*
1598157 (Ph)	scf7180000650862:188320-188165	MDNFDTIDSFIEPELIAPITSSRMDEDSMTYDVPIDAEHQPTTSHFWCIIA*
1598158 (Ste3)	scf7180000650862: 188973-190471	MSGVPSDPTYPAFPILAILGAVVVLVPLPWHFQAWNAGTCLFMIWTSIACLNLAINAIVWNNNALNLAPVWCDISSRIIIATGVALPAASLCIQRRLYNIASIKTVSITHAEKRKGILIDLAIGVGIPLLQVLFQYIVSGHRFDIYEGIGCYPFTYNTPLAYPLSIVWPLVVALVSACYCVLTFAAFLRRRAHFNAYLASNTSLTANRYFRLMGIASMEIVCTVPISAYGLYLNLAAAPVSPWISWADTHFNYSKVDQFPAVLWRMDRTAVISFELSRWLAPFCAFVFFGFFGFAQEARSSYRKAFLWICQHLPFRRMFPSRPPKGAVSIEKAAGWHSKQSQDILPQYSVRPPPLPSFSSSAATEDTLSISDTPLYEKFPDTPSTAGSDLSHDGLPHHGPHEV*
1775002 (Ph)	scf7180000650862:192157-191948 [gene has two potential starts: scf7180000650862:192100-191948]	MDEFSTIVILPADSVGICMDESLPCATPSSVSHPAPFRPNARTGLDSAMPVNEDRPDPTQVAGYCVIA* [MDESLPCATPSSVSHPAPFRPNARTGLDSAMPVNEDRPDPTQVAGYCVIA*]
1598161 (Ste3)	scf7180000650862:194621-193071	MHRELPAASILAALLVLLPTPWHWRAGNVATLSMIVWLFVVNVIYAVDSIIWSHNVARVALVWCDITTKILIGANIALPAACMCVCIHLEQVASIRQALTTLAQKRRRQIMEAILCYLVPIIWMGLHYIVQGHRFDIIEEFGCRPSVYVSIPAIFLLWVPSLIMSAVSLIFAGLAFTHFIRRRITFAKHLESSNSGLNASRYLRLMLLAVIEMFASAAAVSATLGFSVVSDMRPWTNWSDVHWDFGRIDTYPTVFLPPFLYNFYYA

		CWWIAPISAYVFCAFFAFGQEAMNEYRSYARWIWTRVL<br>RQKPRTSSSKASNGGFMPMPPSRSTLGFGQSVSISSLP<br>PAHLSMMSGELSPTTPGSPQTPSDIPDRKHVLGEHSKQ<br>DVILIGAPIVVYSRDDDLPSYYASSLSHSRPHSPSQTV<br>PTDVEPSSNDQDRIDQHGYHAV*
1583603 (Ste3)	scf7180000650862:200814-202688	MHAELPTVSFLCVASLLLVAPVYASSRNIPVLSLAAWL<br>ICCNLIHGINALVWAGNSAEHIPAWCDIVTRVLLAAQL<br>ALPGSALALVMKLRRCALGQETTGRRSPVAMDVILCLA<br>LPMIYIITHVIVQPHRFDIAAEFGCVASIHTSSVSIIL<br>IWIPPLLLCATTFVYTFLAIRARLESGLFFFSHMQDAP<br>RMSMLAFIRPLVISVSITLISLSMTIFSMTAHLVSVGG<br>LQAWAIDSWAQVHAEMSQVFVIPPTSHLILKSVEADWW<br>AIPAYTLVFVAMTGLAFIHPESLKPHGPVQRWFHRAVL<br>RRSPEDSFSQAKSFGGQTLCSSPSSPTSMYEMKAGWDD<br>TWRPSAPAKVKLPPLVIPAAPSETTIAFAEPEQDDLFA<br>RSTMRYVESPTGREALGLPPIPPAIYEPGRRNGSVSPP<br>MTPPRVASPPKPNTTLQDGETAQARPDSMILSSGAWPR<br>PPSTIPASPRTPSPKTPVMVTPASPVPPHSPSSSPRPP<br>SILSMTTSLASSTISVGGYAYDDEPYVAPFQDSVSDAP<br>GPGLAVPKHIRKVRSRDMLLPRSFSVSSRGRRNGSNGG<br>LSEGIYMTVVKETE*
1583617 (Ste3)	scf7180000650862:212378-213414	MSIPWLVQVDIVAAFSLLGFLLVSIPLYWHLEAWNVGC<br>VLYIFWIGTQSLFQFINVMVWRDNAINWAPVWCDITTR<br>FTIGSSIGICCASLVINRRLYHIANISTVSISRADKRR<br>NIITDLAIGLGIPVVAIAVYWFYQGHRFDILEGIGCIE<br>EYPNSFLAYLLYVAWPIPIGLVSATYCTLTLRAFFKRR<br>RDFSTLVASNNNLTFNRYFRLMGLAAIEVLFTIPLTTY<br>NIVQNLTTLPIYPFIGYADLHYWFSRVDQKSAVSWRAD<br>PGTVARMAFKQWTVIGCALLFFLFFGLAEEARKHYRLA<br>LSSVAKRVGLSTFEKGTGFSSTGYPTTSGFGKITIPTF<br>VQRSTRRGSMDSFSDRLSTNISISDAIDEKAAPYSPVG<br>GSSTGGSSTCIASPVDTEKGISPLESLPPVSLPPVSFP<br>PPPRAYDPESPTRRDADVPASVRPNSIDMV*
***STE20* locus**		
1774996 (Phl)	scf7180000650810:223340-223221	MDAFFTIAPPVPVDEPTTEVLVDRDDTGTNGDHGSCTI<br>A*
1775003 (Ste20)	scf7180000650810:227312-224926 (5’end missing by poor DNA sequences)	...SSSSSSDNGRGYQPKQLSKSPPPSPGSERKARPEP<br>WPKARPEPWPFVPSNNPAAQYSLLEKLGTGSFGTVYKA<br>IHNETKQIVAIKQIDLEDTDDDISEIQQEIANLAQCDS<br>EYVTRYYGSFVVAYKLWIVMEFLAGGSCLDLLKAGSFS<br>EAHIAVICRELLLGLDYLHSEGTIHRDIKAANVLLSAS<br>GKVKLADFGVAAQLTSTLRHTFVGTPFWMAPEVIRQAG<br>YDAKADIWSLGITAIEMAKGEPPLAEYHPMRVLFLIPK<br>AKPPVLEGPFSMAFKDFVAQCLTKDPHSRPTTKELLQH<br>RFIKNARKTSYLTELIERYQDYRNRSPGRGPQMYQATV<br>RNSGAWDGTLRSEWDFNTIRSTSAMGSLRSMAKDIMPP<br>GMEPDDQYYDEMQEEDESTYEAQSSIDTAAATTKGSDV<br>PLQPQIRGLGMNADAGHSTVVIRSYAPTGEQKDAPSLM<br>SDNGSDETTGTAVVTPLQSVEDVEAQVADATEPPPAYS<br>GSVRSNRRASYAMRNNVSSGTVLSEADIGTGVDTIRPV<br>KKVDAVRSLRLSEEYVGSLRSREGSVSSVPSSPQSSKG<br>SHKRGPSEFAKAGKAIVEDVLLPLLQKTTRDDMDAREI<br>ESLSMISRGFEELGAVNPELAYNLVLDMLSGINENSAI<br>RNHIQTSRSLFPHKRIIRRSEMTAKGLVVTEEEEISGL<br>PTPSTSSPAQPQAEPGSPVRKSPISELLYLRWLEGLKL<br>KWPSIL*

Table S3. Numbers of predicted B mating type genes and similar non-mating-type genes found in the genomes of the four *Pycnoporus* strains

Strain/gene type	*P. cinnabarinus* BRMF 137	*P. coccineus* BRMF 310	*P. puniceus* BRMF 1868	*P. sanguineus* BRFM 1264
Ste3 B mating type genes	2	3	4	2
Ste3 non-mating-type genes	2	2	2	2
Ph B mating type genes pheromones	4	5	6	7
Phl non-mating-type genes	5	6	6	5

Table S4. Identities and similarities between orthologous proteins linked to mating type or mating-type-like genes in *Pycnoporus* species

**Protein**	**Identity/similarity**				
**Strain**	*P. cinnabarinus* BRFM 137	*P. puniceus* BRFM 1868	*P. coccineus* BRFM 310	*P. sanguineus* BRFM 1264	*Trametes versicolor* FP-101664 SS1
**Mip1**					
P. cinnabarinus BRFM 137	100 %	80/86 %	82/87 %	82/88 %	80/85 %
P. puniceus BRMF 1868		100 %	82/89 %	83/91 %	80/88 %
P. coccineus BRFM 310			100 %	96/98 %	84/89 %
P. sanguineus BRFM 1264				100 %	83/89%
**HD1**					
P. cinnabarinus BRFM 137	100 %	31/47 %	30/47 %	33/51 %	30/46 %
P. puniceus BRFM 1868		100 %	35/50 %	36/54 %	37/50 %
P. coccineus BRFM 310			100 %	46/60 %	32/48 %
P. sanguineus BRFM 1264				100 %	35/50 %
**HD2**					
P. cinnabarinus BRFM 137	100 %	36/51 %	34/48 %	38/51 %	41/57 %
P. puniceus BRFM 1868		100 %	37/50 %	34/49 %	38/51 %
P. coccineus BRFM 310			100 %	55/67 %	38/54 %
P. sanguineus BRFM 1264				100 %	39/53 %
**β-fg**					
P. cinnabarinus BRFM 137	100 %	80/89 %	85/90 %	83/90 %	81/88 %
P. puniceus BRFM 1868		100 %	82/89 %	83/91 %	78/83 %
P. coccineus BRFM 310			100 %	96/98 %	84/86 %
P. sanguineus BRFM 1264				100 %	88/90 %
**Ste3.1**					
P. cinnabarinus BRFM 137	100 %	52/68 %	45/62 %	46/64 %	48/63 %
P. puniceus BRFM 1868		100 %	43/61 %	47/68 %	52/68 %
P. coccineus BRFM 310			100 %	90/94 %	51/67 %
P. sanguineus BRFM 1264				100 %	44/63 %
**Ste3.2**					
P. cinnabarinus BRFM 137	100 %	49/71 %	38/62 %	53/76 %	52/75 %
P. puniceus BRFM 1868		100 %	37/58 %	35/37 %	53/71 %
P. coccineus BRFM 310			100 %	64/81 %	38/56 %
P. sanguineus BRFM 1264				100 %	34/52 %
**Ste3.3**					
P. cinnabarinus BRFM 137	100 %	65/77 %	73/83 %	75/83 %	57/69 %
P. puniceus BRFM 1868		100 %	70/83 %	69/82 %	66/78 %
P. coccineus BRFM 310			100 %	90/95 %	62/75 %
P. sanguineus BRFM 1264				100 %	63/75 %
**Ste3.4**					
P. cinnabarinus BRFM 137	100 %	80/89 %	87/92 %	86/93 %	74/84 %
P. puniceus BRFM 1868		100 %	83/90 %	83/90 %	71/81 %
P. coccineus BRFM 310			100 %	92/95 %	74/83 %
P. sanguineus BRFM 1264				100 %	73/83 %
**Dam1**					
P. cinnabarinus BRFM 137	100 %	80/84 %	78/82 %	77/81 %	73/81 %
P. puniceus BRFM 1868		100 %	82/85 %	83/ 86 %	80/84 %
P. coccineus BRFM 310			100 %	88/92 %	72/79%
P. sanguineus BRFM 1264				100 %	71/77 %
**Ste20**					
P. cinnabarinus BRFM 137	100 %	90/93 %	93/95 %	90/93 %	84/90 %
P. puniceus BRFM 1868		100 %	90/93 %	91/94 %	87/92 %
P. coccineus BRFM 310			100 %	96/97 %	87/92 %
P. sanguineus BRFM 1264				100 %	86/91 %

Table S5. JGI protIDs for the 60 protein models of class I peroxidase (CCP), class II peroxidase (short MnP, LiP, and typical and atypical VP) and HTP peroxidase genes in the genomes of three *Pycnoporus* species.

	P. cinnabarinus BRFM 137	*P. coccineus* BRFM 310	*P. sanguineus* BRFM 1264
MnP-short	480<br>737<br>7498	1369658<br>1436321<br>1464049<br>1468611	35461<br>1603419<br>1651739<br>1672299
LiP	7499<br>7500<br>7501<br>7860	779035<br>859168<br>1403742<br>1431101	1603832<br>1676766<br>1676767<br>1748658[b] (LiP?)
VP	6829	1468768<br>1469331	1587729<br>1607934
VP-atypical	6481	1438352	1679991
HTP	5123[a]<br>6655<br>6658	1374080<br>1438096<br>1438101<br>1470054	339361<br>1585728<br>1585846<br>1682339
CCP	1679[a]	1449695	1686233

[a] Two new heme peroxidases identified in the *P. cinnabarinu* s genome not described by Levasseur et al., 2014. [b] Putative pseudogene of a LiP in *P. sanguineus* BRFM 1264.

Table S6. JGI protIDs for the copper radical oxidase gene models in the genomes of three *Pycnoporus* species. CRO, Copper Radical Oxidase; GLOX, Glyoxal Oxidase

	ProteinID	family	expert annot	Ref
P. cinnabarinus	35	AA5_1	CRO1	
P. cinnabarinus	7854	AA5_1	CRO2	
P. cinnabarinus	9003	AA5_1	CRO3-5 possible mis annotation of intron-exon structure	
P. cinnabarinus	9339	AA5_1	CRO6	
P. cinnabarinus	2059	AA5_1	Characterized PciGLOX1	Daou et al., 2016
P. cinnabarinus	2053	AA5_1	Characterized PciGLOX2	Daou et al., 2016
P. cinnabarinus	2052	AA5_1	PciGLOX3	
P. coccineus	1382948	AA5_1	CRO1	
P. coccineus	1357891	AA5_1	CRO2	
P. coccineus	1367686	AA5_1	CRO3-5 possible mis annotation of intron-exon structure	
P. coccineus	1356493	AA5_1	CRO6	
P. coccineus	1376269	AA5_1	GLOX1	
P. coccineus	1454649	AA5_1	GLOX2	
P. coccineus	1480943	AA5_1	GLOX3	
P. sanguineus	1673053	AA5_1	CRO1	
P. sanguineus	1689209	AA5_1	CRO2	
P. sanguineus	1672103	AA5_1	CRO3-5 possible mis annotation of intron-exon structure	
P. sanguineus	1587126	AA5_1	CRO6	
P. sanguineus	1605696	AA5_1	GLOX1	
P. sanguineus	1578731	AA5_1	GLOX2	
P. sanguineus	1605701	AA5_1	GLOX3	

Table S7. Genes coding for laccases, multicopper oxidases and ferroxidases in the genomes of *P. cinnabarinus* BRFM 137, *P. coccineus* BRFM 310 and *P. sanguineus* BRFM 1264.

		ProtID		Reference
P. cinnabarinus	scf185007.g107	4351	lac1	Lomascolo et al., 2003
P. cinnabarinus	scf184817.g29	4273	lac2	Otterben et al., 2000
P. cinnabarinus	scf185007.g100	5626	lac3	Levasseur et al. 2014
P. cinnabarinus	scf184918.g4	8665	lac4	Levasseur et al. 2014
P. cinnabarinus	scf184851.g84	3446	lac5	Levasseur et al. 2014
P. cinnabarinus	scf184857.g29	8672	MCO	Levasseur et al. 2014
P. cinnabarinus	scf184845.g66	4119	ferroxidase	Levasseur et al. 2014
P. sanguineus	scf_71800006508	1583166	lac1	this manuscript
P. sanguineus	scf_71800006506	1560767	lac2	this manuscript
P. sanguineus	scf_71800006508	1326600	lac3	this manuscript
P. sanguineus	scf_71800006508	1639180	lac4	this manuscript
P. sanguineus	scf_71800006507	1562799	lac5	this manuscript
P. sanguineus	scf_71800006508	1578711	MCO	this manuscript
P. sanguineus	scf_71800006508	104411	ferroxidase	this manuscript
P. coccineus	scf_16:614620-61	1366139	lac1	this manuscript
P. coccineus	scf_23:118619-12	1452465	lac2	this manuscript
P. coccineus	scf_16:586144-58	1434777	lac3	this manuscript
P. coccineus	scf_18:182367-18	1477425	lac4	this manuscript
P. coccineus	scf_10:670001-67	1363242	lac5	this manuscript
P. coccineus	scf_29:289535-29	1370215	MCO	this manuscript
P. coccineus	scf_19:448013-45	1425237	ferroxidase	this manuscript

Table S8: AA3 gene transcription regulation and protein detection in the secretomes of *Pycnoporus coccineus* BRFM310. For some enzymes, activities could be predicted from a plylogenetic analysis with 46 characterized fungal AA3 GMC-oxidoreductases. Prediction for secretion was deduced from the presence of a predicted signal peptide, the absence of other trans-membrane domains and the absence of signal sequence for retention in the endoplasmic reticulum. Secretomes and transcriptomes were collected after three day growth on maltose (M); avicel (AVI), wheat straw (WS), Aspen (As) or Pine (Pi).

protID	CAZyme sub-family	predicted activity	secretomes					log2 fold change as compared to M					predicted secreted
			M	AVI	WS	As	Pi	M	AVI	WS	As	Pi	
1429888	AA3_2	-						5,19	5,91	6,67	5,32	5,32	Y
1368367	AA3_2	-						-2,11	NA	NA	1,83	1,83	Y
1434275	AA3_2	-						NA	2,51	1,80	NA	NA	
1475753	AA3_2	-						NA	NA	NA	NA	NA	
1362859	AA3_2	-						1,66	NA	1,62	2,71	2,71	
1367218	AA3_2	-						NA	1,49	1,21	NA	NA	Y
1367759	AA3_2	-						NA	NA	NA	NA	NA	
1432108	AA3_2	GDH	Y	Y	Y	Y	Y	1,93	NA	1,53	1,77	1,77	Y
1453351	AA3_2	-						NA	NA	NA	NA	NA	
1433476	AA3_2	-						NA	NA	-2,28	NA	NA	
1463000	AA3_2	-						1,40	1,91	2,34	2,69	2,69	Y
1462838	AA3_2	-						-2,05	-2,46	-2,20	-3,11	-3,11	
1437841	AA3_2	-						NA	NA	NA	NA	NA	
1437749	AA3_2	-						NA	NA	NA	NA	NA	
1465734	AA3_2	AAO	Y	Y				2,80	2,68	3,26	3,91	3,91	Y
1370120	AA3_2	AAO						NA	NA	NA	1,27	1,27	Y
1466067	AA3_2	-						1,80	NA	NA	NA	NA	
1462832	AA3_2	-						NA	NA	NA	NA	NA	
68108	AA3_2	AAO	Y	Y				1,06	-2,29	-1,93	-1,85	-1,85	
1436734	AA3_2	-						NA	NA	NA	-1,25	-1,25	
1368318	AA3_3	AOx						5,09	7,27	8,30	7,53	7,53	
1465271	AA3_3	AOx						4,46	2,97	4,79	4,16	4,16	
1414868	AA3_3	AOx						2,46	NA	1,84	NA	NA	
1471985	AA3_4	POx						NA	NA	NA	2,56	2,56	
1440372	AA3_4	POx						NA	NA	NA	3,38	3,38	

Table S9. *P. cinnabarinus* BRFM 137 genome annotation related to protein secretion pathways

**ENTRY INTO ER**				
**SIGNAL RECOGNITION**				
Pycnoporus cinnabarinus Gene ID	*Aspergillus niger* Gene ID	*Saccharomyces cerevisiae* Gene ID	Gene Name	Description in SGD
No hits found	An01g02800	YPL243w	SRP68	Core component of the signal recognition particle (SRP) ribonucleoprotein (RNP) complex that functions in targeting nascent secretory proteins to the endoplasmic reticulum (ER) membrane
No hits found	An04g06890	YPL210c	SRP72	Core component of the signal recognition particle (SRP) ribonucleoprotein (RNP) complex that functions in targeting nascent secretory proteins to the endoplasmic reticulum (ER) membrane
scf184845.g53	An01g10070	YML105c	SEC65	Subunit of the signal recognition particle (SRP), involved in protein targeting to the ER; interacts with Srp54p; homolog of mammalian SRP19
scf184637.g5	An15g06470*		-	similarity to signal sequence receptor alpha chain Canis lupus
No hits found	An07g05800	YDL092w	SRP14	Signal recognition particle (SRP) subunit
scf184785.g11	An09g06320	YPR088c	SRP54	Signal recognition particle (SRP) subunit (homolog of mammalian SRP54)
scf184798.g87	An15g01670	YDR292c	SRP101	Signal recognition particle (SRP) receptor alpha subunit; contain GTPase domains; involved in SRP-dependent protein targeting; interacts with the beta subunit, Srp102p
No hits found	An05g00140	YKL154w	SRP102	Signal recognition particle (SRP) receptor beta subunit; involved in SRP-dependent protein targeting; anchors the alpha subunit, Srp101p to the ER membrane
**SIGNAL PEPTIDASE COMPLEX**				
scf184996.g50	An01g00560	YIR022w	SEC11	subunit of the Signal Peptidase Complex which cleaves the signal sequence of proteins targeted to the ER
No hits found	An17g02095	YJR010c-a	SPC1	subunit of the Signal Peptidase Complex which cleaves the signal sequence of proteins targeted to the ER
scf184600.g16	An16g07390*	YML055w	SPC2	subunit of the Signal Peptidase Complex which cleaves the signal sequence of proteins targeted to the ER
scf184696.g16	An09g05420*	YLR066w	SPC3	subunit of the Signal Peptidase Complex which cleaves the signal sequence of proteins targeted to the ER
**TRANSLOCATION INTO ER**				
scf184707.g3<br>scf184707.g1<br>scf184874.g3<br>scf184711.g1<br>scf184492.g1	An03g04340	YLR378c	SEC61	subunit of Sec61 complex (Sec61p, Sbh1p, and Sss1p); forms a channel for SRP-dependent protein import and retrograde transport of misfolded proteins out of the ER;
No hits found	An01g03820	YER087c-b	SBH1	Beta subunit of the Sec61p ER translocation complex (Sec61p-Sss1p-Sbh1p); involved in protein translocation into the endoplasmic reticulum
scf185007.g14	An01g11630	YDR086c	SSS1	Beta subunit of the Sec61p ER translocation complex (Sec61p-Sss1p-Sbh1p); involved in protein translocation into the endoplasmic reticulum
No hits found	An01g03820	YER019c-a	SBH2	Ssh1p-Sss1p-Sbh2p complex component, involved in protein translocation into the endoplasmic reticulum
scf184583.g3	An02g01510	YPL094c	SEC62	Essential subunit of Sec63 complex

				(Sec63p, Sec62p, Sec66p and Sec72p); with Sec61 complex, Kar2p/BiP and Lhs1p forms a channel competent for SRP-dependent and post-translational SRP-independent protein targeting and import into the ER
scf184370.g7	An01g13070	YOR254c	SEC63	Essential subunit of Sec63 complex (Sec63p, Sec62p, Sec66p and Sec72p); with Sec61 complex, Kar2p/BiP and Lhs1p forms a channel competent for SRP-dependent and post-translational SRP-independent protein targeting and import into the ER
scf184863.g26	An16g08830	YBR171w	SEC66	Non-essential subunit of Sec63 complex (Sec63p, Sec62p, Sec66p and Sec72p); with Sec61 complex, Kar2p/BiP and Lhs1p forms a channel competent for SRP-dependent and post-translational SRP-independent protein targeting and import into the ER
**PROTEIN FOLDING IN THE ER**				
scf184858.g22	An02g14800	YCL043c	PDI1	Protein disulfide isomerase; essential for the formation of disulfide bonds in secretory and cell-surface proteins, unscrambles non-native disulfide bonds
scf184844.g23	An18g02020	YCL043c	PDI1	Protein disulfide isomerase; essential for the formation of disulfide bonds in secretory and cell-surface proteins, unscrambles non-native disulfide bonds
scf184844.g23<br>scf184858.g22	An01g04600	YOR288C	MPD1	Member of the protein disulfide isomerase (PDI) family
scf184613.g2	An02g05890	YIL005W	EPS1	ER protein with chaperone and co-chaperone activity, involved in retention of resident ER proteins; has a role in recognizing proteins targeted for ER-associated degradation (ERAD), member of the protein disulfide isomerase family
scf184775.g6	An16g07620	YML130c	ERO1	Thiol oxidase required for oxidative protein folding in the endoplasmic reticulum
scf184395.g1	An08g07810	YDL045c	FAD1	Flavin adenine dinucleotide (FAD) synthetase, performs the second step in synthesis of FAD from riboflavin
scf185015.g72	An08g06370	YBL033c	RIB1	GTP cyclohydrolase II; catalyzes the first step of the riboflavin biosynthesis p
scf184900.g4	An10g00350	-	-	similarity to GTP cyclohydrolase II ribA - Actinobacillus<br>pleuropneumoniae
scf184794.g18	An12g06490	-	-	similarity to N-oxide-forming dimethylaniline monooxygenase<br>FMO1 - Homo sapiens
No hits found	An18g06470	-	-	similarity to N-oxide-forming dimethylaniline monooxygenase<br>FMO1 - Homo sapiens
scf184797.g7	An05g00880	YMR214w	SCJ1	homolog of bacterial chaperone DnaJ, located in the ER lumen where it cooperates with Kar2p to mediate maturation of proteins
scf185007.g186	An01g08420	YAL058w	CNE1	Calnexin; integral membrane ER chaperone involved in folding and quality control of glycoproteins
scf184966.g13	An04g02020	YDR155c	CPR1	Cytoplasmic peptidyl-prolyl cis-trans isomerase (cyclophilin), catalyzes the cis-trans isomerization of peptide bonds N-terminal to proline residues
scf184914.g1	An01g06670	YDR519w	FPR2	Membrane-bound peptidyl-prolyl cis-trans isomerase (PPIase
scf184999.g48	An11g04180	YJL034w	KAR2	ATPase involved in protein import into the ER, also acts as a chaperone to mediate protein folding in the ER and may play a role in ER export of soluble proteins; regulates the unfolded protein response via interaction with Ire1p

scf184902.g6	An01g13220	YJL034w	KAR2	ATPase involved in protein import into the ER, also acts as a chaperone to mediate protein folding in the ER and may play a role in ER export of soluble proteins; regulates the unfolded protein response via interaction with Ire1p
**PROTEIN MISFOLDING**				
**UNFOLDED PROTEIN RESPONSE (UPR)**				
No hits found	An01g00160	YFL031W	HAC1	Basic leucine zipper (bZIP) transcription factor (ATF/CREB1 homolog) that regulates the unfolded protein response
scf184776.g5	An01g06550	YHR079c	IRE1	Serine-threonine kinase and endoribonuclease; transmembrane protein that mediates the UPR by regulating Hac1p synthesis through HAC1 mRNA splicing
scf184855.g11<br>scf184961.g15	An14g04770	YDL006w	PTC1	Type 2C protein phosphatase (PP2C)
scf184909.g43	An08g00830	YER089c	PTC2	Type 2C protein phosphatase (PP2C); dephosphorylates Ire1p to downregulate the unfolded protein response
scf184926.g8	An08g01480	YJL087c	TRL1	tRNA ligase, required for tRNA splicing and for both splicing and translation of HAC1 mRNA in the UPR
No hits found	An01g07900	YEL009c	GCN4	bZIP transcriptional activator of amino acid biosynthetic genes in response to amino acid starvation; expression is tightly regulated at both the transcriptional and translational
scf184817.g21	An11g11250	YLR090w	XDJ1	Putative chaperone, homolog of E. coli DnaJ
scf184759.g9	An01g08980	YGR038w	ORM1	required for resistance to agents that induce unfolded protein response;
**ER ASSOCIATED DEGRADATION (ERAD)**				
scf184805.g32<br>scf184970.g113<br>scf184908.g154	An04g09170	YDL126c	CDC48	ATPase involved in ubiquitin-mediated protein degradation
scf184990.g26	An01g05330	YBR170c	NPL4	Ubiquitin-binding protein involved in protein degradation; Cdc48p-Npl4p-Ufd1p complex participates in (ERAD)
scf185002.g115	An01g05760	YGR048w	UFD1	Involved in recognition of polyubiquitinated proteins and their presentation to the 26S proteasome for degradation
scf184909.g16<br>scf184693.g11	An15g00640	YBR201W	DER1	ER membrane protein, required for ERAD of misfolded or unassembled proteins;
scf185014.g85	An03g04600	YKL213c	DOA1	WD repeat protein required for ubiquitin-mediated protein degradation
scf184499.g21	An08g09000	YMR276w	DSK2	Nuclear-enriched ubiquitin-like polyubiquitin-binding protein
scf184677.g8	An16g07970	YOL013c	HRD1	Ubiquitin-protein ligase; required for ERAD of misfolded proteins
scf184970.g123	An17g00260	YDR177w	UBC1	Ubiquitin-conjugating enzyme that mediates selective degradation of short-lived and abnormal proteins
scf184767.g2	An06g01120	YGL058w	RAD6	Ubiquitin-conjugating enzyme (E2),
scf184999.g39	An09g06110	YMR022w	UBC7	Ubiquitin conjugating enzyme, involved in the ER-associated protein degradation pathway;
scf184909.g21	An04g01730	YDL190c	UFD2	Ubiquitin chain assembly factor (E4) that cooperates with a ubiquitin-activating enzyme (E1), a ubiquitin-conjugating enzyme (E2), and a ubiquitin protein ligase (E3) to conjugate ubiquitin to substrates
scf184851.g10	An04g01720	YMR161w	HLJ1	Co-chaperone for Hsp40p, anchored in the ER membrane; with its homolog Ydj1p promotes ER-associated protein degradation (ERAD) of integral membrane substrates
scf185043.g124	An12g00340	YHR204w	MNL1	Alpha-1,2-specific exomannosidase of the endoplasmic reticulum;
scf184763.g4	An18g06220	YJR131w	MNS1	Alpha-1,2-mannosidase involved in ER-associated protein degradation (ERAD
scf184650.g6	An04g00360	YLR208w	SEC13	Structural component of three distinct

scf184981.g1				complexes
scf184917.g17	An12g04000	YIL030c	SSM4	Ubiquitin-protein ligase involved in ER-associated protein degradation
scf184962.g12	An14g00230	YER100w	UBC6	Ubiquitin-conjugating enzyme involved in ER-associated protein degradation
scf184636.g10	An01g04280	YNL064c	YDJ1	Type I HSP40 co-chaperone involved in regulation of the HSP90 and HSP70 functions; involved in protein translocation across membranes
scf184750.g6	An15g01420	YGL027c	CWH41	Processing alpha glucosidase I, ER type II integral membrane N-glycoprotein involved in assembly of cell wall beta 1,6 glucan and asparagine-linked protein glycosylation; also involved in ER protein quality control and sensing of ER stress
**PROTEASOME**				
No hits found	An18g06520	YHL030w	ECM29	Scaffold protein that assists in association of the proteasome core particle with the regulatory particle
scf184280.g10	An18g03010	YHR027c	RPN1	Non-ATPase base subunit of the 19S regulatory particle of the 26S proteasome;
scf185013.g137	An04g03270*	YIL075c	RPN2	Subunit of the 26S proteasome, substrate of the N-acetyltransferase Nat1p
scf184569.g18	An11g10380	YER021w	RPN3	Essential, non-ATPase regulatory subunit of the 26S proteasome lid
No hits found	An08g06850	YDL020c	RPN4	Transcription factor that stimulates expression of proteasome genes;
scf185013.g98	An11g09690	YDL147w	RPN5	Subunit of the COP9 signalosome (CSN) and non-ATPase regulatory subunit of the 26S proteasome
scf184895.g14<br>scf184702.g6<br>scf184895.g15	An18g05070	YDL097c	RPN6	Essential, non-ATPase regulatory subunit of the 26S proteasome
scf184895.g13	An11g02610	YPR108w	RPN7	Essential, non-ATPase regulatory subunit of the 26S proteasome, similar
scf184996.g61	An07g10110	YOR261c	RPN8	Essential, non-ATPase regulatory subunit of the 26S proteasome
scf184788.g16	An08g10710	YDR427w	RPN9	Non-ATPase regulatory subunit of the 26S proteasome
scf184613.g5	An15g03020	YHR200w	RPN10	Non-ATPase base subunit of the 19S regulatory particle (RP) of the 26S proteasome
scf184908.g81	An07g07860	YFR004w	RPN11	Metalloprotease subunit of the 19S regulatory particle of the 26S proteasome lid
scf184652.g4	An16g02210	YFR052w	RPN12	Subunit of the 19S regulatory particle of the 26S proteasome lid;
scf184593.g7	An02g12760	YKL145w	RPT1	One of six ATPases of the 19S regulatory particle of the 26S proteasome
scf184756.g27	An17g00270	YDL007w	RPT2	One of six ATPases of the 19S regulatory particle of the 26S proteasome
scf184785.g44	An02g07190	YDR394w	RPT3	One of six ATPases of the 19S regulatory particle of the 26S proteasome
scf184815.g19	An18g06230	YOR259c	RPT4	One of six ATPases of the 19S regulatory particle of the 26S proteasome
scf184915.g60	An18g05230	YOR117w	RPT5	One of six ATPases of the 19S regulatory particle of the 26S proteasome
scf184845.g22	An14g00180	YGL048c	RPT6	One of six ATPases of the 19S regulatory particle of the 26S proteasome

**Protein complex involved in protein transport**				
**Exocyst Complex**				
scf185007.g35	An01g03190	YER008c	SEC3	Subunit of the exocyst complex which mediates targeting of post-Golgi vesicles to sites of active exocytosis
scf184921.g16	An08g05570	YDR166c	SEC5	Subunit of the exocyst complex which mediates targeting of post-Golgi vesicles to sites of active exocytosis
scf184962.g24	An04g06180	YIL068c	SEC6	Subunit of the exocyst complex which

				mediates targeting of post-Golgi vesicles to sites of active exocytosis
scf185007.g49	An03g04210	YPR055w	SEC8	Subunit of the exocyst complex which mediates targeting of post-Golgi vesicles to sites of active exocytosis
scf185003.g18	An03g06900	YLR166c	SEC10	Subunit of the exocyst complex which mediates targeting of post-Golgi vesicles to sites of active exocytosis
scf184908.g161	An15g00010	YGL233w	SEC15	Subunit of the exocyst complex which mediates targeting of post-Golgi vesicles to sites of active exocytosis
scf184926.g7	An02g04030*	YJL085w	EXO70	Subunit of the exocyst complex which mediates targeting of post-Golgi vesicles to sites of active exocytosis
scf184753.g42	An08g07370	YBR102c	EXO84	Subunit of the exocyst complex which mediates targeting of post-Golgi vesicles to sites of active exocytosis
scf185001.g27	An14g00010	YFL005w	SAR1	Subunit of the exocyst complex which mediates targeting of post-Golgi vesicles to sites of active exocytosis
scf184981.g2	An18g05980	YPR165w	RHO1	Subunit of the exocyst complex which mediates targeting of post-Golgi vesicles to sites of active exocytosis
scf184981.g2	An16g04200	YNL090w	RHO2	Non-essential small GTPase of the Rho/Rac subfamily involved in the establishment of cell polarity and in microtubule assembly
scf185008.g5	An11g09620	YIL118w	RHO3	Non-essential small GTPase of the Rho/Rac subfamily of Ras-like proteins involved in the establishment of cell polarity
scf184636.g11	An14g05530	YKR055w	RHO4	Non-essential small GTPase of the Rho/Rac subfamily of Ras-like proteins, likely to be involved in the establishment of cell polarity
scf185007.g269	An11g10030	YNL180c	RHO5/RAC	Non-essential small GTPase of the Rho/Rac subfamily of Ras-like proteins, likely involved in protein kinase C (Pkc1p)-dependent signal transduction pathway that controls cell integrity
scf185000.g58	An02g14200	YLR229c	CDC42	Small rho-like GTPase, essential for establishment and maintenance of cell polarity; mutants have defects in the organization of actin and septins
**SEC34/SEC35 Complex**				
scf184585.g3	An02g06840	YER157w	SEC34	Essential component of the conserved oligomeric Golgi complex (Cog1p through Cog8p), a cytosolic tethering complex that functions in protein trafficking to mediate fusion of transport vesicles to Golgi compartments
scf184834.g50	An02g14400	YPR105c	COG4	Essential component of the conserved oligomeric Golgi complex (Cog1p through Cog8p), a cytosolic tethering complex that functions in protein trafficking to mediate fusion of transport vesicles to Golgi compartments
No hits	An16g03450	YNL051w	COG5	Component of the conserved oligomeric Golgi complex (Cog1p through Cog8p), a cytosolic tethering complex that functions in protein trafficking to mediate fusion of transport vesicles to Golgi compartments (1, 3)
scf185014.g111	An06g01630	YNL041c	COG6/SEC37	Component of the conserved oligomeric Golgi complex (Cog1p through Cog8p), a cytosolic tethering complex that functions in protein trafficking to mediate fusion of transport vesicles to Golgi compartments
**Trapp Complex**				
scf184799.g53	An12g00380	YKR068c	BET3	Hydrophilic protein that acts in conjunction with SNARE proteins in targeting and fusion of ER to Golgi transport vesicles;

				component of the TRAPP
scf184979.g5	An17g01875	YML077w	BET5	Component of the TRAPP (transport protein particle) complex, which plays an essential role in the vesicular transport from endoplasmic reticulum to Golgi
No hits found	An04g08690	YDR108w	TRS85	Subunit of TRAPPIII (transport protein particle), a multimeric guanine nucleotide-exchange factor for Ypt1p, required for membrane expansion during autophagy and the CVT pathway; directs Ypt1p to the PAS; late post-replication meiotic role
scf184863.g20	An15g00470	YDR407c	TRS120	One of 10 subunits of the transport protein particle (TRAPP) complex of the cis-Golgi which mediates vesicle docking and fusion; involved in endoplasmic reticulum (ER) to Golgi membrane traffic
scf185002.g59	An08g05190	YMR218c	TRS130	One of 10 subunits of the transport protein particle (TRAPP) complex of the cis-Golgi which mediates vesicle docking and fusion; involved in ER to Golgi membrane traffic; mutation activates transcription of OCH1
No hits found	An15g03010	YDR246w	TRS23	One of 10 subunits of the transport protein particle (TRAPP) complex of the cis-Golgi which mediates vesicle docking and fusion; involved in endoplasmic reticulum (ER) to Golgi membrane traffic; human homolog is TRAPPC4
scf184829.g45	An14g06440	YDR472w	TRS31	One of 10 subunits of the transport protein particle (TRAPP) complex of the cis-Golgi which mediates vesicle docking and fusion; involved in endoplasmic reticulum (ER) to Golgi membrane traffic
scf184829.g75	An15g00060	YOR115c	TRS33	One of 10 subunits of the transport protein particle (TRAPP) complex of the cis-Golgi which mediates vesicle docking and fusion; involved in endoplasmic reticulum (ER) to Golgi membrane traffic
**COPI SUBUNITS**				
scf184970.g118	An16g05370	YER122c	GLO3	ADP-ribosylation factor GTPase activating protein (ARF GAP), involved in ER-Golgi transport;
scf184569.g24	An16g02460	YDL145c	COP1	Alpha subunit of COPI vesicle coatomer complex, which surrounds transport vesicles in the early secretory pathway
scf185013.g48	An01g14260	YFR051c	RET2	Delta subunit of the coatomer complex (COPI), which coats Golgi-derived transport vesicles; involved in retrograde transport between Golgi and ER
scf184847.g10	An12g04830	YPL010w	RET3	Zeta subunit of the coatomer complex (COPI), which coats Golgi-derived transport vesicles; involved in retrograde transport between Golgi and ER
scf185007.g92	An07g06030<br>An14g03050	YNL287w	SEC21	Gamma subunit of coatomer, a heptameric protein complex that together with Arf1p forms the COPI coat; involved in ER to Golgi transport of selective cargo
scf184494.g2	An02g05870	YGL137w	SEC27	Essential beta'-coat protein of the COPI coatomer, involved in ER-to-Golgi and Golgi-to-ER transport; contains WD40 domains that mediate cargo selective interactions;
scf184908.g162	An08g03690	YDL137w	ARF1/ARF2	ADP-ribosylation factor, GTPase of the Ras superfamily involved in regulation of coated formation vesicles in intracellular trafficking within the Golgi;
**COPII SUBUNITS**				
scf185002.g129	An01g04040	YPL218w	SAR1	GTPase, GTP-binding protein of the ARF family, component of COPII coat of vesicles; required for transport vesicle formation during ER to Golgi protein transport
scf185007.g231	An08g03270	YDR238c	SEC26	Essential beta-coat protein of the COPI

				coatomer, involved in ER-to-Golgi protein trafficking and maintenance of normal ER morphology
scf184650.g6	An04g00360	YLR208w	SEC13	Structural component of three distinct complexes; subunit of the Nup84 nuclear pore sub-complex (NPC), the COPII vesicle coat, and the Seh1-associated (SEA) complex
scf185002.g1	An02g01690	YDL195w	SEC31	ponent of the Sec13p-Sec31p complex of the COPII vesicle coat, required for vesicle formation in ER to Golgi transport
scf184844.g77	An01g04730	YPR181c	SEC23	GTPase-activating protein, stimulates the GTPase activity of Sar1p; component of the Sec23p-Sec24p heterodimer of the COPII vesicle coat, involved in ER to Golgi transport
scf184790.g19	An08g10650	YIL109c	SEC24	Component of the Sec23p-Sec24p heterodimer of the COPII vesicle coat, required for cargo selection during vesicle formation in ER to Golgi transport; homologous to Sfb2p and Sfb3p
scf184473.g15	An16g03320	YHR098c	SFB3	Component of the Sec23p-Sfb3p heterodimer of the COPII vesicle coat, required for cargo selection during vesicle formation in ER to Golgi transport; homologous to Sec24p and Sfb2p
scf184863.g14	An15g01520	YPL085w	SEC16	COPII vesicle coat protein required for ER transport vesicle budding; Sec16p is bound to the periphery of ER membranes and may act to stabilize initial COPII complexes
**Proteins involved in vesicle formation and docking**				
**SNARE proteins**				
scf185002.g61	An12g01190	YPL232w	SSO1/SSO2	Plasma membrane t-SNARE involved in fusion of secretory vesicles at the plasma membrane and in vesicle fusion during sporulation; forms a complex with Sec9p that binds v-SNARE Snc2p
scf185002.g28	An12g01190*	YPL232w	SSO1/SSO2	Plasma membrane t-SNARE involved in fusion of secretory vesicles at the plasma membrane and in vesicle fusion during sporulation; forms a complex with Sec9p that binds v-SNARE Snc2p
scf185039.g6	An07g02170	YLR078c	BOS1	V-SNARE (vesicle specific SNAP receptor), localized to the endoplasmic reticulum membrane and necessary for vesicular transport from the ER to the Golgi
scf185007.g149	An08g02460	YHL031c	GOS1	v-SNARE protein involved in Golgi transport
scf184911.g2	An02g05390	YGR009c	SEC9	T-SNARE protein important for fusion of secretory vesicles with the plasma membrane
No hits found	An07g09960	YIL004c	BET1	Type II membrane protein required for vesicular transport between the endoplasmic reticulum and Golgi complex; v-SNARE with similarity to synaptobrevins
No hits found	An15g01380	YLR268w	SEC22	-SNARE protein; assembles into SNARE complex with Bet1p, Bos1p and Sed5p; cycles between the ER and Golgi complex; involved in anterograde and retrograde transport between the ER and Golgi
scf184994.g13	An04g07020	YOL018c	TLG2	Syntaxin-like t-SNARE that forms a complex with Tlg1p and Vti1p and mediates fusion of endosome-derived vesicles with the late Golgi;
No hits found	An04g01530	YOR036w	PEP12	Target membrane receptor (t-SNARE) for vesicular intermediates traveling between the Golgi apparatus and the vacuole
No hits found	An04g05980	YMR197c	VTI1	Protein involved in cis-Golgi membrane traffic; v-SNARE that interacts with two t-SNARES, Sed5p and Pep12p; required for multiple vacuolar sorting pathways
scf184569.g72	An09g04890	YDR468c	TLG1	Essential t-SNARE that forms a complex with Tlg2p and Vti1p and mediates fusion

				of endosome-derived vesicles with the late Golgi; binds the docking complex VFT (Vps fifty-three) through interaction with Vps51p
scf185016.g92	An04g08480	YKL196c	YKT6	Vesicle membrane protein (v-SNARE) with acyltransferase activity; involved in trafficking to and within the Golgi, endocytic trafficking to the vacuole, and vacuolar fusion; membrane localization due to prenylation at the carboxy-terminus
scf184806.g16	An08g07470	YLR093c	NYV1	v-SNARE component of the vacuolar SNARE complex involved in vesicle fusion; inhibits ATP-dependent Ca(2+) transport activity of Pmc1p in the vacuolar membrane (3, 4 and see *Summary Paragraph*)
scf184806.g6	An12g07570	YAL030w	SNC1	Vesicle membrane receptor protein (v-SNARE) involved in the fusion between Golgi-derived secretory vesicles with the plasma membrane; proposed to be involved in endocytosis; member of the synaptobrevin/VAMP family of R-type v-SNARE proteins
No hits found	An07g05990	YGL212w	VAM7	Vacuolar SNARE protein that functions with Vam3p in vacuolar protein trafficking; has an N-terminal PX domain (phosphoinositide-binding module) that binds PtdIns-3-P and mediates membrane binding; SNAP-25 homolog
scf185013.g106	An02g12980	YLR026c	SED5	cis-Golgi t-SNARE syntaxin required for vesicular transport between the ER and the Golgi complex
No hits found	An04g01530	YOR106w	VAM3	Syntaxin-like vacuolar t-SNARE that functions with Vam7p in vacuolar protein trafficking; mediates docking/fusion of late transport intermediates with the vacuole; has an acidic di-leucine sorting signal and C-terminal transmembrane region
scf185007.g166	An02g06780	YPR032w	SRO7	Effector of Rab GTPase Sec4p, forms a complex with Sec4p and t-SNARE Sec9p; involved in exocytosis and docking and fusion of post-Golgi vesicles with plasma membrane; homolog of Sro77p and Drosophila lgl tumor suppressor
**Secretion related GTPases and interacting proteins**				
scf185001.g27	An14g00010	YFL005w	SEC4	Rab family GTPase essential for vesicle-mediated exocytic secretion and autophagy;
scf184998.g22	An14g00010*	YFL005w	SEC4	Rab family GTPase essential for vesicle-mediated exocytic secretion and autophagy
scf184798.g95	An09g06790	YFL038c	YPT1	Rab family GTPase, involved in the ER-to-Golgi step of the secretory pathway
scf184940.g51	An09g06790*	YFL038c	YPT1	Rab family GTPase, involved in the ER-to-Golgi step of the secretory pathway
scf185000.g56	An01g06060	YER031c	YPT31/32	Rab family GTPase, very similar to Ypt32p; involved in the exocytic pathway; mediates intra-Golgi traffic or the budding of post-Golgi vesicles from the trans-Golgi
scf185014.g102	An15g05740	YLR262c	YPT6	Rab family GTPase, Ras-like GTP binding protein involved in the secretory pathway, required for fusion of endosome-derived vesicles with the late Golgi,
scf185007.g191	An04g02470	YOR089c	YPT51/52/53	Rab family GTPase required for endocytic transport and for sorting of vacuolar hydrolases;
scf184977.g98	An18g02210	YML001w	YPT7	Rab family GTPase; GTP-binding protein of the rab family; required for homotypic fusion event in vacuole inheritance, for endosome-endosome fusion, similar to mammalian Rab7
scf184803.g25	An18g02210*	YML001w	YPT7	Rab family GTPase; GTP-binding protein of the rab family; required for homotypic fusion event in vacuole inheritance, for endosome-endosome fusion, similar to

				mammalian Rab7
scf184977.g48	An02g06400	YOR089c	YPT51/52/53	Rab family GTPase required for endocytic transport and for sorting of vacuolar hydrolases;
scf184798.g95*	An07g10340	YER031c	YPT31	Rab family GTPase, very similar to Ypt32p; involved in the exocytic pathway; mediates intra-Golgi traffic or the budding of post-Golgi vesicles from the trans-Golgi
scf184652.g57	An14g02260	YKR014c	YPT52	Rab family GTPase required for endocytic transport and for sorting of vacuolar hydrolases;
scf185000.g24	An02g10450	YKR001c	VPS1	Dynamin-like GTPase required for vacuolar sorting; also involved in actin cytoskeleton organization, endocytosis, late Golgi-retention of some proteins, regulation of peroxisome
scf184790.g16	An11g09910	YNL272c	SEC2	Guanyl-nucleotide exchange factor for the small G-protein Sec4p
scf185007.g252	An02g03120	YER136w	GDI1	GDP dissociation inhibitor, regulates vesicle traffic in secretory pathways by regulating the dissociation of GDP from the Sec4/Ypt/rab family of GTP binding proteins
scf185002.g129	An01g04040	YPL218w	SARA	GTPase, GTP-binding protein of the ARF family, component of COPII coat of vesicles; required for transport vesicle formation during ER to Golgi protein transport
scf185042.g116	An02g07780	YOR094w	ARF3	Glucose-repressible ADP-ribosylation factor, GTPase of the Ras superfamily involved in development of polarity; also has mRNA binding activity
scf184908.g162	An08g03690<br>An11g01790	YDL137w<br>YDL192w	ARF1<br>ARF2	ADP-ribosylation factor, GTPase of the Ras superfamily involved in regulation of coated formation vesicles in intracellular trafficking within the Golgi; functionally interchangeable with Arf1p
No hits found	An17g00400	YDL226c	GCS1	ADP-ribosylation factor GTPase activating protein (ARF GAP), involved in ER-Golgi transport
scf184798.g65	An18g02490	YEL022w	GEA2	Guanine nucleotide exchange factor for ADP ribosylation factors (ARFs), involved in vesicular transport between the Golgi and ER,
**ER to Golgi and Intra-Golgi transport**				
No hits found	An11g02650	YIL044c	AGE2	ADP-ribosylation factor (ARF) GTPase activating protein (GAP) effector, involved in Trans-Golgi-Network (TGN) transport
scf184829.g71	An08g04350	YHR142w	CHS7	involved in chitin biosynthesis by regulating Chs3p export from the ER
scf184470.g26	An08g03590	YGL200c	EMP24	Component of the p24 complex; binds to GPI anchor proteins and mediates their efficient transport from the ER to the Golgi;
scf184761.g8	An09g05490	YDL018c	ERP3	Protein with similarity to Emp24p and Erv25p, member of the p24 family involved in ER to Golgi transport
scf184977.g160	An07g09160	YGL054c	ERV14	Protein localized to COPII-coated vesicles, involved in vesicle formation and incorporation of specific secretory cargo
scf184844.g135	An01g08870*	YML012w	ERV25	Protein that forms a heterotrimeric complex with Erp1, Erp2p, and Emp24, member of the p24 family involved in endoplasmic reticulum to Golgi transport
scf184921.g7	An08g03960	YGR284c	ERV29	Protein localized to COPII-coated vesicles, involved in vesicle formation and incorporation of specific secretory cargo
scf185031.g6	An03g04940	YML067c	ERV41	Protein localized to COPII-coated vesicles, forms a complex with Erv46p; involved in the membrane fusion stage of transport;
scf184973.g7	An01g04320	YAL042w	ERV46	Protein localized to COPII-coated vesicles, forms a complex with Erv41p; involved in the membrane fusion stage of transport
scf184748.g5	An18g05740	YHR108w	GGA2	Protein that interacts with and regulates

				Arf1p and Arf2p in a GTP-dependent manner to facilitate traffic through the late Golgi;
scf184974.g4	An02g02830	YCL001w	RER1	Protein involved in retention of membrane proteins, including Sec12p, in the ER; localized to Golgi; functions as a retrieval receptor in returning membrane proteins to the ER
scf184908.g122	An07g02190	YDR170c	SEC7	Guanine nucleotide exchange factor (GEF) for ADP ribosylation factors involved in proliferation of the Golgi, intra-Golgi transport and ER-to-Golgi transport
scf184911.g98	An02g01580	YBL050w	SEC17	Peripheral membrane protein required for vesicular transport between ER and Golgi
scf184851.g26	An09g06840	YOR307c	SLY41	Protein involved in ER-to-Golgi transport
scf184888.g15	An08g06780	YDL058w	USO1	Essential protein involved in the vesicle-mediated ER to Golgi transport step
scf184911.g48	An04g04950	YLL040c	VPS13	Protein of unknown function involved in sporulation, vacuolar protein sorting, prospore membrane formation and protein-Golgi retention
scf185042.g105	An14g00210	YGR172c	YIP1	Integral membrane protein required for the biogenesis of ER-derived COPII transport vesicles
scf184656.g7	An18g06440	YNL044w	YIP3	Protein localized to COPII vesicles, proposed to be involved in ER to Golgi transport; interacts with members of the Rab GTPase family and Yip1p
No hits found	An01g08465	YER074w-a	YOS1	Integral membrane protein required for ER to Golgi transport; localized to the Golgi, the ER, and COPII
**Golgi to endosome transport**				
scf184999.g45	An08g01410	YBL102w	SFT2	similar to mammalian syntaxin
scf184970.g40	An07g08220	YJR125c	ENT3	Protein containing an N-terminal epsin-like domain involved in clathrin recruitment and traffic between the Golgi and endosomes
No hits found	An16g03420	YJL004c	SYS1	Integral membrane protein of the Golgi required for targeting of the Arf-like GTPase Arl3p to the Golgi
**Vacuolar protein sorting**				
scf184895.g10	An02g06960	YDR080w	VPS41	Vacuolar membrane protein that is a subunit of the homotypic vacuole fusion and vacuole protein sorting
scf184973.g6	An15g00460	YGL095c	VPS45	Protein of the Sec1p/Munc-18 family, essential for vacuolar protein sorting; required for the function of Pep12p and the early endosome/late Golgi SNARE Tlg2p; essential for fusion of Golgi-derived vesicles with the prevacuolar compartment
scf184766.g20	An14g03790	YDR164c	SEC1	Sm-like protein involved in docking and fusion of exocytic vesicles; binds to assembled SNARE complexes at the membrane and stimulates membrane
scf184920.g34	An16g03010	YPR173c	VPS4	AAA-ATPase involved in multivesicular body (MVB) protein sorting
scf185008.g12	An02g01390	YDR425w	SNX41	Sorting nexin, involved in the retrieval of late-Golgi SNAREs from the post-Golgi endosome to the trans-Golgi network; interacts with Snx4p
scf185007.g93	An02g05380	YLR396c	VPS33	ATP-binding protein that is a subunit of the HOPS complex and of the CORVET tethering complex; essential for protein sorting, vesicle docking and fusion at the vacuole
scf184920.g25	An11g04400	YNR006w	VPS27	Endosomal protein that forms a complex with Hse1p; required for recycling Golgi proteins
scf184583.g15	An14g05130	YPL045w	VPS16	Subunit of the vacuole fusion and protein sorting HOPS complex and the CORVET tethering complex
scf184844.g15	An15g00540	YOL129w	VPS68	Vacuolar membrane protein of unknown function involved in vacuolar

scf184938.g54	An01g08400	YOR069w	VPS5	Nexin-1 homolog required for localizing membrane proteins from a prevacuolar/late endosomal compartment back to the late Golgi apparatus
scf184970.g11	An01g04080	OR359w	VTS1	shows genetic interactions with Vti1p, a v-SNARE involved in cis-Golgi membrane traffic
scf184783.g13	An01g10830	YPL065w	VPS28	Component of the ESCRT-I complex (Stp22p, Srn2p, Vps28p, and Mvb12p
scf185042.g41	An01g04550	YAL002w	VPS8	Membrane-binding component of the CORVET complex; involved in endosomal vesicle tethering and fusion in the endosome to vacuole protein targeting pathway; interacts with
scf185002.g79	An02g09460	YDR323c	PEP7	Multivalent adaptor protein that facilitates vesicle-mediated vacuolar protein sorting by ensuring high-fidelity vesicle docking and fusion
scf184606.g2	An08g01030	YHR012w	VPS29	Endosomal protein that is a subunit of the membrane-associated retromer complex essential for endosome-to-Golgi retrograde transport
scf185008.g79	An01g07920	YML097c	VPS9	A guanine nucleotide exchange factor involved in vesicle-mediated vacuolar protein transport; specifically stimulates the intrinsic guanine nucleotide exchange activity of Vps21p/Rab5
**Cellular export and secretion**				
scf184759.g1	An02g08450	YBR080c	SEC18	ATPase required for vesicular transport between ER and Golgi
scf184813.g9	An02g14450	YGL167c	PMR1	High affinity Ca2+/Mn2+ P-type ATPase required for Ca2+ and Mn2+ transport into Golgi
scf185014.g104	An01g08530	YNL238w	KEX2	Subtilisin-like protease (proprotein convertase), a calcium-dependent serine protease involved in the activation of proproteins of the secretory pathway

Table S10. *P. cinnabarinus* BRFM 137 genome annotation related to protein glycosylation pathways

Pycnoporus cinnabarinus Gene ID	*Aspergillus niger* Gene ID	*Saccharomyces cerevisiae* Gene ID	Gene Name	Description in SGD
**Biosynthesis of nucleotide sugars for glycosylation events**				
**UDP-Glucose**				
scf185043.g193	An12g08610	YCL040w	GLK1	Glucokinase, catalyzes the phosphorylation of glucose at C6
scf184901.g5	An02g14380<br>An15g05940*<br>An16g01620*<br>An06g00380*<br>An13g00510*	YFR053c	HXK1	Hexokinase, catalyzes phosphorylation of glucose
scf184845.g15	An02g07650	YMR105c	PGM2	Phosphoglucomutase, catalyzes the conversion from glucose-1-phosphate to glucose-6-phosphate
scf185032.g4	An07g06780	YMR278w	PGM3	Phosphoglucomutase, catalyzes interconversion of glucose-1-phosphate and glucose-6-phospate;
scf184976.g14<br>scf184665.g5*	An12g00820	YKL035w	UGP1	UDP-glucose pyrophosphorylase, catalyses the reversible formation of UDP-Glc from glucose 1-phosphate and UTP
scf184992.g24	An16g05420	YBR196c	PGI1	Phosphoglucose isomerase, catalyzes the interconversion of glucose-6-phosphate and fructose-6-phosphate
**UDP-N-acetyl-glucosamine**				
scf184790.g17	An18g06820<br>An03g05940*	YKL104c	GFA1	Glutamine-fructose-6-phosphate amidotransferase, catalyzes the formation of glucosamine-6-P and glutamate from fructose-6-P and glutamine.
scf184806.g26	An12g07840	YFL017c	GNA1	Glucosamine-6-phosphate acetyltransferase; involved in UDP-N-acetylglucosamine synthesis, forms GlcNAc6P from AcCoA
scf184911.g99	An18g05160	YEL058w	PCM1	N-acetylglucosamine-phosphate mutase; converts GlcNAc-6-P to GlcNAc-1-P.
scf185007.g57	An12g00480	YDL103c	UAP1	UDP-N-acetylglucosamine pyrophosphorylase, catalyzes the formation of UDP-N-acetylglucosamine (UDP-GlcNAc).
**GDP-mannose**				
scf184934.g4<br>scf185008.g128	An04g03200<br>An08g06350	YER003c	PMI40	Mannose-6-phosphate isomerase, catalyzes the interconversion of fructose-6-P and mannose-6-P;
scf184951.g21	An18g06500	YFL045c	SEC53	Phosphomannomutase, involved in synthesis of GDP-mannose and dolichol-phosphate-mannose
scf185032.g4	An07g06780	YMR278w	PGM3	Phosphoglucomutase, catalyzes interconversion of glucose-1-phosphate and glucose-6-phospate
No hits found (e-value < e-10)	An04g04990	YDL055c	PSA1	GDP-mannose pyrophosphorylase (mannose-1-phosphate guanyltransferase), synthesizes GDP-mannose from GTP and mannose-1-phosphate
No hits found (e-value < e-10)	An11g02380	YDL055c	PSA1	GDP-mannose pyrophosphorylase (mannose-1-phosphate guanyltransferase), synthesizes GDP-mannose from GTP and mannose-1-phosphate
**UDP-galactose**				
scf184979.g64	An16g04160	YBR020w	GAL1	Galactokinase, phosphorylates alpha-D-galactose to alpha-D-galactose-1-phosphate

scf184636.g6	An02g03590	YBR018c	GAL7	Galactose-1-phosphate uridyl transferase, synthesizes glucose-1-phosphate and UDP-galactose from UDP-D-glucose and alpha-D-galactose-1-phosphate
scf184817.g2	An02g09090	YBR019c	GAL10	UDP-glucose-4-epimerase, catalyzes the interconversion of UDP-galactose and UDP-D-glucose in galactose metabolism
scf184753.g44	An14g03820	YBR019c	GAL10	UDP-glucose-4-epimerase, catalyzes the interconversion of UDP-galactose and UDP-D-glucose in galactose metabolism
scf185043.g176	An16g00180	YBR019c	GAL10	UDP-glucose-4-epimerase, catalyzes the interconversion of UDP-galactose and UDP-D-glucose in galactose metabolism
scf184979.g52	An01g11440	-	-	Strong similarity to UDP-glucose-4-epimerase
scf184817.g2	An02g09090	YHR210c	-	sequence similarity to aldose 1-epimerases such as GAL10
No hits found (e-value < e-10)	An02g08660	-	-	UDP-galactopyranose mutase
No hits found (e-value < e-10)	An16g02380	-	-	UDP-galactopyranose mutase
**Transporters of sugar nucleotide donors**				
**GDP-mannose**				
scf184969.g4	An17g02140	YGL225w	VRG4	Golgi GDP-mannose transporter
scf185042.g161	An11g02020	YML038c	YMD8	Putative nucleotide sugar transporter, similar to Vrg4
**UDP-GlcNac**				
scf184969.g10	An03g06940	YEL004w	YEA4	Uridine diphosphate-N-acetylglucosamine transporter required for cell wall chitin synthesis
**UDP-galactose**				
scf184799.g26 scf185014.g127	An08g10400	-	-	Strong similarity to UDP-Gal transporter
scf184649.g22	An18g04260	YPL244c	HUT1	Protein with a role in UDP-galactose transport to the Golgi lumen
**UDP-galactofuranose**				
No hits found (e-value < e-10)	An02g08670	-	-	Putative UDP-galactofuranose transporter
**Oligosaccharyltransferase subunits**				
scf184908.g89	An02g14560	YJL002c	OST1	Subunit of the OST complex of the ER lumen
scf184569.g15	An04g03495	YMR149w	SWP1	Subunit of the OST complex of the ER lumen
scf184940.g5	An07g04190	YEL002c	WBP1	Subunit of the OST complex of the ER lumen
scf185043.g188	An18g03920	YOR103c	OST2	Subunit of the OST complex of the ER lumen
scf184863.g13	An02g14930	YOR085w	OST3	Subunit of the OST complex of the ER lumen
scf184805.g41	An16g08570	YGL022w	SST3	Subunit of the OST complex of the ER lumen
No hits found (e-value < e-10)	An08g07485	YDL232W	OST4	Subunit of the OST complex of the ER lumen
**Synthesis of the dolicholphosphate linked ER-precursor Glc3Man9GlcNAc2**				
scf184931.g2	An04g03960	YMR013c	SEC59	Dolichol kinase, catalyzes the terminal step in dolichyl monophosphate (Dol-P) biosynthesis
scf184806.g20	An16g04330	YPR183w	DPM1	Dol-P-Man synthase of the ER membrane, catalyzes the formation of Dol-P-Man from Dol-P and GDP-Man
No hits found (e-value < e-10)	An01g05200	-	-	Dolichol phosphate-mannose biosynthesis regulatory
No hits found	An14g00270	-	-	Dolichol-phosphate

(e-value < e-10)				mannosyltransferase subunit 3
No hits found (e-value < e-10)	An03g04410	YPL227c	ALG5	UDP-glucose:dolichyl-phosphate glucosyltransferase
scf184801.g52	An02g03240	YBR243c	ALG7	UDP-N-acetyl-glucosamine-1-P transferase, transfers Glc-Nac-P from UDP-GlcNac to Dol-P
scf184785.g46	An01g09110	YGL047w	ALG13	Catalytic component of UDP-GlcNAc transferase, required for the second step of dolichyl-linked oligosaccharide synthesis;
scf185001.g38	An06g01100	YBR110w	ALG1	Mannosyltransferase, involved in asparagine-linked glycosylation in the endoplasmic reticulum
scf184940.g36	An14g05910	YGL065c	ALG2	Mannosyltransferase that catalyzes two consecutive steps in the N-linked glycosylation pathway
scf184970.g62	An18g05910	YNL048w	ALG11	Alpha-1,2-mannosyltransferase, catalyzes sequential addition of the two terminal alpha 1,2-mannose residues
		YBL020w	RFT1	translocation of Man5GlcNac2-PP-Dol from the cytoplasmic side to the lumenal side of the ER membrane but is not the flippase
scf184801.g4	An04g03130	-	-	Strong similarity to flippase = flipping the precursor Oligosaccharide
scf185002.g48	An18g02360	YBL082c	ALG3	Dolichol-P-Man dependent alpha(1-3) mannosyltransferase
scf185007.g234	An02g14940		ALG9	both the transfer of seventh mannose residue on B-arm and ninth mannose residue on the C-arm from Dol-P-Man to lipid-linked oligosaccharides
scf184939.g28	An01g08460	YNR030w	ALG12	Alpha-1,6-mannosyltransferase localized to the ER; responsible for the addition of the alpha-1,6 mannose to dolichol-linked Man7GlcNAc2
scf184909.g11	An02g12630	YOR002w	ALG6	Alpha 1,3 glucosyltransferase, involved in transfer of oligosaccharides from dolichyl pyrophosphate to asparagine
scf184652.g33	An04g08820	YOR067c	ALG8	Glucosyl transferase, involved in N-linked glycosylation; adds glucose to the dolichol-linked oligosaccharide precursor prior to transfer to protein during lipid-linked oligosaccharide biosynthesis; similar to Alg6p
scf184672.g13	An02g02980	YGR227w	ALG10	Dolichyl-phosphoglucose-dependent alpha-1,2 glucosyltransferase of the ER, functions in the pathway that synthesizes the dolichol-linked oligosaccharide precursor for N-linked protein glycosylation
scf184857.g35*	An15g01460	YGR036C	CWH8	Dolichyl pyrophosphate (Dol-P-P) phosphatase
**Processing of the ER-precursor Glc3Man9GlcNAc2 after transfer to a polypeptide**				
scf184750.g6	An15g01420	YGL027c	CWH41	Processing alpha glucosidase I, ER type II
scf184985.g33	An09g05880	YBR229c	ROT2	Glucosidase II catalytic subunit
scf184940.g83	An01g10930	YBR229c	ROT2	Glucosidase II catalytic subunit
scf185022.g7	An04g06920	YBR229c	ROT2	Glucosidase II catalytic subunit
scf184603.g6	An04g06920*	YBR229c	ROT2	Glucosidase II catalytic subunit
scf184798.g2	An01g04880	YBR229c	ROT2	Glucosidase II catalytic subunit
scf185043.g220	An13g00620	YDR221w	GTB1	Glucosidase II beta subunit,
scf184763.g4	An18g06220	YJR131w	MNS1	α-1,2-mannosidase involved in ERAD
scf184815.g42	An04g06990	YJR131w	MNS1	α-1,2-mannosidase involved in ERAD
scf184815.g58	An04g06990*	YJR131w	MNS1	α-1,2-mannosidase involved in ERAD
scf184569.g13	An01g12550	YJR131w	MNS1	α-1,2-mannosidase involved in ERAD
No bidirectional best hit	An06g01510	YLR057w	MNL2	Putative mannosidase involved in ERAD
scf185043.g124	An12g00340	YHR204w	MNL1	Alpha-1,2-specific exomannosidase of the endoplasmic reticulum
**Golgi mannosyltransferase**				

No hits found (e-value < e-10)	An03g01090 An05g01750 An05g02320 An11g07490 An12g07020 An14g07140	YJR075w	HOC1	α-1,6-mannosyltransferase involved in cell wall mannan biosynthesis
No hits found (e-value < e-10)	An07g04940	YGL038c	OCH1	Mannosyltransferase of the cis-Golgi apparatus, initiates the mannose outer chain elongation of N-linked oligosaccharides
No hits found (e-value < e-10)	An03g05010	YPL050c	MNN9	Subunit of the alpha-1,6 mannosyltransferase complex
No hits found (e-value < e-10)	An04g01260	YEL036c	ANP1	Subunit of the alpha-1,6 mannosyltransferase complex
No hits found (e-value < e-10)	An15g06230 An15g03330	YDR245w	MNN10	Subunit of the alpha-1,6 mannosyltransferase complex
No hits found (e-value < e-10)	An04g05940	YJL183w	MNN11	Subunit of the alpha-1,6 mannosyltransferase complex
No hits found (e-value < e-10)	An04g06730 An14g06060 An15g00920	YBR015c	MNN2	α-1,2-mannosyltransferase, responsible for addition of the first α- -1,2-linked mannose
**O-Glycosylation in ER**				
scf184588.g3	An11g09890	YDL095w	PMT1	Protein O-mannosyltransferase, transfers mannose residues from dolichyl phosphate-D-mannose to protein Ser/Thr residues;
scf184996.g57	An07g10350	YAL023c YOR321w	PMT2 PMT3	Protein O-mannosyltransferase, transfers mannose residues from dolichyl phosphate-D-mannose to protein Ser/Thr residues;
scf184938.g56	An16g08490	YJR143c	PMT4	Protein O-mannosyltransferase, transfers mannose residues from dolichyl phosphate-D-mannose to protein Ser/Thr residues;
**Other genes possibly involved in glycosylation events**				
scf185033.g56 scf184863.g22* scf184943.g72*	An14g03910	YOR099w	KRE2family	Alpha-1,2-mannosyltransferase involved in O- and N-linked protein glycosylation
No hits found (e-value < e-10)	An18g03940 An08g05380	YKL201c	MNN4	Putative positive regulator of mannosylphosphate transferase (Mnn6p), involved in mannosylphosphorylation of N-linked oligosaccharides
No hits found (e-value < e-10)	An15g04810	YIL014w	MNT3	Alpha-1,3-mannosyltransferase, adds the fourth and fifth alpha-1,3-linked mannose residues to O-linked
No hits found (e-value < e-10)	An03g02990 An11g10260	YOR320c	GNT1	N-acetylglucosaminyltransferase capable of modification of N-linked glycans in the Golgi apparatus
**Other putative alpha-1,2-mannosidase (no homology to the MNS1/ER-alpha-1,2-mannosidase family**				
scf184969.g71	An08g03060	-	-	putative alpha-1,2-mannosidase
scf184501.g5	An08g03060*	-	-	putative alpha-1,2-mannosidase
scf184969.g74	An13g01260*	-	-	putative alpha-1,2-mannosidase
scf184817.g11	An14g04240*	-	-	putative alpha-1,2-mannosidase
scf184817.g13	An14g04240*	-	-	putative alpha-1,2-mannosidase
scf184590.g13	An02g11720	YGL156w	AMS1	Vacuolar alpha mannosidase, involved in free oligosaccharide (fos) degradation
**GPI ANCHOR BIOSYNTHESIS**				
scf184996.g52	An16g03530	YGR216C	GPI1	Protein involved in the synthesis of GlcNAc-PI
No hits found (e-value < e-10)	An02g13570	YPL076W	GPI2	Protein involved in the synthesis of GlcNAc-PI
scf184693.g7	An01g09910	YPL175W	GPI3	Protein involved in the synthesis of GlcNAc-PI
No hits found (e-value < e-10) (e-value < e-10)	An02g09230	YNL038W	GPI15	Protein involved in the synthesis of GlcNAc-PI
scf184830.g9	An16g01530	YDR437W	GPI19	Protein involved in the synthesis of GlcNAc-PI

scf184569.g56	An14g06640	YMR281W	GPI12	Protein involved in the second step of GPI anchor assembly, the de-N-acetylation of the N-acetylglucosaminyl-phosphatidylinositol
scf184969.g82	An01g12990	YJL091C	GWT1	Protein involved in the inositol acylation of glucosaminyl phosphatidylinositol (GlcN-PI) to form glucosaminyl(acyl)phosphatidylinositol (GlcN(acyl)PI
scf185002.g128	An12g01880	YJR013W	GPI14	GPI-alpha-1,4 mannosyltransferase I
No hits found (e-value < e-10)	An17g00780	YCL052C	PBN1	Essential component of GPI-mannosyltransferase I,
scf184836.g39	An14g00900	YKL165C	MCD4	multimembrane-spanning protein that localizes to the ER
scf184830.g9	An16g01530		GPI18	mannosyltransferase that transfers the second mannose in glycosylphosphatidylinositol biosynthesis
scf185014.g36	An04g04110	YGL142C	GPI10	putative alpha 1,2 mannosyltransferase required for addition of the third mannose onto the GPI core structure
No hits found (e-value < e-10)	An10g00480	YKL165C	GPI11	involved in the addition of phosphoethanolamine to the multiply mannosylated GPI intermediate
scf185033.g18	An09g02800<br>An04g05100	YLL031C	GPI13	Phosphoryltransferase that adds phosphoethanolamine onto the third mannose residue of the GPI-anchor precursor
**GPI-anchor transamidase complex; removes the GPI-Anchoring signal and attaches GPI to proteins in the ER**				
No hit found	An08g10720	YLR088W	GAA1	Subunit of the GPI (glycosylphosphatidylinositol):protein transamidase complex; ScGaa1-like
scf184915.g49	An04g02650	YLR459W	GAB1	Subunit of the GPI protein transamidase complex
scf184970.g112	An11g06770	YHR188C	GPI16	Subunit of the GPI protein transamidase complex
scf184830.g15	An07g09270	YDR434W	GPI17	Subunit of the GPI protein transamidase complex
scf184943.g62	An01g13530	YDR331W	GPI8	Subunit of the GPI protein transamidase complex

Table S11. Propagation of functional annotations for predicted proteins involved in the secretory pathway, from *P. cinnabarinus* BRFM 137 to *P. coccineus* BRFM 310 and *P. sanguineus* BRFM 1264. Putative orthologs of *A. niger* and *P. cinnabarinus* genes were identified using orthoMCL.

***P. cinnabarinus* ProtID**	***P. coccineus* ProtID**	***P. sanguineus* ProtID**	***Aspergillus niger* Gene ID**	***P. cinnabarinus* ProtID**	***P. coccineus* ProtID**	***P. sanguineus* ProtID**	***Aspergillus niger* Gene ID**
**ENTRY INTO ER**							
**SIGNAL RECOGNITION**							
1173	926219	1759759	An15g06470	2940	1502961	1570754	An15g01670
2616	1196039	1591884	An09g06320				
**SIGNAL PEPTIDASE COMPLEX**							
8005	1437377	1586001	An01g00560	1696	1400232	1667503	An09g05420*
988	1432946	1587010	An16g07390				
**TRANSLOCATION INTO ER**							
1783	1428811	1586282	An03g04340	272	1435989	1586785	An01g13070
8579	1467116	1674431	An01g11630	4482	1438656	1590928	An16g08830
840	1397615	1624424	An02g01510				
**PROTEIN FOLDING IN THE ER**							
4404	1364171	1666656	An02g14800	2811	1439793	1331091	An12g06490
3941	1423552	1725653	An18g02020	2812	1465891	1583486	An12g06490
3941	1423552	1725653	An01g04600	2813	1465892	1660671	An12g06490
4404	1364171	1666656	An01g04600	2814	1465893	1681323	An12g06490
1077	1435980	1580621	An02g05890	2850	1404520	1654700	An05g00880
2453	1431052	1573162	An16g07620	8751	1431989	1584617	An01g08420
310	1455348	1662505	An08g07810	6742	766953	1066844	An04g02020
9511	1363828	1561526	An08g06370	5522	1382917	1651699	An01g06670
4926	1430717	1580268	An10g00350	4946	1360346	1658898	An01g13220
**PROTEIN MISFOLDING**							
**UNFOLDED PROTEIN RESPONSE (UPR)**							
2483	1372612	1586082	An01g06550	5755	1389718	1594359	An08g01480
4309	1359382	1601091	An14g04770	3438	1368404	1587320	An11g11250
4309	1359382	1601091	An14g04770	2262	1433362	1648617	An01g08980
5233	1422036	1669227	An08g00830				
**ER ASSOCIATED DEGRADATION (ERAD)**							
3225	1432544	1593418	An04g09170	4199	1433145	1595947	An04g01720
3225	1432544	1593418	An04g09170	10267	1446926	1650951	An12g00340
3225	1432544	1593418	An04g09170	2338	1436992	1589591	An18g06220
7802	1384960	1614133	An01g05330	1293	1435665	1590331	An04g00360
8468	1380333	1762115	An01g05760	1293	1435665	1590331	An04g00360
9381	1356617	1568053	An03g04600	1293	1435665	1590331	An04g00360
585	1436621	1598876	An08g09000	5614	1405960	1667072	An12g04000
1592	1399393	1634577	An16g07970	6621	1443233	1667037	An14g00230
6998	1375679	1566790	An17g00260	1158	1444845,137	1762980,159	An01g04280
8138	1434079	1637572	An09g06110	2115	1495369	1588982	An15g01420
5211	1430507	1597396	An04g01730				
**PROTEASOME**							
26	1430290	1611703	An18g03010	1080	1435975	1596662	An15g03020
9267	1438272,1415[illegible]	1632606,158	An04g03270*	5106	1378402	1589287	An07g07860
697	1436421	1589047	An11g10380	1299	1433765	1599283	An16g02210
9228	1441061	1655832	An11g09690	931	1410209	1615805	An02g12760
4913	1379750	1754238	An18g05070	2233	1383761	1589738	An17g00270
4913	1379750	1754238	An18g05070	2649	1438699	1611530	An02g07190

4913	1379750	1754238	An18g05070	3362	1443569,146	1573703,159	An18g06230
4912	1429350	1587409	An11g02610	5582	1430665	1581717	An18g05230
8016	1356593	1756741	An07g10110	4075	1367082	1654390	An14g00180
2716	1451499	1594001	An08g10710				

Protein complex involved in protein transport

Exocyst Complex

8600	1434698	1588012	An01g03190	8313	1464965	1562308	An14g00010
5700	1448893	1610523	An08g05570	8313	1464965	1562308	An14g00010
6633	1450449	1593292	An04g06180	8313	1464965	1562308	An14g00010
8614	1444640	1607095	An03g04210	7452	1447569	1614641	An18g05980
8504	1374049	1589689	An03g06900	7452	1447569	1614641	An16g04200
5186	1416836	1589714	An15g00010	8834	1434480	1595597	An11g10030
5754	1435235	1594357	An02g04030*	8249	1468024	1595999	An02g14200
2179	1392308	1588859	An08g07370				

SEC34/SEC35 Complex

858	1408645	1594225	An02g06840	9407	1380049,142	1567970,167	An06g01630
3719	1440033	1661994	An02g14400				

Trapp Complex

3032	1436176	1570824	An12g00380	8412	1429757	1674998	An08g05190
7322	1430961	1072453	An17g01875	3577	1432332	1579493	An14g06440
4476	1454002	1590932	An15g00470	3607	1405579	1657332	An15g00060

COPI SUBUNITS

6993	1439546	1590032	An16g05370	8658	1434766	1660573	An14g03050
703	1436411	1609709	An16g02460	8659	148	158	An14g03050
4145	1398995	1677400	An12g04830	540	1480987	1762275	An02g05870
8657	1434766	1660573	An07g06030	5187	1416841	1684505	An08g03690
8658	148	158	An07g06030	5187	1416841	1684505	An08g03690

COPII SUBUNITS

8482	1455645	1669467	An01g04040	1293	1435665	1590331	An04g00360
8482	1455645	1669467	An01g04040	8354	1410552	1575621	An02g01690
8796	1431934	1584675	An08g03270	3995	1432848	1574864	An01g04730
1293	1435665	1590331	An04g00360	2747	1418878	1648061	An08g10650
1293	1435665	1590331	An04g00360	471	1436293	1589218	An16g03320

Proteins involved in vesicle formation and docking

SNARE proteins

8414	1429754	1665564	An12g01190	751	1452759	1676180	An09g04890
8414	1429754	1665564	An12g01190	9703	1429898	1560808	An04g08480
9935	1440528		An07g02170	3265	1381265	1676346	An08g07470
8714	1475049	1584365	An08g02460	3255	1430186	1571608	An12g07570
5317	1434370	1592993	An02g05390	9236	1460702	1595297	An02g12980
7895	1436456	1664758	An04g07020				

Secretion related GTPases and interacting proteins

8313	1464965	1562308	An14g00010	2948	490186	1715902	An07g10340
8313	1464965	1562308	An14g00010	8215	1440947	1598831	An02g10450
8313	1464965	1562308	An14g00010	8817	1388509	1595619	An02g03120
2948	490186	1715902	An09g06790	8482	1455645	1669467	An01g04040
2948	490186	1715902	An09g06790	8482	1455645	1669467	An01g04040
8247	436301	1595998	An01g06060	10076	1449785	1609536	An02g07780
9398	1400929	1624095	An15g05740	5187	1416841	1684505	An08g03690
8756	1431983	1638299	An04g02470	5187	1416841	1684505	An11g01790
7173	1474966	1598574	An02g06400	2918	1436276	1757574	An18g02490
2948	490186	1715902	An07g10340				

ER to Golgi and Intra-Golgi transport

3603	1361443	1634013	An08g04350	5147	1374061	1587024	An07g02190
440	1433682	1572526	An08g03590	5413	1388657	1586411	An02g01580
2318	1437458	1596771	An09g05490	4215	1407062	1591174	An09g06840
7285	1437967	1591373	An07g09160	4864	1440814	1620364	An08g06780
5691	1429824	1599614	An08g03960	5363	1476833	1573266	An04g04950
9803	1440641	1563142	An03g04940	10065	1404003	1755266	An14g00210
2079	1402223	1718643	An18g05740	1385	1431041	1651863	An18g06440
7069	1437585,144	1612535	An02g02830				

Golgi to endosome transport

8144	1434088	1583641	An08g01410				

Vacuolar protein sorting

4909	1357177	91729	An02g06960	3933	1432757	1630083	An15g00540
2363	1433097	1666957	An14g03790	5960	1434809	1571147	An01g08400
5664	1432231	1709987	An16g03010	6886	1390003	1591234	An01g04080
8871	1434877	1587817	An02g01390	2582	1533547	1652823	An01g10830
8872	143	161	An02g01390	10001	1359573	1588652	An01g04550
8658	1434771	1615166	An02g05380	1046	1435211	1575706	An08g01030
5655	1384658	1567213	An11g04400	8938	1432665	1591446	An01g07920
852	1439587	1590752	An14g05130				

Cellular export and secretion

2254	1433370	1625091	An02g08450	9400	1400922	1647634	An01g08530
3327	1481236	1595936	An02g14450				

Table S12. Propagation of functional annotations from *P. cinnabarinus* BRFM 137 to *P. coccineus* BRFM 310 and *P. sanguineus* BRFM 1264 predicted proteins involved in the glycosylation pathway. Putative orthologs of *Aspergillus niger* and *P. cinnabarinus* genes were identified using orthoMCL.

P. cinnabarinus ProtID	*P. coccineus* ProtID	*P. sanguineus* ProtID	*Aspergillus niger* Gene ID	*P. cinnabarinus* ProtID	*P. coccineus* ProtID	*P. sanguineus* ProtID	*Aspergillus niger* *Gene ID*
**Biosynthesis of nucleotide sugars for glycosylation events**							
**UDP-Glucose**							
10336	1368804	1592515	An12g08610	7101	174808	159417	An12g00820
4933	1373367	1672395	An02g14380	1499	1451391	1594181	An12g00820
4068	1367276	1594406	An02g07650	7833	1401869	1597689	An16g05420
9809	1435058	1639174	An07g06780				
**UDP-N-acetyl-glucosamine**							
2745	1439886	1665538	An18g06820	5414	1434563	1623225	An18g05160
3275	1402152	1592322	An12g07840	8622	1409673	1320524	An12g00480
**UDP-galactose**							
7381	1112144	1759901	An16g04160	10319	1436108	1603610	An16g00180
1154	1366875	1598930	An02g03590	7369	1439453	1667810	An01g11440
3419	1368320	1560516	An02g09090	3419	1368320	1560516	An02g09090
2181	1392304	1620883	An14g03820				
**Transporters of sugar nucleotide donors**							
**GDP-mannose**							
6790	1434910	603179	An17g02140	10121	1387457	1621988	An11g02020
**UDP-GlcNac**							
6796	1409975	1591552	An03g06940				
**UDP-galactose**							
9423	1356958	1539772	An08g10400	1276	1506075	1597537	An18g04260
**Oligosaccharyltransferase subunits**							
5114	1399819	1621646	An02g14560	10331	1436095	1592507	An18g03920
694	1391914	1564546	An04g03495	4469	1470518	1586231	An02g14930
6052	1435743	1596031	An07g04190	3234	1432529	1758864	An16g08570
**Synthesis of the dolicholphosphate linked ER-precursor Glc3Man9GlcNAc2**							
5772	1404216	1584185	An04g03960	3068	1430879	1657625	An04g03130
3269	1381272	690541	An16g04330	8401	1429769	1624673	An18g02360
3116	1430821	1598186	An02g03240	5997	1418951	1594869	An01g08460
2651	1470544	647126	An01g09110	5201	1358493	1614786	An02g12630
8324	1405373	1567721	An06g01100	1328	1433732	1662999	An04g08820
6083	1411479	1614029	An14g05910	1551	1361065	1663951	An02g02980
6937	1376072	1566867	An18g05910	4357	1413398	1595833	An15g01460

Processing of the ER-precursor Glc3Man9GlcNAc2 after transfer to a polypeptide

2115	1495369	1588982	An15g01420	2855	1435128	1591617	An01g04880
7576	1417756	1676227	An09g05880	10363	1478212	1627739	An13g00620
6130	1426153	1574406	An01g10930	2338	1436992	1589591	An18g06220
9738	1361992	1593698	An04g06920	3385	1465680	1573901	An04g06990
9738	1361992	1593698	An04g06920	692	1369367	1609201	An01g12550
1027	1361884	1559723	An04g06920	10267	1446926	1650951	An12g00340
1027	1361884	1559723	An04g06920				

O-Glycosylation in ER

887	1368781	1586791	An11g09890	5962	1434813	1685864	An16g08490
8012	1400787	1624254	An07g10350				

Other genes possibly involved in glycosylation events

9868	1446713	1595128	An14g03910	6361	1438184	1730991	An14g03910
4478	1453999	1685184	An14g03910				

Other putative alpha-1,2-mannosidase (no homology to the MNS1/ER-alpha-1,2-mannosidase family

6857	1373467	1609029	An08g03060	6860	1453868	1663870	An13g01260*
6857	1373467	1609029	An08g03060	6860	1453868	1663870	An13g01260*
598	1514584	1721241	An08g03060	903	1480666	1760376	An02g11720
598	1514584	1721241	An08g03060				

GPI ANCHOR BIOSYNTHESIS

8007	1414257	1639580	An16g03530	735	1437856	1666335	An14g06640
1667	1441575	1671008	An01g09910	6868	1395445	1562231	An01g12990
3619	1362938	1550771	An16g01530	8481	1473188	1660339	An12g01880
3783	1388125	1575012	An14g00900	9332	1473154	1597904	An04g04110
3619	1362938	1550771	An16g01530	9830	1480848	1647113	An09g02800/An04g05100
9332	1473155	1582986	An04g04110				

GPI-anchor transamidase complex; removes the GPI-Anchoring signal and attaches GPI to proteins in the ER

5571	1430652	1761677	An04g02650	3625	1432983	1647900	An07g09270
6987	1439536	1590021	An11g06770	6351	1372810	1613432	An01g13530

Table S13. Number of predicted Cyt450 genes up-regulated after 3 day growth of the three *Pycnoporus* strains on Avicel, wheat straw, pine or aspen.

	P. cinnabarinus **BRFM 137**	***P. coccineus*** **BRFM 310**	***P. sanguineus*** **BRFM 1264**
CYP53	1	-	-
CYP63	1	1	-
CYP512	-	3	-
CYP620	1	-	-
CYP5035	-	1	1
CYP5136	1	2	-
CYP5139	2	2	-
CYP5144	3	3	-
CYP5150	4	2	-
CYP5151	-	1	-
CYP5152	1	-	-
CYP5157	-	1	-
unclassified	-	1	5
total	14	17	6

Table S14. Gene count for predicted peptidases in the genomes of *P. cinnabarinus* BRFM 137, *P. coccineus* B and *P. sanguineus* BRFM 1264.

	P. cinnabarinus	*P. coccineus*	*P. sanguineus*
**Aspartic peptidases**			
Peptidase A1	42	41	47
Peptidase A22	1	1	1
Peptidase aspartic	50	17	19
**Total**	**93**	**59**	**67**
**Cystein Peptidases**			
Peptidase C12	3	22	31
Peptidase C13	1	3	3
Peptidase C14	5	1	1
Peptidase C14	1	7	9
Peptidase C15	1	1	1
Peptidase C19	16	17	16
Peptidase C1B	1	1	1
Peptidase C2	1	1	1
Peptidase C45	1	1	1
Peptidase C48	4	7	5
Peptidase C50	1	1	1
Peptidase C54	1	1	1
Peptidase C78	1	1	1
**Total**	**37**	**64**	**72**
**Metallo Peptidases**			
Peptidase M	22	27	24
Peptidase M1	2	1	2
Peptidase M10A and M1:	0	1	1
Peptidase M14	8	7	7
Peptidase M16	6	7	6
Peptidase M17	2	1	1
Peptidase M18	1	1	1
Peptidase M19	1	1	1
Peptidase M20	10	9	9
Peptidase M22	2	2	2
Peptidase M24	13	12	14
Peptidase M28	7	11	10
Peptidase M35	1	4	4
Peptidase M36	1	1	1
Peptidase M41	2	3	2
Peptidase M43B	2	2	2
Peptidase M48	1	0	0
Peptidase M49	1	1	1
Peptidase M50	0	1	1
**Total**	**82**	**92**	**89**

	Pycci	Pycco	Pycsa
**(C, S, T) Catalytic Type**			
Peptidase S1 and S6	3	3	2
Peptidase S58 DmpA/arg	2	3	2
Peptidase T1A	2	3	3
Peptidase T2	1	2	2
**Total**	**8**	**11**	**9**
**Serine Peptidases**			
Peptidase S10	20	20	20
Peptidase S14	0	1	1
Peptidase S15	1	1	
Peptidase S16	3	4	4
Peptidase S24	3	3	3
Peptidase S26A	6	5	4
Peptidase S28	5	7	7
Peptidase S33	13	16	14
Peptidase S41	1	1	1
Peptidase S53	21	24	25
Peptidase S54	3	3	3
Peptidase S59	1	1	1
Peptidase S8 and S53	12	11	12
Peptidase S9	4	4	4
Peptidase S9A	0	1	4
**Total**	**93**	**102**	**103**
**Glutamic Peptidases**			
Peptidase G1	1	2	2
other	7	10	12
**Total**	**321**	**340**	**354**

Table S15. Numbers of peptidases identified in at least one secretome obtained from *P. cinnabarinus* BRFM 137, *P. coccineus* BRFM 310 and *P. sanguineus* BRFM 1264 grown on maltose, Avicel, wheat straw, aspen or pine.

Peptidase family	*P. cinnabarinus*	*P. coccineus*	*P. sanguineus*
Peptidase S53	11	14	8
Peptidase S41	1	0	1
Peptidase S33	1	0	2
Peptidase S28	2	1	1
Peptidase S10	9	10	11
Peptidase M36	1	1	1
Peptidase M35	1	1	2
Peptidase M28	1	0	0
Peptidase G1	1	1	0
Peptidase A1	8	8	13

Table S16. List of proteins detected in the secretomes of *P. cinnabarinus* BRFM 137 grown on Aspen (As), Pine (P), wheat straw (W), Avicel (A) or maltose (M).

Pycci1 protID	secretome				expert_annot	Pycci1 protID	secretome				expert_annot
2284		W			Peptidase S53	2356			A		Peptidase S10
6931	As			M	GH37	3507	As	W			Peptidase S10
929	As	W	A	M	GH95	2682	As		A	M	Peptidase S10
1092	As	W	A	M	GH2	8552	As		A	M	GH27
4566	As	W		M	GH3	8673				M	GH3
6130	As	W		M	GH31	6687		W		M	Peptidase S33
8219		W			GH88	8256	As	W	A	M	GH5_9
9738	As	W	A	M	GH31	8257	As	W	A	M	GH5_9
6860	As	W	A	M	GH92	289		W			Peptidase S28
3446	As	W	A	M	AA1_1	9504	As	W			GH16
3798	As	W			GH28	10110	As	W	A		PL14_5
598	As	W	A	M	GH92	2469	As	W	A	M	Peptidase A1
6051		W			EXPN	3097	As	W	A		GH16
692		W			GH47	3438		W			AA9
7245	As	W		M	GH27	7745			A		GH16
7421	As	W	A	M	GH55	8439	As	W	A		GH5_9
8220		W			GH88	3447		W			GH79
10401		W			EXPN	8440	As		A		GH55
7004	As	W	A	M	GH35	2095	As	W			GH51
8001	As	W	A		GH28	3220		W			GH5_15
8672	As	W	A	M	AA1_1	4254		W	A		Peptidase S53
1412	As	W	A	M	GH30_3	6253	As		A		GH18-CBM5-CBM5
7188		W			GH131	4028	As	W			Peptidase S41
5993					GH18	68					Peptidase S10
7927		W			GH5_9	6090		W	A		cerato-platanin
1111	As	W	A	M	GH15	6879		W			GH25
1686		W			GH2	9003		W		M	AA5_1
7538	As	W	A	M	GH16	1104		W			CBM1
7546	As	W	A		GH28	372	As	W	A		GH28
2037		W		M	Peptidase A1	7494		W			CBM1-GH18
5992		W			GH18-CBM5	1070	As	W		M	AA3_2
7820	As	W		M	GH13_32-CBM20	3428		W		M	GH92
8665	As	W	A		AA1_1	5675	As	W			Peptidase S28
6914		W		M	GH20	5902	As				Peptidase S53
7818		W			GH18-CBM5	8810		W			Peptidase M35
1433	As	W	A	M	GH30_3	6929	As	W	A	M	Peptidase A1
1644	As	W	A		GH18	8374	As	W			Peptidase M36
2129	As	W			CE16	2943	As	W	A		GH43-CBM35
3435	As	W			CBM1-GH10	2209	As		A		Peptidase S10
7817		W			GH18-CBM5	9091			A		Peptidase S10
7835	As				Peptidase S53	3884			A		EXPN
8611	As			M	GH20	1194	As	W	A	M	Peptidase S53
3542	As	W	A		GH16	3613	As	W	A	M	CBM18-GH16
4712		W			Peptidase S53	4099	As	W	A	M	GH18
4963			A		GH16	5321		W			PL8_4
5298	As	W	A	M	Peptidase A1	6327	As	W			Peptidase G1
1984	As	W	A	M	AA3_2	689		W			Peptidase S53
2012			A		GH18	7022		W			GH35
2360			A		Peptidase S10	7984	As		A	M	GH28
301	As	W	A		GH16	5750	As	W	A		GH76
3147	As	W	A	M	AA3_2	5751	As	W	A		GH76
5320	As	W	A	M	PL8_4	826	As		A		GH28
7415	As	W			GH17	5324				M	PL4
7620		W			GH5_9	5626	As	W	A	M	AA1_1
7844	As	W	A		Peptidase S53	10014	As	W	A	M	Peptidase A1
4229	As	W	A	M	Peptidase S53						
172	As	W	A	M	GH18						
6211	As	W	A		CBM50-CBM50						

6235	As	W	A		GH72-CBM43
90		W			Peptidase S53
1027	As	W	A	M	GH31
3359		W			GH45
161	As	W			GH30
6857	As		A	M	GH92
3589	As				Peptidase S10
5385	As	W			Peptidase S8 and S53
5520		W		M	AA3_4
10255	As	W		M	GH125
1233	As	W			GH17
5804		W			GH16
6004		W			GH18
6608	As	W	A		CBM1-GH5_7
9131	As	W			AA8-AA3_1
1365	As	W	A		GH131-CBM1
1929	As	W	A		CBM1-GH5_5
5579		W			AA9-CBM1
5845	As	W	A		GH7
6605	As	W	A	M	Peptidase S10
8616	As	W	A		CBM1-GH5_5
1643	As	W	A	M	GH18
465	As				Peptidase M28
1423	As	W	A	M	Peptidase A1
3171	As	W	A	M	Peptidase A1
6236	As	W	A		CE4
7854	As	W	A	M	AA5_1
4408		W			Peptidase A1
2028		W			AA9-CBM1
6809	As	W	A		GH7
7129	As	W	A		GH74-CBM1
8640	As	W	A	M	GH7
9352		W	A		CBM1-GH6
3646		W			AA9
4319		W			CBM1-CE15
4523	As	W	A		GH10
4524	As	W	A		CBM1-GH10
5150		W			AA9
5713		W	A		CBM1-GH5_5
5863		W			CE16
6994		W	A		GH12
10062		W			AA9
4047	As	W	A		AA9
4521	As	W	A		CBM1-GH10
6822		W			CBM1-CE1
7225	As	W	A		GH3
7436	As	W	A		CBM1-CE1
8853	As	W	A		GH5_7
9908		W			AA9
3282		W			GH12
9246	As	W			CE8

Table S17. List of proteins detected in the secretomes of *P. coccineus* BRFM 310 grown on Aspen (As), Pine (P), wheat straw (W), Avicel (A) or maltose (M).

Pycco1 protID	secretome				expert_annot
1395316	As				GH10
1370947	As	W	A	M	Peptidase A1
1372016	As				GH16
1440257	As	W	A	M	GH27
1445051	As	W			GH18
1507511	As	W	A	M	GH15-CBM20
651762					GH16
672392		W			Peptidase S53
1436321		W			AA2; MnP-short
1357629	As	W	A	M	GH51
1439531	As	W	A	M	GH35
1423919		W			Peptidase S53
1438837	As				Peptidase S53
1434777				M	AA1_1
1418425					GH20
1425989				M	Peptidase S53
1368066	As		A	M	Peptidase S53
1373549					GH125
1404940	As	W	A		CBM1-GH3
1446585			A		Peptidase S53
1438412			A	M	Peptidase S10
1438352				M	AA2;VP-atypical
1391002	As		A	M	Peptidase S10
1435938	As		A	M	Peptidase S10
1477425				M	AA1_1
68108			A	M	AA3_2
1434821	As		A	M	GH18
1445175	As	W	A	M	GH16
1445623	As	W	A		GH43-CBM35
1456924	As		A		GH18-CBM5-CBM5
1463186	As				GH18-CBM5
1471466	As		A	M	GH18
1434274		W			GH3
1357645	As	W	A	M	GH30
1435229	As	W	A	M	Peptidase M36
320973	As	W	A	M	GH76
1042478			A		Peptidase A1
1401294	As	W	A	M	GH55
1424871	As		A	M	GH18
1373420				M	Peptidase S10
1106774	As	W			GH18-CBM5
1358510	As				GH30_3
1421509	As		A	M	GH5_9
1451086			A	M	GH16
1480891	As	W		M	GH37
1426153	As		A	M	GH31
1432108	As	W	A	M	AA3_2
1437289	As	W	A	M	Peptidase S33
1441629			A	M	Peptidase S53
1359658	As	W	A	M	Peptidase A1
1434191					GH78
1446545					Peptidase S53
1369367	As	W	A	M	GH47
1425475	As	W	A	M	GH5_9
1447621	As		A		CBM18-GH16
316489					EXPN
1438246	As	W	A	M	CE8
688728	As	W	A		GH28
718112	As	W	A	M	GH32
1435997	As	W	A	M	Peptidase S10
1407107	As	W	A	M	Peptidase S53
1435894	As	W	A	M	GH79
1361613				M	GH16
1418820					GH28
408613		W			GH43
1368522		W			AA9
1423642	As	W	A		AA9
1476810		W			Peptidase S8 and S53
1430367				M	Peptidase S10
1422852			A	M	GH16
1424636	As	W	A	M	Peptidase M35
1373467	As	W	A	M	GH92
1418607					Peptidase A1
1428287		W	A	M	GH18
1430584	As		A	M	GH30_3
1431843	As	W	A	M	GH27
1433845	As	W	A	M	GH17
1434602					GH16
1442230		W	A	M	GH16
1447824	As				GH18-CBM5
1466845	As	W	A	M	PL8_4
1473510	As		A	M	GH16
1357326	As	W	A		CBM1-GH6
1359888	As	W	A		CBM1-GH5_7
1370654	As	W	A		GH28
1377179	As	W	A		GH55
1389216	As	W	A	M	GH7
1401955	As	W	A		AA8-AA3_1
1411666	As	W	A		GH3
1428145		W			AA9-CBM1
1433077	As	W			GH45
1437875			A		Peptidase S10
1439328		W	A		CBM1-CE16
1444114	As	W	A	M	Peptidase S53
1453868	As				GH92
1467772	As	W	A		GH131-CBM1
1464049	As				AA2; MnP-short
1067038		W			GH78
1463187	As				GH18-CBM5
1452465	As	W			AA1_1
1427559				M	CBM50-CBM50
1432218		W	A	M	Peptidase S28
1433108	As	W	A	M	Peptidase S10
1361311	As	W	A		GH72-CBM43
1372762	As	W	A	M	Peptidase G1
1442243	As	W	A	M	Peptidase S53
1451679	As	W	A	M	EXPN
1369807	As	W	A	M	GH2
1378812		W	A	M	GH88
1432550		W			GH5_15
1443101	As	W	A	M	GH95
1465734			A	M	AA3_2
1468129		W		M	GH31
1468712		W	A		GH79
1514584	As		A	M	GH92
1358049		W			GH12
1435501	As	W			GH43

1465736	As	W	A	M	Peptidase S10
1365564	As	W	A	M	GH74-CBM1
1374028	As	W	A		AA9
1424818	As	W	A	M	GH7
1366028	As	W	A		GH7
1369390		W			AA9
1373375	As		A	M	CE16
1375024	As	W	A		CBM1-GH5_5
1375723		W	A		GH12
1377173	As	W	A		CBM1-CE1
1392142	As	W	A		CBM1-CE1
1426850	As	W	A		CBM1-GH10
1429791	As	W	A		CBM1-GH5_5
1434718	As	W			CBM1-GH5_5
1437837	As	W	A		CBM1-GH10
1470260		W	A		CBM1-CE15
1471070	As	W	A	M	GH25
1472584		W			CBM1
1430912	As		A		Peptidase S10
793241		W			AA9
1382005	As	W	A	M	Peptidase A1
1431197	As	W	A	M	Peptidase A1
1357891	As		A	M	AA5_1
1361091	As		A	M	CE4
1366139	As	W	A	M	AA1_1
1367686	As	W	A	M	AA5_1
1431377		W			Peptidase A1
1433632		W			Peptidase A1
1433997	As	W	A	M	GH18
1468666		W			Peptidase S53
1417214		W	A		AA9
1439310	As	W	A		GH28
1466495		W			AA9
1497577	As	W		M	GH13_32-CBM20
1435885		W			CBM1-GH10

Table S18. List of proteins detected in the secretomes of *P. sanguineus* BRFM 1264 grown on Aspen (As), Pine (P), wheat straw (W), Avicel (A) or maltose (M).

Pycsa1 protID	secretome				expert_annot
1588377					GH125
1589244	As	W	A	M	GH27
1593166	As	W			CE4
1617673		W			Peptidase S41
1639180		W	A		AA1_1
1664834	As	W	A	M	GH2
1672749	As	W			GH18-CBM5
1705196	As	W			GH31
1721241		W	A		GH92
1560767	As	W		M	AA1_1
1575750	As	W	A	M	GH76
1577230		W			GH28
1594881		W	A	M	GH18-CBM5
1609029					GH92
1675320	As	W			GH25
1680674					GH30_3
1695354	As	W			GH43
1560745	As	W	A		GH79
1571620	As	W	A		GH51
1572980		W	A		Peptidase M35
1590027	As	W			GH35
1593425					GH5_15
1663870	As	W	A	M	GH92
1750423	As	W	A		GH28
1225964				M	GH16
1561243	As		A	M	Peptidase A1
1595487				M	Peptidase S53
1590768					GH28
1588747		W			AA9
1672495		W	A		AA9
1679991			A	M	AA2; VP-atypical
1562112		W	A		Peptidase S10
1571710				M	Peptidase A1
1579336	As	W	A	M	GH5_9
12062	As	W			Peptidase A1
1428239	As	W			GH88
1571187	As	W	A	M	GH18
1596128					Peptidase S10
1707539		W			Peptidase A1
174056					Peptidase A1
368711					GH28
1597347	As	W	A	M	GH30_3
1676925	As				PL8_4
1590841	As	W	A	M	GH55
1676158	As			M	GH18
9472					Peptidase S10
1589391	As				Peptidase S10
1610761	As		A	M	CBM18-GH16
1637500	As	W			Peptidase A1
1660200	As	W	A	M	GH5_9
1567160	As	W	A	M	GH95
1583177	As	W	A	M	GH16
1183761	As	W	A		Peptidase S10
1537322		W			GH3
1561085	As	W			GH43
1561925	As	W		M	GH79
1577052		W			GH3
1601452	As		A	M	GH18
1574363	As				AA3_2
1598175					GH78
1603355	As	W	A	M	GH32
1755702	As	W			GH78
1327655					GH3
1668810	As				Peptidase S10
924280				M	Peptidase S33
1575571	As	W			Peptidase M36
1581106		W	A	M	GH16
1608349	As	W	A	M	Peptidase S10
1677049	As	W	A	M	Peptidase S10
1612352		W			GH45
1675671	As	W	A	M	GH7
1680487					GH115
1721886	As	W	A		GH74
1727634	As	W	A	M	GH55
1738933	As	W	A		GH28
571793	As	W	A		GH131-CBM1
1586161	As	W	A		Peptidase S33
1609963				M	PL14_5
1668179				M	CBM50-CBM50
1671724	As	W	A	M	Peptidase S53
1674138				M	AA3_2
1127567	As	W			GH16
1610208				M	Peptidase S10
1631901	As	W	A		GH18-CBM5
1747035					GH3
1719016	As	W			GH30
1000963		W			Peptidase M35
1674899				M	Peptidase S10
790266	As	W			Peptidase S8 and S53
1581227	As		A	M	GH16
1590088	As	W	A	M	GH37
1614322	As	W	A	M	GH15-CBM20
1615309	As	W	A		GH16
1639615		W	A		GH16

1590513		W	A		Peptidase S28
1602327			A		Peptidase A1
1602673		W	A	M	Peptidase S53
1588252	As	W	A		GH16
1588246	As	W	A	M	GH13_32-CBM20
1593766	As	W	A	M	GH18
1598645	As	W	A		GH27
1609201	As	W	A		GH47
1645517		W			GH17
1615206		W	A		GH18
1672345	As	W	A		CBM1-GH5_7
1612129	As	W	A	M	Peptidase A1
1636119	As	W	A	M	Peptidase A1
1565342	As		A	M	GH72-CBM43
1601936					AA3_2
1611547		W			GH5_9
1615325		W	A	M	Peptidase S53
1672103	As	W	A	M	AA5_1
1677054	As	W	A	M	Peptidase S10
1749163		W			Peptidase S53
1571675		W			GH12
1589823	As	W	A	M	CBM1-GH10
1590621		W			CE16
1600918	As	W			GH18-CBM5
1679445		W			GH10
1755743		W	A	M	GH18-CBM5-CBM5
620905	As	W	A		GH43-CBM35
1562796	As	W	A	M	Peptidase S53
1583166	As	W	A	M	AA1_1
1602760		W			Peptidase S53
1674360	As	W			GH16
1567787	As	W	A		CBM1-GH6
1583489		W	A		AA9-CBM1
1672751	As	W	A		AA8-AA3_1
1560782		W			CBM1-GH10
1598628	As	W	A		CBM1-GH3
1600019		W	A		AA9
1610181	As	W	A		CBM1-GH10
1674106	As	W	A		CBM1-GH5_5
385598	As	W	A		AA9
1610435	As	W	A		GH7
1689209	As	W	A	M	AA5_1
272447	As	W	A	M	Peptidase A1
1562669					Peptidase A1
1565370	As	W	A	M	CE4
1592699		W			Peptidase A1
1595563	As	W	A	M	EXPN
1653985	As	W	A		GH18
1678350					Peptidase A1
1695590			A	M	AA3_2
1583090	As	W	A	M	GH7
1559723		W			GH31
1586920		W			GH20
1674581	As	W	A		CBM1-GH5_5
1677933		W	A		CBM1-CE1
1561648		W	A		GH53
1561762		W	A		CBM1-CE1
1566842	As	W	A		GH12
1577721	As	W	A		CE8
1597938	As	W	A		CBM1-GH5_5

Table S19. List of the groups of genes (nodes) from *P. cinnabarinus* BRFM 137, *P. coccineus* BRFM 310 and *P. sanguineus* BRFM 1264 up-regulated in response to Avicel, and their transcription profile on wheat straw and aspen. For each node the mean of log2 read counts and log2 fold change are indicated. Nodes for which the mean log2 read count was ≥ 12 on cellulose and nodes for which the mean log2 fold change as compared to maltose was ≥ 2 were considered respectively as highly transcribed or up-regulated on the substrates. Non significant changes in transcript level as deduced from DESeq2 are indicated by log2 fold change = 0. All genes were identified after expert annotation. M: maltose, AVI: avicel, WS: wheat straw, Asp: aspen.

protID	species	Expert annotation	detected in the secretome on Avicel	node ID	log2 read count				log2 fold change vs. maltose		
					M	AVI	WS	Asp	AVI	WS	Asp
1749457	*P. sanguineus*	CBM1		4	8,89	**13,90**	10,90	10,26	**5,94**	2,61	1,88
1404940	*P. coccineus*	GH3	yes	4	9,14	**12,88**	9,27	10,84	**4,36**	0,00	0,00
1373549	*P. coccineus*	GH125		4	10,31	**12,60**	10,03	10,90	**2,90**	0,00	0,00
6959	*P. cinnabarinus*	GH131		4	9,84	**13,28**	11,22	9,26	**3,74**	1,87	0,00
7188	*P. cinnabarinus*	GH131		4	9,60	**12,89**	10,05	9,02	**3,56**	1,09	0,00
1361992	*P. coccineus*	GH31		4	10,14	**12,52**	10,59	10,74	**3,23**	1,09	1,34
1596804	*P. sanguineus*	GH5_22		4	9,75	**12,14**	10,38	9,67	**2,79**	0,00	0,00
1438101	*P. coccineus*	HTP		4	9,70	**12,90**	9,99	10,31	**3,98**	0,00	0,00
1517	*P. cinnabarinus*	Peptidase A1		4	10,01	**11,84**	9,73	9,01	**2,16**	0,00	0,00
1368066	*P. coccineus*	Peptidase S53	yes	4	10,82	**13,18**	9,28	8,88	**3,03**	0,00	0,00
1446585	*P. coccineus*	Peptidase S53	yes	4	9,67	**13,78**	10,45	9,13	**5,27**	0,00	0,00
MEAN node 4					9,81	**12,90**	10,17	9,82	**3,72**	0,61	0,29
9327	*P. cinnabarinus*	AA8		8	8,07	**13,47**	8,01	7,53	**5,48**	0,00	0,00
1672495	*P. sanguineus*	AA9	yes	8	8,80	**14,91**	8,25	6,97	**6,90**	0,00	0,00
1571272	*P. sanguineus*	AA9		8	8,42	**14,98**	9,40	7,40	**7,33**	0,00	0,00
1588747	*P. sanguineus*	AA9		8	8,30	**14,25**	10,23	9,22	**6,35**	0,00	0,00
1572897	*P. sanguineus*	CBM1		8	7,15	**13,49**	9,49	9,30	**7,18**	2,97	2,76
MEAN node 8					8,15	**14,22**	9,08	8,08	**6,65**	0,59	0,55
1414868	*P. coccineus*	AA3_3		11	10,86	**12,73**	10,69	12,01	2,46	0,00	1,84
1445175	*P. coccineus*	GH16	yes	11	10,77	**10,69**	9,43	12,03	0,00	0,00	1,90
1434821	*P. coccineus*	GH18	yes	11	11,37	**10,81**	9,53	12,15	0,00	0,00	0,00
1471466	*P. coccineus*	GH18	yes	11	10,60	**12,96**	10,38	12,82	3,04	0,00	2,97
1463186	*P. coccineus*	GH18		11	11,39	**13,19**	9,89	12,35	2,53	0,00	1,63
1456924	*P. coccineus*	GH18	yes	11	10,02	**11,81**	9,32	12,39	2,49	0,00	3,19
1445623	*P. coccineus*	GH43	yes	11	11,31	**12,22**	9,74	11,95	0,00	0,00	0,00
1473624	*P. coccineus*	hydrophobin		11	11,82	**13,65**	8,06	12,57	2,97	-3,02	0,00
1438619	*P. coccineus*	Peptidase A1		11	12,16	**13,38**	10,03	12,12	1,77	0,00	0,00
1465489	*P. coccineus*	Peptidase C14		11	10,99	**12,03**	9,99	10,72	1,68	0,00	0,00
MEAN node 11					11,13	**12,35**	9,71	12,11	1,08	-0,30	1,15
1597900	*P. sanguineus*	AA8-CBM1		27	7,06	10,35	6,94	7,62	**3,74**	0,00	0,00
1672983	*P. sanguineus*	GH5_7		27	8,24	11,90	8,24	8,31	**4,32**	0,00	0,00
1436016	*P. coccineus*	Collagen related		27	8,45	10,44	8,01	8,15	**2,58**	0,00	0,00
1464770	*P. coccineus*	GH131		27	9,70	11,01	7,78	7,84	**2,11**	0,00	0,00
1580084	*P. sanguineus*	GH16		27	8,08	9,22	6,91	7,20	**0,00**	0,00	0,00
1186661	*P. coccineus*	GST FuA		27	9,53	11,39	7,77	7,01	**2,89**	0,00	-1,70
1602785	*P. sanguineus*	GT1		27	8,12	9,39	7,33	7,37	**0,00**	0,00	0,00
1427639	*P. coccineus*	Peptidase M43B		27	7,31	10,23	5,87	8,02	**3,39**	0,00	0,00

1361674	*P. coccineus*	Peptidase S10		27	7,97	10,91	6,71	7,99	**3,51**	0,00	0,00
1438989	*P. coccineus*	Peptidase S28		27	8,97	9,97	7,28	8,78	**1,80**	0,00	0,00
654188	*P. sanguineus*	Peptidase S28		27	8,56	10,84	7,54	6,74	**2,83**	0,00	0,00
1377932	*P. coccineus*	Peptidase S53		27	8,13	11,06	7,75	6,42	**4,11**	0,00	0,00
1379531	*P. coccineus*	Peptidase S53		27	9,00	9,84	7,76	7,25	**1,68**	0,00	0,00
1409152	*P. coccineus*	PL4_3		27	8,09	9,50	8,69	7,15	**2,18**	1,55	0,00
MEAN node 27					8,37	10,43	7,47	7,56	**2,51**	0,11	-0,12
1104	*P. cinnabarinus*	CBM1		29	11,65	**15,57**	**13,31**	**11,76**	**4,72**	**1,94**	0,00
7494	*P. cinnabarinus*	GH18		29	11,56	**16,20**	**12,64**	**11,23**	**5,49**	**1,44**	0,00
1578812	*P. sanguineus*	GH1		29	10,09	**14,28**	**12,76**	**11,86**	**5,38**	**3,19**	2,12
1680487	*P. sanguineus*	GH115		29	10,45	**13,84**	**14,01**	**13,17**	**4,15**	**3,89**	2,84
571793	*P. sanguineus*	GH131	yes	29	9,17	**15,53**	**12,95**	**12,13**	**7,92**	**4,24**	3,26
1581087	*P. sanguineus*	GH18		29	10,06	**13,74**	**14,17**	**12,27**	**2,92**	**0,00**	0,00
1738933	*P. sanguineus*	GH28	yes	29	9,75	**15,16**	**13,29**	**12,30**	**6,51**	**3,83**	2,67
372	*P. cinnabarinus*	GH28	yes	29	10,46	**13,79**	**13,73**	**11,88**	**3,67**	**3,28**	1,72
9953	*P. cinnabarinus*	GH3		29	10,08	**13,65**	**13,82**	**12,77**	**3,95**	**3,69**	2,76
1612352	*P. sanguineus*	GH45		29	11,70	**15,35**	**12,56**	**12,31**	**4,53**	**0,00**	0,00
1369367	*P. coccineus*	GH47	yes	29	11,00	**14,60**	**13,67**	**13,24**	**4,55**	**3,14**	2,86
1192330	*P. sanguineus*	GH5_22		29	11,49	**14,24**	**12,66**	**11,10**	**3,47**	**0,00**	0,00
1727634	*P. sanguineus*	GH55	yes	29	11,11	**13,13**	**13,01**	**11,28**	**2,67**	**2,22**	0,00
1675671	*P. sanguineus*	GH7	yes	29	10,38	**17,25**	**12,42**	**10,50**	**8,50**	**0,00**	0,00
1721886	*P. sanguineus*	GH74	yes	29	9,92	**15,08**	**13,24**	**12,48**	**6,80**	**3,85**	2,98
MEAN node 29					10,59	**14,76**	**13,22**	**12,02**	**5,02**	**2,31**	1,41
1368522	*P. coccineus*	AA9		34	8,02	11,01	10,16	9,33	**4,04**	**3,35**	**2,33**
1423642	*P. coccineus*	AA9	yes	34	7,76	11,99	9,69	9,57	**5,00**	**3,12**	**2,79**
1431096	*P. coccineus*	CBM1		34	7,39	11,05	8,66	10,17	**4,51**	**2,42**	**3,71**
1679979	*P. sanguineus*	CE15		34	5,83	10,85	10,87	9,43	**4,67**	**4,78**	**3,43**
1449232	*P. coccineus*	GH1		34	7,45	11,23	10,19	10,72	**4,48**	**3,65**	**4,23**
1614238	*P. sanguineus*	GH16		34	7,22	10,27	8,64	10,92	**3,39**	**0,00**	**3,78**
1439660	*P. coccineus*	GH20		34	7,53	10,00	8,13	9,41	**2,76**	**0,00**	**2,74**
650518	*P. coccineus*	GH20		34	7,54	10,42	8,69	9,22	**3,89**	**2,11**	**2,76**
1469372	*P. coccineus*	GH28		34	7,85	9,91	9,48	9,60	**2,71**	**2,53**	**2,78**
1719016	*P. sanguineus*	GH30		34	8,79	11,43	8,85	9,33	**3,81**	**0,00**	**0,00**
2943	*P. cinnabarinus*	GH43	yes	34	7,52	10,00	9,49	8,79	**2,73**	**2,43**	**1,85**
1150083	*P. sanguineus*	GH45		34	6,64	9,35	9,22	9,14	**3,22**	**3,16**	**3,07**
4664	*P. cinnabarinus*	GH5_22		34	6,63	9,40	9,49	9,56	**2,98**	**3,14**	**3,18**
1388575	*P. coccineus*	GH5_7		34	7,62	11,06	8,11	9,79	**4,18**	**0,00**	**3,18**
1377613	*P. coccineus*	GST FuA		34	7,81	10,31	9,54	9,81	**3,19**	**2,66**	**3,07**
1463286	*P. coccineus*	hydrophobin		34	7,65	10,39	9,83	11,19	**3,18**	**3,05**	**4,42**
1273531	*P. coccineus*	Peptidase		34	7,84	10,19	10,08	11,14	**3,02**	**3,13**	**4,32**
141	*P. cinnabarinus*	Peptidase M43B		34	7,49	11,06	7,76	10,39	**3,76**	**1,04**	**3,15**
1573969	*P. sanguineus*	Peptidase S28		34	7,11	9,08	8,79	9,40	**2,11**	**2,10**	**2,65**
MEAN node 34					7,46	10,47	9,25	9,84	**3,56**	**2,24**	**3,02**
1465271	*P. coccineus*	AA3_3		39	9,47	**13,25**	**11,86**	**13,41**	**4,46**	**2,97**	**4,79**
1401955	*P. coccineus*	AA8-AA3_1	yes	39	8,59	**14,94**	**13,48**	**13,38**	**7,06**	**4,80**	**4,99**
1382161	*P. coccineus*	AA9		39	8,66	**14,47**	**11,65**	**13,17**	**7,07**	**3,67**	**5,31**
1428145	*P. coccineus*	AA9		39	9,38	**14,53**	**13,00**	**13,76**	**6,48**	**4,15**	**5,16**
1439328	*P. coccineus*	CE16	yes	39	6,99	**13,72**	**13,80**	**12,55**	**6,81**	**6,74**	**5,73**
1359888	*P. coccineus*	GH5_7	yes	39	8,69	**14,05**	**12,45**	**13,23**	**5,88**	**4,16**	**4,98**
1672345	*P. sanguineus*	GH5_7	yes	39	8,85	**14,55**	**13,56**	**13,25**	**6,69**	**4,96**	**4,43**
1357326	*P. coccineus*	GH6	yes	39	8,11	**14,76**	**12,29**	**14,72**	**7,14**	**4,05**	**6,50**

1672909	*P. sanguineus*	EXPN		39	9,05	**13,15**	**13,64**	**12,92**	**0,00**	**0,00**	**0,00**
5717	*P. cinnabarinus*	GH1		39	7,77	**13,05**	**11,45**	**12,03**	**5,29**	**3,77**	**4,18**
1426831	*P. coccineus*	GH115		39	7,79	**13,66**	**11,76**	**13,12**	**6,73**	**4,57**	**6,12**
1467772	*P. coccineus*	GH131	yes	39	8,13	**14,48**	**11,90**	**12,83**	**6,88**	**3,89**	**4,77**
1615206	*P. sanguineus*	GH18	yes	39	9,00	**12,21**	**12,90**	**13,12**	**3,77**	**4,33**	**4,28**
1370654	*P. coccineus*	GH28	yes	39	8,48	**12,01**	**12,69**	**12,49**	**3,83**	**4,95**	**4,59**
1411666	*P. coccineus*	GH3	yes	39	8,45	**12,43**	**12,17**	**12,02**	**4,89**	**4,48**	**4,56**
1433077	*P. coccineus*	GH45		39	6,37	**12,84**	**12,02**	**12,77**	**7,46**	**6,39**	**7,32**
4663	*P. cinnabarinus*	GH5_22		39	8,03	**13,36**	**12,90**	**12,34**	**5,49**	**4,84**	**4,31**
1377179	*P. coccineus*	GH55	yes	39	9,33	**13,43**	**11,58**	**12,80**	**4,48**	**2,87**	**4,05**
1389216	*P. coccineus*	GH7	yes	39	8,08	**14,72**	**11,79**	**12,67**	**6,62**	**3,48**	**4,36**
1453868	*P. coccineus*	GH92		39	9,26	**13,52**	**13,55**	**13,73**	**5,11**	**4,91**	**5,41**
1437875	*P. coccineus*	Peptidase S10	yes	39	8,01	**11,82**	**12,83**	**12,36**	**4,63**	**5,61**	**5,44**
1444114	*P. coccineus*	Peptidase S53	yes	39	7,31	**12,84**	**15,00**	**13,61**	**6,09**	**8,08**	**7,19**
MEAN node 39					8,35	**13,54**	**12,65**	**13,01**	**5,58**	**4,44**	**4,93**
1465734	*P. coccineus*	AA3_2	yes	49	9,31	**11,49**	11,26	11,65	**2,80**	2,68	**3,26**
1672206	*P. sanguineus*	AA3_2		49	9,84	**11,55**	11,88	11,59	**0,00**	0,00	**0,00**
5578	*P. cinnabarinus*	AA9		49	10,45	**13,76**	11,38	11,01	**3,66**	1,45	**1,01**
1589823	*P. sanguineus*	GH10	yes	49	10,26	**13,86**	11,81	10,22	**4,21**	0,00	**0,00**
1590621	*P. sanguineus*	CE16		49	10,02	**12,39**	11,27	11,23	**2,66**	0,00	**0,00**
1679445	*P. sanguineus*	GH10		49	9,99	**11,73**	12,09	11,30	**2,18**	2,55	**1,61**
1571675	*P. sanguineus*	GH12		49	9,40	**13,00**	11,83	10,57	**4,22**	2,91	**0,00**
1600918	*P. sanguineus*	GH18		49	8,85	**11,94**	11,83	11,53	**3,75**	3,62	**3,14**
1755743	*P. sanguineus*	GH18	yes	49	9,95	**12,14**	12,57	12,30	**2,51**	2,82	**2,36**
1369807	*P. coccineus*	GH2	yes	49	9,34	**12,35**	11,14	11,32	**3,78**	2,54	**2,83**
5171	*P. cinnabarinus*	GH2		49	9,49	**11,93**	12,30	11,45	**2,86**	2,98	**2,22**
1435009	*P. coccineus*	GH3		49	9,97	**13,21**	11,09	11,09	**4,01**	1,68	**1,77**
1468141	*P. coccineus*	GH3		49	9,80	**13,01**	12,81	12,18	**3,82**	3,39	**3,08**
8515	*P. cinnabarinus*	GH3		49	9,52	**12,54**	11,32	11,26	**3,39**	2,21	**2,04**
1468129	*P. coccineus*	GH31		49	10,53	**12,74**	11,74	12,29	**2,84**	1,74	**2,51**
1027	*P. cinnabarinus*	GH31	yes	49	10,53	**12,43**	11,18	11,72	**2,34**	1,19	**1,53**
620905	*P. sanguineus*	GH43	yes	49	9,76	**12,73**	12,04	10,69	**3,65**	2,74	**0,00**
3359	*P. cinnabarinus*	GH45		49	9,29	**12,52**	11,40	10,38	**3,58**	2,47	**1,55**
1432550	*P. coccineus*	GH5_15		49	9,30	**14,49**	10,26	12,54	**6,38**	1,61	**4,10**
1578240	*P. sanguineus*	GH5_7		49	8,66	**11,91**	11,47	11,24	**3,77**	3,40	**3,00**
1468712	*P. coccineus*	GH79	yes	49	10,25	**12,55**	11,87	12,68	**2,70**	2,23	**3,11**
1378812	*P. coccineus*	GH88	yes	49	9,28	**12,80**	11,93	12,43	**4,29**	3,31	**4,05**
1514584	*P. coccineus*	GH92	yes	49	10,42	**12,82**	10,90	11,59	**3,03**	0,00	**1,91**
1443101	*P. coccineus*	GH95	yes	49	10,87	**13,47**	11,84	11,80	**3,23**	0,00	**1,61**
1464220	*P. coccineus*	Peptidase aspartic		49	10,10	**12,83**	11,39	12,04	**3,53**	1,94	**2,75**
1359313	*P. coccineus*	Peptidase M		49	9,48	**13,49**	10,35	11,78	**4,74**	0,00	**3,08**
1430478	*P. coccineus*	Peptidase M		49	8,98	**12,11**	10,61	11,44	**3,98**	2,37	**3,35**
1582079	*P. sanguineus*	Peptidase M		49	10,25	**14,27**	11,57	10,93	**5,37**	1,85	**0,00**
5276	*P. cinnabarinus*	Peptidase M		49	10,61	**14,39**	11,71	11,05	**4,32**	1,53	**0,90**
1444442	*P. coccineus*	PL8		49	9,67	**13,25**	11,57	12,70	**4,36**	2,48	**3,85**
MEAN node 49					9,81	**12,79**	11,55	11,53	**3,53**	1,92	**2,02**
1429888	*P. coccineus*	AA3_2		50	6,81	11,05	11,96	12,49	**5,19**	**5,91**	**6,67**
1435885	*P. coccineus*	GH10		50	7,54	8,94	10,70	12,53	**3,28**	**3,58**	**5,46**
1438246	*P. coccineus*	CE8	yes	50	7,94	10,64	12,22	11,45	**3,56**	**5,13**	**4,53**
7738	*P. cinnabarinus*	GH115		50	8,19	9,41	12,35	11,42	**1,85**	**4,14**	**3,31**
1358049	*P. coccineus*	GH12		50	6,23	9,40	10,77	10,80	**3,51**	**5,35**	**5,38**
688728	*P. coccineus*	GH28	yes	50	7,79	8,39	12,83	11,59	**0,00**	**5,76**	**4,53**
161	*P. cinnabarinus*	GH30		50	5,62	9,51	11,32	12,14	**3,84**	**5,24**	**5,79**
718112	*P. coccineus*	GH32	yes	50	6,73	10,27	11,52	11,32	**4,25**	**5,56**	**5,46**
1435501	*P. coccineus*	GH43		50	6,68	9,69	11,66	12,21	**0,00**	**4,90**	**5,02**
1363671	*P. coccineus*	GH53		50	6,23	9,45	10,46	12,42	**3,84**	**5,11**	**7,05**
6857	*P. cinnabarinus*	GH92	yes	50	8,34	10,49	11,58	12,63	**2,51**	**3,24**	**3,95**
1419800	*P. coccineus*	hydrophobin		50	7,28	9,55	11,25	13,91	**0,00**	**4,29**	**7,02**
1643269	*P. sanguineus*	Peptidase S53		50	7,34	9,81	11,66	11,24	**3,39**	**4,40**	**3,75**

1360083	*P. coccineus*	Peptidase S8 and S53		50	7,25	9,42	11,58	11,36	**2,55**	**5,13**	**5,00**
MEAN node 50					7,14	9,72	11,56	11,96	**2,70**	**4,84**	**5,21**
1672751	*P. sanguineus*	AA8-AA3_1	yes	57	9,20	**15,45**	**14,68**	**12,66**	**7,55**	**5,51**	**3,59**
9131	*P. cinnabarinus*	AA8-AA3_1		57	11,72	**16,25**	**16,13**	**12,90**	**5,36**	**3,89**	**1,41**
1374028	*P. coccineus*	AA9	yes	57	9,57	**16,15**	**15,32**	**14,18**	**6,91**	**4,80**	**4,00**
1583489	*P. sanguineus*	AA9	yes	57	9,56	**16,22**	**13,85**	**12,54**	**8,16**	**4,39**	**3,03**
6608	*P. cinnabarinus*	GH5_7	yes	57	10,82	**15,87**	**15,78**	**12,89**	**5,65**	**4,40**	**2,18**
1567787	*P. sanguineus*	GH6	yes	57	8,39	**16,39**	**14,54**	**13,41**	**9,93**	**6,42**	**5,26**
1370248	*P. coccineus*	GH1		57	10,34	**15,79**	**14,26**	**12,91**	**7,14**	**4,17**	**3,27**
1424818	*P. coccineus*	GH7	yes	57	9,46	**16,29**	**15,12**	**15,89**	**7,29**	**4,64**	**5,67**
1365564	*P. coccineus*	GH74	yes	57	9,23	**14,43**	**14,27**	**14,14**	**6,35**	**5,48**	**5,71**
MEAN node 57					9,81	**15,87**	**14,88**	**13,50**	**7,15**	**4,85**	**3,79**
1600019	*P. sanguineus*	AA9	yes	58	6,22	**15,78**	**14,04**	13,03	**10,88**	**7,87**	**6,75**
385598	*P. sanguineus*	AA9	yes	58	5,77	**15,22**	**13,48**	12,49	**10,57**	**7,83**	**6,64**
5579	*P. cinnabarinus*	AA9		58	7,63	**14,86**	**12,40**	9,73	**7,45**	**4,75**	**2,54**
1560782	*P. sanguineus*	GH10		58	6,41	**14,05**	**13,05**	11,58	**7,31**	**6,14**	**4,63**
1610181	*P. sanguineus*	GH10	yes	58	8,18	**14,80**	**12,48**	10,46	**7,50**	**4,31**	**0,00**
1598628	*P. sanguineus*	GH3	yes	58	8,63	**15,00**	**12,23**	11,03	**7,78**	**4,11**	**2,86**
1674106	*P. sanguineus*	GH5_5	yes	58	7,11	**14,53**	**12,60**	11,69	**8,68**	**5,93**	**4,91**
1929	*P. cinnabarinus*	GH5_5	yes	58	6,78	**15,49**	**13,54**	10,24	**8,53**	**6,19**	**3,54**
8616	*P. cinnabarinus*	GH5_5	yes	58	8,21	**16,56**	**12,82**	10,30	**8,90**	**4,55**	**2,45**
4378	*P. cinnabarinus*	GH1		58	8,00	**15,90**	**13,63**	11,53	**8,56**	**5,50**	**3,66**
1365	*P. cinnabarinus*	GH131	yes	58	6,66	**16,14**	**13,96**	10,20	**9,43**	**6,59**	**3,59**
5845	*P. cinnabarinus*	GH7	yes	58	8,52	**17,02**	**12,80**	8,66	**8,92**	**4,18**	**0,00**
6605	*P. cinnabarinus*	Peptidase S10	yes	58	7,42	**13,73**	**15,42**	10,98	**6,19**	**7,07**	**3,61**
MEAN node 58					7,35	**15,31**	**13,27**	10,92	**8,52**	**5,77**	**3,48**
1369390	*P. coccineus*	AA9		59	6,74	**11,38**	9,06	11,00	**5,29**	**3,10**	**5,15**
1472584	*P. coccineus*	CBM1		59	7,52	**13,46**	8,91	11,75	**6,87**	**2,24**	**5,20**
1377173	*P. coccineus*	CE1	yes	59	6,01	**11,28**	9,07	11,19	**5,64**	**3,21**	**5,32**
1392142	*P. coccineus*	CE1	yes	59	8,25	**11,65**	8,39	12,04	**4,71**	**0,00**	**4,87**
1470260	*P. coccineus*	CE15	yes	59	7,42	**12,02**	9,54	11,08	**6,05**	**0,00**	**4,21**
1426850	*P. coccineus*	GH10	yes	59	8,08	**11,23**	8,74	13,32	**4,10**	**1,55**	**6,47**
1437837	*P. coccineus*	GH10	yes	59	6,91	**12,92**	9,46	11,10	**6,02**	**3,31**	**4,55**
1375024	*P. coccineus*	GH5_5	yes	59	6,71	**13,14**	10,99	11,82	**6,82**	**4,69**	**5,52**
1429791	*P. coccineus*	GH5_5	yes	59	6,20	**12,75**	10,28	12,17	**6,78**	**4,35**	**6,25**
1434718	*P. coccineus*	GH5_5		59	7,24	**12,89**	8,89	12,25	**6,87**	**2,50**	**5,90**
1470261	*P. coccineus*	CE15		59	5,28	**11,61**	10,33	12,18	**6,54**	**5,43**	**7,43**
1373375	*P. coccineus*	CE16	yes	59	8,73	**13,06**	9,42	11,45	**5,06**	**0,00**	**3,60**
1429512	*P. coccineus*	CE16		59	8,22	**11,60**	9,98	12,94	**4,02**	**2,57**	**5,62**
1437703	*P. coccineus*	CE16		59	6,67	**13,05**	9,71	12,86	**6,97**	**3,72**	**6,96**
1468081	*P. coccineus*	cerato-platanin		59	8,24	**12,56**	8,85	10,89	**4,63**	**0,00**	**3,39**
1375723	*P. coccineus*	GH12		59	7,31	**13,46**	8,11	11,44	**6,36**	**0,00**	**4,23**
1643	*P. cinnabarinus*	GH18	yes	59	8,68	**11,32**	9,66	13,21	**2,96**	**1,55**	**4,29**
1471070	*P. coccineus*	GH25	yes	59	8,98	**11,87**	10,46	13,17	**3,78**	**2,31**	**5,17**
1366028	*P. coccineus*	GH7	yes	59	7,49	**13,62**	10,39	12,17	**7,53**	**3,63**	**5,55**
1390886	*P. coccineus*	GH92		59	8,41	**11,67**	9,49	10,82	**3,79**	**1,94**	**3,28**
MEAN node 59					7,45	**12,33**	9,49	11,94	**5,54**	**2,31**	**5,15**
1430659	*P. coccineus*	AA9		60	6,06	13,13	7,11	10,97	**6,80**	**0,00**	**5,01**
793241	*P. coccineus*	AA9		60	6,72	13,30	9,07	9,14	**6,56**	**0,00**	**0,00**
107546	*P. sanguineus*	AA9		60	5,90	10,57	8,74	9,53	**5,46**	**3,43**	**4,11**
1740610	*P. sanguineus*	AA9		60	6,26	12,00	7,37	8,41	**6,37**	**1,76**	**2,87**
1376091	*P. coccineus*	GH131		60	4,80	10,80	7,93	8,04	**6,27**	**3,55**	**3,73**
1610435	*P. sanguineus*	GH7	yes	60	4,61	11,37	9,44	7,49	**6,88**	**5,13**	**3,30**
1430912	*P. coccineus*	Peptidase S10	yes	60	5,42	11,44	6,50	9,46	**6,52**	**0,00**	**4,54**
MEAN node 60					5,68	11,80	8,02	9,01	**6,41**	**1,98**	**3,37**
1439081	*P. coccineus*	CBM13		61	4,84	6,66	6,86	8,58	**2,28**	**2,75**	**4,59**
7437	*P. cinnabarinus*	CE1		61	2,99	6,16	5,75	9,14	**2,71**	**2,38**	**5,33**
1367487	*P. coccineus*	CE16		61	4,18	7,49	7,42	9,79	**3,16**	**3,66**	**6,07**
1378034	*P. coccineus*	GH45		61	4,10	7,84	6,52	7,72	**3,98**	**2,96**	**4,26**

1462467	*P. coccineus*	GH51		61	4,13	5,97	8,76	8,55	**0,00**	**5,00**	**4,92**
1393742	*P. coccineus*	GST GHR		61	4,65	6,02	8,07	7,24	**2,03**	**4,14**	**3,49**
1467651	*P. coccineus*	GST omega		61	4,91	6,94	7,37	6,75	**2,56**	**3,42**	**2,79**
1677288	*P. sanguineus*	Peptidase A1		61	5,70	6,52	7,56	8,32	**0,00**	**2,28**	**3,12**
465	*P. cinnabarinus*	Peptidase M28		61	3,91	4,71	6,17	8,42	**0,00**	**2,19**	**4,25**
MEAN node 61					4,38	6,48	7,16	8,28	**2,79**	**3,20**	**4,31**
8672	*P. cinnabarinus*	AA1_1	yes	66	9,65	11,25	**14,35**	**17,72**	**2,05**	**4,33**	**6,94**
1368318	*P. coccineus*	AA3_3		66	8,16	12,59	**15,15**	**15,58**	**5,09**	**7,27**	**8,30**
1596703	*P. sanguineus*	AA3_3		66	10,41	11,38	**17,10**	**16,72**	**0,00**	**6,35**	**5,64**
10401	*P. cinnabarinus*	EXPN		66	10,21	11,21	**17,13**	**14,76**	**1,47**	**5,89**	**4,22**
8001	*P. cinnabarinus*	GH28	yes	66	9,97	12,90	**15,52**	**15,33**	**3,28**	**4,96**	**4,80**
7004	*P. cinnabarinus*	GH35	yes	66	10,90	10,61	**15,42**	**14,93**	**0,00**	**4,08**	**3,72**
1423919	*P. coccineus*	Peptidase S53		66	10,16	12,06	**15,49**	**14,04**	**2,30**	**5,45**	**4,67**
1438837	*P. coccineus*	Peptidase S53		66	7,75	11,81	**16,26**	**15,92**	**4,09**	**8,23**	**8,23**
MEAN node 66					9,65	11,73	**15,80**	**15,63**	**2,29**	**5,82**	**5,81**
2028	*P. cinnabarinus*	AA9		67	6,12	**17,09**	**16,42**	**12,07**	**10,00**	**7,87**	**4,85**
9352	*P. cinnabarinus*	GH6	yes	67	7,84	**17,51**	**17,00**	**13,99**	**9,67**	**7,41**	**5,28**
1583090	*P. sanguineus*	GH7	yes	67	8,29	**17,34**	**16,45**	**15,12**	**11,56**	**8,20**	**6,91**
6809	*P. cinnabarinus*	GH7	yes	67	5,38	**18,14**	**15,65**	**11,80**	**10,33**	**7,21**	**4,63**
8640	*P. cinnabarinus*	GH7	yes	67	5,42	**19,14**	**18,14**	**14,42**	**10,02**	**9,26**	**6,03**
7129	*P. cinnabarinus*	GH74	yes	67	6,32	**16,85**	**16,75**	**13,83**	**10,99**	**8,98**	**6,78**
MEAN node 67					6,56	**17,68**	**16,73**	**13,54**	**10,43**	**8,15**	**5,75**
3646	*P. cinnabarinus*	AA9		68	2,99	**15,20**	**13,01**	**10,23**	**9,60**	**7,42**	**5,33**
5150	*P. cinnabarinus*	AA9		68	2,69	**15,74**	**14,67**	**10,74**	**10,44**	**8,69**	**5,85**
4319	*P. cinnabarinus*	CE15		68	1,24	**11,69**	**12,80**	**9,76**	**7,71**	**8,47**	**6,21**
4524	*P. cinnabarinus*	GH10	yes	68	4,01	**16,48**	**14,99**	**11,32**	**10,28**	**8,02**	**5,41**
5713	*P. cinnabarinus*	GH5_5	yes	68	2,39	**14,90**	**12,54**	**8,99**	**10,08**	**7,71**	**5,09**
5863	*P. cinnabarinus*	CE16		68	0,21	**15,25**	**14,09**	**9,86**	**12,20**	**10,57**	**7,19**
4523	*P. cinnabarinus*	GH10	yes	68	2,69	**16,15**	**14,12**	**10,65**	**11,35**	**8,79**	**6,10**
6994	*P. cinnabarinus*	GH12	yes	68	2,95	**15,15**	**11,99**	**8,72**	**9,72**	**6,77**	**4,42**
MEAN node 68					2,40	**15,07**	**13,53**	**10,03**	**10,17**	**8,31**	**5,70**
1417214	*P. coccineus*	AA9	yes	69	6,01	**13,49**	11,70	11,85	**7,52**	**5,59**	**5,70**
4047	*P. cinnabarinus*	AA9	yes	69	5,54	**14,79**	12,15	8,10	**8,82**	**6,02**	**2,86**
10062	*P. cinnabarinus*	AA9		69	4,86	**13,40**	11,20	8,94	**7,88**	**5,85**	**3,95**
1606668	*P. sanguineus*	AA9		69	5,11	**13,94**	11,64	10,92	**9,37**	**6,58**	**5,80**
9908	*P. cinnabarinus*	AA9		69	4,57	**11,12**	12,70	10,01	**5,86**	**7,15**	**4,91**
1561762	*P. sanguineus*	CE1	yes	69	5,87	**13,62**	10,51	8,96	**8,23**	**4,90**	**3,49**
1677933	*P. sanguineus*	CE1	yes	69	7,19	**14,19**	11,81	9,77	**7,57**	**4,72**	**2,79**
6822	*P. cinnabarinus*	CE1		69	5,77	**14,52**	11,88	8,34	**8,47**	**5,72**	**2,92**
7436	*P. cinnabarinus*	CE1	yes	69	5,70	**13,57**	13,52	10,07	**6,95**	**6,56**	**3,96**
1569202	*P. sanguineus*	CE16		69	4,63	**14,44**	10,44	9,10	**10,25**	**5,85**	**4,52**
4521	*P. cinnabarinus*	GH10	yes	69	6,88	**13,92**	11,04	8,82	**6,80**	**4,17**	**2,37**
1597938	*P. sanguineus*	GH5_5	yes	69	5,45	**14,84**	11,88	10,36	**10,39**	**6,77**	**5,05**
1674581	*P. sanguineus*	GH5_5	yes	69	6,47	**14,11**	12,12	10,34	**8,57**	**6,07**	**4,27**
1577721	*P. sanguineus*	CE8	yes	69	7,45	**12,14**	11,11	9,27	**4,85**	**3,89**	**2,16**
1566842	*P. sanguineus*	GH12	yes	69	5,71	**14,30**	10,44	10,63	**9,07**	**4,71**	**4,71**
1756265	*P. sanguineus*	GH131		69	6,40	**13,09**	10,24	9,75	**7,01**	**3,93**	**3,44**
1439310	*P. coccineus*	GH28	yes	69	7,07	**12,59**	11,31	10,70	**6,04**	**4,95**	**4,36**
7225	*P. cinnabarinus*	GH3	yes	69	6,46	**15,00**	11,33	8,06	**8,50**	**4,82**	**2,18**
8853	*P. cinnabarinus*	GH5_7	yes	69	5,56	**13,18**	12,31	10,53	**7,15**	**6,21**	**4,68**
1561648	*P. sanguineus*	GH53	yes	69	4,88	**14,61**	12,14	12,06	**10,61**	**7,38**	**7,11**
MEAN node 69					5,88	**13,74**	11,57	9,83	**8,00**	**5,59**	**4,06**
1466495	*P. coccineus*	AA9		70	3,96	10,06	12,15	8,55	**6,20**	**8,19**	**4,66**
1588880	*P. sanguineus*	AA9		70	-0,08	8,75	9,27	8,09	**6,60**	**6,88**	**5,88**
1634656	*P. sanguineus*	CE15		70	4,65	8,76	10,08	7,63	**4,06**	**5,71**	**3,40**
5946	*P. cinnabarinus*	CE16		70	3,98	8,47	9,90	11,83	**4,05**	**5,23**	**6,73**
9246	*P. cinnabarinus*	CE8		70	4,47	6,75	10,84	10,52	**2,16**	**5,68**	**5,35**
3282	*P. cinnabarinus*	GH12		70	4,10	10,05	11,15	8,93	**5,20**	**6,18**	**4,40**
7921	*P. cinnabarinus*	GH79		70	4,84	7,53	9,07	8,69	**2,56**	**4,11**	**3,82**
7922	*P. cinnabarinus*	GH79		70	4,29	7,86	9,12	9,02	**3,17**	**4,51**	**4,43**

1468559	*P. coccineus*	Peptidase M28		70	4,72	7,27	10,59	10,23	**0,00**	**5,99**	**5,79**
1466373	*P. coccineus*	Peptidase S53		70	5,45	7,20	10,84	9,19	**2,25**	**6,35**	**4,73**
MEAN node 70					4,04	8,27	10,30	9,27	**3,62**	**5,88**	**4,92**

Table S20. List of the groups of genes (nodes) from *P. cinnabarinus BRFM 137* , *P. coccineus* BRFM 310 and *P. sanguineus* BRFM 1264 up-regulated in response to wheat straw or aspen, not Avicel. For each node the mean of log2 read counts and log2 fold change are indicated. Nodes for which the mean log2 read count was ≥ 12 and nodes for which the mean log2 fold change as compared to maltose was ≥ 2 were considered respectively as highly transcribed or up-regulated on the substrates (bold). Non significant changes in transcript level as deduced from DESeq2 are indicated by log2 fold change = 0. All genes were identified after expert annotation.

protID	species	Expert annotation	detected in the secretome on wheat straw or aspen	nodeID	log2 read count maltose	log2 read count Avicel	log2 read count wheat straw	log2 read count aspen	log2 fold change vs. maltose Avicel	log2 fold change vs. maltose wheat straw	log2 fold change vs. maltose aspen
4518	*P. cinnabarinus*	AA3_2		13	10,00	9,06	11,32	10,98	0,00	1,78	**1,38**
8361	*P. cinnabarinus*	AA3_2		13	10,14	9,64	10,74	12,46	0,00	1,16	**2,46**
1195462	*P. sanguineus*	AA9		13	8,98	8,84	10,66	11,29	0,00	0,00	**0,00**
3435	*P. cinnabarinus*	GH10	yes	13	8,60	8,78	10,58	11,08	0,00	2,40	**2,62**
1592100	*P. sanguineus*	CE16		13	8,56	10,19	10,82	12,20	2,18	2,55	**3,70**
2129	*P. cinnabarinus*	CE16	yes	13	10,09	9,47	10,16	11,61	0,00	0,00	**1,84**
4944	*P. cinnabarinus*	GH16		13	10,04	9,68	10,64	11,12	0,00	1,19	**1,49**
1571187	*P. sanguineus*	GH18	yes	13	9,78	9,85	11,43	12,24	0,00	2,10	**2,68**
1644	*P. cinnabarinus*	GH18	yes	13	9,81	9,80	10,16	11,03	0,00	0,99	**1,61**
7817	*P. cinnabarinus*	GH18	yes	13	9,73	10,13	10,36	12,89	0,00	1,23	**3,18**
8611	*P. cinnabarinus*	GH20	yes	13	9,70	9,32	11,65	11,25	0,00	2,32	**1,89**
1377553	*P. coccineus*	GH28		13	8,03	9,68	10,25	11,40	2,21	3,03	**4,28**
1446047	*P. coccineus*	GH28		13	8,53	9,69	11,82	11,53	1,84	4,08	**3,98**
368711	*P. sanguineus*	GH28		13	8,91	8,89	10,68	10,97	0,00	2,29	**2,36**
1357645	*P. coccineus*	GH30	yes	13	9,26	9,90	11,31	12,14	0,00	2,72	**3,57**
1433	*P. cinnabarinus*	GH30_3	yes	13	10,29	8,85	9,39	11,66	0,00	0,00	**1,70**
320973	*P. coccineus*	GH76	yes	13	8,78	11,04	10,84	12,34	2,72	2,84	**4,45**
1428239	*P. sanguineus*	GH88	yes	13	10,30	9,70	11,32	12,07	0,00	1,57	**2,13**
360866	*P. sanguineus*	GT1		13	9,10	7,86	10,26	12,01	0,00	1,61	**3,10**
1463290	*P. coccineus*	hydrophobin		13	9,18	9,91	10,12	11,60	1,70	1,67	**3,29**
12062	*P. sanguineus*	Peptidase A1	yes	13	9,45	8,93	10,40	10,79	0,00	1,61	**1,83**
1438416	*P. coccineus*	Peptidase A1		13	9,97	9,82	10,93	10,99	0,00	1,73	**1,74**
1707539	*P. sanguineus*	Peptidase A1	yes	13	10,15	8,80	10,66	10,73	0,00	0,00	**0,00**
174056	*P. sanguineus*	Peptidase A1		13	9,16	9,47	10,47	12,83	0,00	1,85	**3,90**
1677650	*P. sanguineus*	Peptidase C14		13	9,85	9,49	10,51	11,61	0,00	0,00	**2,08**
1656	*P. cinnabarinus*	Peptidase M20		13	9,64	9,03	10,48	11,26	0,00	1,42	**1,98**
1678731	*P. sanguineus*	Peptidase M20		13	9,71	9,01	10,42	11,84	0,00	1,35	**2,52**
6355	*P. cinnabarinus*	Peptidase M20		13	10,33	9,91	11,44	11,79	0,00	1,57	**1,78**
1435229	*P. coccineus*	Peptidase M36	yes	13	8,95	8,76	11,22	10,88	0,00	3,05	**2,71**
1596128	*P. sanguineus*	Peptidase S10		13	8,40	7,97	10,26	11,50	0,00	2,33	**3,31**
7835	*P. cinnabarinus*	Peptidase S53	yes	13	9,88	9,71	10,95	10,79	0,00	1,63	**1,37**
1674248	*P. sanguineus*	Peptidase S8 and S53		13	9,26	9,84	10,65	11,84	1,19	2,04	**3,02**
6103	*P. cinnabarinus*	PL14_4		13	9,25	9,84	10,64	11,54	1,12	1,89	**2,53**
MEAN node 13					9,45	9,42	10,71	11,58	0,39	1,70	**2,44**
9339	*P. cinnabarinus*	AA5_1		22	9,65	11,07	11,11	9,59	1,86	**1,92**	0,00
1601452	*P. sanguineus*	GH18	yes	22	8,59	11,15	11,25	9,78	3,02	**2,99**	0,00
1438782	*P. coccineus*	GH2		22	8,96	10,52	10,33	9,97	2,31	**2,11**	1,92
374996	*P. sanguineus*	GH20		22	10,26	11,26	10,83	9,73	0,00	**0,00**	0,00
1537322	*P. sanguineus*	GH3	yes	22	9,06	10,08	12,14	9,97	0,00	**3,40**	0,00
1577052	*P. sanguineus*	GH3	yes	22	8,37	10,82	10,99	10,20	3,02	**3,00**	2,16
3431	*P. cinnabarinus*	GH3		22	10,15	11,00	11,56	9,46	1,37	**1,82**	0,00
1561085	*P. sanguineus*	GH43	yes	22	8,01	10,81	11,52	10,43	3,20	**3,97**	2,79

1573901	*P. sanguineus*	GH47		22	9,60	11,12	10,88	10,88	2,10	**1,94**	1,74
3220	*P. cinnabarinus*	GH5_15	yes	22	10,06	10,31	11,59	10,99	0,00	**0,00**	0,00
1600589	*P. sanguineus*	GH5_9		22	10,26	9,85	11,21	9,60	0,00	**1,48**	0,00
2095	*P. cinnabarinus*	GH51	yes	22	9,55	10,44	11,17	10,08	1,40	**2,09**	1,12
1561925	*P. sanguineus*	GH79	yes	22	9,05	10,82	11,23	10,62	2,21	**2,75**	1,98
5390	*P. cinnabarinus*	GST GTT2.2		22	10,37	11,61	10,94	10,22	1,71	**1,15**	0,00
1458648	*P. coccineus*	GT8		22	10,35	11,04	11,38	10,46	0,00	**1,59**	0,00
927394	*P. sanguineus*	Peptidase aspartic		22	9,17	9,73	10,85	10,50	0,00	**2,32**	1,82
1448471	*P. coccineus*	Peptidase C2		22	9,69	10,00	13,80	9,54	0,00	**4,79**	0,00
1183761	*P. sanguineus*	Peptidase S10	yes	22	8,96	11,25	10,62	10,24	2,66	**2,10**	0,00
1436117	*P. coccineus*	Peptidase S10		22	9,97	10,34	11,45	11,02	0,97	**2,12**	1,78
4254	*P. cinnabarinus*	Peptidase S53	yes	22	9,38	11,08	11,03	10,31	2,12	**2,07**	1,40
1438882	*P. coccineus*	Peptidase S58		22	10,15	10,29	11,38	10,36	0,00	**1,82**	0,00
MEAN node 22					9,51	10,69	11,30	10,19	1,33	**2,16**	0,80
1434275	*P. coccineus*	AA3_2		23	8,14	8,49	9,73	8,88	0,00	**2,51**	1,80
1574363	*P. sanguineus*	AA3_2	yes	23	8,44	8,41	10,09	9,04	0,00	**2,19**	0,00
1470943	*P. coccineus*	AA9		23	8,52	5,82	10,51	9,23	-2,04	**2,78**	1,59
5589	*P. cinnabarinus*	CE16		23	8,24	7,55	9,50	10,59	0,00	**1,83**	2,68
7727	*P. cinnabarinus*	GH115		23	9,28	8,38	10,85	9,81	0,00	**2,06**	1,14
6995	*P. cinnabarinus*	GH12		23	8,70	8,91	10,20	10,13	0,77	**2,04**	1,94
1575410	*P. sanguineus*	GH2		23	8,62	7,61	9,82	10,02	0,00	**0,00**	0,00
4540	*P. cinnabarinus*	GH20		23	7,99	8,06	10,01	9,15	0,00	**2,46**	1,75
3491	*P. cinnabarinus*	GH3		23	9,30	8,43	9,86	9,49	0,00	**1,18**	0,88
1603355	*P. sanguineus*	GH32	yes	23	8,93	8,31	10,44	10,52	0,00	**2,10**	1,99
1588232	*P. sanguineus*	GH51		23	8,17	8,88	10,63	9,12	0,00	**3,09**	1,56
1463537	*P. coccineus*	GH71		23	8,90	8,62	10,23	10,06	0,00	**2,10**	2,06
1434191	*P. coccineus*	GH78		23	7,86	8,89	10,48	10,53	0,00	**3,51**	3,58
1598175	*P. sanguineus*	GH78		23	8,08	7,66	9,99	9,29	0,00	**2,43**	0,00
1755702	*P. sanguineus*	GH78	yes	23	8,64	8,58	11,17	10,14	0,00	**3,11**	1,99
938	*P. cinnabarinus*	GH79		23	10,09	7,45	10,32	10,24	-1,80	**0,88**	0,00
1477603	*P. coccineus*	GST omega		23	9,69	5,84	9,45	9,37	-3,12	**0,00**	0,00
1599385	*P. sanguineus*	GT1		23	8,09	8,68	10,54	8,88	0,00	**2,97**	0,00
7502	*P. cinnabarinus*	Peptidase C1B		23	9,12	8,22	10,31	9,61	0,00	**1,73**	1,13
1613766	*P. sanguineus*	Peptidase S53		23	7,16	9,49	11,22	9,39	2,74	**4,63**	2,77
1674165	*P. sanguineus*	Peptidase S53		23	9,09	8,31	9,82	9,67	0,00	**1,41**	1,17
MEAN node 23					8,62	8,12	10,24	9,67	-0,16	**2,14**	1,33
1639180	*P. sanguineus*	AA1_1	yes	31	10,57	11,09	**12,77**	**12,03**	0,00	2,41	0,00
1463000	*P. coccineus*	AA3_2		31	11,08	11,96	**12,45**	**12,73**	1,40	1,91	2,34
3421	*P. cinnabarinus*	AA3_2		31	11,43	10,60	**12,63**	**11,94**	0,00	0,00	0,00
1464041	*P. coccineus*	AA9		31	10,84	11,21	**12,70**	**12,90**	0,00	2,31	2,77
1580939	*P. sanguineus*	AA9		31	11,10	11,59	**12,11**	**12,86**	0,00	1,52	1,98
1370357	*P. coccineus*	CBM21		31	11,37	11,68	**13,40**	**11,64**	0,00	2,40	0,00
1593166	*P. sanguineus*	CE4	yes	31	11,37	11,77	**12,64**	**12,38**	0,00	1,69	0,00
1588377	*P. sanguineus*	GH125		31	10,79	10,21	**12,82**	**12,61**	0,00	2,44	2,07
1507511	*P. coccineus*	GH15	yes	31	10,91	10,34	**12,07**	**11,48**	0,00	1,62	0,00
1372016	*P. coccineus*	GH16	yes	31	11,13	10,59	**12,33**	**11,75**	0,00	1,61	1,27
1465130	*P. coccineus*	GH16		31	11,45	11,18	**11,87**	**12,14**	0,00	0,82	1,33
651762	*P. coccineus*	GH16		31	10,91	11,78	**12,13**	**11,81**	1,57	1,70	1,58
1445051	*P. coccineus*	GH18	yes	31	11,75	10,80	**12,75**	**12,98**	0,00	0,00	1,80
1609752	*P. sanguineus*	GH18		31	10,27	12,33	**13,08**	**12,17**	2,59	2,66	0,00
1672749	*P. sanguineus*	GH18	yes	31	11,55	12,06	**12,49**	**12,62**	0,00	0,00	0,00
1664834	*P. sanguineus*	GH2	yes	31	11,25	10,74	**12,89**	**11,91**	0,00	1,97	0,00
2449	*P. cinnabarinus*	GH23		31	11,57	10,50	**12,25**	**12,51**	0,00	1,11	1,25
1440257	*P. coccineus*	GH27	yes	31	10,59	12,13	**11,70**	**12,83**	1,94	0,00	2,94
1589244	*P. sanguineus*	GH27	yes	31	10,47	10,74	**12,03**	**11,62**	0,00	0,00	0,00
1673487	*P. sanguineus*	GH3		31	11,00	12,81	**12,70**	**11,37**	2,52	2,09	0,00
1435128	*P. coccineus*	GH31		31	11,13	11,06	**12,18**	**12,06**	0,00	1,46	1,57
1705196	*P. sanguineus*	GH31	yes	31	11,94	11,70	**13,28**	**12,33**	0,00	1,75	0,00

6931	*P. cinnabarinus*	GH37	yes	31	11,88	10,26	**12,81**	**11,60**	0,00	1,27	0,00
1677131	*P. sanguineus*	GH47		31	11,13	12,42	**12,14**	**11,77**	1,86	1,42	0,00
2526	*P. cinnabarinus*	GH79		31	11,36	11,73	**12,35**	**12,55**	0,93	1,39	1,48
4974	*P. cinnabarinus*	GH79		31	11,59	11,09	**12,88**	**12,06**	0,00	1,55	0,00
1721241	*P. sanguineus*	GH92	yes	31	11,03	10,87	**12,61**	**12,78**	0,00	0,00	0,00
929	*P. cinnabarinus*	GH95	yes	31	11,35	10,93	**13,03**	**13,23**	0,00	1,90	1,97
1676887	*P. sanguineus*	GST GTT2.1		31	11,84	11,44	**12,69**	**12,01**	0,00	0,00	0,00
1364471	*P. coccineus*	GT32		31	12,37	11,03	**12,67**	**12,93**	0,00	0,00	0,00
5123	*P. cinnabarinus*	HTP		31	11,31	11,55	**11,55**	**13,07**	0,00	0,00	1,90
1360037	*P. coccineus*	Peptidase A1		31	11,78	11,92	**12,80**	**12,75**	0,00	1,35	1,58
1370947	*P. coccineus*	Peptidase A1	yes	31	10,76	10,65	**12,51**	**13,35**	0,00	2,22	3,35
1464014	*P. coccineus*	Peptidase A1		31	12,08	11,26	**12,52**	**12,61**	0,00	0,00	1,14
1682142	*P. sanguineus*	Peptidase A1		31	10,46	11,13	**12,71**	**11,08**	0,00	2,27	0,00
5737	*P. cinnabarinus*	aspartic		31	11,53	10,95	**12,23**	**12,39**	0,00	1,14	1,20
8347	*P. cinnabarinus*	Peptidase M		31	11,81	11,53	**12,29**	**12,15**	0,00	0,92	0,73
1434129	*P. coccineus*	Peptidase M28		31	10,88	11,36	**12,00**	**11,77**	1,08	1,63	1,58
1438826	*P. coccineus*	Peptidase S10		31	10,57	10,99	**12,25**	**11,55**	0,00	2,21	1,72
1447902	*P. coccineus*	Peptidase S28		31	11,68	10,04	**12,74**	**12,15**	0,00	0,00	0,00
1589769	*P. sanguineus*	Peptidase S33		31	11,16	12,24	**12,49**	**11,39**	1,50	1,64	0,00
1617673	*P. sanguineus*	Peptidase S41	yes	31	11,00	10,31	**12,02**	**11,56**	0,00	1,54	0,89
1468659	*P. coccineus*	Peptidase S53		31	11,30	11,92	**12,50**	**12,71**	0,00	1,66	2,07
2284	*P. cinnabarinus*	Peptidase S53	yes	31	11,93	9,81	**12,86**	**12,22**	-1,30	1,28	0,00
672392	*P. coccineus*	Peptidase S53	yes	31	10,99	12,05	**12,32**	**11,38**	1,80	1,76	0,00
67779	*P. sanguineus*	PL14_4		31	11,48	11,51	**12,17**	**12,48**	0,00	0,00	0,00
MEAN node 31		0,88			11,26	11,26	**12,49**	**12,22**	0,35	1,27	0,88
1560767	*P. sanguineus*	AA1_1	yes	32	9,78	7,13	**11,34**	**13,24**	0,00	**0,00**	**3,06**
1480943	*P. coccineus*	AA5_1		32	8,83	8,60	**12,25**	**13,71**	0,00	**3,88**	**5,57**
1433098	*P. coccineus*	CE4		32	10,31	9,14	**12,28**	**11,31**	0,00	**2,51**	**1,69**
1594881	*P. sanguineus*	GH18	yes	32	9,67	9,88	**12,37**	**12,41**	0,00	**3,23**	**3,14**
1092	*P. cinnabarinus*	GH2	yes	32	9,14	8,81	**12,68**	**12,64**	0,00	**3,65**	**3,56**
1675320	*P. sanguineus*	GH25	yes	32	9,46	8,23	**11,92**	**12,06**	0,00	**0,00**	**0,00**
1577230	*P. sanguineus*	GH28	yes	32	8,59	9,32	**12,42**	**12,18**	0,00	**4,26**	**3,86**
4566	*P. cinnabarinus*	GH3	yes	32	10,07	9,64	**13,50**	**11,79**	0,00	**3,46**	**1,98**
1680674	*P. sanguineus*	GH30_3		32	9,84	9,23	**11,88**	**11,71**	0,00	**2,28**	**0,00**
6130	*P. cinnabarinus*	GH31	yes	32	9,94	8,97	**11,68**	**11,87**	0,00	**2,11**	**2,19**
9738	*P. cinnabarinus*	GH31	yes	32	10,48	9,29	**13,68**	**12,59**	0,00	**3,27**	**2,29**
1695354	*P. sanguineus*	GH43	yes	32	8,08	8,02	**12,42**	**13,20**	0,00	**4,23**	**4,90**
1575750	*P. sanguineus*	GH76	yes	32	9,19	9,13	**12,57**	**11,65**	0,00	**3,70**	**2,71**
8219	*P. cinnabarinus*	GH88	yes	32	9,89	10,25	**12,21**	**12,68**	0,00	**2,57**	**2,89**
1609029	*P. sanguineus*	GH92		32	9,69	9,08	**12,98**	**13,90**	0,00	**3,30**	**3,91**
1592999	*P. sanguineus*	PL8_4		32	8,85	10,09	**11,99**	**12,06**	1,90	**3,65**	**3,57**
MEAN node 32					9,49	9,05	**12,39**	**12,44**	0,12	**2,88**	**2,83**
1436321	*P. coccineus*	AA2 / MnP short	yes	40	7,27	7,10	**13,86**	**12,92**	NA	**6,80**	**6,26**
1468611	*P. coccineus*	AA2 / MnP short		40	6,51	6,46	**14,34**	**15,39**	NA	**8,27**	**9,71**
6860	*P. cinnabarinus*	GH92	yes	40	9,31	8,79	**14,51**	**15,16**	NA	**4,71**	**5,20**
MEAN node 40					7,69	7,45	**14,23**	**14,49**	NA	**6,59**	**7,06**
1464049	*P. coccineus*	AA2 / MnP short	yes	41	7,40	5,78	10,46	13,35	0,00	**3,87**	**6,91**
1376269	*P. coccineus*	AA5_1		41	6,74	6,39	11,80	11,74	0,00	**5,73**	**5,90**
1454649	*P. coccineus*	AA5_1		41	6,03	7,75	10,19	10,87	0,00	**5,13**	**5,67**
4318	*P. cinnabarinus*	CE15		41	7,35	7,04	10,02	10,22	0,00	**3,02**	**3,12**
7984	*P. cinnabarinus*	GH28	yes	41	7,77	7,28	10,29	10,74	0,00	**2,86**	**3,13**
7022	*P. cinnabarinus*	GH35	yes	41	6,89	8,20	11,93	9,16	1,68	**4,97**	**2,68**
1420090	*P. coccineus*	Peptidase aspartic		41	8,04	7,03	11,60	11,38	0,00	**4,39**	**4,30**
5703	*P. cinnabarinus*	Peptidase aspartic		41	7,08	6,88	9,96	10,53	0,00	**3,10**	**3,46**
1471389	*P. coccineus*	Peptidase S53		41	7,55	7,49	11,92	10,25	0,00	**5,21**	**3,64**
689	*P. cinnabarinus*	Peptidase S53	yes	41	6,25	6,17	11,97	10,46	0,00	**5,52**	**4,19**
MEAN node 41					7,11	7,00	11,01	10,87	0,17	**4,38**	**4,30**
1594971	*P. sanguineus*	CE16		42	6,05	6,41	7,92	8,59	0,00	**2,43**	**3,09**
1611075	*P. sanguineus*	CE8		42	6,94	8,12	9,10	8,74	0,00	**2,63**	**2,33**
1419320	*P. coccineus*	GH10		42	6,15	8,20	7,72	11,24	2,55	**2,61**	**5,97**
1597080	*P. sanguineus*	GH115		42	7,23	6,79	8,80	7,84	0,00	**0,00**	**0,00**

1559984	*P. sanguineus*	GH16		42	7,09	7,71	8,88	9,39	0,00	2,47	2,97
1463187	*P. coccineus*	GH18	yes	42	7,99	7,13	9,39	8,02	0,00	2,23	0,00
1465855	*P. sanguineus*	GH28		42	6,36	6,81	9,86	9,37	0,00	4,03	3,55
826	*P. cinnabarinus*	GH28	yes	42	7,58	6,33	8,62	8,99	0,00	1,69	2,00
1439507	*P. coccineus*	GH35		42	7,47	8,40	9,15	9,30	0,00	2,36	2,63
5750	*P. cinnabarinus*	GH76	yes	42	7,77	6,72	9,27	10,02	0,00	2,07	2,61
5751	*P. cinnabarinus*	GH76	yes	42	7,39	7,23	8,83	9,63	0,00	2,02	2,64
1067038	*P. coccineus*	GH78	yes	42	7,80	6,29	8,92	9,13	0,00	2,25	2,31
1470054	*P. coccineus*	HTP		42	6,19	7,38	8,00	9,92	0,00	2,83	4,79
1149518	*P. coccineus*	Peptidase A1		42	6,63	8,23	8,69	8,55	2,27	3,03	3,05
925665	*P. sanguineus*	Peptidase aspartic		42	7,21	7,18	8,15	8,85	0,00	0,00	0,00
1322	*P. cinnabarinus*	Peptidase S33		42	6,54	7,42	9,70	8,99	0,00	3,33	2,74
1436433	*P. coccineus*	Peptidase S53		42	7,81	6,91	9,09	9,09	0,00	2,19	2,28
1425521	*P. coccineus*	PL14_4		42	5,82	6,82	8,71	8,71	0,00	3,52	3,63
MEAN node 42					7,00	7,23	8,82	9,13	0,27	2,32	2,59
1452465	*P. coccineus*	AA1_1	yes	43	7,23	5,60	9,78	6,53	NA	3,84	0,00
10400	*P. cinnabarinus*	EXPN		43	6,72	5,93	10,43	7,67	NA	3,81	1,62
2894	*P. cinnabarinus*	EXPN		43	5,77	4,56	8,76	6,86	NA	3,16	1,84
4818	*P. cinnabarinus*	GH43		43	5,50	5,46	11,82	7,21	NA	5,98	2,27
8122	*P. cinnabarinus*	GH51		43	6,01	5,81	9,11	7,78	NA	3,38	2,36
MEAN node 43					6,25	5,47	9,98	7,21	NA	4,03	1,62
3446	*P. cinnabarinus*	AA1_1	yes	48	10,76	10,73	15,23	12,95	0,00	4,12	2,32
4432	*P. cinnabarinus*	CE4		48	9,97	11,66	12,92	12,58	2,11	3,10	2,76
1595466	*P. sanguineus*	EXPN		48	10,89	12,54	13,10	13,46	2,30	2,38	2,49
6051	*P. cinnabarinus*	EXPN	yes	48	8,91	11,92	13,95	12,83	3,25	4,81	3,83
1682233	*P. sanguineus*	GH13_1		48	11,23	12,61	13,30	13,32	1,96	2,40	2,22
1445188	*P. coccineus*	GH16		48	10,35	12,40	13,11	12,54	2,79	3,27	2,85
7245	*P. cinnabarinus*	GH27	yes	48	11,89	11,16	13,70	13,83	0,00	2,05	2,05
1750423	*P. sanguineus*	GH28	yes	48	10,79	13,25	13,52	12,94	3,03	0,00	0,00
3798	*P. cinnabarinus*	GH28	yes	48	11,03	10,18	13,36	13,52	0,00	2,52	2,56
1439531	*P. coccineus*	GH35	yes	48	9,91	11,77	13,15	13,32	2,41	3,82	4,18
1590027	*P. sanguineus*	GH35	yes	48	10,42	11,21	14,27	14,06	0,00	3,78	3,50
692	*P. cinnabarinus*	GH47	yes	48	9,58	12,09	12,91	12,75	2,97	3,38	3,18
1593425	*P. sanguineus*	GH5_15		48	10,85	12,20	12,83	13,28	0,00	0,00	0,00
1357629	*P. coccineus*	GH51	yes	48	9,18	11,14	13,41	13,10	2,29	4,84	4,67
1571620	*P. sanguineus*	GH51	yes	48	10,36	11,92	14,23	14,13	2,54	3,84	3,63
7421	*P. cinnabarinus*	GH55	yes	48	10,36	13,00	12,95	15,87	3,12	2,69	4,68
1560745	*P. sanguineus*	GH79	yes	48	11,01	11,44	13,96	14,27	0,00	3,21	3,19
8220	*P. cinnabarinus*	GH88	yes	48	10,74	11,90	13,23	13,89	1,59	2,64	3,09
1663870	*P. sanguineus*	GH92	yes	48	10,36	12,17	13,56	14,18	2,41	3,47	3,77
598	*P. cinnabarinus*	GH92	yes	48	11,05	11,67	13,43	13,60	1,23	2,48	2,54
1572980	*P. sanguineus*	Peptidase M35	yes	48	10,72	11,90	14,32	14,15	2,03	3,77	3,36
MEAN node 48					10,49	11,85	13,54	13,55	1,72	2,98	2,90
1431101	*P. coccineus*	AA2/LiP		51	6,04	5,35	9,27	15,59	0,00	4,25	10,56
1377160	*P. coccineus*	CBM1-CE1		51	6,48	7,30	7,90	13,09	0,00	2,33	7,72
1395316	*P. coccineus*	GH10	yes	51	6,12	5,98	6,44	13,06	0,00	0,00	8,12
1468135	*P. coccineus*	hydrophobin		51	3,64	7,48	7,20	13,91	3,85	3,82	10,55
MEAN node 51					5,57	6,53	7,70	13,91	0,96	2,60	9,24
1583166	*P. sanguineus*	AA1_1	yes	56	13,18	11,94	15,00	16,16	0,00	0,00	2,59
1390248	*P. coccineus*	CBM12		56	13,23	13,78	14,95	14,45	1,31	1,70	1,75
1561087	*P. sanguineus*	CBM12		56	13,28	11,72	13,32	15,10	0,00	0,00	0,00
10255	*P. cinnabarinus*	GH125	yes	56	12,47	11,07	13,32	14,63	0,00	1,20	2,15
1674360	*P. sanguineus*	GH16	yes	56	11,93	12,17	14,48	14,80	0,95	2,89	3,00
5804	*P. cinnabarinus*	GH16	yes	56	12,70	12,34	14,91	15,24	0,00	2,14	2,39
1233	*P. cinnabarinus*	GH17	yes	56	11,89	12,69	14,32	14,84	1,47	2,38	2,74
6004	*P. cinnabarinus*	GH18	yes	56	11,55	14,07	14,54	14,16	3,13	2,83	2,56
1435894	*P. coccineus*	GH79	yes	56	11,11	14,27	13,98	15,04	3,99	3,23	4,63
5180	*P. cinnabarinus*	GH79		56	12,51	13,11	14,14	14,28	1,21	1,81	1,85
380332	*P. coccineus*	hydrophobin		56	13,04	14,66	13,76	16,33	2,64	0,00	3,48
421	*P. cinnabarinus*	Peptidase A1		56	12,66	12,83	13,91	13,78	0,00	1,50	1,33
6880	*P. cinnabarinus*	Peptidase aspartic		56	12,52	12,37	14,85	15,64	0,00	2,29	2,90
6154	*P. cinnabarinus*	Peptidase M		56	12,59	11,93	13,81	14,05	0,00	1,52	1,60
1465736	*P. coccineus*	Peptidase S10	yes	56	11,60	13,68	13,95	14,22	2,74	2,61	3,24

1407107	*P. coccineus*	Peptidase S53	yes	56	12,26	14,24	**14,77**	**14,70**	2,80	2,64	**3,05**
1562796	*P. sanguineus*	Peptidase S53	yes	56	13,35	12,23	**14,68**	**14,37**	0,00	0,00	**0,00**
1602760	*P. sanguineus*	Peptidase S53	yes	56	13,20	12,35	**14,59**	**14,48**	0,00	0,00	**0,00**
MEAN node 56					12,50	12,86	**14,29**	**14,79**	1,12	1,60	**2,18**
1439081	*P. coccineus*	CBM13		61	4,84	6,66	6,86	8,58	2,28	**2,75**	**4,59**
7437	*P. cinnabarinus*	CBM1-CE1		61	2,99	6,16	5,75	9,14	2,71	**2,38**	**5,33**
1367487	*P. coccineus*	CE16		61	4,18	7,49	7,42	9,79	3,16	**3,66**	**6,07**
1378034	*P. coccineus*	GH45		61	4,10	7,84	6,52	7,72	3,98	**2,96**	**4,26**
1462467	*P. coccineus*	GH51		61	4,13	5,97	8,76	8,55	0,00	**5,00**	**4,92**
1393742	*P. coccineus*	GST GHR		61	4,65	6,02	8,07	7,24	2,03	**4,14**	**3,49**
1467651	*P. coccineus*	GST omega		61	4,91	6,94	7,37	6,75	2,56	**3,42**	**2,79**
1677288	*P. sanguineus*	Peptidase A1		61	5,70	6,52	7,56	8,32	0,00	**2,28**	**3,12**
465	*P. cinnabarinus*	Peptidase M28	yes	61	3,91	4,71	6,17	8,42	0,00	**2,19**	**4,25**
MEAN node 61					4,38	6,48	7,16	8,28	1,86	**3,20**	**4,31**

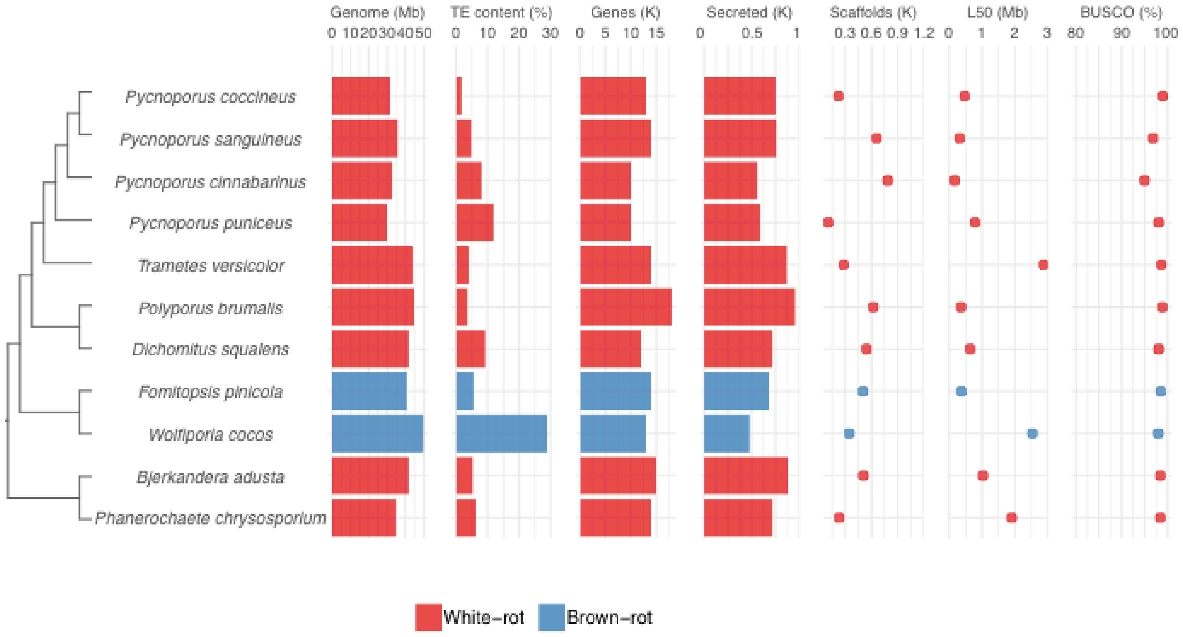

Fig S1. Genomic features of 11 fungi. Colors in the plots represent different rot types. Genome: Genome size. TE content: The coverage of transposable elements in the genomes. Genes: The number of genes. Secreted: The number of predicted secreted proteins (see Methods). Scaffolds: The number of scaffolds. L50: N50 length. BUSCO: Genome completeness. See details in supplementary Table S18.

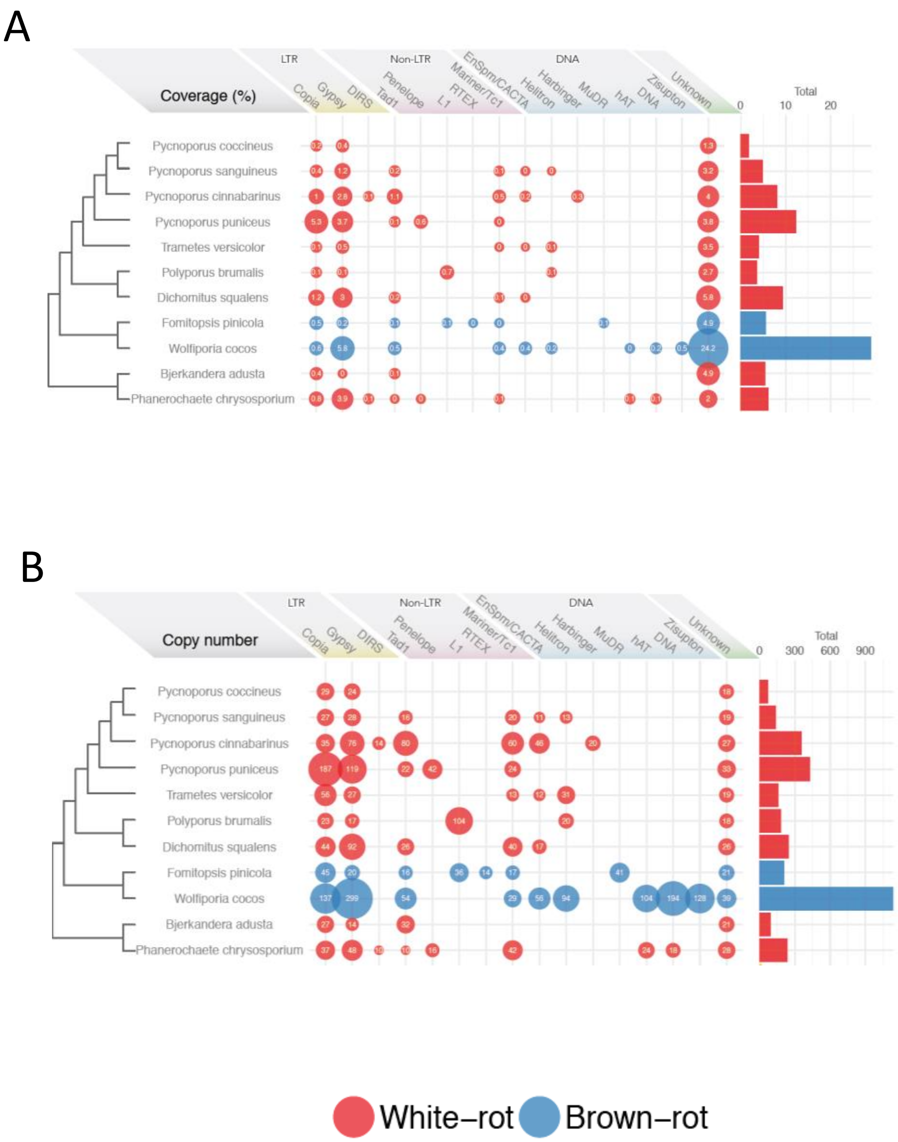

Fig S2. Transposable elements present in 11 Polyporales genomes. The coverage of transposable elements in the genomes (A) and the copy number (B) are shown. LTR: Long terminal repeat retrotransposons. Non-LTR: Non-long terminal repeat retrotransposons. DNA: DNA transposons. Unknown: Unclassified repeated sequences. The bubble size is proportional to the genome coverage by each transposable element (showing inside the bubbles). The bars on right show the total coverage per genome.

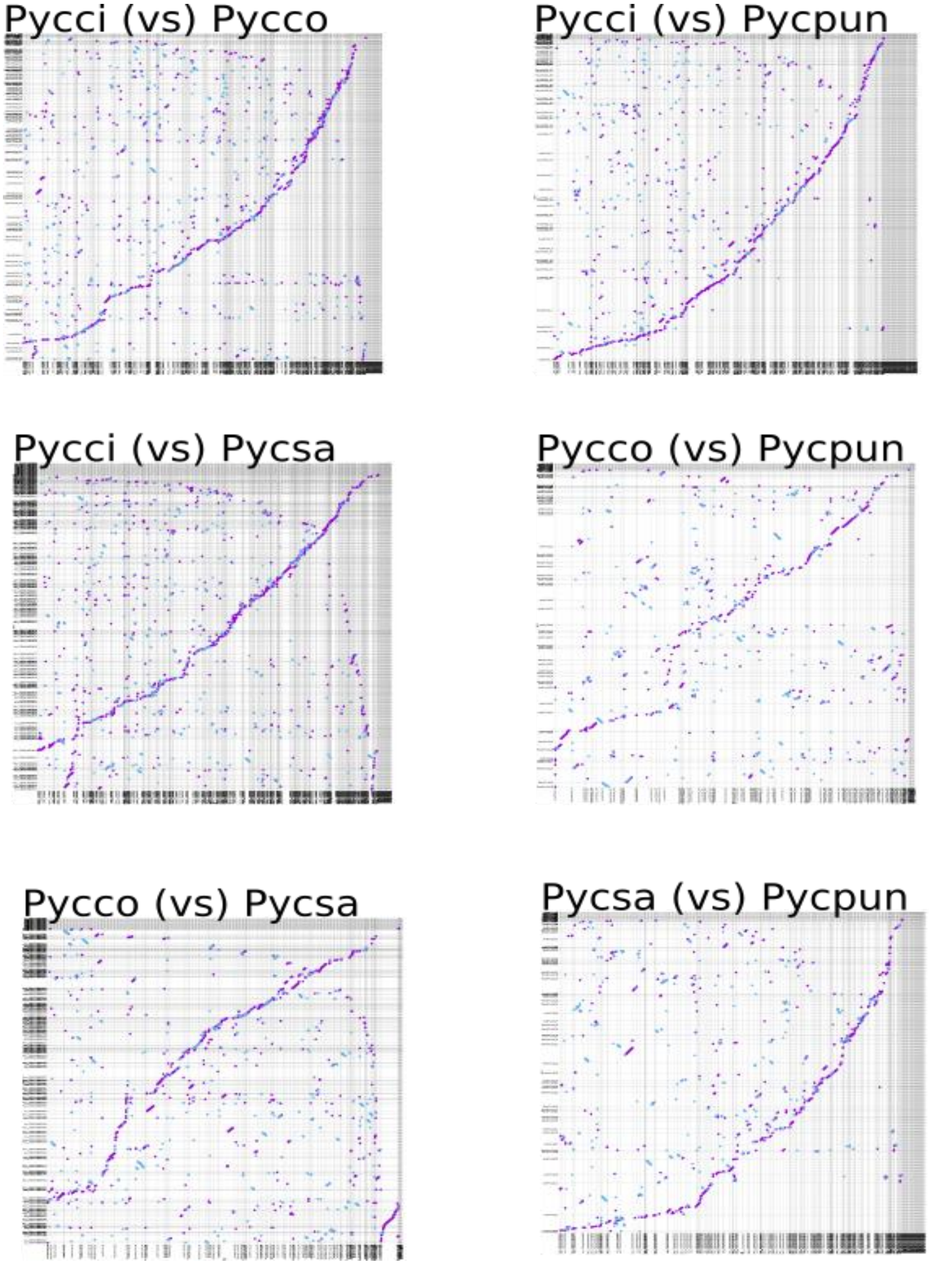

Fig. S3. Dot plot representation of the pair-wise alignments of Pycci, Pycco, Pycsa and Pycpun scaffolds generated by Mummer (Kurtz et al., 2004). Pycci: *P. cinnabarinus* BRFM 137, Pycco: *P. coccineus* BRFM 310, Pycsa: *P. sanguineus* BRFM 1264

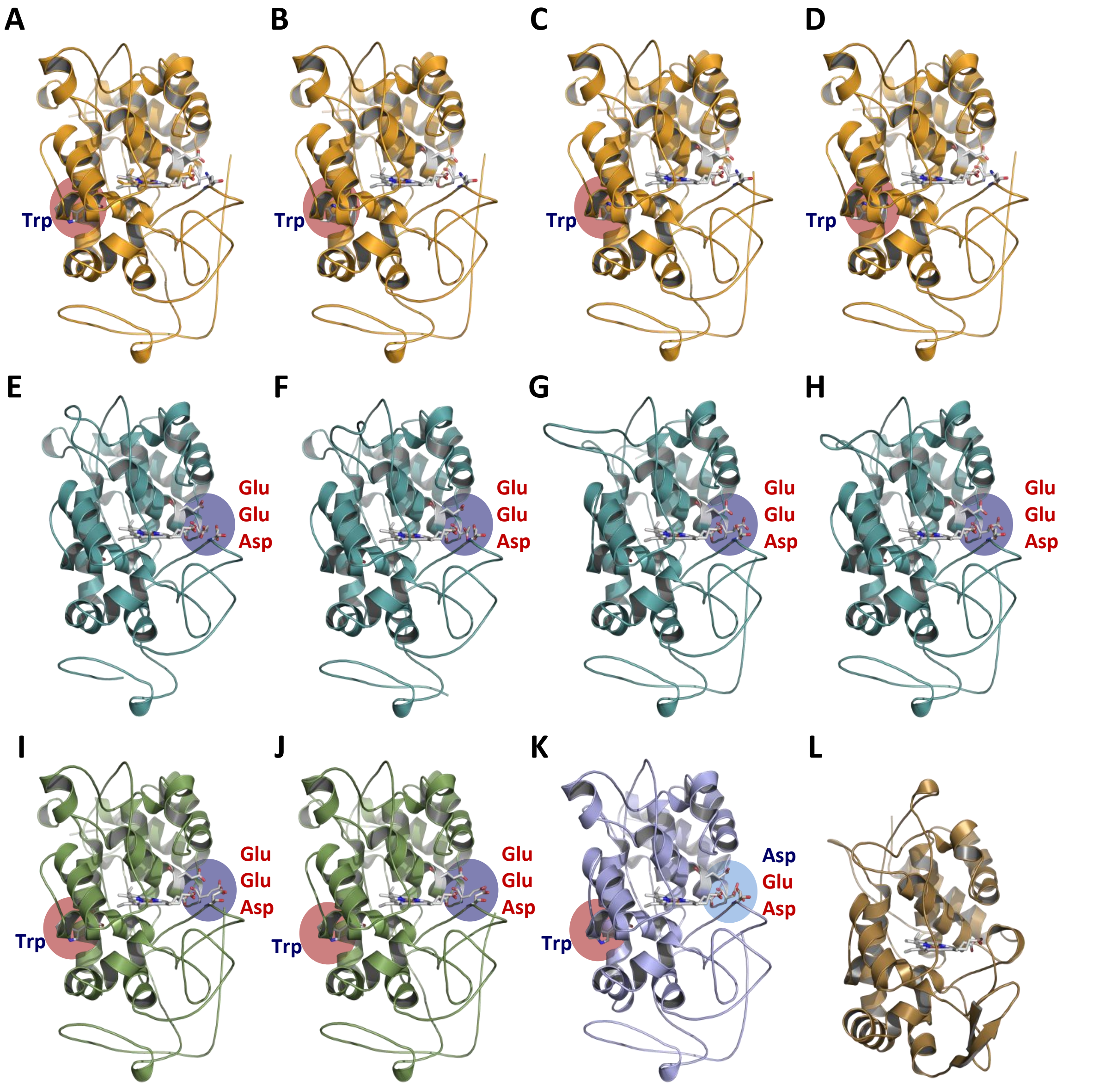

Fig. S4. Homology models for the molecular structures of class I and II heme peroxidases from the *P. coccineus* BRFM 310 genome. Ligninolytic peroxidases, including LiP models - A) LiP1 (1431101), B) LiP2 (1403742), C) LiP3 (779035) and D) LiP4 (859168) - harboring an exposed tryptophan potentially involved in oxidation of high redox-potential substrates, MnP models - E) MnP1-short (1468611), F) MnP2-short (1436321), G) MnP3-short (1464049) and H) MnP4-short (1369658) - harboring a putative Mn^{2+} oxidation site (formed by two glutamates and one aspartate), two VP models - I) VP1 (1468768) and J) VP2 (1469331) - harboring the two catalytic sites described for LiPs and MnPs, one atypical VP - K) VP-atypical (1438352) containing an atypical Mn^{2+} oxidation site formed by two aspartates and one glutamate; and L) CCP (1449695).

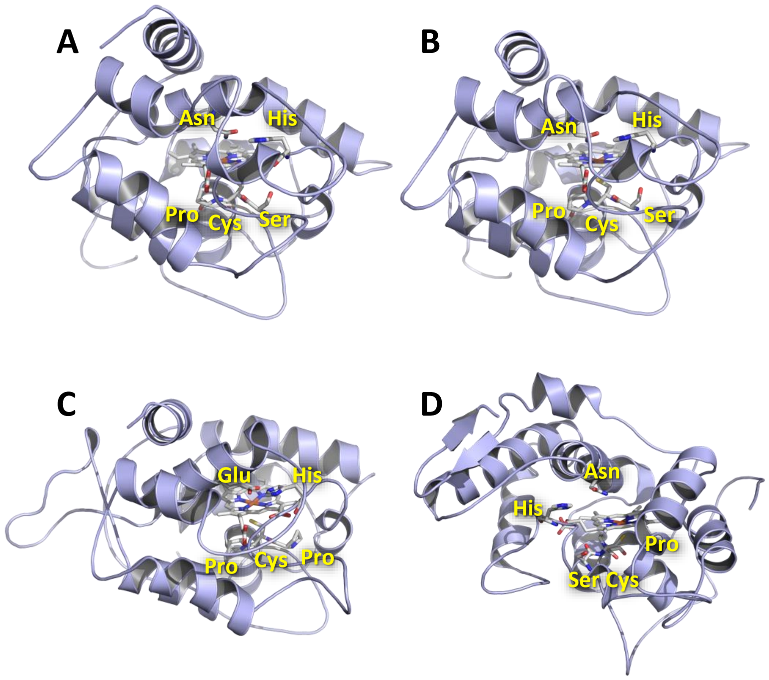

Fig. S5. Homology models for the molecular structures of heme-thiolate peroxidases (HTPs) from the *P. coccineus* BRFM 310 genome. A) HTP1 (1438096), B) HTP2 (1438101), C) HTP3 (1374080) and C) HTP4 (1470054) including proximal cysteine residue acting as the fifth heme iron ligand and a few more amino acid residues of the active center.

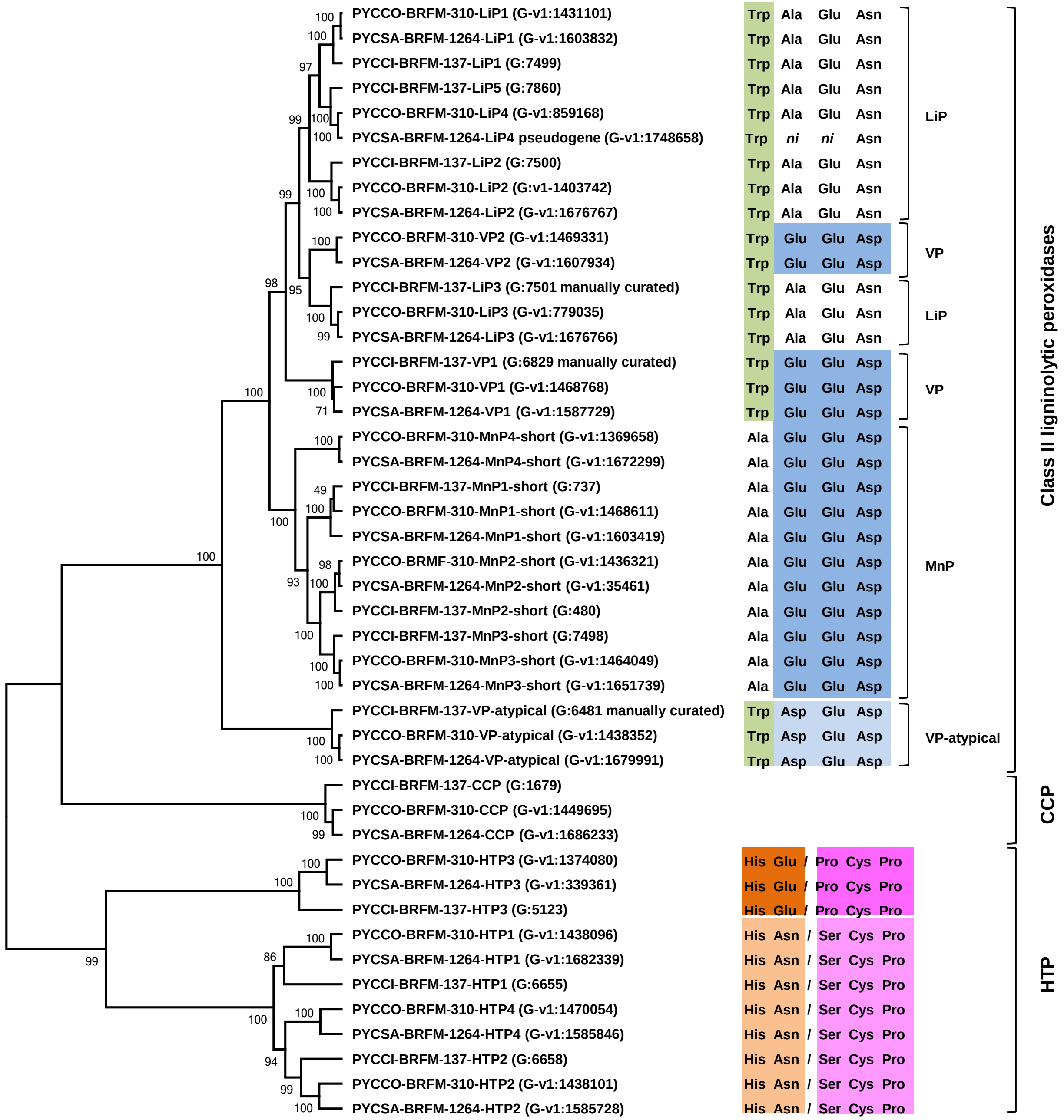

Fig. S6. Dendrogram of sixty deduced heme peroxidase sequences (and one lip pseudogene) (JGI's ID numbers of the annotated genomes, version 1.0, in brackets) from three *Pycnoporus* species. The dendrogram shows sequence relationships and structural-functional classification of the ligninolytic peroxidases (short MnPs have a Mn^{2+}-oxidation site formed by two glutamate and one aspartate residues, shown on blue background; LiPs contain a catalytic tryptophan, shown on green background; VPs harbor the catalytic sites described for both MnPs and LiPs; and atypical VPs have an aspartate residue in the position occupied by one of the glutamic residues at the Mn^{2+}-oxidation site, shown on pale blue background), and provides the residues homologous to L. fumago CPO/A. aegerita UPO heme distal residues (His105/Arg189 and Glu183/Glu196) (shown on orange background) and heme proximal residues (Pro28/Pro35, Cys29/Cys36 and Pro30/Pro37) (shown on pink background) identified in the HTPs. Sequence comparison as Poisson distances, and clustering using the UPGMA method and ‘‘pairwise deletion’’ option of MEGA5 . Numbers on branches represent bootstrap values (based on 1000 replications) supporting that branch. Only the values ≥50% are presented.

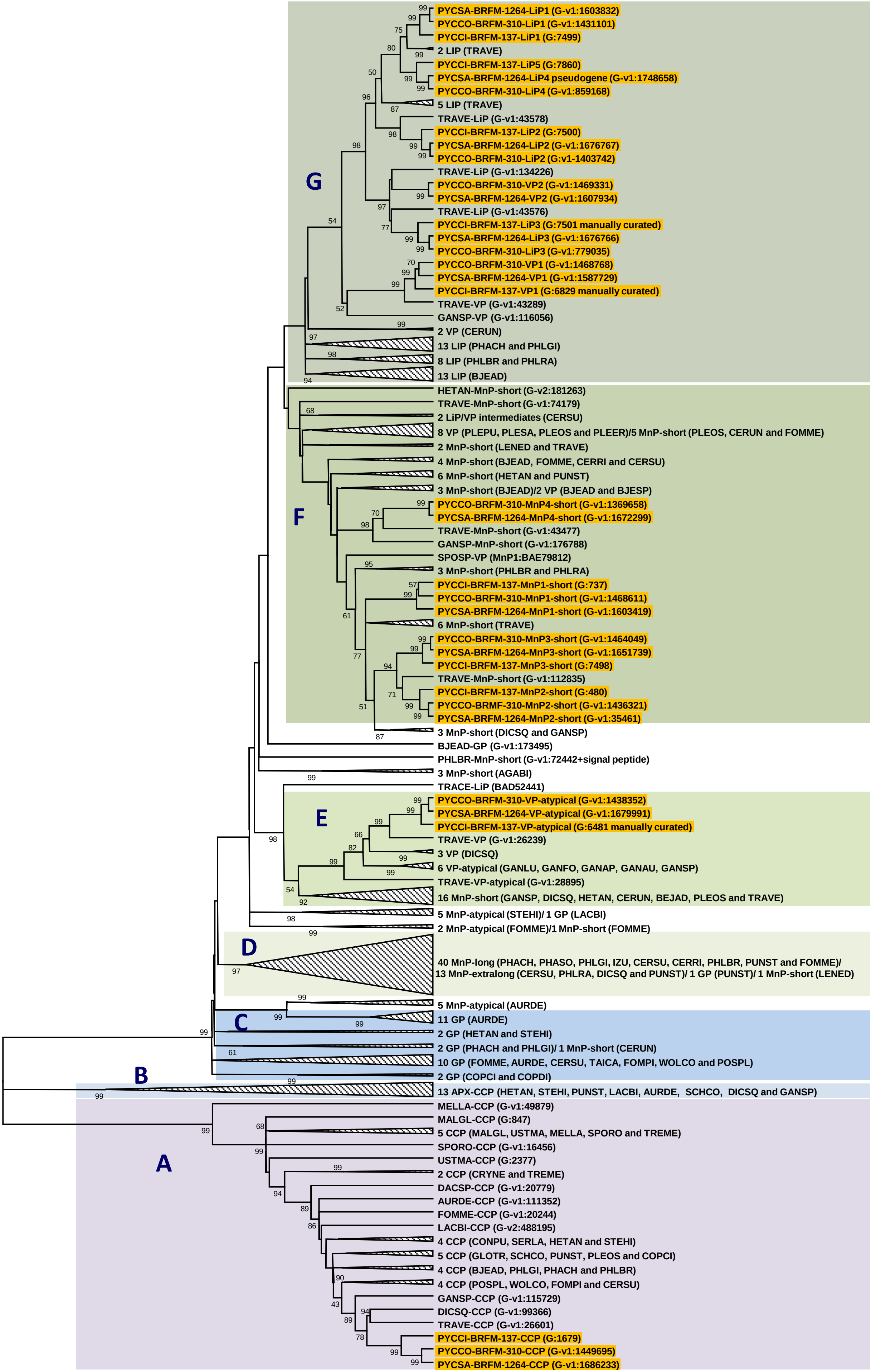
G
PYCSA-BRFM-1264-LiP1 (G-v1:1603832)
PYCCO-BRFM-310-LiP1 (G-v1:1431101)
PYCCI-BRFM-137-LiP1 (G:7499)
2 LIP (TRAVE)
PYCCI-BRFM-137-LiP5 (G:7860)
PYCSA-BRFM-1264-LiP4 pseudogene (G-v1:1748658)
PYCCO-BRFM-310-LiP4 (G-v1:859168)
5 LIP (TRAVE)
TRAVE-LiP (G-v1:43578)
PYCCI-BRFM-137-LiP2 (G:7500)
PYCSA-BRFM-1264-LiP2 (G-v1:1676767)
PYCCO-BRFM-310-LiP2 (G:v1-1403742)
TRAVE-LiP (G-v1:134226)
PYCCO-BRFM-310-VP2 (G-v1:1469331)
PYCSA-BRFM-1264-VP2 (G-v1:1607934)
TRAVE-LiP (G-v1:43576)
PYCCI-BRFM-137-LiP3 (G:7501 manually curated)
PYCSA-BRFM-1264-LiP3 (G-v1:1676766)
PYCCO-BRFM-310-LiP3 (G-v1:779035)
PYCCO-BRFM-310-VP1 (G-v1:1468768)
PYCSA-BRFM-1264-VP1 (G-v1:1587729)
PYCCI-BRFM-137-VP1 (G:6829 manually curated)
TRAVE-VP (G-v1:43289)
GANSP-VP (G-v1:116056)
2 VP (CERUN)
13 LIP (PHACH and PHLGI)
8 LIP (PHLBR and PHLRA)
13 LIP (BJEAD)
F
HETAN-MnP-short (G-v2:181263)
TRAVE-MnP-short (G-v1:74179)
2 LiP/VP intermediates (CERSU)
8 VP (PLEPU, PLESA, PLEOS and PLEER)/5 MnP-short (PLEOS, CERUN and FOMME)
2 MnP-short (LENED and TRAVE)
4 MnP-short (BJEAD, FOMME, CERRI and CERSU)
6 MnP-short (HETAN and PUNST)
3 MnP-short (BJEAD)/2 VP (BJEAD and BJESP)
PYCCO-BRFM-310-MnP4-short (G-v1:1369658)
PYCSA-BRFM-1264-MnP4-short (G-v1:1672299)
TRAVE-MnP-short (G-v1:43477)
GANSP-MnP-short (G-v1:176788)
SPOSP-VP (MnP1:BAE79812)
3 MnP-short (PHLBR and PHLRA)
PYCCI-BRFM-137-MnP1-short (G:737)
PYCCO-BRFM-310-MnP1-short (G-v1:1468611)
PYCSA-BRFM-1264-MnP1-short (G-v1:1603419)
6 MnP-short (TRAVE)
PYCCO-BRFM-310-MnP3-short (G-v1:1464049)
PYCSA-BRFM-1264-MnP3-short (G-v1:1651739)
PYCCI-BRFM-137-MnP3-short (G:7498)
TRAVE-MnP-short (G-v1:112835)
PYCCI-BRFM-137-MnP2-short (G:480)
PYCCO-BRMF-310-MnP2-short (G-v1:1436321)
PYCSA-BRFM-1264-MnP2-short (G-v1:35461)
3 MnP-short (DICSQ and GANSP)
BJEAD-GP (G-v1:173495)
PHLBR-MnP-short (G-v1:72442+signal peptide)
3 MnP-short (AGABI)
TRACE-LiP (BAD52441)
E
PYCCO-BRFM-310-VP-atypical (G-v1:1438352)
PYCSA-BRFM-1264-VP-atypical (G-v1:1679991)
PYCCI-BRFM-137-VP-atypical (G:6481 manually curated)
TRAVE-VP (G-v1:26239)
3 VP (DICSQ)
6 VP-atypical (GANLU, GANFO, GANAP, GANAU, GANSP)
TRAVE-VP-atypical (G-v1:28895)
16 MnP-short (GANSP, DICSQ, HETAN, CERUN, BEJAD, PLEOS and TRAVE)
5 MnP-atypical (STEHI)/ 1 GP (LACBI)
2 MnP-atypical (FOMME)/1 MnP-short (FOMME)
D
40 MnP-long (PHACH, PHASO, PHLGI, IZU, CERSU, CERRI, PHLBR, PUNST and FOMME)/
13 MnP-extralong (CERSU, PHLRA, DICSQ and PUNST)/ 1 GP (PUNST)/ 1 MnP-short (LENED)
5 MnP-atypical (AURDE)
C
11 GP (AURDE)
2 GP (HETAN and STEHI)
2 GP (PHACH and PHLGI)/ 1 MnP-short (CERUN)
10 GP (FOMME, AURDE, CERSU, TAICA, FOMPI, WOLCO and POSPL)
2 GP (COPCI and COPDI)
B
13 APX-CCP (HETAN, STEHI, PUNST, LACBI, AURDE, SCHCO, DICSQ and GANSP)
A
MELLA-CCP (G-v1:49879)
MALGL-CCP (G:847)
5 CCP (MALGL, USTMA, MELLA, SPORO and TREME)
SPORO-CCP (G-v1:16456)
USTMA-CCP (G:2377)
2 CCP (CRYNE and TREME)
DACSP-CCP (G-v1:20779)
AURDE-CCP (G-v1:111352)
FOMME-CCP (G-v1:20244)
LACBI-CCP (G-v2:488195)
4 CCP (CONPU, SERLA, HETAN and STEHI)
5 CCP (GLOTR, SCHCO, PUNST, PLEOS and COPCI)
4 CCP (BJEAD, PHLGI, PHACH and PHLBR)
4 CCP (POSPL, WOLCO, FOMPI and CERSU)
GANSP-CCP (G-v1:115729)
DICSQ-CCP (G-v1:99366)
TRAVE-CCP (G-v1:26601)
PYCCI-BRFM-137-CCP (G:1679)
PYCCO-BRFM-310-CCP (G-v1:1449695)
PYCSA-BRFM-1264-CCP (G-v1:1686233)

Fig. S7. Dendrogram showing evolutionary relationships among 310 basidiomycete Class I and Class II heme peroxidases, including structural-functional classification of Class II heme peroxidases based on Ruiz-Dueñas et al. (2009) (GeneBank and JGI identification numbers in parentheses and *Pycnoporus* strains genome identification numbers on orange background). Amino-acid sequence comparisons as Poisson distances and clustering based on UPGMA and "pairwise deletion" option of MEGA5 (Tamura et al. 2011). Compressed sub-trees are shown to facilitate the *Pycnoporus* strains peroxidases analysis. Numbers on branches represent bootstrap values (based on 1000 replications) supporting that branch; only the values ≥50% are presented. Fungal abbreviations: AGABI, *Agaricus bisporus*; AURDE, *Auricularia delicata*; BJEAD, *Bjerkandera adusta*; BJESP, *Bjerkandera* sp; CERRI, *Ceriporiopsis rivulosa*; CERSU, *Ceriporiopsis subvermispora*-B; CERUN, *Cerrena unicolor*; COPCI, *Coprinopsis cinerea*; CONPU, *Coniophora puteana* v1.0; COPDI, *Coprinellus disseminatus*; CRYNE, *Cryptococcus neoformans* var. *grubii* H99; DACSP, *Dacryopinax* sp.; DICSQ, *Dichomitus squalens* v1.0; FOMME, *Fomitiporia mediterranea* v1.0; FOMPI, *Fomitopsis pinicola* SS1 v1.0; GANSP, *Ganoderma* sp.; GLOTR, *Gloeophyllum trabeum* v1.0; HETAN, *Heterobasidion annosum* v2.0; IZU, basidiomycete IZU-154; GANSP, *Ganoderma* sp.; LACBI, *Laccaria bicolor* v2.0; LENED, *Lentinula edodes*; MALGL, *Malassezia globosa*; MELLA, *Melampsora laricis-populina* v1.0; PHACH, *Phanerochaete chrysosporium*; PHASO, *Phanerochaete sordida*; PHLBR, *Phlebia brevispora* HHB-7030 SS6 v1.0; PHLRA, *Phlebia radiata*; PHLGI, *Phlebiopsis gigantea*; PLEER, *Pleurotus eryngii*; PLEOS, *Pleurotus ostreatus*; PLEPU, *Pleurotus pulmonarius*; PLESA, *Pleurotus sapidus*; POSPL, *Postia placenta*; PUNST, *Punctularia strigosozonata* v1.0; PYCCO, *Pycnoporus coccineus* BRFM-310 v1.0; PYCCI, *Pycnoporus cinnabarinus* BRFM-137; PYCSA, *Pycnoporus sanguineus* BRFM-1264 v1.0; SCHCO, *Schizophyllum commune*; SERLA, *Serpula lacrymans*; SPORO, *Sporobolomyces roseus* v1.0; SPOSP, *Spongipellis* sp.; STEHI, *Stereum hirsutum* FP-91666 SS1 v1.0; TAICA, *Taiwanofungus camphoratus*; TRACE, *Trametopsis cervina*; TRAVE, *Trametes versicolor*; TREME, *Tremella mesenterica*; USTMA, *Ustilago maydis*; WOLCO, *Wolfiporia cocos* MD-104 SS10 v1.0. Most of the sequences included in the dendrogram were obtained from the analysis of fungal genome sequences. The genome version from which the peroxidase sequence was obtained is in some cases indicated as v1.0 and v2.0. Peroxidase abbreviations: i) APX-CCP, hybrid ascorbate-cytocrome c peroxidases; ii) CCP, cytochrome c peroxidase; iii) i) GP, generic peroxidase; iv) MnP-short, MnP-long and MnP-extralong, three different mangenese peroxidase (MnP) subfamilies differing in the length of their C-terminal tails; v) LiP, lignin peroxidase; vi) VP, versatile peroxidase; vii) VP-LiP intermediate states, two Ceriporiopsis subvermispora peroxidases occupying an intermediate position between typical LiPs and VPs according to their structural and catalytic properties (Fernández-Fueyo et al. 2012); and viii) MnP-atypical and VP-atypical, MnP and VP lacking one of the three acid residues forming the typical Mn(II)-oxidation site present in MnP and VP (Glu35/36, Glu39/40 and Asp179/175 in *P. chrysosporium* MnP/P. eryngii VP).

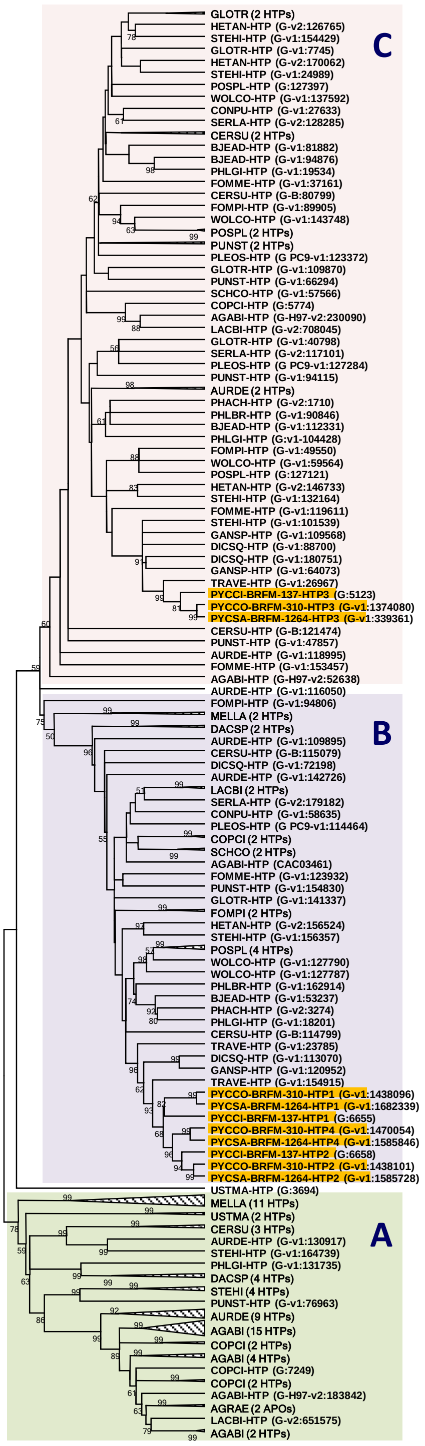

Fig. S8. Dendrogram focused on HTP peroxidases (a total of 179) showing evolutionary relationships. The analysis and fungal abbreviations are described in Figure S4. GenBank and JGI identification numbers are shown in parentheses and *Pycnoporus* strains genome identification numbers on orange background.

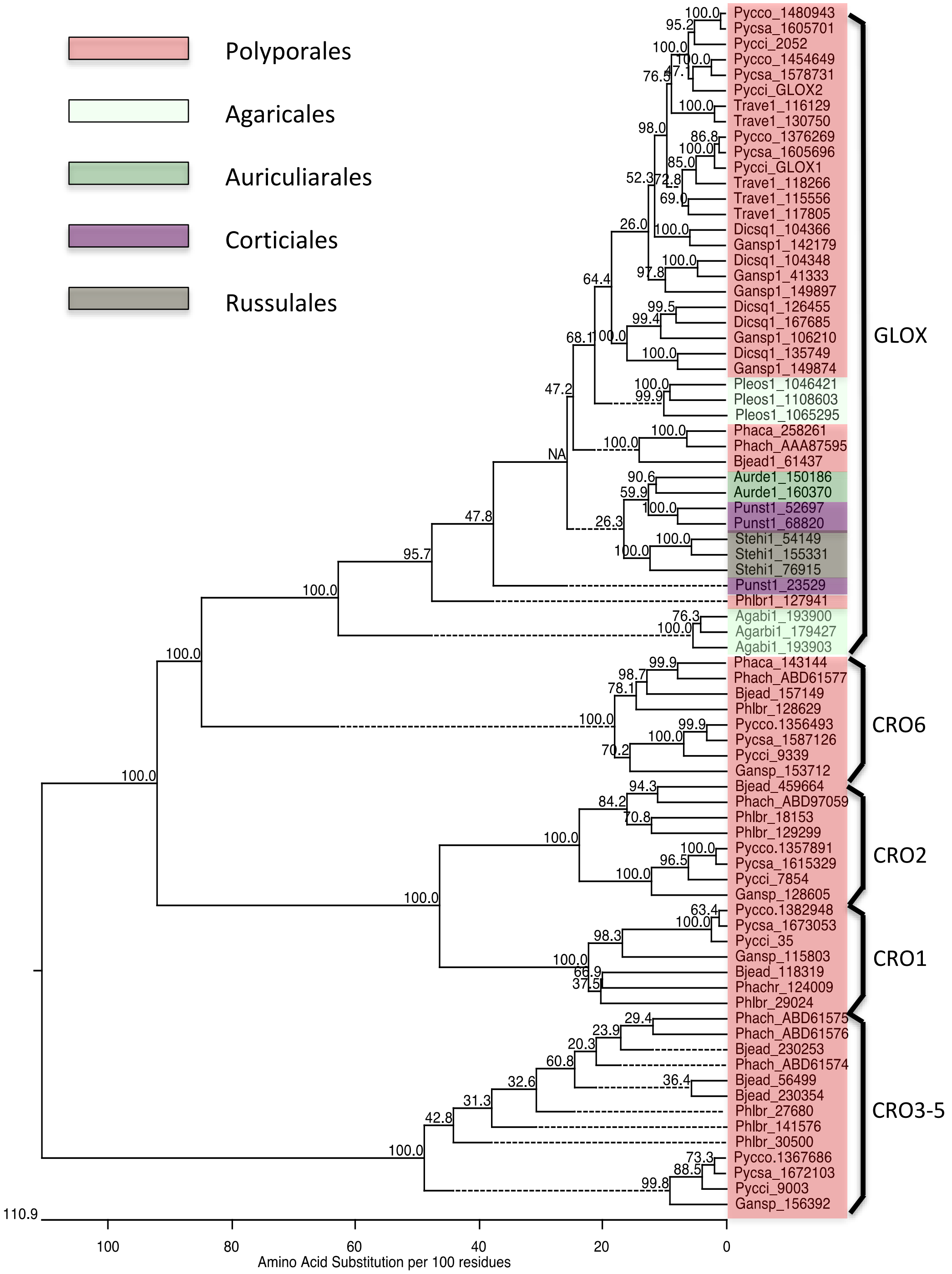

Fig. S9. Relationships between predicted CRO proteins of 15 Basidiomycete species. Bootstrap values (100 trials) are shown at nodes and the Kimura distance formula used to calculate substitutions. The JGI identification numbers are indicated. Taxonomic orders are highlighted by colored shading. Genome sequence abbreviations: Pycco, *Pycnoporus coccineus* BRFM-310 v1.0; Pycsa, *Pycnoporus sanguineus* BRFM-1264 v1.0; Pycci, *Pycnoporus cinnabarinus* BRFM-137v1.0; Trave1, *Trametes versicolor* v1.0; Dicsq1, *Dichomitus squalens* v1.0; Gansp1, *Ganoderma* sp. 10597 SS1 v1.0; Pleos1, *Pleurotus ostreatus*; Phaca, *Phanerochaete carnosa* HHB-10118-Sp v1.0; Bjead1, *Bjerkandera adusta* v1.0; Aurde1, *Auricularia delicata*; Punst1, *Punctularia strigosozonata* v1.0; Stehi1, *Stereum hirsutum* FP-91666 SS1 v1.0; Phlbr1, *Phlebia brevispora* HHB-7030 SS6 v1.0; Agabi1, *Agaricus bisporus*; Phach, *Phanerochaete chrysosporium*

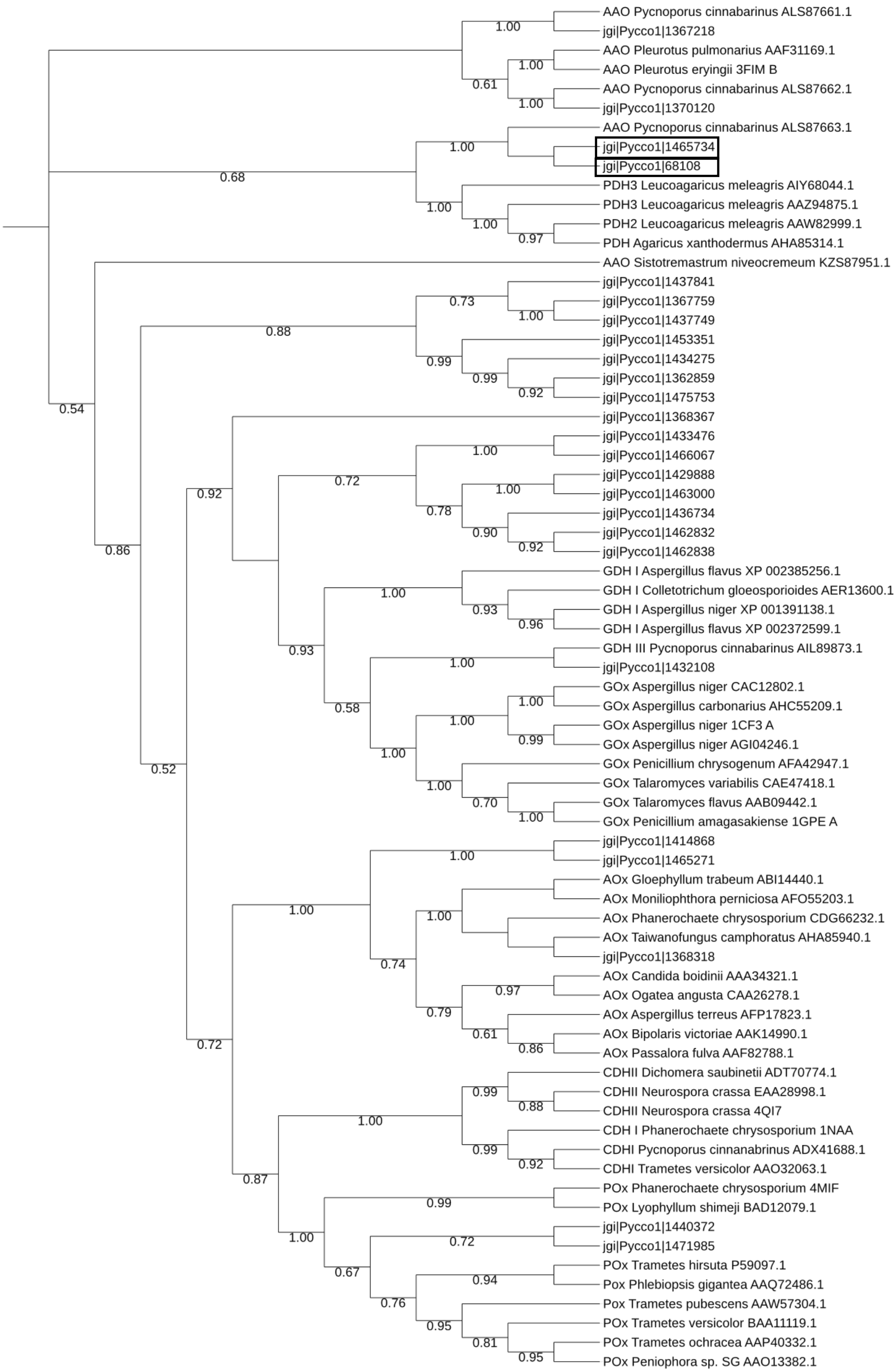

Fig. S10. Phylogenetic analysis of AA3 protein sequences from *P. coccineus* and 46 fungal AA3s with characterized activities. Protein sequences were aligned using ClustalW and the tree was obtained using maximum likelihood with 500 bootstrap values. The predicted Aryl Alcohol Oxidases secreted during growth on Avicel are indicated in boxes.

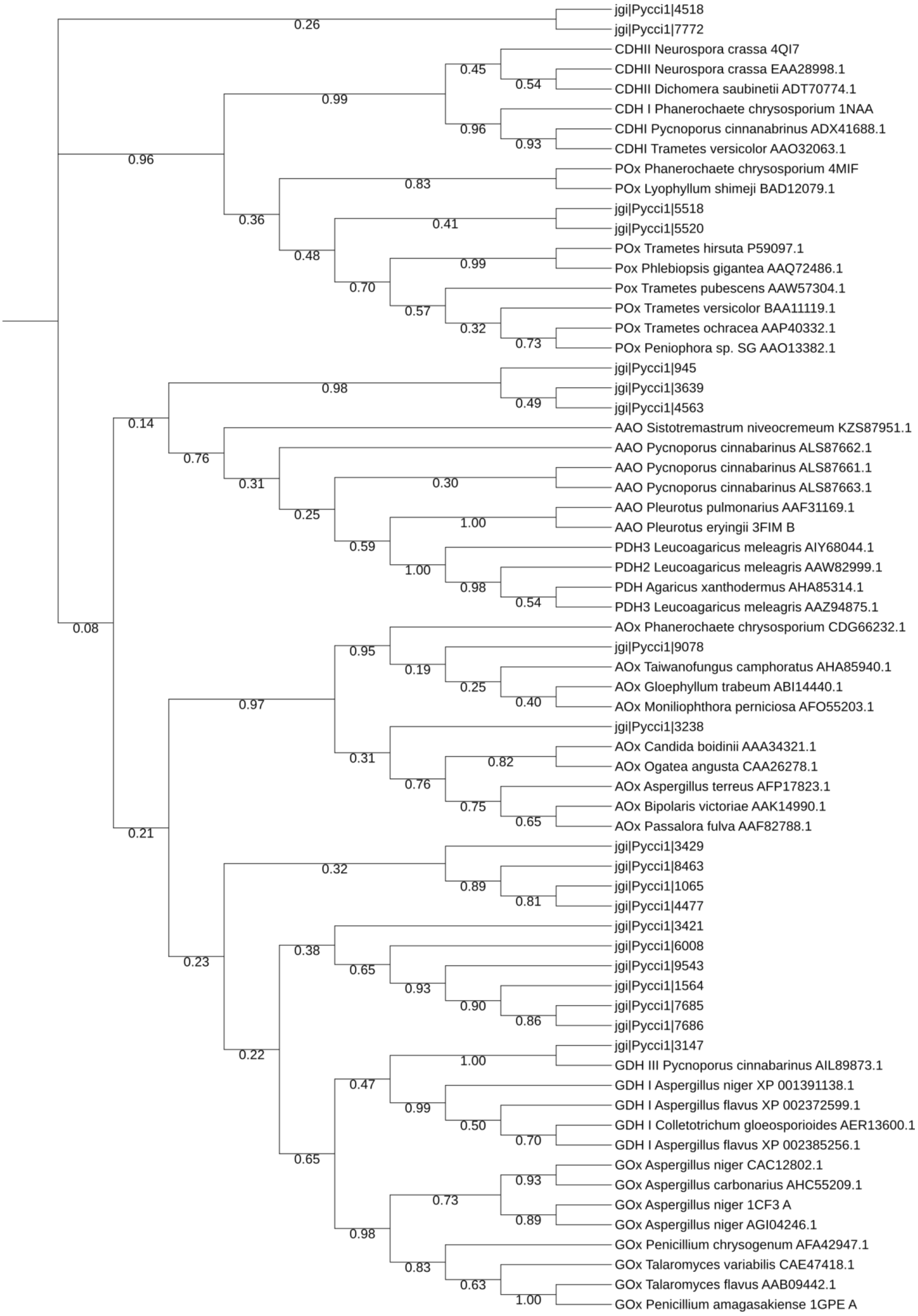

Fig. S11. Phylogenetic analysis of AA3 protein sequences from *P. cinnabarinus* BRFM 137 and 46 fungal AA3s with characterized activities. Protein sequences were aligned using ClustalW and the tree was obtained using maximum likelihood with 500 bootstrap values.

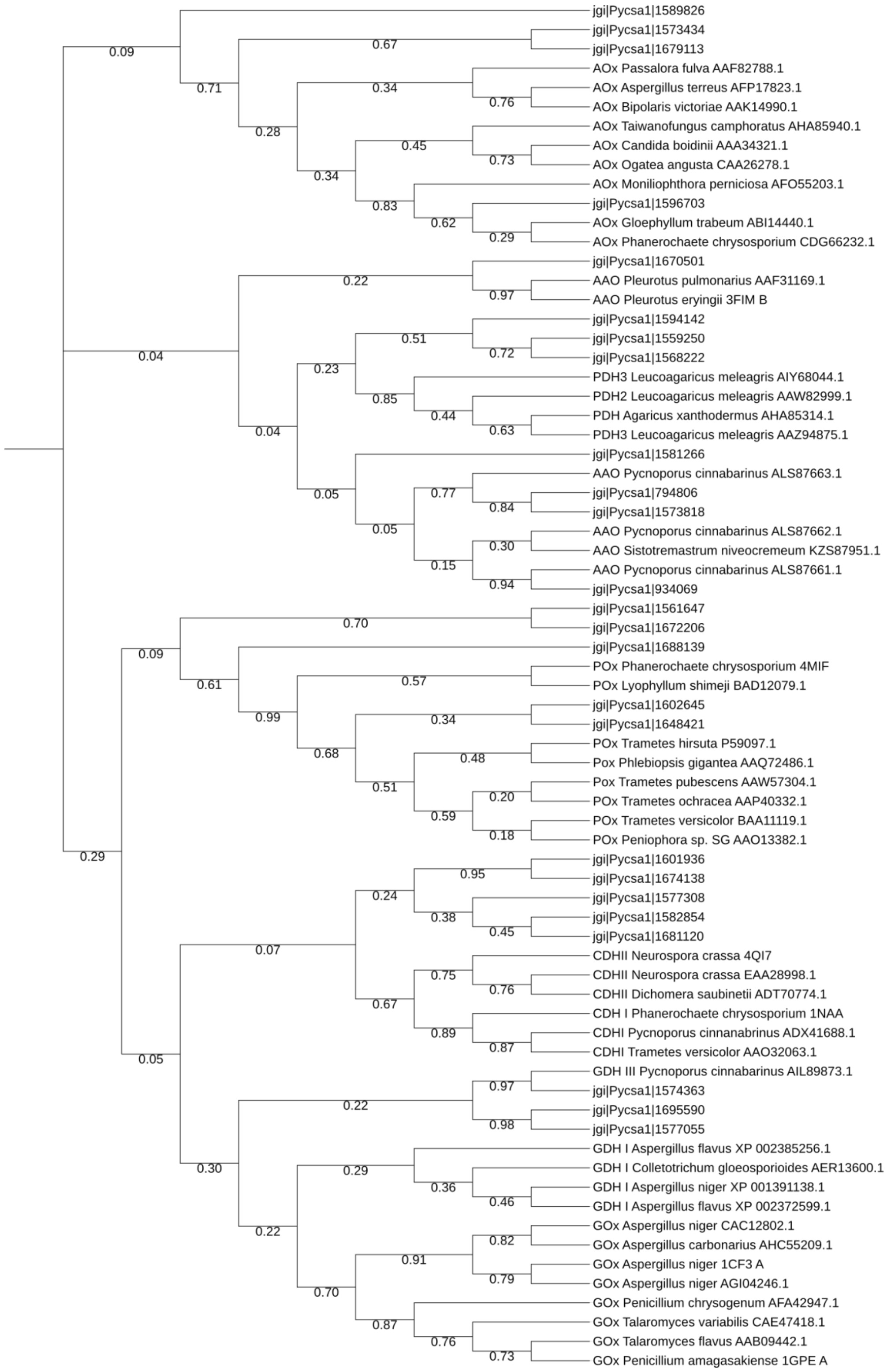

Fig. S12. Phylogenetic analysis of AA3 protein sequences from *P. sanguineus* BRFM 1264 and 46 fungal AA3s with characterized activities. Protein sequences were aligned using ClustalW and the tree was obtained using maximum likelihood with 500 bootstrap values.

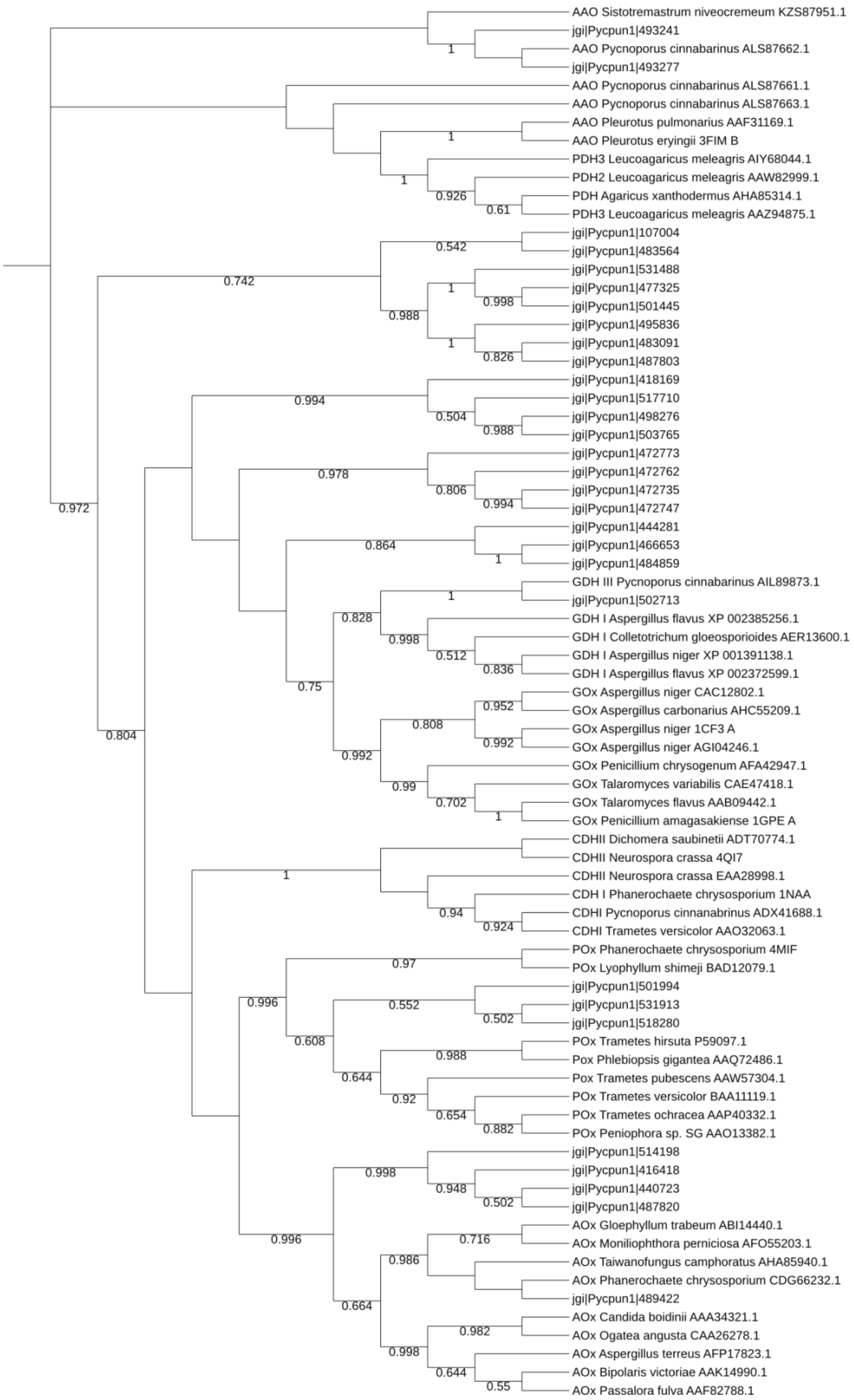

Fig. S13. Phylogenetic analysis of AA3 protein sequences from *P. puniceus* BRFM 1868 and 46 fungal AA3s with characterized activities. Protein sequences were aligned using ClustalW and the tree was obtained using maximum likelihood with 500 bootstrap values.

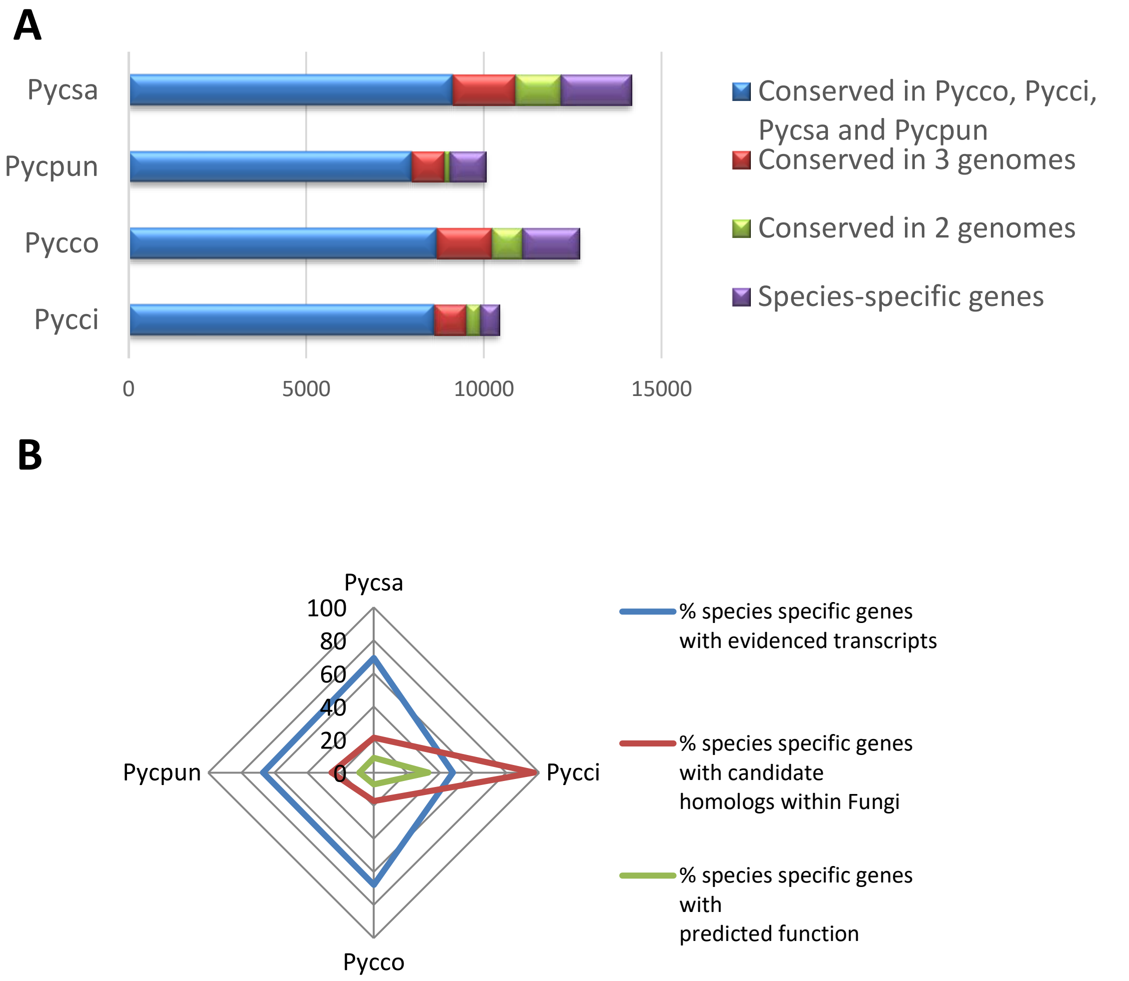

Fig. S14. Conservation of predicted protein coding genes between the four *Pycnoporus* genomes (A) and analysis of the species-specific protein coding genes (B). Evidence for transcription was obtained from EST sequences generated in six growth conditions. Predicted functions were deduced from GO, IPR, KEGG or KOG features. The presence of candidate homologs within Fungi was deduced from MCL analysis of 468 fungal genomes. Pycci: *P. cinnabarinus* BRFM 137, Pycco: *P. coccineus* BRFM 310, Pycsa: *P. sanguineus* BRFM 1264

	AA10	AA15	CBM18	CBM5	CE4	GH5_15	GH5_31	GH5_9	GH17	GH18	GH19	GH20	GH27	GH30_3	GH30_5	GH46	GH48	GH55	GH64	GH71	GH72	GH75	GH76	GH81	GH85	GH89	GH92	GH125	GH128	GH132	GH135
Trave1	0	0	1	6	3	1	1	9	3	20	0	6	4	3	0	0	0	2	0	5	1	0	0	0	1	1	4	1	4	0	2
Trapub1	0	0	0	1	3	1	1	7	3	18	0	8	3	3	0	0	0	2	0	7	1	0	0	0	1	1	4	1	4	0	2
Pycci1	0	0	1	5	3	1	0	7	2	16	0	5	2	2	0	0	0	2	0	5	1	0	2	0	1	1	5	1	4	0	2
Pycco1	0	0	1	6	3	1	0	6	2	19	0	5	4	2	0	0	0	2	0	4	1	0	1	0	1	1	5	1	4	0	1
Pycpun1	0	0	1	6	3	1	0	6	2	16	0	5	2	2	0	0	0	2	0	4	1	0	0	0	1	1	4	1	3	0	1
Pycsa1	0	0	1	6	3	1	0	6	2	20	0	5	2	2	0	0	0	2	0	4	1	0	1	0	1	1	5	1	5	0	1
Polar1	0	0	2	6	3	1	0	7	3	23	0	5	3	2	0	0	0	2	0	5	1	0	1	0	2	1	6	1	7	0	1
Polbr1	0	0	2	5	3	1	0	7	3	25	0	5	3	2	0	0	0	2	0	5	1	0	1	0	1	1	5	1	7	0	1
Gansp1	0	0	1	9	4	2	0	6	3	38	0	5	3	3	0	0	0	3	0	6	1	0	2	0	1	2	5	1	7	0	1
Dicsq1	0	0	1	5	2	1	1	6	2	21	0	4	6	2	0	0	0	2	0	4	1	0	1	0	1	1	5	1	4	0	2
Dicsqu18370_1	0	0	1	5	3	1	1	7	4	23	0	4	6	2	0	0	0	2	0	4	1	0	1	0	1	1	5	1	5	0	2
Dicsqu463_1	0	0	1	5	3	1	1	6	4	21	0	4	6	2	0	0	0	2	0	4	1	0	1	0	1	1	6	1	5	0	2
Dicsqu464_1	0	0	1	6	3	1	1	7	4	23	0	4	6	2	0	0	0	2	0	4	1	0	1	0	2	1	6	1	5	0	2
Fompi3	0	0	1	4	3	1	2	5	3	21	0	4	4	4	0	0	0	3	0	4	1	0	0	0	1	2	5	1	4	0	0
Daequ1	0	0	1	4	3	1	3	4	2	12	0	3	4	3	0	0	0	3	0	5	1	0	0	0	1	2	3	1	3	0	0
Laesu1	0	0	1	4	3	3	0	6	2	9	0	3	3	1	0	0	0	4	0	5	1	0	0	0	1	2	4	1	2	0	0
Wolco1	0	0	1	4	4	1	2	5	2	11	0	2	3	2	0	0	0	3	0	1	2	0	0	0	1	1	3	1	2	0	0
Pospl1	0	0	2	5	3	2	2	12	4	19	0	6	3	6	0	0	0	6	0	5	4	0	0	0	2	2	6	1	6	0	0
Posplrsb12_1	0	0	1	5	2	1	1	4	2	11	0	3	3	3	0	0	0	3	0	2	2	0	0	0	1	1	3	1	5	0	0
Fibra1	0	0	3	4	3	1	1	2	1	9	0	2	3	1	0	0	0	3	0	1	1	0	0	0	1	1	3	1	2	0	0
Cersu1	0	0	1	3	3	1	1	6	3	14	0	3	4	1	0	0	0	2	0	1	1	0	0	0	1	2	3	1	3	0	2
Obbri1	0	0	1	4	3	1	1	5	2	14	0	4	4	2	0	0	0	2	0	1	1	0	0	0	1	2	4	1	3	0	1
Phaca1	0	0	0	3	2	1	1	6	2	11	0	3	3	1	0	0	0	2	0	2	1	0	0	0	1	1	4	1	4	0	1
Phchr2	0	0	1	4	4	1	1	6	2	11	0	4	3	1	0	0	0	2	0	3	1	0	0	0	1	2	4	1	4	0	1
Phlgi1	0	0	1	3	3	1	2	5	2	12	0	4	3	2	0	0	0	2	0	4	1	0	0	0	1	2	4	1	6	0	1
Bjead1_1	0	0	1	4	3	1	1	5	3	16	0	4	3	1	0	0	0	3	0	3	1	0	0	0	1	1	3	1	5	0	2
Phlcen1	0	0	0	1	3	2	1	5	2	12	0	4	2	1	0	0	0	5	0	2	2	0	3	0	1	1	4	1	6	0	0
Phlbr1	0	0	5	6	2	2	2	6	2	22	0	6	2	2	0	0	0	4	0	4	1	0	0	0	3	3	4	1	3	0	2

Fig. S15. Gene counts for CAZyme domains active on fungal cell walls in Polyporales genomes. Bjead1_1: *Bjerkandera adusta*, Cersu1: *Ceriporiopsis (Gelatoporia) subvermispora* B, Daequ1: *Daedalea quercina*, Dicsq1: *Dichomitus squalens* v1, Dicsqu18370_1: *Dichomitus squalens* OM18370.1 v1.0 , Dicsqu463_1: *Dichomitus squalens* CBS463.89 v1.0 , Dicsqu464_1: *Dichomitus squalens* CBS464.89 v1.0 , Fibra1: *Fibroporia radiculosa* TFFH 294, Fompi3: *Fomitopsis pinicola* FP-58527 SS1 v3.0, Gansp1: *Ganoderma* sp. 10597 SS1, Laesu1: *Laetiporus sulphureus* var. *sulphureus*, Obbri1: *Obba rivulosa* 3A-2 v1, Phaca1: *Phanerochaete carnosa* HHB-10118-sp, Phchr2: *Phanerochaete chrysosporium* RP-78 v2.2, Phlbr1: *Phlebia brevispora* HHB-7030 SS6, Phlcen1: *Phlebia centrifuga*, Phlgi1: *Phlebiopsis gigantea*, Polar1: *Polyporus arcularius*, Polbr1: *Polyporus brumalis* BRFM 1820 v1.0, Pospl1: *Postia placenta* Mad-698-R, PosplRSB12_1: *Postia placenta* MAD-698-R-SB12, Pycci1: *Pycnoporus cinnabarinus* BRFM137, Pycco1: *Pycnoporus coccineus* BRFM 310 v1, Pycpun1: *Pycnoporus puniceus* CIRM-BRFM 1868 v1.0, Pycsa1: *Pycnoporus sanguineus* BRFM1264 v1, Trapub1: *Trametes pubescens*, Trave1: *Trametes versicolor* FP-101664 SS1, Wolco1: *Wolfiporia cocos* MD-104 SS10.

AA8	AA8.AA3_1	AA8.CBM1	AA9	AA10	AA15	AA16	CBM1	CBM63	GH1	GH5_1	GH5_4	GH5_5	GH5_22	GH6	GH7	GH9	GH12	GH44	GH45	GH48	GH131	
0	1	1	18	0	0	0	22	0	2	0	0	3	2	1	4	1	5	0	2	0	3	Trave1
0	1	1	17	0	0	0	17	0	3	0	0	4	2	1	4	1	4	0	3	0	2	Trapub1
0	1	1	17	0	0	0	19	0	2	0	0	3	2	1	3	1	3	0	2	0	3	Pycci1
0	1	1	16	0	0	0	21	0	2	0	0	3	2	1	3	1	3	0	3	0	3	Pycco1
0	1	1	13	0	0	0	19	0	2	0	0	3	2	1	3	1	3	0	3	0	3	Pycpun1
0	1	1	16	0	0	0	20	0	2	0	0	4	2	1	3	1	3	0	2	0	3	Pycsa1
0	1	1	19	0	0	0	21	0	2	0	0	3	2	1	3	1	3	1	3	0	3	Polar1
0	1	1	17	0	0	0	19	0	2	0	0	3	2	1	3	1	3	1	3	0	3	Polbr1
0	1	1	16	0	0	0	17	0	3	0	0	3	2	1	3	1	3	0	2	0	3	Gansp1
0	1	1	15	0	0	0	16	0	4	0	0	3	2	1	4	0	3	1	1	0	3	Dicsq1
0	1	1	16	0	0	0	17	0	3	0	0	3	2	1	4	1	3	1	3	0	3	Dicsqu18370_1
0	1	1	16	0	0	0	16	0	3	0	0	3	2	1	4	1	3	1	3	0	3	Dicsqu463_1
0	1	1	16	0	0	0	17	0	3	0	0	3	2	1	4	1	3	1	3	0	3	Dicsqu464_1
0	0	0	4	0	0	0	0	0	2	0	0	3	2	0	0	0	2	0	2	0	1	Fompi3
0	0	0	4	0	0	0	0	0	2	0	0	2	2	0	0	0	2	0	1	0	1	Daequ1
0	0	0	2	0	0	0	0	0	2	0	0	2	2	0	2	0	2	0	2	0	0	Laesu1
0	0	0	2	0	0	0	0	0	1	0	0	2	2	0	0	0	2	0	0	0	0	Wolco1
0	0	0	4	0	0	0	0	0	4	0	0	5	3	0	0	0	4	0	2	0	0	Pospl1
0	0	0	2	0	0	0	0	0	2	0	0	3	2	0	0	0	2	0	1	0	0	Posplrsb12_1
0	0	0	2	0	0	0	1	0	2	0	0	3	2	0	0	0	2	0	1	0	0	Fibra1
0	1	1	9	0	0	0	16	0	3	0	0	2	2	1	3	0	2	0	2	0	1	Cersu1
0	1	1	8	0	0	0	16	0	3	0	0	2	2	1	2	1	2	0	3	0	2	Obbri1
0	1	1	11	0	0	0	26	0	2	0	0	6	2	1	5	1	3	0	1	0	2	Phaca1
0	1	1	16	0	0	0	35	0	2	0	0	2	2	1	8	1	2	0	2	0	3	Phchr2
0	1	1	15	0	0	0	24	0	2	0	0	4	2	1	5	1	3	0	1	0	2	Phlgi1
0	1	1	28	0	0	1	31	0	2	0	0	4	2	1	5	1	2	0	1	0	3	Bjead1_1
1	1	0	11	0	0	0	12	0	2	0	0	2	2	1	3	1	2	1	1	0	2	Phlcen1
0	1	1	12	0	0	0	27	0	2	0	0	4	2	1	4	1	2	2	3	0	4	Phlbr1

Fig. S16. Gene counts for CAZyme domains active on cellulose and β-1,4-glucans in Polyporales genomes. The fungal genomes are listed in Fig. S15.

	AA14	CBM13	CBM35	CE1	CE2	CE3	CE4	CE6	CE15	CE16	GH2	GH5_1	GH5_7	GH5_22	GH8	GH10	GH11	GH29	GH30_1	GH30_7	GH36	GH39	GH43	GH51	GH52	GH54	GH62	GH67	GH74	GH93	GH95	GH113	GH115	GH120	GH134	GH141
Trave1	4	5	1	3	0	0	3	0	2	6	5	0	2	2	0	6	0	0	0	0	0	0	0	2	0	0	0	0	1	0	1	0	2	0	0	0
Trapub1	4	6	1	3	0	0	3	0	2	6	5	0	2	2	0	6	0	0	0	0	0	0	0	2	0	0	0	0	1	0	1	0	2	0	0	0
Pycci1	4	4	1	3	0	0	3	0	2	4	3	0	2	2	0	6	0	0	0	0	0	0	0	2	0	0	0	0	1	0	1	0	2	0	0	0
Pycco1	4	5	1	3	0	0	3	0	2	7	2	0	4	2	0	6	0	0	0	0	0	0	0	2	0	0	0	0	1	0	1	0	2	0	0	0
Pycpun1	4	5	1	3	0	0	3	0	2	5	3	0	2	2	0	7	0	0	0	0	0	0	0	2	0	0	0	0	1	0	1	0	2	0	0	0
Pycsa1	4	6	1	3	0	0	3	0	2	6	4	0	3	2	0	6	0	0	0	0	0	0	0	2	0	0	0	0	1	0	1	0	2	0	0	0
Polar1	4	3	1	3	0	0	3	0	2	7	4	0	2	2	0	6	0	0	0	0	0	0	0	2	0	0	0	0	1	1	1	0	1	0	0	0
Polbr1	4	7	1	4	0	0	3	0	2	7	4	0	2	2	0	6	0	0	0	0	0	0	0	2	0	0	0	0	1	1	1	0	1	0	0	0
Gansp1	4	8	1	2	0	0	4	0	2	9	3	0	2	2	0	10	0	0	0	0	0	0	0	2	0	0	0	0	1	2	2	0	3	0	0	0
Dicsq1	4	11	1	0	0	0	2	0	2	7	4	0	2	2	0	5	0	0	0	0	0	0	0	2	0	0	0	0	1	1	1	0	2	0	0	0
Dicsqu18370_1	4	16	1	1	0	0	3	0	2	7	4	0	2	2	0	5	0	0	0	0	0	0	0	2	0	0	0	0	1	1	1	0	2	0	0	0
Dicsqu463_1	4	15	1	1	0	0	3	0	2	7	4	0	2	2	0	5	0	0	0	0	0	0	0	3	0	0	0	0	1	1	1	0	2	0	0	0
Dicsqu464_1	4	14	1	1	0	0	3	0	2	7	4	0	2	2	0	5	0	0	0	0	0	0	0	2	0	0	0	0	1	1	1	0	2	0	0	0
Fompi3	3	11	1	0	0	0	3	0	1	7	4	0	2	2	0	3	0	0	0	0	0	0	0	3	0	0	0	0	0	0	1	0	1	0	0	0
Daequ1	2	1	1	1	0	0	3	0	1	9	3	0	2	2	0	4	0	0	0	0	0	0	1	1	0	0	0	0	0	0	1	0	1	0	0	0
Laesu1	3	5	1	2	0	0	3	0	0	6	4	0	2	2	0	3	0	0	0	0	0	0	0	2	0	0	0	0	0	0	1	0	3	0	0	0
Wolco1	2	3	1	0	0	0	4	0	1	6	3	0	2	2	0	4	0	0	0	0	0	0	0	4	0	0	0	0	0	0	1	0	2	0	0	0
Pospl1	3	20	1	0	0	0	3	0	2	10	6	0	3	3	0	3	0	0	0	0	0	0	0	3	0	0	0	0	0	0	1	0	1	0	0	0
Posplrsb12_1	2	15	1	0	0	0	2	0	1	4	4	0	2	2	0	3	0	0	0	0	0	0	0	1	0	0	0	0	0	0	1	0	1	0	0	0
Fibra1	2	0	1	0	0	0	3	0	0	4	3	0	2	2	0	3	0	0	0	0	0	0	0	3	0	0	0	0	0	0	1	0	2	0	0	0
Cersu1	2	6	0	2	0	0	3	0	2	5	4	0	2	2	0	6	1	0	0	0	0	0	0	2	0	0	0	0	1	0	1	0	2	0	0	0
Obbri1	2	2	0	2	0	0	3	0	2	8	3	0	2	2	0	6	0	0	0	0	0	0	0	2	0	0	0	0	1	0	1	0	2	0	0	0
Phaca1	2	0	1	2	0	0	2	0	3	5	2	0	2	2	0	5	1	0	0	0	0	0	1	2	0	0	0	0	2	0	1	0	1	0	0	0
Phchr2	2	8	1	4	0	0	4	0	2	6	2	0	3	2	0	6	1	0	0	0	0	0	0	2	0	0	0	0	4	0	1	0	1	0	0	0
Phlgi1	3	7	1	1	0	0	3	0	1	8	3	0	2	2	0	4	3	0	0	0	0	0	1	2	0	0	0	0	2	0	0	0	1	0	0	0
Bjead1_1	3	13	1	1	0	0	3	0	2	15	3	0	2	2	0	4	0	0	0	0	0	0	0	2	0	0	0	0	2	0	1	0	2	0	0	0
Phlcen1	1	2	2	1	0	0	3	0	6	4	3	0	3	2	0	6	0	1	0	0	0	0	0	4	0	0	0	0	1	0	1	0	2	0	0	0
Phlbr1	2	30	1	1	0	0	2	0	2	5	2	0	2	2	0	8	0	1	0	0	0	0	0	1	0	0	0	0	1	0	3	0	2	0	0	0

Fig. S17. Gene counts for CAZyme domains active on hemicellulose in Polyporales genomes. The fungal genomes are listed in Fig. S15.

	CBM35	CBM67	CE8	CE12	GH28	GH39	GH51	GH53	GH78	GH88	GH105	GH106	PL1	PL3	PL4	PL8_4	PL9	PL11	PL14_4
Trave1	1	0	2	0	11	0	2	1	3	1	0	0	0	0	0	2	0	0	4
Trapub1	1	0	3	1	9	0	2	1	2	1	0	0	0	0	0	2	0	0	5
Pycci1	1	0	2	0	7	0	2	0	2	2	0	0	0	0	1	2	0	0	3
Pycco1	1	0	2	0	7	0	2	1	2	1	0	0	0	0	0	2	0	0	4
Pycpun1	1	0	1	0	7	0	2	1	2	1	0	0	0	0	0	2	0	0	2
Pycsa1	1	0	2	0	8	0	2	1	2	1	0	0	0	0	0	2	0	0	4
Polar1	1	0	4	1	11	0	2	1	2	1	2	0	0	0	2	3	0	0	4
Polbr1	1	0	4	1	11	0	2	1	2	1	2	0	0	0	0	3	0	0	4
Gansp1	1	0	3	1	10	0	2	1	4	1	1	0	0	0	0	3	0	0	4
Dicsq1	1	0	3	2	7	0	2	1	5	1	1	0	0	0	0	3	0	0	5
Dicsqu18370_1	1	0	3	2	7	0	2	1	3	1	1	0	0	0	0	3	0	0	5
Dicsqu463_1	1	0	3	2	7	0	3	1	4	1	1	0	0	0	0	3	0	0	5
Dicsqu464_1	1	0	3	2	7	0	2	1	4	1	1	0	0	0	0	3	0	0	5
Fompi3	1	0	2	0	12	0	3	1	4	1	1	0	0	0	0	0	0	0	0
Daequ1	1	0	1	0	9	0	1	1	3	1	0	0	0	0	0	0	0	0	0
Laesu1	1	0	1	0	8	0	2	1	3	1	0	0	0	0	0	0	0	0	1
Wolco1	1	0	1	0	9	0	4	1	3	1	0	0	0	0	0	0	0	0	0
Pospl1	1	0	4	0	12	0	3	2	4	2	0	0	0	0	0	0	0	0	3
Posplrsb12_1	1	0	2	0	8	0	1	1	3	1	0	0	0	0	0	0	0	0	1
Fibra1	1	0	2	0	9	0	3	1	3	1	0	0	0	0	0	0	0	0	0
Cersu1	0	0	2	0	6	0	2	6	1	1	0	0	0	0	0	2	0	0	2
Obbri1	0	0	2	0	5	0	2	1	1	1	0	0	0	0	0	2	0	0	2
Phaca1	1	0	2	0	4	0	2	1	1	1	0	0	0	0	0	1	0	0	3
Phchr2	1	0	2	0	5	0	2	1	1	1	0	0	0	0	0	1	0	0	3
Phlgi1	1	0	4	1	10	0	2	1	1	1	0	0	0	0	0	1	0	0	3
Bjead1_1	1	0	2	1	6	0	2	1	2	1	1	0	0	0	0	1	0	0	3
Phlcen1	2	0	1	0	5	0	4	1	2	1	2	0	0	0	0	1	0	0	3
Phlbr1	1	0	3	0	5	0	1	1	1	1	0	0	0	0	0	1	0	0	3

Fig. S18. Gene counts for CAZyme domains active on pectin in Polyporales genomes. The fungal genomes are listed in Fig. S15.

Genome	AA1	AA1_1	AA1_2	AA1_3	AA2	AA2_cyt	AA3	AA3_1	AA3_2	AA3_3	AA3_4	AA4	AA5	AA5_1	AA5_2	AA6	AA7	AA8	AA9	AA10	AA11	AA12	AA13	AA14	AA15	AA16
Trave1	1	7	2	0	26	0	0	0	17	4	1	0	0	9	0	1	0	1	18	0	0	0	0	4	0	0
Trapub1	1	6	1	0	23	0	0	0	30	4	2	0	0	7	0	1	0	1	17	0	0	0	0	4	0	0
Pycci1	1	5	1	0	11	0	0	0	20	2	2	0	0	7	0	1	0	1	17	0	0	0	0	4	0	0
Pycco1	1	5	1	0	11	0	0	0	20	3	2	0	0	7	0	1	0	1	16	0	0	0	0	4	0	0
Pycpun1	1	4	1	0	8	0	0	0	22	5	3	0	0	6	0	1	0	1	13	0	0	0	0	4	0	0
Pycsa1	1	5	1	0	11	0	0	0	20	3	2	0	0	7	0	1	0	1	16	0	0	0	0	4	0	0
Polar1	1	9	1	0	20	0	1	0	30	3	1	0	0	8	0	4	1	1	19	0	0	0	0	4	0	0
Polbr1	1	9	1	0	19	0	2	0	30	3	1	0	0	8	0	3	1	1	17	0	0	0	0	4	0	0
Gansp1	1	16	1	0	9	0	0	0	27	5	0	0	0	9	0	2	4	1	16	0	0	0	0	4	0	0
Dicsq1	1	11	1	0	12	0	0	0	27	4	0	0	0	9	0	1	4	1	15	0	0	0	0	4	0	0
Dicsqu18370_1	2	11	1	0	13	0	0	0	38	4	0	0	0	10	0	1	4	1	16	0	0	0	0	4	0	0
Dicsqu463_1	2	11	1	0	12	0	2	0	30	4	0	0	0	9	0	1	4	1	16	0	0	0	0	4	0	0
Dicsqu464_1	2	12	1	0	12	0	2	0	31	4	0	0	0	9	0	1	4	1	16	0	0	0	0	4	0	0
Fompi3	1	5	1	0	1	0	0	0	16	5	0	0	0	4	0	1	4	0	4	0	0	0	0	3	0	0
Daequ1	2	3	1	0	1	0	0	0	13	4	0	0	0	4	0	1	3	0	4	0	0	0	0	2	0	0
Laesu1	3	3	1	0	1	0	0	0	26	4	0	0	0	5	0	1	0	0	2	0	0	0	0	3	0	0
Wolco1	1	3	2	0	1	0	0	0	8	6	0	0	0	4	0	1	0	0	2	0	0	0	0	2	0	0
Pospl1	1	5	2	0	2	0	2	0	36	8	0	0	0	5	0	2	0	0	4	0	0	0	0	3	0	0
Posplrsb12_1	1	2	1	0	1	0	0	0	24	5	0	0	0	3	0	1	0	0	2	0	0	0	0	2	0	0
Fibra1	1	2	1	0	1	0	0	0	16	6	0	0	0	3	0	1	0	0	2	0	0	0	0	2	0	0
Cersu1	1	7	1	0	17	0	0	0	17	4	0	0	0	3	0	0	0	1	9	0	0	0	0	2	0	0
Obbri1	1	8	1	0	15	0	0	0	19	5	0	0	0	4	0	1	0	1	8	0	0	0	0	2	0	0
Phaca1	9	0	1	0	11	0	0	0	32	4	0	0	0	6	0	3	0	1	11	0	0	3	0	2	0	0
Phchr2	4	0	1	0	16	0	0	0	34	3	1	0	0	7	0	4	0	1	16	0	0	1	0	2	0	0
Phlgi1	4	0	1	0	9	0	0	0	19	3	1	0	0	6	0	4	0	1	15	0	0	2	0	3	0	0
Bjead1_1	1	0	1	0	20	0	0	0	30	8	1	0	0	7	0	4	0	1	28	0	0	1	0	3	0	1
Phlcen1	1	4	1	0	8	0	1	0	16	5	1	0	0	6	0	3	0	1	11	0	0	1	0	1	0	0
Phlbr1	1	8	2	0	15	0	0	0	32	6	1	0	0	8	0	4	0	1	12	0	0	1	0	2	0	0

Fig. S19. Gene counts for CAZyme domains of Auxiliary Activity enzymes. The fungal genomes are listed in Fig. S15.

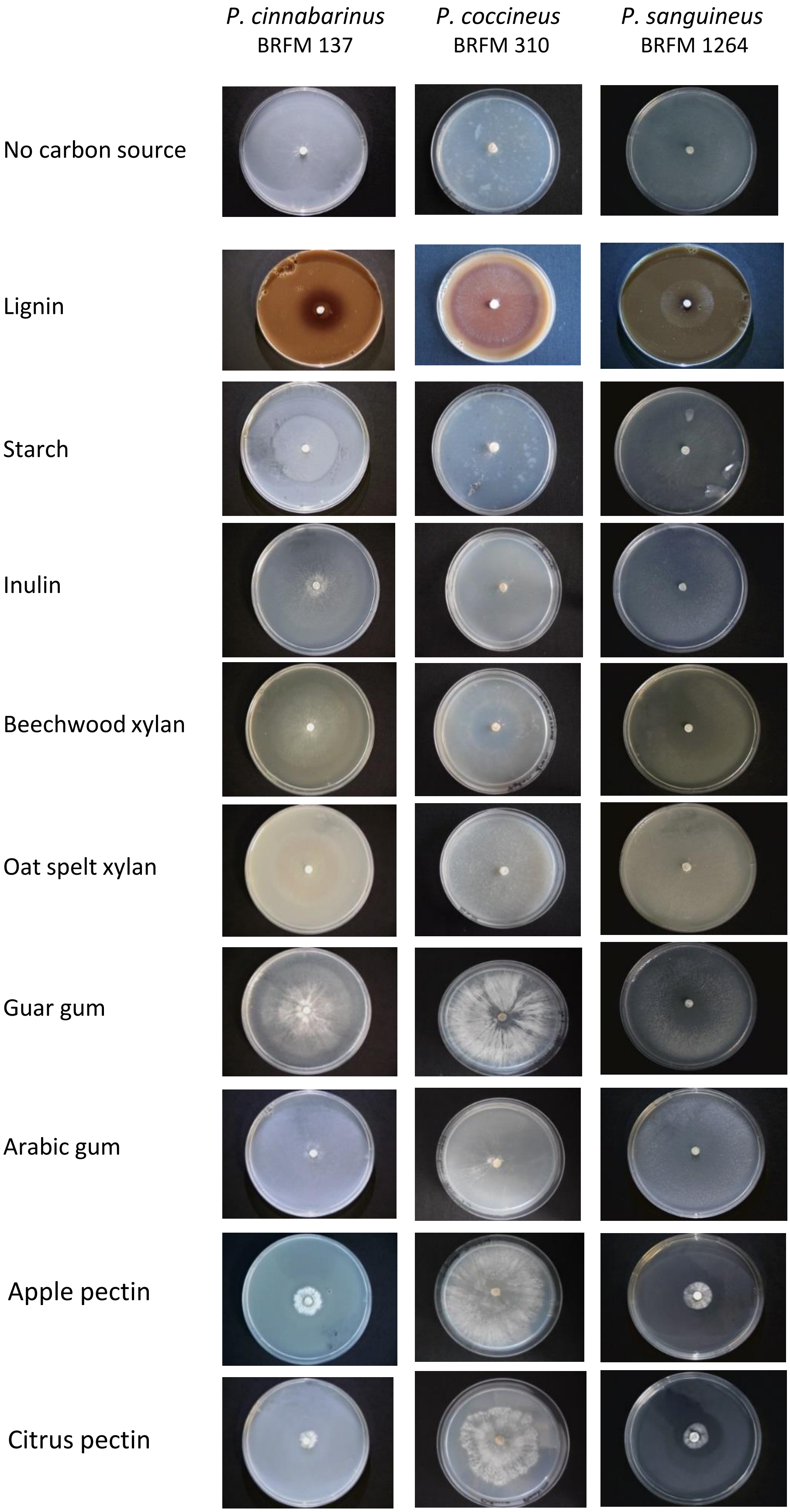

P. cinnabarinus
BRFM 137
P. coccineus
BRFM 310
P. sanguineus
BRFM 1264
No carbon source
Lignin
Starch
Inulin
Beechwood xylan
Oat spelt xylan
Guar gum
Arabic gum
Apple pectin
Citrus pectin

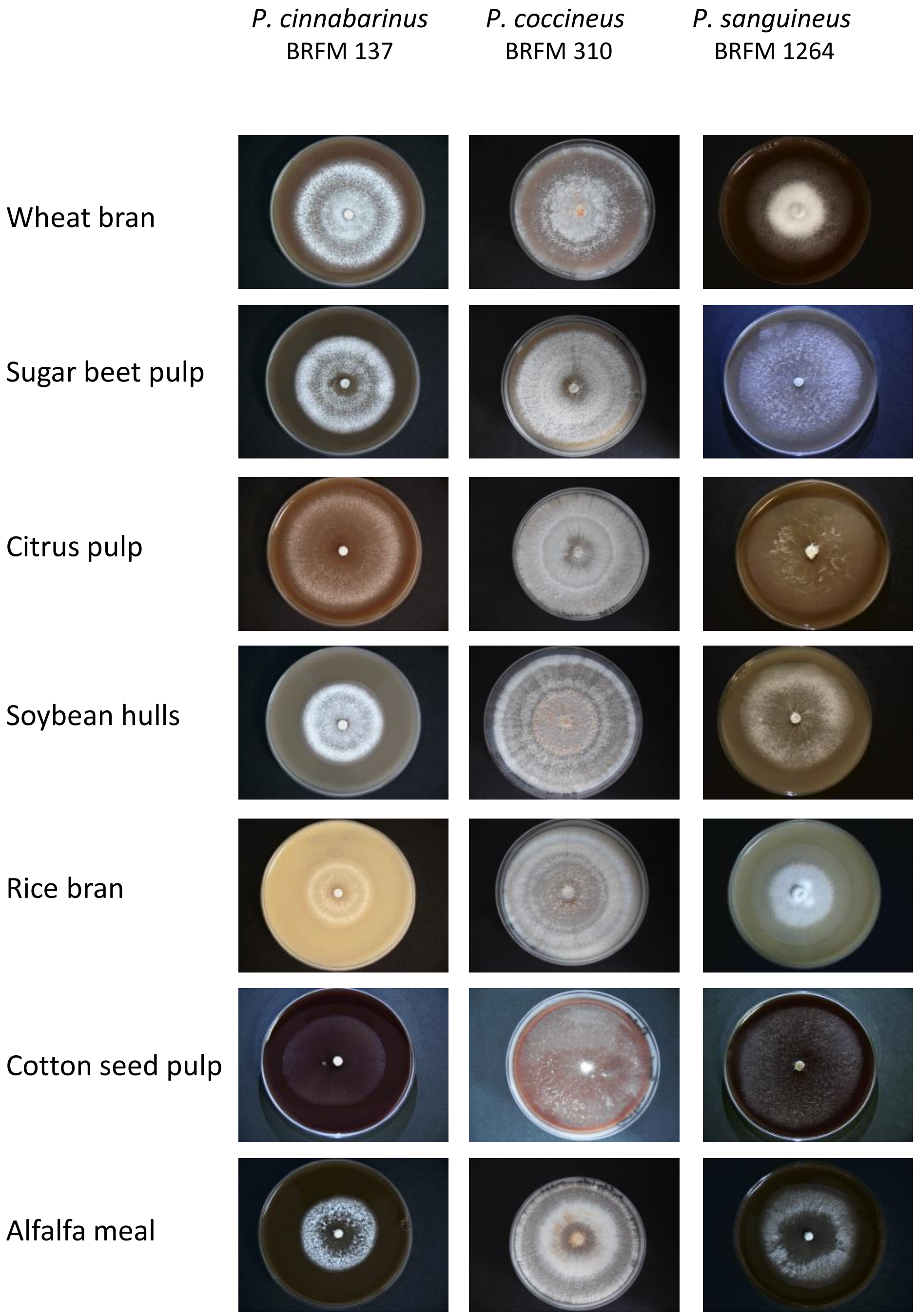

Fig. S20. Growth of the three *Pycnoporus* strains on 17 different carbon sources. The Petri dishes were inoculated with one fungal disk (4 mm diameter) of 7-day-old mycelia and incubated 7 days at 30°C.

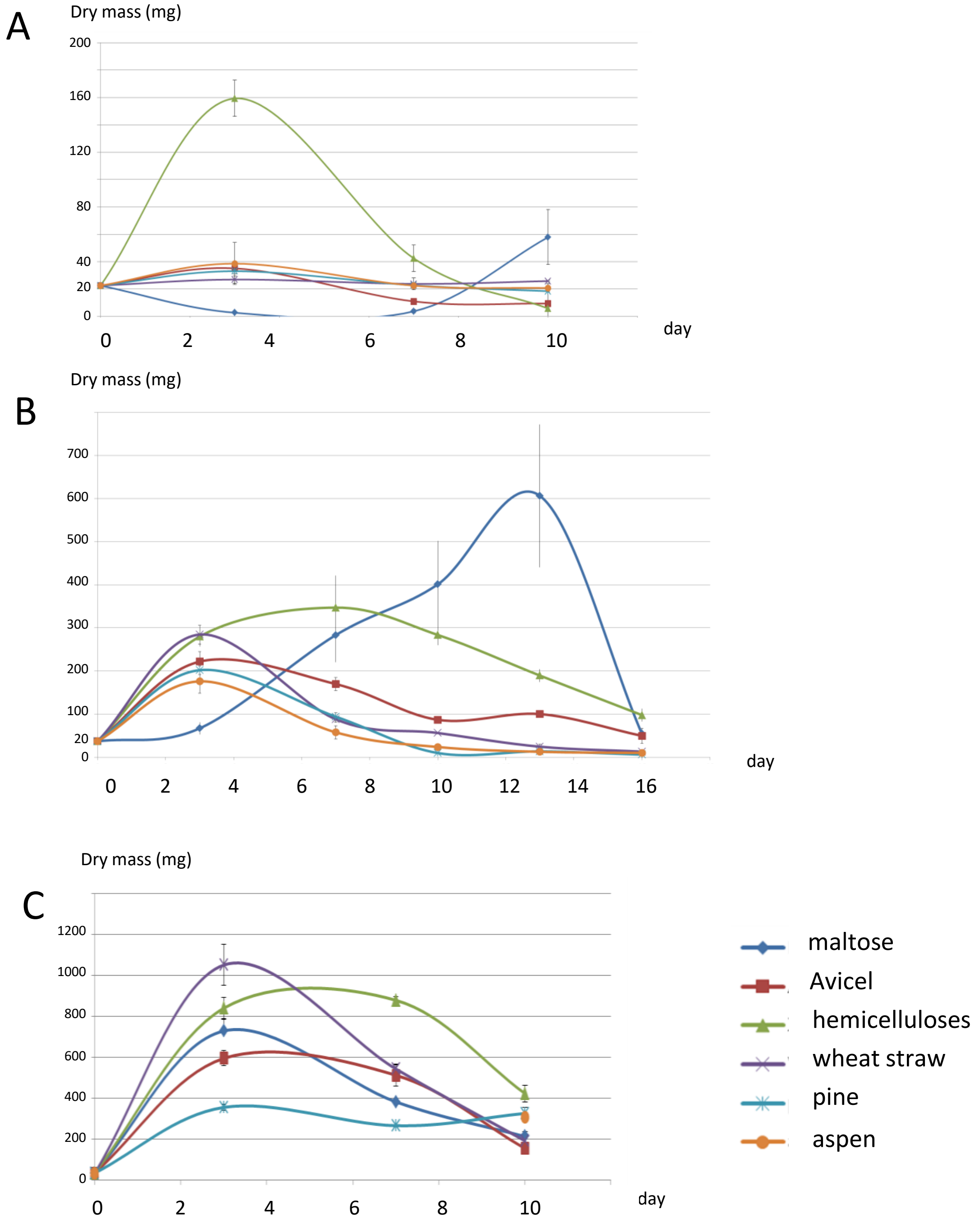

Fig S21. Growth of *P. cinnabarinus* BRFM 137 (A), *P. coccineus* BRFM 310 (B) and *P. sanguineus* BRFM 1264 (C) on six different carbon sources in liquid cultures as determined by qPCR. Day 3 was selected for total RNA extraction and comparative transcriptomics.

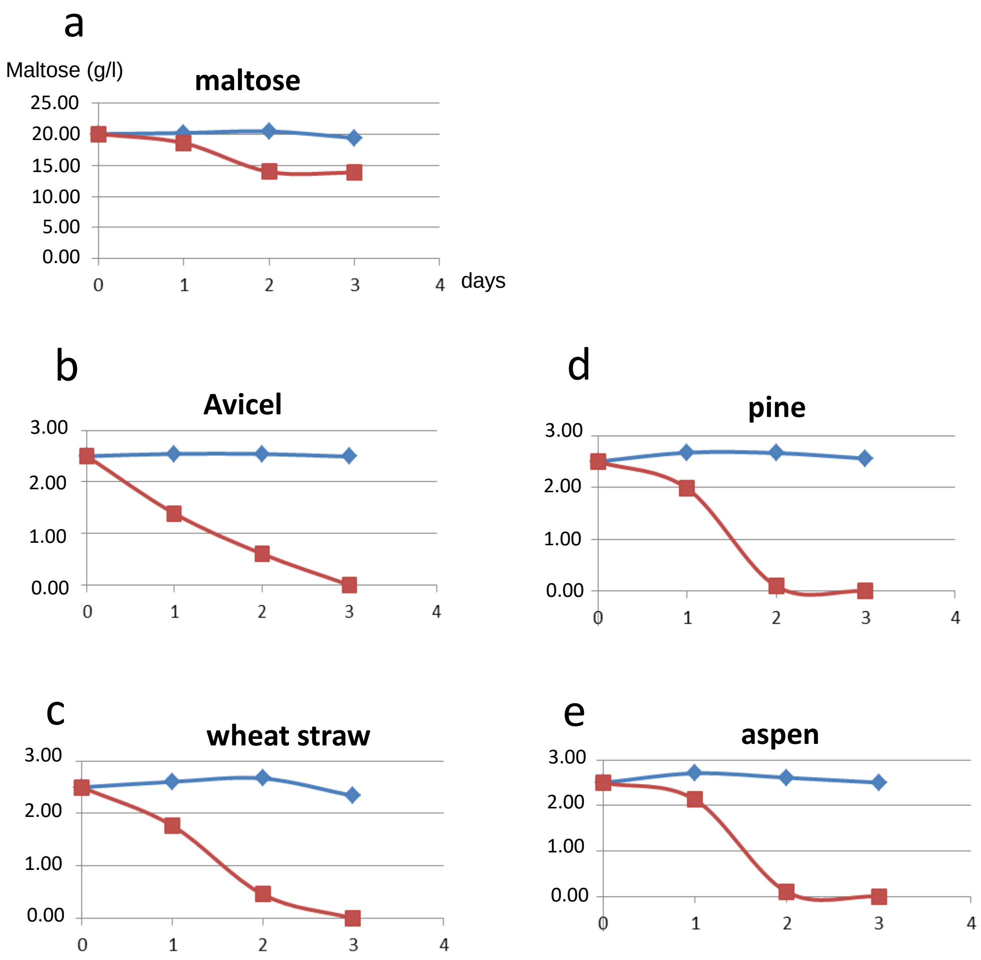

Fig. S22. Maltose concentration in the culture medium during growth of *Pycnoporus coccineus* BRFM 310 on maltose 20 g.l^{-1} (a), or on 2.5 g.l^{-1} maltose additioned with 15 g.l^{-1} Avicel, (b) wheat straw (c), pine (d), or aspen (e). Maltose concentrations in inoculated (red) and non inoculated (blue) medium are indicated.

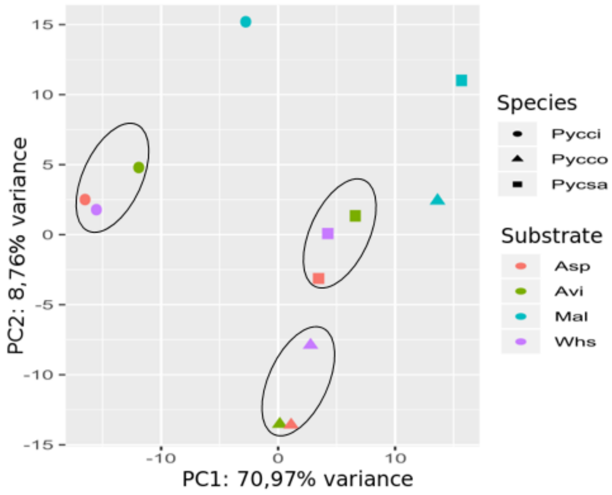

Fig. S23. Comparison of the transcript levels for CAZyme coding genes between the three strains grown on four different substrates by Principal Component Analysis. Pycci: *P. cinnabarinus* BRFM 137, Pycco: *P. coccineus* BRFM 310, Pycsa: *P. sanguineus* BRFM 1264, Asp: aspen, Avi: avicel, Mal: maltose, Whs: wheat straw.

a

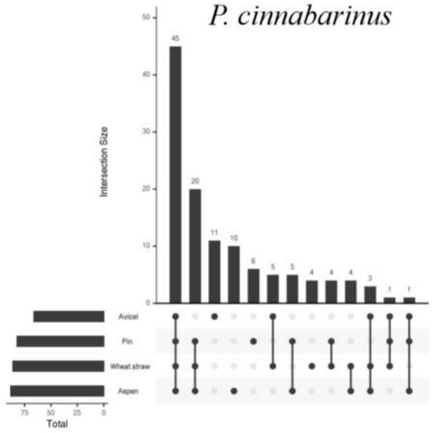

b

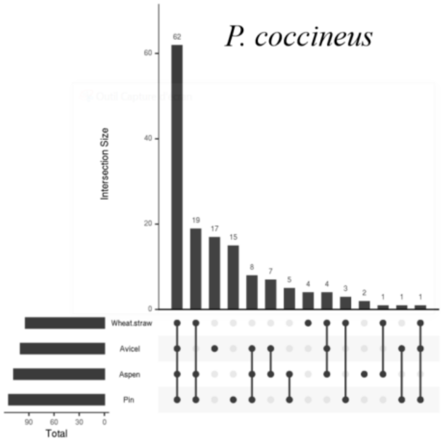

c

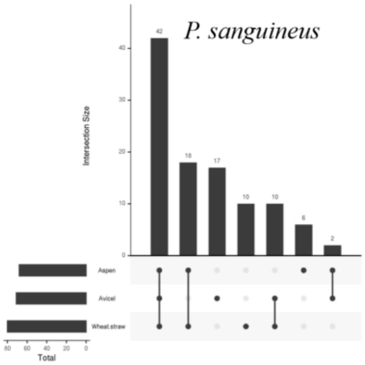

Fig. S24. Numbers of CAZyme coding genes differentially regulated on cellulose (Avicel), wheat straw, aspen or pine, in *Pycnoporus cinnabarinus* BRFM 137 (**a**), *Pycnoporus coccineus BRFM 310* (**b**), and *Pycnoporus sanguineus* BRFM 1264 (**c**). The transcriptome response of P. sanguineus to pine could not be analyzed because of poor quality of the extracted RNAs

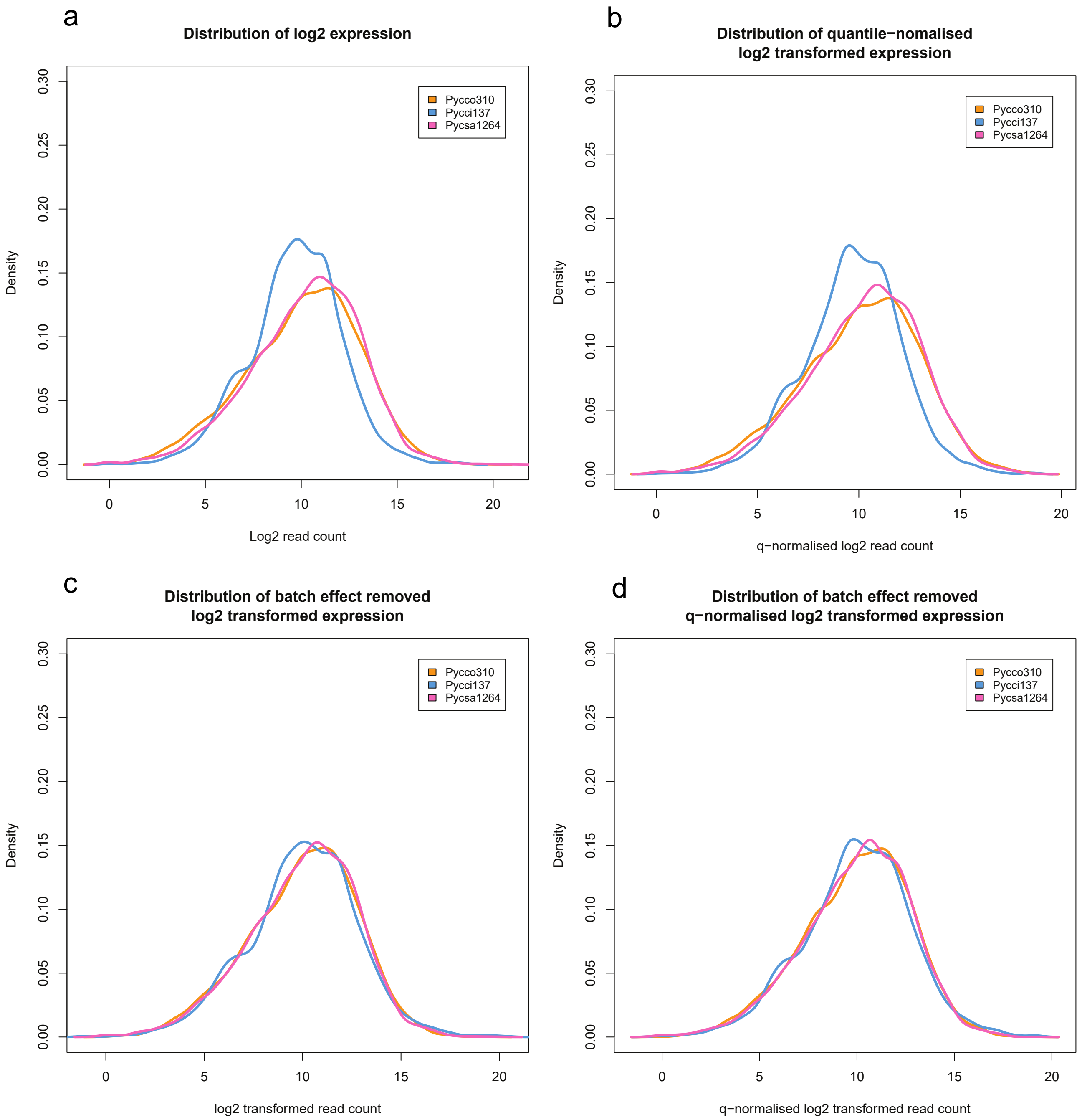

Fig. S25. Distribution of log2 transformed transcript count per gene after normalization with DESeq2 (a), and after additional quantile normalization (b), batch effect removal (c), or both quantile normalization and batch effect removal (d). After the removal of batch effect and quantile normalization, the distributions are almost identical except for P. cinnabarinus BRFM 137 which maintains two peaks possibly reflecting unique biological variability. Pycci: *P. cinnabarinus*, Pycco: *P. coccineus*, Pycsa: *P. sanguineus*

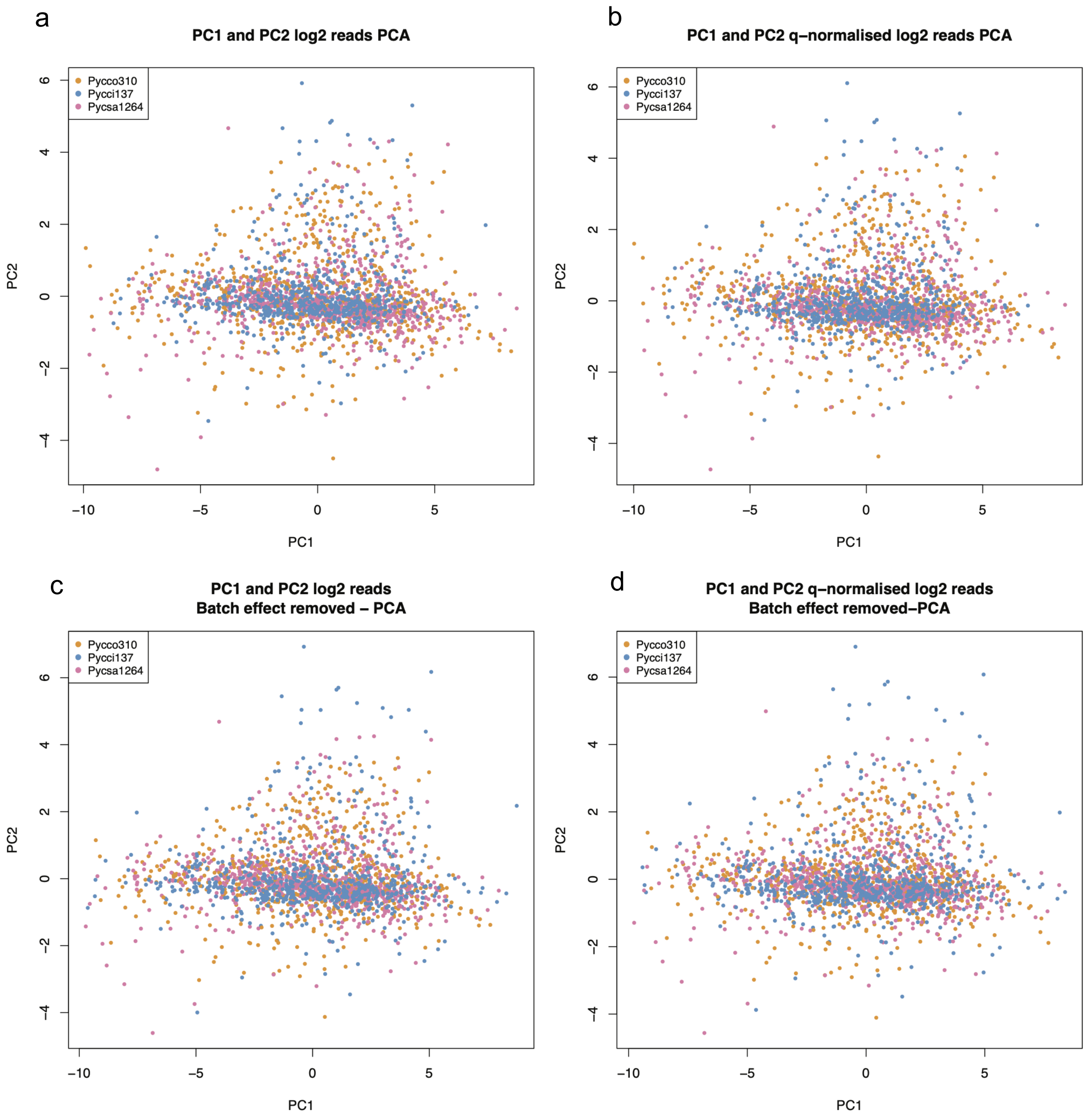

Fig. S26. The first and second principal components from principal component analysis (PCA) using the DESeq2-normalized log2-transformed transcript count per gene (a), and after additional quantile normalization (b), batch effect removal (c), or both quantile normalization and batch effect removal (d). Pycci: *P. cinnabarinus,* Pycco: *P. coccineus*, Pycsa: *P. sanguineus*. The absence of major difference among the four sets of data indicated that the variations in gene transcript levels remained even after batch effect removal and quantile normalization.

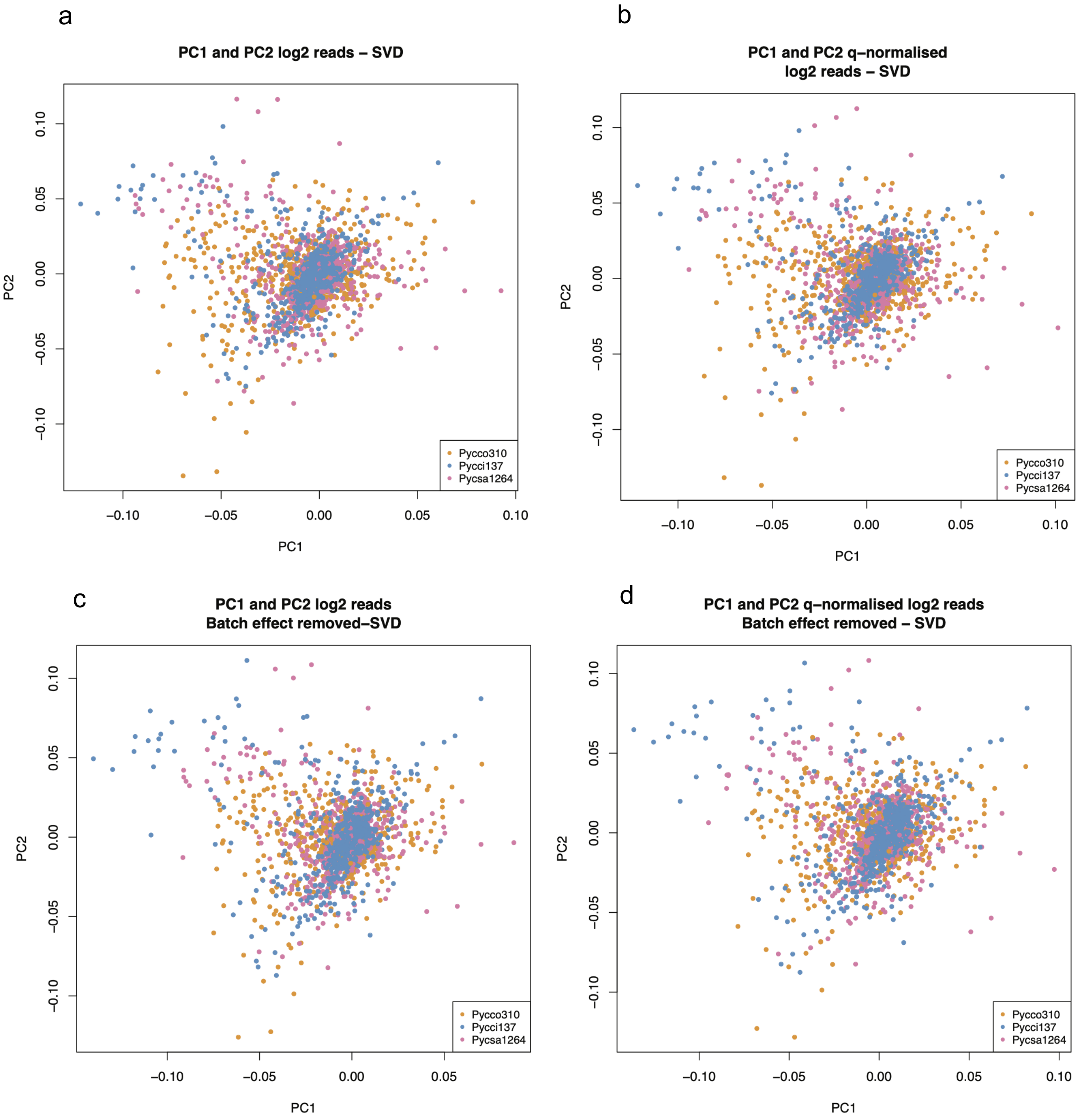

Fig. S27. The first and second principal components from Single Value Decomposition (SVD) with the DESeq2-normalized log2-transformed transcript count per (a), and after additional quantile normalization (b), batch effect removal (c), or both quantile normalization and the batch effect removal (d). Pycci: *P. cinnabarinus*, Pycco: *P. coccineus*, Pycsa: *P. sanguineus*. The absence of major difference among the four sets of data indicated that the variations in gene transcript levels remained even after the batch effect removal and quantile normalization.

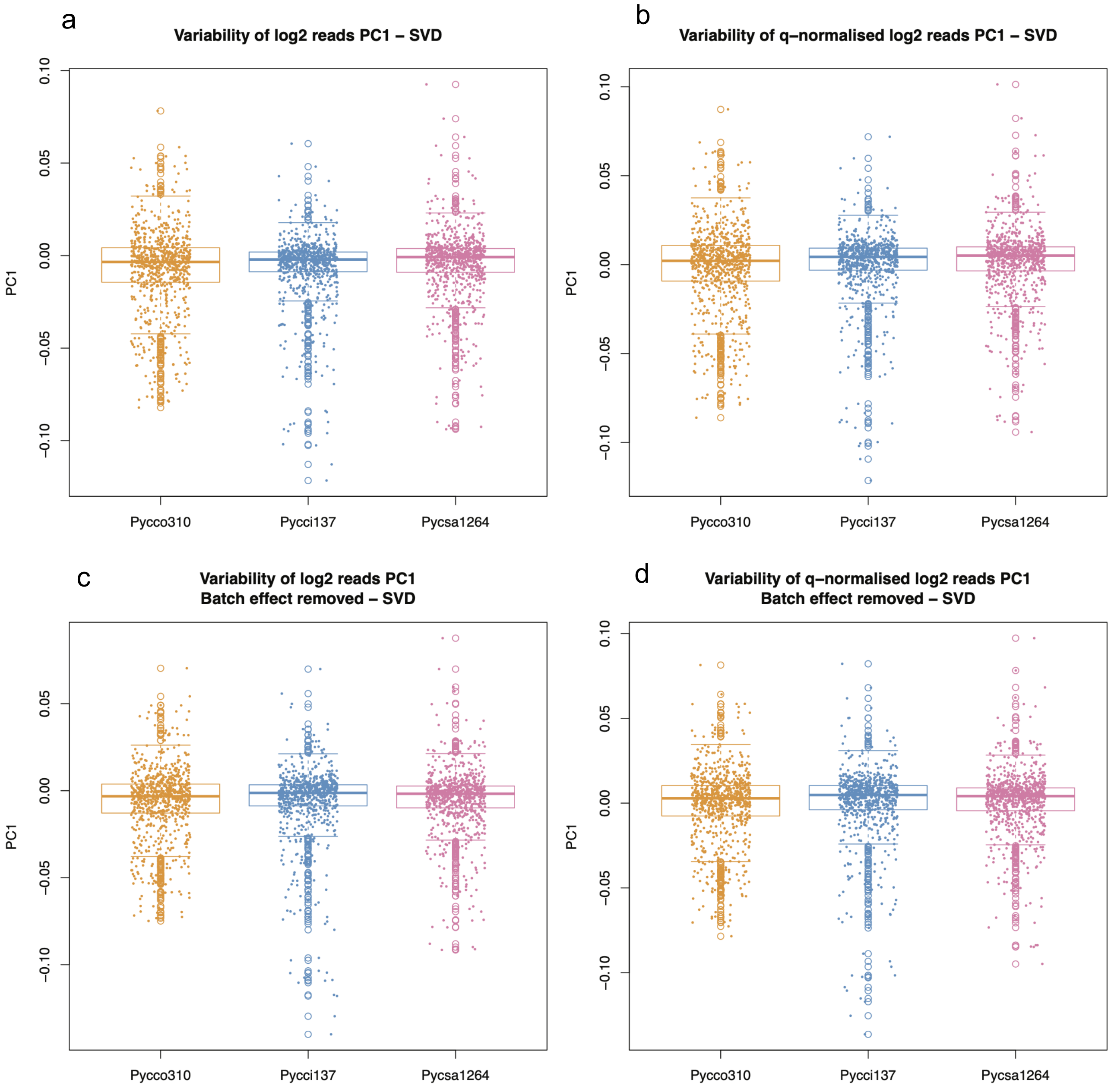

Fig. S28. Distribution of the first principal component from Single Value Decomposition (SVD) with the DESeq2-normalized log2-transformed transcript count per gene (a), and after additional quantile normalization (b), the removal of batch effect (c), or both quantile normalization and the batch effect removal (d). Pycci: *P. cinnabarinus*, Pycco: *P. coccineus*, Pycsa: *P. sanguineus*. The absence of major difference among the four sets of data indicated that the variations in gene transcript levels remained even after the batch effect removal and quantile normalization.
